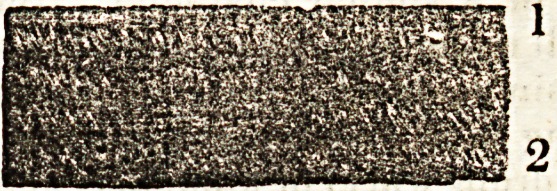# Periscope

**Published:** 1833-01-01

**Authors:** 


### Periscope;

OR,

### CIRCUMSPECTIVE REVIEW.

" Ore trahit quodcunque potest, atque addit acervo."

### I.

### Dr. Hancock on external Stimulants.

In the London Medical and Surgical Journal, for September 15 and 22, there is a paper on the utility of external stimulants in internal inflammations, of which we shall here take a brief notice. Dr. H. first makes some observations on the sensibilities of certain internal tissues, as of the mucous membrane of the stomach and bowels, and then proceeds to the consideration of cutaneous stimulation, as offering a powerful means of arresting the progress of inflammation, " by deriving the blood and humours from internal congested parts, equalizing the circulation, restoring a mutual balance between the powers of the heart and the external capillaries, and so ultimately obtaining the desired resolution." While Dr. H. acknowledges the utility of blisters, he considers them, in severe cases, as very partial remedies, being too circumscribed in their operation. The more general application of friction to the skin with lime-juice and pepper, or vinegar and common salt, in cases of severe internal inflammation, is proposed by our author. The following extract, containing facts rather than arguments, we shall insert here, as it illustrates Dr. Hancock's views on the subject of external stimulation.

" The bird-pepper, (capsicum frutescens) is the kind I have always used, it being more hot and pungent than the other kinds in general. A small handful should be well bruised in a mortar, so as to reduce the seeds and other parts to a fine powder or mass. The powder sold in the shops should not be trusted, it is shamefully sophisticated, like most other articles. Let the former be mixed with an equal quantity of table salt, and add a pint of lime-juice or good vinegar. With this composition the body is to be rubbed entirely over, until it shall produce a sufficient demonstration of excitement and pain.

In a case on Plantation Anna Regina, which I propose to annex to this paper, the patient was so far advanced as to be quite insensible to the stimulus until the second day of the application, and shewed no sensibility till after at least five or six repetitions of the process.

Another instance of the value of frictions with pepper and lime-juice, is that of a boy, George, belonging to Mr. Evans, one amongst several unexpected recoveries from violent inflammation, in which early bleeding had been neglected, or in which it had failed or proved insufficient, although accompanied by blisters and the usual treatment.

Several other instances occurred in my practice, giving further proofs of the efficacy of the pepper frictions in vehement inflammatian of the lungs, as two at Exmouth, an African and a Creole, who had each a violent attack of peripneumony about the same time, and in both of whom a relapse occurred, or an aggravation of symptoms from exposure to air, by opening the windows of their apartments during bad weather.

The effects of the remedy were most strikingly manifested on the Creole, Billy. My attendance was required at 9, p. m. when I found him labouring under an excessive aggravation of his disorder, with difficult respiration, cough, anxiety, hot dry skin, and small jerking pulse.* The frictions had been used some days previously, but were discontinued, owing to the great and decided relief they had afforded. I stood by and assisted in performing the operation effectually by smart frictions over the whole body with a small handful of capsicum, bruised down with some table salt and lime-juice, (we used no weights or measures for this composition.) He said it burnt his skin very much, but most readily submitted, however, as he knew, he said, it would help him, as it had done some days before. On the following day he told me he had not slept much from the burning of the skin, but felt quite relieved of the inward pain. His skin was moist, pulse soft, expectoration free, and he experienced no difficulty in breathing. The application was afterwards repeated a few times more moderately. He had no further symptoms of complaint of the chest, except cough and expectoration, which gradually declined; but the severity of the disease had been such as to cause a serous effusion or anasarcous swelling at the feet, and puffed belly, for which we directed bark, wine, and gentle exercise, and he recovered.†

Soon after this the man named Gilbert, also of Exmouth, was taken with the same complaint, (a very severe peripneumony,) but shortly recovered under the same *regimen*, which the sick nurse said caused him to perspire very freely; and indeed I always found it wonderfully to remove the constriction of the skin in cases where bleeding was inadmissible, and when all hope from this, or other means, were gone by.

The man Mars, on the same estate, recovered from pneumonia, under the same remedy, the year ensuing, and that from an almost hopeless condition. Another case of the same kind, Richard, at Hampton Court, rapidly recovered from a severe attack of peripneumony, under the use of the pepper frictions. I was absent, and the hospital attendants commenced it of their own accord, on the second or third day of the disease, as from having seen its benefits in so many previous cases, they considered it a certain remedy. This was stated to me by Mr. M'Lenan, a very careful and excellent young man, who superintended the sick-house.

I must not fail to note here a previous case, in a Creole boy, called Wellington, on the same estate, in a vehement attack of pleurisy. He came into the hospital, and was bled twice the same day, and blistered on the next, with a smaller bleeding and blister repeated, and glysters, nitre, and antimony, from the first, with barley-water, &c. On the third day his respiration was difficult and painful; the fever and all the symptoms were greatly aggravated; his appearance was ghastly, his tongue dry, and he swooned on sitting up in bed. This afternoon I directed frictions with bruised pepper (capsicum frutescens), and the lime-juice.* It was faithfully executed by the sick-nurse, who, following up this idea, had withal bound up his feet, legs, and arms, with the same composition, as a cataplasm. I found him now, to my surprize, sitting up and free from complaint, with composed, easy respiration, and no fever, and saying he felt no pain. The sick-nurse said he appeared to derive sensible relief at about 11 o'clock the preceding night, the hour in which the fever and other symptoms had been aggravated on each preceding night.

---

* Tongue not dry, however, as in the case of Benjamin, at Sparta.

† Mr. W. Brummell, the proprietor, was then on the estate, and doubtless would recollect these cases, such was his constant solicitude for his people, being far more like a father than a master. I am happy to say, indeed, that such a character is due to very many of the planters.

* Lime or lemon juice, being ever at hand, was commonly employed; but good vinegar is fully equivalent, and is free from the unpleasant clamminess resulting from lime-juice and salt. I am told this practice has been verified by two eminent and skilful physicians of Essequebo, Drs. Bell and Buchanan.

In this case, I remarked that the sick-nurse seized the idea, and commenced the process with alacrity, owing to her having witnessed its success in two former similar cases of forlorn hope, in which I had prescribed the pepper friction, and in each of which it was applied over the whole body, and often repeated. I accordingly found powdered pepper now sticking all over the boy's skin; and I was told that he complained of nothing from this time but hunger and the heat of the pepper. Such an early, sudden, and decisive resolution is not to be expected from the use of the common remedies, even with the best efforts of nature.

A still more surprizing recovery was effected last year (1824,) by the same means, in the boy called Christmas, at Anna Regina. This lad, (about fifteen years of age,) recovered from the most hopeless state of inflammation which I ever knew, or which it is possible to conceive one to escape from. The patient was in the latter stage of measles, and the inflammation of the pulmonary organs was in this case so excessive, that the patient could scarcely breathe, and was nearly suffocated; had constant fever, dry skin, parched tongue, and such a metastasis on the brain as rendered him delirious and perfectly insensible for about two days. Both the pulse and respiration were nearly extinct. His eye was glassy, and covered with a film; his appearance, in short, was most cadaverous; he was totally unable to swallow, and the *raucum* or rattle in the throat withal announced a speedy dissolution.

Mr. Gordon, the hospital attendant, observing that I had omitted this patient in the prescription-book, inquired if any attempt should be made to give him anything, as cold water, his mouth being dry and parched. I told him, that would only add to his distress by impeding respiration, as we had just witnessed, that nothing could possibly be of any avail; but if he pleased he could try the *rubbing* with pepper and lime-juice. Having seen its effects at Hampton Court, he commenced instantly (having two assistants employed), and diligently repeated it over the whole body. The patient was entirely insensible to its stimulus until many applications had been made, but the following day he began to shew signs of pain, and soon afterwards he spoke, and complained of the smarting *over all his skin.* From this period the fever abated, the tongue became moist, and perspiration ensued. On visiting him the next day he was sensible, and raised himself in bed, and said he must have some soup and wine, which were instantly brought him; he actually appeared like one risen from the grave. The fever and all other signs of inflammation had vanished; he complained of no pain; coughed, with a copious expectoration, which at first was dark and fetid. The rubbing was still repeated two or three times a day, and, although reduced almost to a skeleton, his recovery was rapid and perfect. Mr. Frost, the manager, and Mr. Gordon and others, can attest the truth of this extraordinary recovery. It was, however, only by the most diligent and persevering application of the remedy that the patient was saved.

Soon after this, George, a servant of Mr. Evans, in a desperate peripneumony, recovered under the same treatment. Bleeding and various medicines had been used without effect; both the nurse and patient scouted all other remedies, and boldly asserted that nothing but the rubbing saved him. I was called away for some days, but learned on my return, that in this case, as in the others I had witnessed, the free expectoration and perspiration came on, and fever abated, together with freedom of respiration and cessation of pain, in a short time after the frictions were commenced. The patient also soon acquired a remarkable craving for food.

Another most striking case occurred on Sparta estate. The man Benjamin was attacked with pleurisy, or rather peripneumony. He was bled four or five times during the first four days, with blisters and the usual remedies. On the fourth evening, finding him much worse, with dry tongue, great pain in the chest, suffocative breathing, and all the symptoms pointing to a speedy termination, the pepper frictions being resolved on, as the only hope remaining, were promptly applied and repeated several times during the night. He took nothing but barley-water, with a little nitre and an opiate pill. On the morning following he was quite tranquil; the tongue was moist, heat gone, and skin soft and moist. Mr. Campbell saw him well rubbed again yesterday (Oct. 14th). He is now out of danger.

I have passed unnoticed many other cases of less importance, or of less severity.

In similar cases, when remedial treatment can be applied early, the lancet should be employed and the usual auxiliaries, as laxatives, antimonials, diluents, blisters, &c. We ought not to fritter the time and life of a patient under the use of single remedies, as, when a formidable enemy is within the walls, we should not put forward a force in single file to repel his approach, and thus trifle till he gets possession of the castle. Yet, how common is it in physic to trust, as we may say, to a single piquet guard, until the breach has become irreparable.

In fine, I have found the above practice most effectual and decisive, altogether I may say, without a parallel, not having been disappointed in a single instance; and it has been to me a source of high gratification to see patients brought round, under its use, from the most desperate conditions, and such as I had ever before considered to be utterly past recovery."

---

### II.

### Fear of Contagion.

We have heard of numerous instances of unparalleled selfishness, terror, and even brutality, brought forth by the dread of cholera; but we shall content ourselves, at present, with the following melancholy examples, extracted from an Ayrshire paper, and vouched for by a physician of the first respectability in this country.

"Within a few days after the first appearance of Cholera at Air, the Postman was seized by that fatal distemper at Girvan, and there abandoned, under the prevailing panic of contagion, by all but Samuel Wallace, a poor and humble friend, who, like the good Samaritan, carried the sufferer home, and there administered unto him until he died, and afterwards got a coffin and buried his body. But for these brave and generous offices of christian and brotherly benevolence, his own recompense from his panic-struck neighbours and townsmen, was to drive himself from his own home and family, and compel him to wander until the Board of Health in that populous Town assembled and provided a lodging for him, remote from his own family, there to abide, while their own alarm for contagion should last.

Afterwards, on the evening of Tuesday, the 4th inst. the attention of Dr. M'Tyer, Physician in Air, was, when travelling to Maybole, drawn to a poor woman lying on the road-side, a little way beyond the village of Slateford. Upon examination, he apprehended that she might have an attack of cholera, and directed the people at hand to convey her to a place of shelter, give her a hot draught and warm her by all possible means, while he should hasten to Maybole for medical remedies. The Surgeon there to whom he applied followed him back to Slateford with these as soon as possible, and, on arriving at this village, was directed to a small comfortless byre, where he found the poor woman lying on straw, with little covering, and Dr. M'Tyer, with John and James Finlay, two humane inhabitants of the place, who had admitted her, busy in doing all they were permitted to attempt for her relief. Medicine was then administered with a little warm toddy. It was with great difficulty this could be at all accomplished. Dr. M'Tyer was then under the necessity of proceeding on his journey.—The villagers, insane from terror of contagion, refused to give the least assistance, or even to approach the patient, and the Surgeon had the greatest difficulty in obtaining any means of restoring warmth to her body, by external applications, aided then by the two Finlays, alone and while he had dispatched one of them to Maybole, he and the other were assailed by a torrent of abuse, and threats of personal violence, and even of burning the house in which they were with the sufferer, if they should not instantly remove her from Slateford. It was in vain the surgeon attempted to reason with them on the inhumanity and absurdity of their conduct, in forcing her from a place, in which all the danger that could exist from her continuance there—if danger there was—had been already incurred. He was overpowered by a multitude of voices of men and women, repeating their threats, with the additional one of 'dookin of him in the burn,' if he persisted. He was thus reluctantly forced to yield; and Finlay, having placed her on a wheelbarrow, removed her a short distance up the road leading to Maybole. Mrs. Hutchison, a blacksmith's wife, had offered the accommodation she could afford, but they still remained relentless, and would not permit her. She gave, however, some blankets, and her husband furnished hot plates of iron, which were found to be well-adapted for the purpose—for, with all the disadvantages of having only a wheelbarrow, some boards, and a little straw for her bed, and the canopy of the heavens for a roof, the surgeon and his few assistants had succeeded in restoring, in a great measure, the natural temperature of her body, and in diminishing the spasms and other symptoms. The Board of Health, too, having been then made aware of what had happened, the Rev. Mr. Grey, Minister of Maybole, and others of its members, lost no time in hastening to the spot. It was their opinion, as the woman had come from Kilmarnock, and as they, therefore, could not think of adopting the apparently unjust and inhumane step of sending her back to Air, that she should be conveyed to the hospital which had been provided, about a quarter of a mile from Maybole, and this could easily have been effected without having the least communication with any part of the town. But for this well-meant proposal, they met with much abuse from a mob of the inhabitants, and received from them the intimation, that they were determined to resist by violence any attempt to carry her to that place. As the people of Slateford, too, had equally opposed her introduction into any of the houses there, no course remained but to send her to Air, or leave her to perish. Accordingly, a horse and cart having been with difficulty procured, they wrapped her up as comfortably as they could, and dispatched two men with her, bearing a letter from Mr. Grey to the Medical Attendant at the Cholera Hospital in Air, where she was admitted, but then so much too late, that she died in the course of that morning.

It is, Sir, in sorrow, not in anger, and against the delusion, which is the sole cause of such enormities, and not against those deluded individuals who have been the misled and ignorant perpetrators, that I wish to bring the facts under general consideration. For I could gratefully bear my personal testimony in unison with much that is far higher and more honourable to the merits, and loyalty, and charity of the people, in these very communities where they took place, in many, I might even say in all, the former times of trial I have witnessed."

The scene acted at Dawlish was nearly as bad. A man, of the name of Vicary, was dragged by force into a cart, and sent 10 miles, to Exeter, labouring under a violent attack of cholera. He died soon after his arrival. It is a consolation, that a coroner's jury returned a verdict of manslaughter against three men in authority at Dawlish!

---

### III.

### Dublin Fever Hospital — Medical Report—January, 1831, to January, 1832. By Dr. P. Hackan.

The above Hospital is an institution of great utility, especially in such a place as Dublin, where fever so much prevails. It appears that cases of fever, connected with latent pneumonia, are occasionally met with in the fever hospital, and are not a little puzzling.

"Here we have symptoms of pectoral oppression, without cough, (at least with very trifling cough,) and considerable fever, joined to unusual prostration of strength, and rather a small labouring pulse. The stethoscope and percussion afford, in such cases, valuable assistance, pulmonary congestion and obstruction being, with their aid, no longer a matter of doubt. The pulse is accordingly found to rise and fill under the cautious use of the lancet. But here, as in most cases, the physician should, if possible, not order the letting any particular quantity of blood, but be entirely guided by the effects produced, and the relief afforded."

Of late, patients have not been able to bear loss of blood so well as in former years. Dr. H. attributes this to the deteriorated condition of the poor. We are of opinion that it is owing to some unknown state of earth or air, for the same inability has been observed all over Great Britain, and not among the poor only, but among all classes of society.

"The history of these fevers generally commences with despondency, the sad attendant on ruined circumstances, or want of employment; this too often leads on to occasional intemperance, but the destructive habit soon grows, and becomes the fruitful parent of inveterate disease, as well as moral degradation.

This subject leads me to make a few observations on *delirium tremens*, that form of fever well known under the vulgar, but not inapt appellation of whiskey fever. That we meet with this disease oftener now than formerly is certain, but the cause of its more frequent occurrence is not so evident. It may possibly arise from the increased consumption of spirituous liquors by the lower order of people. But this can explain it in part only, as persons of the middle class, among which the disease is also more prevalent now than formerly, are, on the other hand, less addicted to spirituous potations in the present than former times. It appears to me that, in endeavouring to account for this fact, we must chiefly look to the altered state of society in our age, the increase of luxury, the sedentary habits of men in business, added to the anxious cares attendant on mercantile speculation; the immoderate use of tea, coffee, and tobacco, and to other causes, all tending to lessen the tone of the system. The irritability, then, and laxity of fibre produced necessarily under such circumstances, seem to be the great predisposing cause, although 19 out of twenty times the exciting cause is some recent excess, or, as it is usually termed, hard living. Some cases, however, of delirium tremens occasionally occur, (and I myself have seen a few,) which could not have been caused by intemperance, the contrary being ascertained clearly, and the habits of the patient being abstemious.

The pathology of this disease is extremely interesting to the medical inquirer. The highest nervous excitement co-exists generally in the commencement, with the greatest debility of the sanguiferous system. The former produces the most extraordinary *spectra* to the mind's eye of the patient, and very frequently delirium of a furious and unmanageable kind, requiring the use of the strait-waistcoat. The latter is manifested by a small, feeble, compressible pulse. It differs essentially from typhus, which resembles it more than fevers of another type. If fatal, it generally runs its course in a week—the skin is cool, oftener cold and clammy, the patient incessantly talks to imaginary by-standers, the pulse is small and rapid, subsultus tendinum so constant, as sometimes to render it difficult to feel the radial artery. General sinking, convulsions, and a deceitful calmness of manner usher in death. Should the fatal event not occur in the early stage, the danger blows over, and the case merges into one of ordinary fever. Our chief reliance, from the very onset must be on the free use of stimulants, wine, camphor, and opium. Woe to the unfortunate patient whose delirium is mistaken for that arising from meningitis! Two such fatal mistakes came to my knowledge during the last year. One patient was a man of robust frame, and of excessively intemperate habits. His delirium had been violent, which was the unfortunate cause of his being largely bled from the temporal artery. The other, a female of slight appearance and delicate habit, the mother of a large family, was, I understood, attacked with fever about eight or nine days before I saw her; delirium supervened; she could with difficulty be kept in bed; blood was freely taken from the head, and her manner became more calm. But in this, as the former case, the calm was deceitful, as the fatal event soon proved. It would, however, be a great mistake to conclude, that in no case of delirium tremens should blood be taken from the patient. On the contrary, reaction in young robust subjects sometimes takes place after the third or fourth day, to that degree, that moderate local bleeding is not only useful, but necessary. The rapid tremulous pulse, and clammy skin, and subsultus, are found to give way to a hard pulse, and warm dry skin, and flushed countenance. At this period, moderate purging becomes still more necessary. It is really sometimes surprising to see what a quantity of dark offensive matter is discharged for some days, demonstrating the great extent over which morbid action of the liver, and of the membrane of the intestinal canal, takes place. In such cases, then particularly, we must not confine ourselves exclusively to stimulants and opiates, nor push the use of these too far. Following the plan of cautiously combining purgatives with these, we may generally expect a favourable result."

Our author has come to the conclusion, that fever does not always depend on topical inflammation, notwithstanding the doctrines of Clutterbuck and Broussais. We shall conclude with the following quotation.

"Next in absurdity to this is, in my opinion, the specious doctrine of great simplicity of practice in fever. It is certainly very convenient for medical men of limited experience, or idle habits, or destitute of practical acumen. Such persons will sometimes talk very fluently of disease, but when brought to the bedside of the patient, it is surprising how far they sometimes mistake his position. The true character of the pulse, the proper time, manner, and measure of depletion or stimulation, the tracing disease through its various channels to its seat,—these are subjects about which they, in practice, blunder grossly, however confidently they may speak, or even lecture on them. Among this class of practitioners we certainly cannot place Dr. Elliotson, a physician of great talents and deserved eminence, to a statement from whom in a clinical lecture on his treatment of fever, published in The Lancet, I beg leave to allude. An erroneous doctrine countenanced by such a man must necessarily produce evil consequences, his opinion being justly entitled to the serious consideration of every medical man, and decidedly influencing many young practitioners who look up to him as a teacher. He states that his practice in simple fever is almost always successful, and that it entirely consists of a purgative at first, then a combination of calomel and opium with occasionally tepid ablutions. No one will, I believe, dispute, that this plan may be successful in slight and favourable cases, but in the management of dangerous fevers few experienced physicians will agree that they find it such plain sailing, as from such a statement might be supposed. Are they not often obliged to vary their course not only in different subjects, but even in the same case, according to the stage of the disease, the organs affected, the patient's constitution, and other considerations? In confirmation of this, I might refer to a lecture of Dr. Elliotson himself, more lately published, in which he declares that he found it necessary to order wine for patients then labouring under the first stage of fever. We may then safely conclude, that no plan of treatment is applicable to every case, notwithstanding the theories with which the history of medicine abounds. In making this assertion, we do not disparage the great merit of some of the authors of these, for instance of John Brown, whose talents must be admired, or of the modern Broussais, whose important services to medicine entitle him to the highest praise. From the foregoing observations, it is almost unnecessary to remark, that our treatment of fever in this hospital is not conducted on one invariable rule. In a great proportion of cases, emetics, bleeding, purgatives, diaphoretics, and stimulants, are used in the order now stated; but, in some cases, to adjust these remedies to the disease, and to balance contrary indications, is no easy task. As success is, generally speaking, a criterion of good management, I do not hesitate to challenge a comparison of our hospital practice with that of any other Institution whatever. The proportion of recoveries to deaths would be infinitely greater, were we to exclude from the latter those which occur during the first two or three days after admission. This allowance might fairly be made, as it is obvious to medical men, that in general no human aid could, in fevers, have warded off the fatal blow two or three days only before death. For my part, without descending to an idle boast, I confidently refer to my hospital journal, (and I make no doubt my colleagues can do the same,) to prove, that in cases of simple fever, not one in fifty cases proves fatal, of such as come under my care before the fifth day. The great majority of those who die in this hospital, are such as are admitted in the last week of fever, or who labour under chronic disease of the heart, lungs, or bowels, the *sequelæ* of former attacks."

---

### IV.

### Dr. H. M'Cormac on Cholera.

The public in this country was a good deal surprised at the comparatively small mortality of the epidemic in Belfast. The pamphlet published by Dr. M'Cormac, who treated 934 cases, will shew the methodus medendi pursued in the above-mentioned town.

"It is of the utmost importance to arrest the purging and vomiting with the least delay; for the fluid discharges are the serum of the blood, and cause weakness and death, as if the blood itself were drawn away. I have often seen a patient pass, in a very short time, a gallon or more of this fluid, which, not long before, was circulating as part of the living blood. This discharge will not cease of itself; it continues till it brings the patient to the verge of death. It is almost the same, as if so much blood streamed from open wounds. I never knew a case in which an individual attacked with Cholera Morbus, grew spontaneously well—the diarrhœa always appears to go on to the production of collapse and death, unless medical aid be interposed. The reader will then see the necessity of losing no time, and he will not, therefore, wonder when I tell him, that I do not wait till the discharges and the cramps have made the patient cold and blue, but commence without the loss of a moment, to give the most powerful medicines. These medicines, powerful as they are, cannot be productive of the least injury; but neglected or inefficiently treated Cholera, surely leads to death. The following is the mode of treatment which I follow, specifically laid down :—

If a person between ten and sixty, whether male or female, not much broken down or previously debilitated, be seized with the disease, whether with purging and vomiting, or purging, vomiting, and cramps, I immediately take away from the arm, if the pulse be not weak and thready, from ten to thirty ounces of blood, the quantity being regulated by the strength of the individual and the effects produced. To a man or strong woman, I give, while the arm is tying up, a scruple of calomel, with two grains of opium, both in powder, and washed down with a mixture of some diluted ardent spirits, as gin, whiskey, or brandy, united with from forty to sixty drops of laudanum. To an ordinary woman or weak man, I administer half this dose; to a child the quarter, and to an infant the eighth part. If the medicine be thrown off, in whole or in part, the whole or a part of the same dose, as it may appear, must be immediately repeated. In two or four hours, or every four hours, the medicine must be repeated, if the symptoms are not subdued. In this case, general directions alone can be given: the whole dose may be given again, the half only, or the fourth part, according to the urgency of the disease; in general, however, I find five grains of calomel, and one grain of opium, sufficient. In this, as in many other particulars, the practitioner must use his own discretion. If the complaint be early treated, it will generally be checked after the first or second dose—very frequently a single dose extinguishes it at once. Time after time, patients have been brought in with almost incessant purging, vomiting, and cramps; yet hardly have they been bled and swallowed their medicine, when all these symptoms have vanished, sleep came on, and the patients have awakened after some hours, weak but well. In most instances, however, the medicines require to be repeated. Sometimes the symptoms will continue in a much diminished form; at others, the disease will make efforts to re-appear, after the stimulus has been exhausted. In both cases, the repetition of the medicine will produce the desired results.

In some very weak, aged, or broken down persons, the system has rallied for a moment, and then sunk; in this last case, medicine ceases to be of use —nature is exhausted—the leverage of life is broken. We must never, however, presume this to be the case, beforehand, but continue our efforts, till death itself bid us cease. Some constitutions appear so irritable, that the vomiting will continue for days with intermissions, causing much annoyance to the poor patient. In these cases, I find mustard sinapisms or blisters, applied to the stomach, and occasionally warm-baths, opium in some form, and a little wine negus at intervals, with some soup or tea, having bread sopped in it, sufficient for its suppression. Some patients complain much of a difficulty in making water. These will require, in many cases, the use of the catheter, abdominal frictions, stupes and warm-baths; mild opiates will generally be necessary in addition. With these remedies, this troublesome affection commonly ceases in a few days. Sometimes the mercury will affect the mouth. In this case, port-wine gargles, the warm-bath, careful nourishment, and opiates, will soon cause this symptom to disappear. Every patient has invariably recovered, whose mouth has been affected; and although I never try to produce this result of mercury, as some practitioners recommend, I do not find the recovery in the least retarded by it. Such a trifling and occasional inconvenience in the employment of so admirable a remedy, when the result is the salvation of human life, and a complete victory over a formidable and malignant disease, is not to be regarded for a moment. The patients will sometimes be troubled with griping pains in the stomach and intestines, during their convalescence; but wine and opium, and now and then a warm-bath, quickly disperse them. If the bowels should prove costive, from ten to thirty grains of the compound powder of jalap may be given, or from half an ounce to an ounce of castor oil, with an ounce of peppermint-water and twenty drops of laudanum. Sometimes I make the attendants administer a simple enema or injection of gruel with or without castor oil; for I have more than once seen the use of purgatives bring back the serous purging of the complaint, to the great danger and prejudice of the patient. Indeed, during the prevalence of Cholera, purgatives should be very carefully dealt with, when it is necessary to use them. They are almost always improper, when purging actually exists—as for common salts and other saline purgatives, they should not be touched. In two fatal cases, which occurred in private practice, castor oil in the one case, and castor oil and salts in the other, were most improperly taken by the patients, when they felt the disease coming on them. And I may here be permitted to remark, that very many lives indeed, might be saved, by people calling in instant advice, when affected with unusual purging, whether during night or day. I shall now, before I detail the diet during convalescence, and the general precautions proper to be put in practice by the community during the prevalence of Cholera, as well as the steps to be taken by those who are remote from medical aid, proceed to state what I have to say relative to the treatment of collapse.

I never found it of any use to bleed the patients in collapse, even so far as it is practicable to do so. The patient in most instances is cold, and requires to be heated with the warm-air bath, a simple contrivance, consisting of a few half hoops, the end ones of iron, stayed together by longitudinal braces. A solid piece of wood is adapted to one end, pierced with a hole, through which a curved tin tube, four inches in diameter, proceeds, and which serves to transmit the heated current of air, impelled upwards by the flame of some spirits of wine, held in a tin cup, supported by a rod on which it slides. By the use of this apparatus, the patient may be well heated in from ten to twenty minutes. The same object, however, is readily effected by bladders or bottles filled with warm water—pillow-cases, containing each a few pounds of hot salt—or hot bricks wrapped up in flannel or other cloths. The heat of the bottles may be tempered in the same manner. As soon as the process for heating is put in operation, the patient must receive a scruple of calomel, two grains of opium, and a drachm of laudanum, mixed with spirits and hot water; an injection, composed of half a pint of starch or gruel at blood-heat, and containing a drachm of laudanum, is to be given at the same time."

---

### V.

### Influenza Epidemica.

The history of epidemics presents a curious phenomenon, which has seldom been wanting and still seldomer reversed—namely, their course from east to west. Sometimes this course is slow, as in plague, cholera, &c.; at others, it is very rapid, as in influenza. The following brief delineation of the march of a recent influenza, must tend to convince us, that there is something analogous in all epidemics—some one common agent or cause, which gives rise to the westward course of them all.

"The influenza, from which we are at present suffering in this country, (America) prevailed in China during January, 1830, and in Manilla in Sept. of the same year. It preceded the cholera both in Russia and Poland, but we do not know the exact period at which it appeared in those countries; it prevailed in France during May and June last, in England during June and July, and about November began to prevail in this country. Mr. Lawson, surgeon of H. C. ship Inglis, states that in China the symptoms of the disease were, 'pain in the head, more especially over the frontal sinus, cough, discharge from the nose, sense of rawness in the throat and chest, rather than severe pain, great prostration of strength; in some of the cases there was pain in the epigastrium as well as across the loins; with severe aching pains in the limbs, pulse frequent, but generally soft. The febrile symptoms, in most cases, had entirely subsided on the third or fourth day, and the cough, in the majority of instances, in about the space of a week from the commencement. There were, however, some exceptions, where a troublesome cough remained for two or three weeks.'*

Mr. George Bennett gives the following description of the disease as it occurred at Manilla. The commencement of an attack from this disease was with a general lassitude, followed by pains referable to the lumbar region, and in some cases with muscular pains over the whole body; an increased secretion of mucus in the nose, as also in the fauces and bronchiæ; intense headache, principally referable to the frontal sinuses; tongue white; eyes suffused with tears; skin hot; much thirst; a rawness of the throat; cough, particularly troublesome at night; in some cases a restriction across the chest was much felt; appetite impaired; bowels generally costive; quick pulse; and in those of very plethoric constitutions, (in whom the attacks are severer,) a very

* Medical Gazette, Vol. VIII. p. 525.

quick full pulse; flushed countenance. The symptoms varied in intensity in different persons; some having the fever and concomitants so high, with a flushed countenance, and a pulse so full, strong, and quick, as to have almost induced me to resort to venesection; other cases again assumed a very mild character, but in a very slight degree incapacitating the patient from pursuing his occupations. The patients were worse during the night than during the day, the accession of the fever and cough being much greater at that period. After the patients had in some degree recovered, a troublesome cough, attended in most cases with much expectoration, remained, and in delicate constitutions may be apt to lay the foundation of pulmonary disease.

In Paris the disease is described as commencing with coryza, head-ache, lachrymation and sneezing; dryness, pain and tickling of the throat; difficulty of swallowing and cough, sometimes dry and at others accompanied with expectoration of clear, or thick mucus. To this first series of symptoms a more or less marked derangement of the stomach and bowels was added. There was loss of appetite, and sometimes nausea and vomiting, but when vomiting occurred it was generally after violent fits of coughing. There was also lassitude and feeling of soreness in the limbs and more or less depression of spirits. There was often no fever; when it did exist, it was commonly continued, moderately intense, and terminated with abundant sweats. In some plethoric persons the cerebral symptoms and those of pulmonary congestion were very intense. In one case delirium continued for four days, and in another the head-ache was extremely violent, and yielded only to the repeated application of ice. In a few cases there were abundant and obstinate hæmoptysis. Previously to the appearance of this epidemic in Paris catarrhs had become frequent; as the hot weather came on, the catarrhal state of the respiratory apparatus almost entirely disappeared, but vomiting and diarrhœa, which had been observed in a few of the patients who were affected with the influenza, became more frequent and violent; in many persons there was no vomiting, but only dysenteric symptoms, in others a simple bilious flux. Finally, during the month of August, spasms of the limbs and body were joined in some patients to the symptoms just described, constituting sporadic cholera.*

In England the epidemic is stated to have commenced like a common cold, but the constitutional disturbance was much more considerable than the catarrhal symptoms seemed to account for. 'Running at the nose and eyes,' says the Editor of the London Medical Gazette, 'with racking pain over the brows, are the most frequent local affections; which, however, are often accompanied or succeeded by cough, and sometimes by nausea and an irritable state of the bowels. The feelings of languor, oppression, and discomfort, are always considerable, and sometimes very distressing; being occasionally attended with anxiety at the chest and tendency to faint. Some have severe muscular pains, of a rheumatic character, with tenderness of the integuments. The attacks generally last from two days to a week, passing off with perspiration, and, in the worst cases, leaving the patients considerably reduced.'† Dr. Burne says that the head is heavy and painful, and is jarred distressingly by the paroxysms of coughing, which gives the sensation as if the head was splitting.

In this city the disease has usually come on with catarrhal symptoms, generally attended with intense head-ache and gastric derangement. Delirium has been by no means an uncommon attendant on the disease, and in some cases the prominent affection has been that of the head, and even occasionally it has been a fatal one. Great depression of spirits has been occasionally present. When the catarrhal symptoms have predominated, there has often been much pain in the side, with rheumatism of the intercostal muscles and sometimes of the loins and limbs. The cough has

---

* Gazette Medicale, June 25th, 1831. and Sept. 10th, 1831.

† Medical Gazette, July, 1831.

been usually dry at the commencement, and sometimes occurring in paroxysms; the efforts to cough cause great racking of the brain.

We have seen the disease commence with vomiting and purging, like cholera, followed by a catarrhal affection of the respiratory mucous membrane, and rheumatic pains in the chest and limbs. The tongue has been in almost all cases exceedingly loaded, but there has been little or no tenderness of the abdomen on pressure.

In the treatment, venesection has been nearly always demanded, and sometimes it has been necessary to repeat it two or three times. After general bleeding, local depletion by cups along the dorsal and lumbar vertebræ, has been exceedingly useful in relieving oppression of the chest, when it was present, or the rheumatic affection of the limbs. Cups to the back of the neck and to the head, relieved the cerebral symptoms. Gum water, rice water, lemonade, and the like, for sole diet, and mild laxatives have, for the most part, completed the cure.

Treated upon these general principles, patients have nearly always speedily convalesced, and their recovery has been complete. Where, on the contrary, active depletion has been neglected at the commencement, the case has frequently terminated fatally; or engorgements of some of the viscera, particularly of the lungs, have taken place, and the foundation has been laid for incurable pulmonary disease. Our space does not permit us to enter into any further details respecting this complaint at present, but we shall probably hereafter recur to it."*

---

### VI.

CLINICAL OBSERVATIONS ON THE EXHIBITION OF OPIUM IN LARGE DOSES, IN CERTAIN CASES OF DISEASE. By Dr. W. STOKES, Physician to the Meath Hospital, Dublin.

IN the second number of our Dublin contemporary there is a paper by Dr. Stokes, on the effects of opium in inflammatory diseases, which deserves notice. Various practitioners in hot climates have demonstrated the utility of opium in conjunction with other remedies, in subduing inflammatory affections, as dysentery, hepatitis, and fever; while Hamilton, Armstrong, and many others have done the same in this country. Dr. Armstrong, indeed, and the late Mr. Hayden, proposed opium *alone*, in abdominal phlegmasiæ—and Dr. Stokes appears to follow in the same track.

"The first form of disease in which the use of opium appears peculiarly advantageous, may be stated to be that of *Peritonitis occurring under circumstances where blood-letting cannot be employed.* Now, the following are the circumstances under which I have seen this condition of parts to arise:—

1st.—Peritonitis arising from the escape of fæcal matters into the peritoneal cavity, through a perforating ulcer of the intestine.

2nd.—Peritonitis arising from the bursting of an abscess into the serous cavity.

3rd.—Peritonitis occurring after the operation of paracentesis in debilitated subjects.

In addition to these cases which I have myself witnessed, we may add, that low typhoid peritonitis, occurring after delivery, as described by Drs. Cusack and Gooch; and the peritonitis which results from rupture of the intestine, induced by external violence."

There can scarcely be a more appalling accident than perforation of the intestine and escape of its contents, among the bowels, with the rapid and dreadful peritonitis that ensues. As this accident generally supervenes on a wasting disease, as intestinal or typhoid fever, there is nothing to be gained by bleeding, as the disease runs a rapid course, and prostration of strength exists from the very beginning. We have then, two indications (according to our author) to pursue—first, to support the strength of the patient;—secondly, to prevent the further effusion into the

---

* Philadelphia Journal of Medical Sciences, No. XVIII.

peritoneal cavity, so that nature may have time and opportunity to surround what has already been extravasated, by boundaries of coagulable lymph. Opium, in large doses, is the remedy on which our author depends. Two cases are condensed from the fifth volume of the Dublin Hospital Reports, in one of which great relief was obtained, but the advantage was lost by exhibiting an aperient too soon, when all the bad symptoms recurred, and the patient died. The next case was more successful, and our readers will find it quoted in No. XXVIII. of this Journal, page 541. There can be little doubt that, in the case alluded to, there was perforation of the intestine, with consequent extravasation of its contents. The power of bearing opium (which in this case was given to a great extent) without injury to the nervous system, is very remarkable, and is only explicable by the *pain*, on which the anodyne effects of the opium appear to be expended.

Since the occurrence of these cases, our author has used the same remedy in examples of ordinary peritonitis, where bleeding was inadmissible; and he assures us that he has had no reason to change his high opinion of its powers. In one case where death took place, the opium was borne without the slightest inconvenience. In two cases lately occurring in the hospital, the same treatment has been pursued with the most striking benefit. A case is quoted from the Dublin Hospital Reports, of hepatic abscess, in which the practice here recommended was first tried.

"I have now detailed cases illustrative of the utility of opium in large doses in peritonitis, arising from the introduction of fæcal and of purulent matter into the serous cavity. I would further propose it as a remedy in cases of rupture of the bladder and uterus, and in peritonitis after the operation for strangulated hernia. I am at present trying its powers in a case of recent pneumothorax from pulmonary fistula. In two cases of peritonitis after tapping, where the patients were in a low state previous to the operation, the exhibition of these large doses of opium, without drawing any blood generally or locally, has succeeded in my hands in removing the disease and saving the life of the patient. This appears to be a peculiarly appropriate case for the opium treatment. The patients are generally cachectic, either from original constitution or the disease. They almost all labour under visceral disease and obstruction, and a state of collapse commonly follows the operation. All these circumstances go strongly against our use of the usual antiphlogistic treatment; it was in a case of this kind which occurred in the old Meath Hospital, in the year 1822, that Dr. Graves first ascertained the great importance of opium in this disease. A woman had laboured under ascites, for which she was tapped; the operation was followed by the symptoms of peritonitis. When Dr. Graves saw her, she appeared in the last stage of the disease, she had constant vomiting, hippocratic countenance, cold extremities, the belly exquisitely tender, and the pulse 160 in the minute, and nearly imperceptible. The case appearing hopeless, Dr. Graves determined on merely endeavouring to allay the distressing symptom of vomiting, and administered a drachm of laudanum. The patient soon after fell asleep, and awoke refreshed, with a more warm surface and fuller pulse; the vomiting had ceased. The same remedy was used in smaller doses every fourth hour, and in the course of two days all the unpleasant symptoms had disappeared.

I now proceed to submit some cases of diseases of mucous membranes, where the use of opium has proved efficacious. From having witnessed its utility in cases of inflammatory states of serous membranes, where the inflammation might be termed, for want of a better name, *asthenic;* it appeared probable, that the same condition of mucous membranes might be benefitted by it.

It is now some months since I was called to see a gentleman labouring under all the symptoms of gastro-enteritis, in a severe form. He had considerable fever; thirst urgent; constant smacking of the lips; respiration hurried, *without disease of the respiratory system;* a red tongue, and great tenderness of the belly. The usual treatment was pursued, but the disease shewed great obstinacy, and after six weeks' continuance the situation of the patient appeared hopeless. At this time a violent bronchitis supervened, and so great were the sufferings of the patient from the accumulation of mucus in the trachea, that, on three occasions I left him, never expecting to see him again. It was remarkable, that during this attack, the symptoms of abdominal disease greatly subsided. Under a stimulating treatment he recovered from the bronchitis, only to relapse into his former state. The abdominal symptoms now became still more urgent; the belly swelled from tympanitis; the verge of the anus became surrounded by large and irritable hæmorrhoids; there was extraordinary prostration and constant low delirium. Under these circumstances, a diarrhœa supervened, at first slight, but afterwards so severe as to threaten every day death from exhaustion. A great variety of means were tried, but without avail. At this time, when the patient seemed in articulo mortis, I ordered him a grain of opium every hour; this he took regularly for the first 12 hours, *without any inconvenience*, and he experienced some refreshing sleep. Next day the remedy was continued in the same dose every second hour, and from this time his improvement was rapid; and I rejoice to say, that he is now in the enjoyment of good health."

This was a fair case, Dr. S. observes for the employment of this *heroic* remedy, since all other means had failed. The next case is one to which he confesses he cannot but look back on with pleasure, was that of a patient who was admitted in February, into the Meath Hospital, complaining of sore throat and shooting pain through both ears. His countenance was haggard—voice raucous—body emaciated.

"An extensive and unhealthy-looking ulcer, covered with a whitish matter, was found to occupy the left tonsil, the back of the pharynx, and left side of the uvula. The patient denied having had venereal, but circumstances led us to suspect this; he had, however, been frequently salivated in India for abdominal disease and fevers. He first felt the soreness of his throat six weeks before admission, which was the time when his vessel made the British Channel. We ordered the patient the sarsaparilla decoction with nitro-muriatic acid, and touched the sore with a strong solution of nitrate of silver, which caustic was changed in some days for the butter of antimony. No good effect was produced by these means, the sore extended quite round the uvula, which it rapidly destroyed. The breath became fœtid; the cough laryngeal; the patient's appearance was still worse than on admission; his nights were sleepless, and he complained much of pain in the head.

I now changed the plan of treatment; omitted the sarsaparilla and the lotion, and ordered a gargle of chloride of lime with the internal use of six grains of opium daily, and an increase of his wine. At once the sore began to assume a more healthy appearance; the fætor of breath diminished greatly, and in a few days wholly disappeared. After a short time, in consequence of want of sleep, we increased the dose to eight grains, on which he has been kept since the 20th of February. The sore is now healed, and the whole state of the patient singularly improved."

At the time of writing, there was in the Meath Hospital, a woman who, for an enteritis, had taken 18 grains of opium in 48 hours. Previous to the exhibition of opium there had been retention of urine, which gave way under the administration of the remedy.

"Hitherto I have alluded to the employment either of simple opium, or its tinctures. In Dr. Bardsley's interesting collection of Medical Observations, several cases are detailed, where, in affections of the stomach, the acetate of morphia was employed with benefit. I am persuaded, that it is a remedy of great power, particularly in chronic cases of dyspepsia, where there is much acidity. I was consulted some months back by a gentleman who has led a very dissipated life, and who for the last ten years has been a martyr to the worst symptoms of dyspepsia. About two years ago, he had a violent attack of hæmatemesis, and latterly the stomach had become so irritable, that it was scarcely possible to find any article of diet to agree with him. His sufferings were dreadful. After trying other means ineffectually, I ordered the $\frac{1}{8}$ of a grain of the acetate twice a day. He took the remedy three times in the day, with the most perfect relief. The secretion of acid which had been enormous, was suddenly checked, and the patient in two days declared that he felt better than he had done at any time for the last ten years. His appetite was good, and he foolishly indulged in articles of diet from which he had long abstained. On the fourth day, while in the highest spirits, he became pale; fell, and threw up several pounds of blood. The remedy was of course omitted. In about a fortnight, all the former unpleasant symptoms returned, and his indigestion was as complete as ever. I now ventured on exhibiting the morphia again, but in the doses of the 1-16th of a grain, twice in the day. This diminished dose again produced the same improvement, but in a few days was followed by a return of the hæmatemesis.

The next case, is one of a gentleman who has been for a length of time in the East Indies, and who has become a victim to hepatic disease. He is subject to attacks of pain in the epigastrium, followed by jaundice and fever. These have been treated by leeching, purgatives, and the use of mercury; but during the last attack, his debility was so great, that I did not wish to venture on this treatment. The acetate of morphia was given twice a day. It was commenced on the 26th of December, and continued till the 18th of February. The greatest improvement has been made in this gentleman's state. The pain has disappeared; there is now no tenderness; the jaundice has subsided, and what is most remarkable, his bowels now act regularly without the assistance of medicine. He has gained flesh, and his whole appearance is singularly improved."

We have now only to append the conclusions to which our author has come relative to the exhibition of opium.

"1st.—That in certain cases of inflammation of serous and mucous membranes, where depletion by blood-letting, or other antiphlogistic measures are inadmissible, and the system in a state of collapse, the exhibition of opium has a powerful effect in controlling the disease.

2d.—That under these circumstances the remedy may be given in very large doses, with great benefit and safety.

3rd.—That its effect then is to raise the powers of life, and remove the local disease.

4th.—That the poisonous effects of opium are rarely observed in these cases; the collapse and debility of the patient appearing to cause a tolerance of the remedy.

5th.—The cases in which the utility of this practice has been ascertained, are as follow:—

Simple peritonitis, in a stage where bleeding cannot be performed. Low puerperal peritonitis. Peritonitis from perforation of the intestine; from the opening of an abscess into the sac; or lastly, after the operation of paracentesis in debilitated subjects. Violent diarrhœa, supervening in an exhausted subject. Phagedenic ulceration of the throat, in a similar individual. And cases of chronic gastritis, and gastro-duodenitis in patients exhausted by the long continuance of the disease.

6th.—The cases in which this mode of treatment would be probably useful are,—peritonitis from rupture of the bladder, or uterus, traumatic rupture of the intestine, or after the operation for strangulated hernia.

The last observation which I shall make here is, that in most of these cases, particularly in those of diseases of serous membranes, wine was given in conjunction with the opium; and in all, the patients were supported by a lightly nutritious diet."

This paper is certainly interesting, and the subject important. On this account we have laid a full account of it before our readers.

### VII.

### ST. THOMAS'S HOSPITAL.

### Case of Spurious Hydrocephalus, connected with Psoas Abscess.

The patient (Mary Tyson), æt. 8, from Richmond, having a fair skin, and light red hair, and very much of a scrofulous appearance, was admitted under Mr. Green into Queen's Ward, Nov. 11th, 1830.

It is difficult to obtain any satisfactory history of this case, even from the friends of the child, who seem to have some motives for secrecy, arising from their doubts as to the propriety of her being taken into the hospital, and the poor girl herself is so peevish and fretful, that it is not easy to induce her to answer at all, and when she does make a reply, scarcely any information is communicated.

In the right groin there is a hollow ulcer, of a florid colour, smooth and glistening (no granulations projecting from its surface), about two inches in length, in the oblique direction of Poupart's ligament, narrow in the centre, and decreasing in width towards each extremity; and from it there issues an extremely profuse discharge of healthy pus, in the collection of which the bottom of the sore is bathed. On the right side of the spine, in the lumbar region, a round and deep ulcer exists, also discharging most copiously. This, as well as the former, is said to have followed an abscess which broke spontaneously in that situation, but it looks exactly like the effects of moxa, or caustic potass. The surface is smooth, and the edges quite defined, the granules being minute and florid. We learn from the friends, that the child ailed not three weeks ago, and that, on the ensuing Sunday, she walked the distance of twelve miles. Disease of the spine is not discernible, and they affirm that no application of a caustic nature has been made to the back. The quantity of pus from both sores must be supplied from a deeper source, and this is believed to be an abscess in the psoas muscle. The patient lies on her left side, with the right lower extremity crossed over the other. That on the affected side thus appears shortened, and the trochanter major being much more prominent than ordinary, dislocation of the head of the femur upon the dorsum ilii is simulated. Pulse is quick, at 100—skin hot—face a little flushed.

*Poultices applied to the ulcers.*

Nov. 16th. The little patient is improved in general health, sleeps well, and is free from pain. Pulse 96; skin moist; bowels regular, without medicine. When the poultices are removed from the sore on the back, pus streams from it, evidently having a deep origin, and it seems probable that it communicates with that in the groin, which latter yields an equally copious discharge.

*Ordered porter, a pint daily.*

26th. Child appears still better. A very abundant flow of pus continues from both ulcers.

Dec. 2d. It was yesterday observed that all discharge had ceased from the groin and dorsal region, and that the ulcers had a dry surface and rather prominent granulations. About two o'clock this morning the patient was attacked with violent convulsions, which lasted till five, and have left her perfectly insensible. There is now evidence of serious mischief within the head—the eyes are rolling and displaying a vacant stare, the pupils being dilated to their utmost —pulse 120—skin hot and dry. She does not moan, nor refer to her head as the seat of pain. Has had an enema, which brought away a natural stool. Was seen by Dr. Elliotson about 12 o'clock, noon; he ordered the scalp to be shaved, and a large blister to be applied upon it: also

*Hydr. c. Cretâ*, gr. v. *3tiâ q. h.*

3d. After the third dose of the medicine, and about 11 o'clock last night, the little patient's senses returned in some measure, and she has been since that time sensible. Has some pain of head—the eyes retain their vacant stare, and the pupils continue dilated, but less than yesterday—pulse 120, more feeble, regular—skin cool. The blister was placed over nearly the whole of the vertex, and rose well—stools fetid, and of a greenish colour.

*Vespere.* Is more rational and the countenance is restored to a more natural expression.

4th. About nine last night, the convulsions returned with great violence, and lasted two hours. She, at the end of that time, awoke from them, perfectly sensible, having injured her tongue with her teeth; the convulsive fit is now present, but her motions are not violent. She is moving her head about on the pillow, and tosses her arms in every direction, occasionally moaning—the eyes are turned upwards and staring—pupils dilated—pulse 100, and small—no heat upon the head, or rest of the body. Her tongue is severely injured by gnashing of the teeth, and would be more so, if it were not protected by the interposition of the handle of a spoon between the jaws. Sore in the groin has contracted, and is quite devoid of secretion; that situated upon the back has also diminished most rapidly, and affords no pus, both being quite dry.

*Blister upon the head to be repeated. Pergat c. medic.*

5th. Is now quiet, but has been convulsed in the night. Her countenance is not distorted, and the eyes are fixed in a natural manner. The surface of the body is cool, and the extremities are still lower in temperature. Pulse quick, and more feeble. Bowels are open, and the motions not unhealthy. Mercury is intermitted, by Dr. Elliotson's direction, no effect being produced on the mouth by this remedy.

6th. Has been restless during the night and greater part of yesterday; and early this morning her powers began to fail, and the skin became colder. She now lies in a state bordering on coma, but is conscious of pain, as she has been endeavouring to tear off the blister from her head, which was reapplied yesterday. Her hands are, therefore, muffled. She has just been removed out of bed, and, by this slight passive exertion, her breathing has been hurried, and is performed with much heaving of the chest—skin cold—eyes not turned in an unnatural direction—pupils still dilated, but less than heretofore. Pulse not to be felt at the wrist—bowels open. Dr. Elliotson visited her, and prescribed—

*Quinæ sulph.* gr. iij. *Inf. rosæ co.* ℥j. *4tis horis. Nourishment, in the form of arrow-root and milk, beef-tea, &c.*

Dec. 7th. At intervals she is sensible; there is very little, if any, alteration in her general condition. Bleeding has taken place from the mouth, but not to any amount of importance, and apparently from the fauces, or upper part of the pharynx. Continues quinine, and takes some little nutriment, by dint almost of force.

8th. Has not been convulsed since yesterday. She is sensible, but lying in a dormant state, crying out at times from pain in her head. Pulse 130, very feeble and irregular—tongue blackened, from the hæmorrhage of yesterday. Sordes is collecting about the lips and teeth, it having been once washed off this morning. She has become surprisingly emaciated. *Continue.*

9th. No change has transpired, and she appears in less pain. Constant watchfulness—countenance placid. Very little nourishment is taken.

10th. There is to-day a plentiful discharge of pus, both from the sore in the groin and that on the back, and the patient is astonishingly better. She is perfectly sensible, has very little uneasiness in her head, and her countenance and eyes exhibit quite a different aspect. Pulse stronger, about 110—skin warmer, not dry, bowels open, and the stools have a proper colour.

11th. Patient still better, the discharge continuing freely from each situation.

12th. Has taken food for the first time since the attack. She is quiet, and devoid of pain.

14th. Nearly the same as on the 12th. Has an appetite for meat, of which she has partaken.

21st. Very little change is to be observed. She is on meat diet, and sleeps a great deal.

26th. Takes her nourishing remedies, and her strength is increasing.

28th. A small and projecting superficial abscess has formed, during the last two days, upon the forehead, which, being punctured, nearly half an ounce of ill-conditioned pus escaped. The discharge from the groin and back are undiminished. *Continues quinine.*

1831, Jan. 6th. Patient has recruited in strength very much; takes her allowance of meat, together with a good quantity of nourishment in other forms, and has regained flesh. The abscess on the scalp has healed under poultices. Discharge from the ulcers is quite as abundant as heretofore.

14th. No further change is perceptible.

28th. The discharge from both ulcers continues equally profuse. She, however, consumes plenty of nutriment, and is recovering flesh. Makes no complaint of head, but the pupils are constantly in a state of dilatation.

Feb. 14th. No change has occurred, but the little girl seems more cheerful and stronger.

March 5th. Her appearance is improved, and the appetite good. A discharge of matter, nearly as plentiful as ever, still issues from both places. The sores themselves have very considerably diminished; but sinuses are established down the loins, and from the groin downwards along the thigh. She has not quitted her bed yet, but is perfectly without pain or feverishness, or cerebral disturbance.

20th. The ulcer in the groin has closed, and that in the lumbar region is reduced to the size of a pea. Some small quantity of pus escapes from the latter still, and, when pressure is made above, it comes away in greater plenty. A small sinus apparently exists here, but not below. Patient is mending by slow degrees, and is free from any affection of the head, but the pupils continue dilated.

April 6th. Patient has been gradually gathering strength, and one small sinus above remains, terminating on the back by a minute opening, and the quantity of purulent matter evacuated in the 24 hours is very trifling.

15th. A few days ago, it was tried what power she had over her limbs in walking; but she was barely able to do this, even when the upper parts of the body were supported, by the nurse placing her hands in the axillæ. A thin, watery discharge was observed yesterday from both the former situations, preceded by slight constitutional disturbance, and to-day there is an abundant flow of pus again. The child is considerably weaker than she lately has been, and has lost her appetite in some measure. There are, however, no febrile symptoms now present.

28th. Up to this time, the purulent discharge exists in almost equal excess, but she does not suffer pain from its sources, nor has any recent symptom of cerebral mischief been observed.

May 27th. She left the hospital this day, in much the same condition as by our last few accounts.

Dr. Elliotson is of opinion that true inflammation did here exist within the cranium, but which was not disposed to proceed to any extent, nor in its ordinary course; hence is sanctioned the designation of "*spurious*" hydrocephalus, which we have assigned to the above collection of symptoms. Whether the cessation of purulent discharge at a distance were the exciting cause of the cerebral symptoms, or whether the occurrence of these had influence over the secretion of pus, supposing that they were being developed within the head before they were manifested externally, would supply matter for much consideration, and probably discrepancy in arriving at a conclusion, if we were to attempt to decide. But, for our own parts, we may perhaps be allowed to state, that the brain affection appeared to have been coming on anteriorly to the arrest of secretion in the ulcers, and that, consequently, as well as for other reasons, which may, probably, admit of different interpretation, and it were, therefore, unnecessary to specify, we do regard this latter circumstance but as secondary.

### COMPOUND FRACTURE OF OS FEMORIS.

William Harding, æt. 67, employed in looking after horses, was conveyed to this hospital, June 7th, 1831, about ten o'clock, a.m. having received a violent kick from a horse upon the lower part of his thigh, he being at the distance of a yard from the animal, so that the blow was delivered with the fullest force. Upon examination of the right thigh, an irregular wound was discovered at its outer part, between two and three inches above the inferior surface of the external condyle, and of the same extent in oblique length, from above downwards and inwards. Along with this outward breach of continuity, a fracture of the femur (having the same very great obliquity) was found, which reached downwards even to the upper border of the inner condyle, and the superior broken end of which protruded through the wound almost two inches. This latter was very rough, and several comminuted pieces were detached from it, and lying amongst the soft parts. The patient seemed, at this time, insensible of any shock to the nervous system, and complained only of local suffering. Mr. Travers, the surgeon of the week, was sent for, and arrived a little after 12 o'clock. We were, unfortunately, not present at the moment, and cannot give an account, from our own observation, of the different steps of the proceedings which he instituted. He removed a considerable portion of the projecting bone with the saw, previously to bringing the fractured surfaces into apposition, which it was not possible to accomplish without this measure, and then closed the external wound with strips of adhesive plaister. Some difficulty was experienced in elevating the inferior portion of bone to a level with the superior, in consequence of its being drawn backwards by the heads of the gastrocnemius externus; by flexing the knee, however, at a moderate angle, and raising the head of the tibia, by placing a pad between it and the inclined plane (formed of two flat pieces of board, joined together at an acute angle, similar to that used by Mr. Tyrrell, but wanting the pegs which produce separate partitions for the two lower extremities), on which the limb was placed, the two extremities were approximated. Some splints were applid with moderate firmness on the thigh in the usual manner, secured by tapes; and the foot was fastened to the end of the bed.

At two o'clock, when every thing was arranged, the man appeared tranquil, little disposed to irritability, was free from pain, and had a natural pulse of 70.

8, p.m. He has been quiet and easy, but incoherency in his answers is in a slight degree perceptible, his manner being sharp and snappish—makes no complaint of pain in the head, nor in leg, but has had no sleep—pulse nearly 90, soft and regular.

*Tinct. opii*, ♏xxx. *Mist. camph.* ℥jss.

June 8th, 1 o'clock, p.m. Mr. Travers visited him at this hour, and learned that, during the night, some little sleep had been obtained; but during the greater part of the morning he had been restless, throwing off the bed-clothes, and not allowing more than a single sheet to cover him, notwithstanding that a strong current of air was directed immediately upon him, in consequence of the windows and the door of the ward being open. There was a remarkable sunken look upon his face, some contortion of the features, he frequently drawing up his lips together in a peculiar way; a gentle groan was occasionally uttered—the tongue and mouth were extremely dry, and the former coated somewhat of a yellow colour—great thirst. A dose (℥j.) of castor oil was given last night, which, failing to act, a second was administered this morning, and a tolerably-copious and healthy stool was passed an hour ago. Was incoherent to a little extent, but not amounting to delirium—no screaming nor loud talking—surface of the lower extremities cold, that of arms and chest felt heated—had had some sensation of chilliness, and complained of head-ache and pain in the seat of fracture, but did not acknowledge any in the back. At first he denied having had head-ache, evidently answering without knowing what he said, and at this time the question was put to him about pain in the course of the spine. His pulse was surprisingly unaffected, beating 100 only, rather strongly and regularly, and allowing of ready compression. Mr. T. had more clothing placed upon him, and shortly afterwards he seemed disposed to sleep, but his eyes were closed merely for a few minutes, and the restless motions were then resumed. It was now determined to try the effect of taking blood from the arm, the condition of the pulse being carefully watched whilst it was flowing. Before five ounces were drawn, the pulse was accelerated to 112; and when the quantity arrived at eight, there was a manifest diminution of power. The bleeding was, therefore, stopped, and the patient confessed he felt relieved in his head, and "less faint." The apparatus on the thigh was not interfered with, the position being unchanged.

Mr. T. observed that it was unusual to witness so much irritability of system as in the present case, succeed within the same short period, and with the trivial alteration of pulse, which could not be regarded as varying much from the healthy standard. He prescribed:

*Sodæ carbon.*, *Magn. sulph.* āā ʒj. *Vin. ant. tart*, ♏xv. *Aquæ fontanæ*, ℥j. *Syrup.* ʒj. *M. 6tis horis, c. Succ. limonis*, ℥ss. *sumend.*

Within a couple of hours after the venesection, we beheld him tossing about his arms and the bed-clothes—in fact, there was equal restlessness to the former. He appeared, however, to express himself in less pain, though it was difficult to satisfy ourselves on this point. The mouth was moistened with toast-water, &c.

9th. Last night he was quieter, and has continued so from six o'clock in the evening till now (three, p.m.) His aspect is a little depressed, but the degree of anxiety is not increased, and he is certainly more rational and collected: we cannot, in fine, perceive any tendency to incoherency. The mouth is excessively parched, the tongue more dry, glazed, and striated, covered with a slight quantity of brown fur, and he is continually craving for drink, though not impatient for it. Skin is extremely hot and dry. Pulse, however, not much excited, about 116, moderately full and soft, and possessing some power. He says, on being minutely questioned, that he experiences no pain of head nor back, and the limb is perfectly easy. His bowels acted in a healthy manner about half an hour since. We find that Mr. Travers visited the patient at one o'clock, and examined the wound, after removing the splints and plaster. The sides of it were not clean, and, indeed, there was a disposition in the cellular tissue to slough. The restless movements of the extremities have quite ceased, together with the contortions of the mouth. A pledget of lint was simply introduced into the wound, from which were expressed some pus and sanious matter and which was treated after the manner of a sinus; and the splints were lightly re-applied upon the thigh. The mixture was ordered to be continued, with thirty minims of antimonial wine in lieu of fifteen; and, as sleep had been obtained without opium last night, this was not, of course, to be repeated.

10th, 11, a.m. His night has been quiet, and there has existed a constant inclination to sleep, or rather a heavy state, more correctly to be denominated somnolency. Last evening his pulse was at 90, and of the same character as in the fore part of the day, and his skin cool. There was, in some measure, less dryness of mouth and tongue, but the skin was hot and arid. At present he is sensible of approaching debility, which is indicated in the countenance as well as by the pulse; this is beating 100 in the minute, but not with its former full and free action—the tongue is now moist, covered with a whitish-brown coat, and the skin is temperate and perspirable. He does not acknowledge any pain. Has taken nothing but his medicine and diluents, and seems to be far more confused than yesterday.

At one o'clock he was seen by Mr. Travers, and he then plainly more needed support — the tossing of the bed-clothes was increased, as also restlessness. Mr. T. directed strong beef-tea to be given liberally, and the following draught every six hours:—

℞. *Ammon. carbon.*, grs. v. *Mist. camph.* ℥j. *Tinct. hyosciami*, ♏xx.

*Tinct. opii,* ♏x. *Aq. cinnam.* ℨiij. M. *Empl. lyttæ nuchæ.*

4 *o'clock, p. m.* More disturbed and restless—perpetually throwing off the bed-clothes—the eyes, both yesterday and this morning, have exhibited a glistening and watery look, which has recently become more observable — tongue has changed to a dry and brown condition since 10 o'clock — pulse quicker, and more feeble—skin colder, and dry, below natural temperature. He is tranquil, and apparently devoid of pain.

Failure of power was more and more observable in the symptoms of the evening, which continued otherwise the same. Low muttering delirium came on about eleven o'clock, which was soon followed by embarrassment of breathing. It was with great trouble that his medicines were got down him up to this period, and they did not appear to revive him even for a time. From this depressed condition it was impossible to rouse the vital energies by stimulants, which were therefore not exhibited, and at half-past nine, a. m., of the 11th, he died.

It appears that the poor fellow had not been of intemperate habits, though accustomed to a large consumption of porter.

June 13th. Examination of the body at two o'clock, p. m.

Right leg not œdematous, but the superficial veins below the fracture were very conspicuous even to the toes. The wound was excessively offensive, and somewhat sloughy. Upon enlarging it, the fractured ends of bone were fully exposed, and the following was the state of parts offered to view. The upper end, which was rendered smooth by the saw, was thrown below and to the outer side of the lower portion, closely pressing against the synovial membrane of the knee-joint, which was not however perforated. The inferior extremity was situated behind it. Several loose spicula were found between the two; one large and flat piece was particularly deserving of notice, two inches in length, and detached from the internal and posterior surface of the upper part of the femur. A second fragment, similar in size and figure, was likewise separated from the lower end.

*In the Head,* the vessels of the dura mater and pia mater were distended far beyond their natural degree, but in the substance of the brain itself no increased vascularity was visible. The thoracic and abdominal viscera shewed no traces of organic disease.

### Dislocation of the Clavicle.

Thomas Merritt, æt. 28, a remarkably muscular and well made man, was admitted into hospital on the evening of December 27th, 1830, with a dislocation of the external end of the clavicle, occasioned in a drunken scuffle, in which he fell with great force backwards upon his right shoulder. The extremity of the bone was readily felt displaced on passing the finger along the spine of the scapula towards the acromion. The shoulder was rather inclined inwards, that is to say, more closely approximated to the sternum than the opposite. Considerable power of motion over the extremity was necessarily lost, but the pain derived from the displacement was very trifling. No difficulty was experienced in replacing the bone, the knee of the dresser being fixed between the scapulæ behind, whilst the shoulders were forcibly drawn backwards and from each other. On the next day the patient was free from any febrile or inflammatory symptom, and the apparatus for fractured clavicle invented by Mr. Amesbury, was put on, with pads under the head of each humerus.

Dec. 31st. The scapular end of the clavicle has become again dislocated, but it was returned into its natural situation with facility by the same means as before.

1831. Jan. 13th. Presented well.

### Dislocation of Humerus into Axilla. Fits succeeding to the Reduction, during which the Clavicle became Dislocated.

Joseph Wright, æt. 24, was brought to the hospital about nine o'clock at night, on Dec. 28, 1830, having the right humerus dislocated downwards into the axilla, an injury for which he had been twice admitted here before. The head of the bone was reduced easily by making extension of the arm downwards with the heel in the axilla. As soon as this was effected, however, very violent fits of epilepsy came on, which required the strength of three men to keep him on the ground. With such a force even he could not be firmly fixed in the same situation, and during his struggles the scapular extremity of the clavicle on that side was dislocated upwards and backwards, most probably from the concussion against the ground. When the convulsions had ceased (in about half an hour), he was removed to a bed in George's ward, and passed a tranquil night. The displaced clavicle was returned with facility, in the same mode as in the foregoing case, on the 29th. He then had some pain of head, and was rather feverish, both from the effects of liquor (he having been intoxicated just before admission) and the injuries he had sustained. This febrile state was speedily relieved by low diet and purgatives, and on the 31st we found him nearly free from complaint, having no pain and only very slight tenderness about the shoulder.

Feb. 6th, 1831. The fits recurred with great violence on the evening of to-day, after the patient had been in bed about two hours, but no mischief to the shoulder ensued.

17th. Presented well.

March 1st. He again dislocated the same shoulder a few nights ago from a trivial cause, and the bone was replaced by some common person near him at the moment, the patient simply desiring him to extend the arm forcibly from the hand.

### LARGE OSTEO-SARCOMATOUS TUMOUR ON THE ARM.

Joseph Hampton, about thirty years of age, of dark complexion, and short in stature, presented himself to Mr. Green in the latter end of July, 1831, having an immense tumour, equal in size to a gallon cask, and somewhat of the same shape, situated on the left upper arm. The magnitude of the enlargement was of course very manifest to the eye at a considerable distance, and he was obliged to support the limb in a sling. The attachments of this body above were almost entirely confined to the humerus, although the shoulder-joint was completely overlapped by it. It was evidently a growth from the periosteum of that bone, and considered by Mr. Green to be periosteal osteo-sarcoma. Some strong fibrous (probably tendinous-like) bands of adhesion existed between its upper part and the acromion and clavicle, but these bones had no share in giving origin to the disease. Below, it extended nearly to the elbow-joint, within an inch of it, there terminating by a round, and its less extremity. The whole mass was of an irregularly oval figure, disproportionately great in the middle, and larger above than at its lower part. Its principal bulk occupied the anterior and outer sides of the limb, but the opposite surfaces of the os humeri were likewise involved. The preternatural structure was remarkably hard and unyielding—in a few parts only was any impression to be made by the finger—very ponderous, and no doubt solid throughout. He stated that there was constant and pretty severe pain in the whole member, from the incessant dragging consequent upon the weight of the tumour; and the inferior angle of the scapula was drawn outwards from the median line fully an inch, from the same cause. Ease was always afforded when it was supported, but he did not then enjoy perfect immunity from pain. The motions of the shoulder-joint were of necessity exceedingly limited, and those of the elbow also in an important degree. There was no numbedness of the hand, nor œdema. He was able at the table to cut his meat with the hand of the same side, and the fingers could be freely flexed and extended. His health had not suffered, and he seemed in good spirits; but he had not been able to undertake any kind of work, further than that required in his own house and garden. The disease had originated eight years before, and its increase had been rapid, especially during the nine preceding months.

Mr. Green recommended him to get rid of it by parting with his limb. It would have been necessary to remove the whole scapula along with the humerus, otherwise no flap could have been made of integument not implicated. The patient, however, did not appear at all disposed to take Mr. G.'s advice; and when we called upon him a month afterwards, at his home in Walworth, he was still more adverse to any proposal of the kind.

About six months previously he was seen by Mr. Travers, who declared his opinion as unfavourable to the removal of the disease; but what he judged the affection to be, we cannot positively assert.

In a case of osteo-sarcoma, very similar, but not so bulky, Sir Astley Cooper amputated the whole arm and scapula, with success. The disease was situated on the upper arm, and no other bone except the os humeri was concerned. A cast of the limb after amputation is preserved in the collection of this hospital.

### Exostoses of Unusual Magnitude originating in Infancy.

The father of the above man (Hampton,) about 60 years old, exhibited some rare specimens of exostosis on the upper extremities. He had one very remarkable enlargement upon the neck of the right humerus, round, and so much like the configuration of the head of the bone, that there was at first supposed to exist an unreduced dislocation of this latter into the axilla. The motions of the joint were, however, nearly as free as natural, and upon further scrutiny, after seeing this mobility, Mr. Green ascertained the real state of the case. Upon the olecranon of the same arm, another considerable one existed, and also a third of large size near the carpal extremity of the left ulna. There were, moreover, two or three of smaller dimensions on other parts of each fore-arm. The largest were equal in size to hens' eggs. They had all originated in his childhood, or rather infancy, according to the man's own statement, and gave rise to no suffering, nor inconvenience. No increase had taken place in them within his recollection. Beta.

### Gangrene of the Feet from Frost Bite, subsequently requiring Amputation of both Legs.

John Wiggins, æt. 39, was admitted into St. Thomas's Hospital, Dec. 15th, 1829. He has followed various occupations during his life time, but has generally lived poorly: he states that about a month ago (having no home) he had lain in the open air during four successive nights, the weather at the time being intensely cold. On the morning after the fourth night, he found himself unable to stand, in consequence of the loss of sensation in his feet. So complete was the loss of sensation that he was totally unconscious of touching the ground. He was in this state conveyed to Cold Bath Fields Prison, as a vagrant, where his diet for some days was gruel and soup, on alternate days. After arriving at the prison, he describes the sensation of his feet as having changed, and instead of the numbness which had before existed, he had a sensation of pricking or stinging heat. This, according to his account, was soon followed by more manifest signs of inflammation, as redness, throbbing, and swelling of the parts. He does not remember what was done for him, but these symptoms, accompanied with great pain, continued for *eight days*. After this period, the feet were discovered during the night to have become sphacelated; the pain then remitted, the feet became of a black colour, and in different parts slight vesications arose; in a day or two after, a line of separation between the dead and living parts became evident, and with slight variations in his health he passed the remaining time in the prison infirmary, until his admission into St. Thomas's Hospital. His state was then as follows:—the entire sole of each foot, together with all the phalanges of the toes, was in a state of gangrene, being black, dry, and shrivelled; a well-marked line of separation existed between the living and dead parts, and numerous well-formed granulations had arisen on the former; there was profuse fetid discharge, and he complained of great pain. Pulse 90 and feeble, tongue moist, slightly furred, appetite good, bowels confined, complains of want of sleep. He was ordered :—

*Pulv. rhei,* grs. xv. *statim. Mist. camph.* ℥iss. *Carb. ammon.* grs. v. *Conf. arom.* ℈j. *Tinct. opii,* ♏x. 6*tis. horis. To have sago, with* ℥ij. *of Port wine daily, extra meat, and half a pint of porter. Chloride of lime lotion and a poultice to be applied to the feet.*

Dec. 16th. He is suffering all the evils of repletion, the nurse having allowed him to take all the above prescribed nourishment at one time. He has head-ache, a furred tongue, the bowels have not been opened, and his pulse is 100, and full. His meat was ordered to be suspended for the present, and to have ℥iss. of cathartic mixture directly, to be repeated in three hours if necessary.

17th. The bowels were not relieved until he had taken two doses of the medicine. The symptoms of excitement have now in a great measure abated. The pulse is soft, though still frequent. The feet are very painful, but the discharge is less offensive.

19th. He still complains of want of sleep, the pulse is improved in power, and his appetite is good. *The mixture to be discontinued, and ordered to take opii,* gr. j. *ter die.*

22d. He complained much of the pain in his feet. Granulations have arisen in considerable quantity, and the process of separation is proceeding rapidly, the dead portions being apparently only held by the metatarsal bones.

26th. With the exception of pain he is in every respect improved; and as it is pretty clear that the sphacelated portion is only retained by a slight connexion of bone, sufficient, however, to render the separation by the unaided natural powers unnecessarily tedious, it was determined *to remove* the right foot. In doing this it was found that the line of separation had passed through the metatarso-phalangeal articulation of the great and little toes; but it was necessary to divide the three intervening metatarsal bones with the saw, near their phalangeal extremities. With the exception of one or two tendons, which were divided by the knife, the whole sole of the foot was completely separated by absorption — passing in its course directly through the tuberosity of the os calcis — slight hæmorrhage followed the removal, but was immediately restrained by a dossil of lint. A poultice was then applied. He was ordered to take—

*Mist. camph.* ℥iss. *Ammon. carbon.* grs. viij. *Conf. arom.* ʒss. 6*tis. horis. Opii,* gr. j. *sexta quaque horâ.*

27th. Has passed a good night, and feels less pain—*to continue his medicines and diet.*

28th. The left foot was this day removed in the same manner as the former. It was found necessary to saw through the three inner metatarsal bones, and the tuberosity of the os calcis.

31st. Seems to improve rapidly, both the stumps are covered with healthy granulations, excepting the surface of the left os calcis. *Medicines as before, and calamine cerate to the sores.*

Jan. 4th, 1830. For the last day or two his health has been deranged, and without any apparent cause—his bowels are confined — and on examination of the extremity, a gangrenous spot, the size of a half-crown, was discovered on that of the left side.

*Pulv. rhei, c. hydr.* grs. xij. *statim.*

5th. The bowels were freely acted on and the slough, which proved to be merely superficial, has separated.

26th. He has continued to improve up to the present date. The ulcers are cicatrizing slowly, from their edges, and the granulations of the left one are healthy on the right side; they are, however, rather flabby. He was ordered—

*Quinine sulph.* grs. ij. *Extr. gentian,* grs. iij. *ter in die. Opii,* gr. j. *ter die. A pint of porter daily.*

Feb. 8th. The cicatrization of the left ulcer has proceeded rapidly, being almost healed, but on the right stump the process is more tardy,—the portion of os calcis here exposed seems about to exfoliate.

20th. Little alteration has taken place. The increased portion of bone still remains, accompanied with considerable fetid discharge.

28th. The portion of bone was removed this day.

April 3rd. During the period between the last report and the present date, there has been nothing to notice. The left ulcer has healed, the right sore has occasionally varied in appearance, as his health has been better or worse.

October 1st. An extremely indolent ulcer, to heal which several applications have been tried in vain, exists upon the plantar surface of the remaining portion of the right foot. As this is not likely to cicatrize, and moreover as this stump would be of no service to the patient hereafter, so as to enable him to earn his living by active means, together with the opposite, a wooden leg would be a much more useful substitute, and amputation of the right leg therefore will be proposed. Some superficial ulceration, partaking also of much the same character as on the right, has been established in the extreme part of the opposite, but is now presenting a surface disposed to contract, and the granulations are tolerably distinct.

Dec. 10th. No favourable change having transpired in the condition of the sore upon the right side, this leg was to-day amputated at the usual distance below knee, by the circular incision. The spine of the tibia was sawed off obliquely downwards from the knee, and then divided in a directly transverse direction together with the fibula, whereby, a piece of bone was removed from the front of the tibia, and the sharp edge here usually projecting, obviated. Three ligatures were applied on the principal arteries of the limb; namely, the anterior and posterior tibial, and the interosseal. The flaps of integument were brought together in the ordinary mode from side to side.

*Vespere.* Slight twitchings of the stump have been experienced, but with this exception, he has been perfectly void of pain.

11th. Has passed a somewhat disturbed night, but has no febrile excitement. The bowels not being opened, a dose of oleum ricini was administered.

12th. Four stools have been obtained, and there is no bad symptom to be complained of, his rest last night having been good.

13th. There has been a very free discharge from the stump, red, and watery.

14th. Patient easy. No alvine evacuation since 12th. The straps were removed from the lower part of the stump; no matter was confined, and adhesion, as favourable as could be desired, has taken place. Two strips were replaced. *Ol. ricini*, ℥j.

15th. Frequent pains of a spasmodic kind have arisen across the umbilical region, attributed to some improper article of food he has taken, unknown to the sister or nurse. Dressings were taken off above, and the edges of the stump adhere, with very slight gaping. No discharge of pus is at all visible.

16th. Spasms have continued throughout the night, but are now abated in a great degree.

21st. Stump going on well. The edges have not been quite clean, and the lot. chloride calcis, on lint, has been applied to them.

26th. Stump is looking remarkably well, and granulations of a florid colour have appeared.

1831, Jan. 2d. No ligatures have yet separated, but the wound is nearly healed.

4th. One ligature came away.

10th. A second ligature separated. Stump quite healed.

13th. The last ligature was removed.

March 30th. The ulcer upon the left foot, which at the time of amputation was not larger than a sixpence, circular, and surrounded by hardened margin, has recently spread over the whole plantar aspect of the part, without any obvious cause, either locally, or in the state of constitution. It is to be apprehended this limb will not be preserved. No deviation from the healthy standard has at any period been observable in the patient's general condition, since particular mention was made on this point.

June 1st. The ulceration on the left sole is at present in a healing condition, but not reduced to the small space it has once occupied. Various applications have been used, according to circumstances, and now it is dressed with dry lint and adhesive plaister. Mr. Green has conferred with him about parting with this leg also, as the stump, if ever cicatrized, will be useless, and ever liable to ulcerate afresh. The poor fellow seems unwilling to submit, being in hopes of obtaining admission into Chelsea Hospital as an in-pensioner, in which capacity no bodily labour would be required of him, and he could pass his time comfortably with only one wooden leg. But if he fail in obtaining this berth, he sees the necessity of then losing his limb, upon which, in its present and probable future condition, he would not be able to work, so as to derive any emolument in addition to his scanty pension.

October 20th. The poor fellow has again been admitted into the hospital, with the full determination to lose his remaining limb. The ulceration of the foot has spread, so as to occupy the entire of the inferior surface, and without evincing any disposition to take on a healing process. Amputation of the leg, below knee, was accordingly performed on the 7th December. Nothing untoward happened; the stump healed well, and he went out Feb. 14th, 1832, being in a much more comfortable condition with his two wooden legs, than whilst retaining his limbs with the indolent and intractable ulcers.

DELTA.

---

### VIII.

**COUNTERACTION, VIEWED AS A MEANS OF CURE, WITH REMARKS ON THE USES OF THE ISSUE. By JOHN EPPS, M.D. Octavo, pp. 69. 1832.**

THE acute observer, who has published this little pamphlet, hopes that he has been able to throw some new light on the subject of counter-irritation, though most of the facts which he has adduced may be well known. The proceedings occasioned by the empiricism of Long and his associates seem to have called our author's attention more especially to the subject, and he has prosecuted it with assiduity. Dr. Epps prefers the term counter-action to that of counter-irritation, because it may be questioned whether irritation is always the result or the concomitant of counter-action, and, allowing that it did always result, the term, he observes, expresses only *one* effect, and not the whole of the effects. He considers the subject under three heads—spontaneous, accidental, and artificial counteraction, to which are added, remarks on individual counter-agents, peculiarity of constitutions, &c.

Under the head of spontaneous counteraction, Dr. Epps adduces the cases of eruptions and discharges behind the ears of children, by which the premonitory symptoms of hydrocephalus are removed—those of dentition relieved by cutaneous eruptions on the body—and those indicating approaching apoplexy by epistaxis, the breaking out of ulcers on the legs, &c. Our author pursues his illustrations through the regions of the head, chest, and abdomen, adducing curious and instructive examples of the sanative efforts of the constitution when threatened with disease.

The second chapter is on accidental counteraction, beneficially induced.

"Captain Ians, of the royal navy, was tapped, and nineteen pints of water were drawn off. The abdomen began again to fill, and several pints of fluid, so far as could be judged, had re-accumulated. The servant one day, in removing the kettle from the fire, accidentally spilled some boiling water on the captain's leg. Inflammation came on. An ulcer was formed. This spread. As it spread—in other words, *as the disease in the leg progressed—the accumulation of water in the abdomen diminished,* and the officer was at length perfectly cured of his dropsical affection. This case occurred in Dr. Hutchinson's and Mr. Houston's practice.

Cases similar to the following are familiar to many practitioners.

A. B., aged nineteen, has been subject to *bad eyes* and a *discharge* from the *ears* ever since she was fourteen. At twenty, A. B. marries; and, becoming pregnant, the eyes and the ears become well. The children are either scrofulous or scorbutic, according as the discharge in the mother partook of the one or other character. The pregnancy here acts as a counteragent; in other words, when impregnation takes place, another action—that of formation—is set up in the system. This new action overcomes that on which the discharge from the eyes and the ears was dependent, consequently the discharge ceases.

A child, who was troubled with severe cough of several months' continuance; whose appetite was sometimes good, sometimes bad; countenance first flushed, then pale; became, either from cold, or from the friction of the tightly-tied bonnet-strings, afflicted with a swelling, and finally a suppuration, of the glands of the neck. All the previous symptoms passed away, and continued absent as long as the discharge continued."

The counteraction by means of the external application of heat, chiefly by baths, &c. is illustrated by many instances of its powerful effects, but these need not be repeated. Next, Dr. E. proceeds to counter-irritants, properly so called, in the form of rubefacients, epispastics, escharotics, and purifacients, as setons, issues, &c. The following passage respecting friction is curious.

"It is a lamentable fact, that practitioners are not generally aware of the benefits arising from friction over the bowels in cases of *constipation*. My plan is to order the bowels to be rubbed with *soap lather* every night on going to bed. This rubbing promotes the peristaltic action on which the evacuation of the fæcal matter in a great measure depends. I have seen such benefits arise from the plan, that I have no doubt of its efficacy. Hence Sydenham's recommendation of horse-exercise, the beneficial agency of which is, in a great measure, connected with the gentle muscular action, bringing about a state of the circulating fluid very similar to that brought about by means of friction."

The work, if widely circulated among the non-professional public, would be very useful in checking quackery, by explaining the rationale of external excitation. The author has keenly exposed the charlatanneries of St. John Long; but the public cannot exist without delusion. The liberty of throwing away their money and their health on ignorant and unprincipled quacks, is as dear to John Bull as the trial by jury, or the Magna Charta.

---

### IX.

### On the Evolution of the Chick, &c.*

While the ovum is yet in the ovarium of the hen, and consists only of the vitellus, or yolk, the rudiment of the embryo may be discovered as a yellow spot, of a lighter colour than the rest of the yolk; if we examine this very carefully, it is found attached to a vesicle, situated under the vitelline membrane, and to which the name of cicatricula has been given. The first six figures in Plate VIII. illustrate this appearance. As the ovum passes along the oviduct, it receives the addition of the albumen, and is subjected to a rotatory movement, arising from the play of the muscular walls of the oviduct, by which it becomes more tapering at its extremities, and in time gives rise to the formation of the two chalazæ, by means of which the ovum is suspended and steadied.

Figure 8 very beautifully displays a vertical section of a mature egg, before incubation. Lining the shell, we observe its investing membrane, which consists of two layers, closely applied to each other every where, except at the base or larger end of the shell, where they are separated, and thus leave a cavity, which is filled with air,

* From Carus' Work, "Tabulæ Anatom. Comp. illustrantes."

and is termed "folliculus aëris." Within this membrane is the albumen, or white, or glaire, and this encloses the yolk, surrounded with the yolk-bag or membrana vitelli. Between the albumen and vitellus, are seen the two chalazæ, attached by their one end to the vitellian membrane, while the other floats free in the albumen. Upon the lateral surface of the membrane, we notice the "cicatricula," or what is vulgarly called the "tread of the cock," which, by being situated on the smaller portion of the sphere of the yolk, is always uppermost, in whatever position the egg happens to be placed.

During incubation, the folliculus aëris becomes gradually larger; the albumen is thinner and more turbid, and is partly consumed by the chick for nourishment, and partly escapes through the shell by evaporation; the cicatricula, by enlarging, forms the amnios, which contains the colliquamentum, or fluid in which the embryo is at first immersed: the umbilical vessels are expanded on the amnios, and on a membrane lining the shell, and which is called the chorion. On the second day of incubation, the embryo is first observed as a gelatinous filament. On the 3d day, the punctum saliens, or heart, is recognized by its motion; and the spine with the rudiments of the brain and eyes, are to be seen. On the 4th, the abdominal viscera and the rudiments of the extremities appear. On the 5th are seen the respiratory organs. On the 6th there is voluntary motion. On the 9th, ossification commences; and, on the 21st, the chick bursts the shell and escapes. In the year 1826, Professor Hüschke, of Dresden, made the singular discovery that, in the early stages of the development of the chick, there are to be seen three fissures, or slits, on each side of the neck, and that these fissures correspond with three bronchial arches, or true gills, each of which is supplied with a bronchial artery, which arises from the aorta; these appearances may be observed from the 4th to the 7th or 8th day of incubation, at which time the bronchial arches have disappeared, and are superseded by lungs, and the pulmonary vessels take the place of the bronchial ones. This metamorphosis of a bronchial into a pulmonary apparatus, is one of the most singular and unlooked-for phenomena of the animal organization, and is, according to Carus, to be regarded as a beautiful exemplification of the law of development that all the animals of a higher grade attain, step by step, and by passing through certain preliminary conditions of existence, to their complete evolution. According to some German writers, among whom is our author, the same changes may be traced in all the mammalia, as well as in all birds, during the very early stages of fœtal life.

The 9th and last plate gives some very good figures of the process of generation in some of the mammalia:—In them, the mode of evolution is, or at least appears to be, very different from what we have observed in the other classes of vertebral animals, since we no longer find that the young being is furnished with a yolk, or any similar supply of nourishment; Professor Carus, however, considers that the vesicula umbilicalis ought to be viewed as a part analogous to the vitellus, and as connected, somehow or other, with the growth of the embryo while in utero; and we know that, after birth, the food is drawn from the mamma of the mother, in the secretion of milk, which is not unlike to a thin or dilated vitellus. Respiration, we have reason to believe, is effected in the very young embryo by bronchiæ, afterwards by the vascular web of the allantois; and, in a later stage, by the vessels of the chorion, which, when conglomerated into one or several masses, receive the designation of placenta or cotyledons.

Figures 1 and 2 are a beautiful illustration of the appearances of an eight-week ovum of the sheep; the amnion, chorion, allantois, vesicula umbilicalis, arachus, &c. are very clearly seen, and also the anastomoses by arches (so as to look not unlike to bronchiæ), of the bloodvessels of a cotyledon.

Figures 3 and 4 exhibit the embryo of a bat; and the 4 following figures represent the young of a marsupial animal (Didelphis Virginiana).

In figure 10, the ovum of a mouse, we see very distinctly that the umbilical cord sends off, in addition to its vein and two arteries, the omphalo-meseraic bloodvessels, which were at first dispersed on the vesicula umbilicalis, or part corresponding (as mentioned above) to the vitellus of oviparous animals, but have been transferred to the inner surface of the chorion, by some denominated, very unnecessarily, the tunica erythroidea: in a like manner, the proper umbilical vessels belonged, at the very early stages, to the allantois, but subsequently to the placenta.

In figure 14, we observe that, in the very young calf, a diverticulum is attached to the intestinal tube, very near to the umbilicus; Professor Carus thinks that this is the remains of the canal which had formerly communicated between the gut and the vesicula umbilicalis, and is analogous to the ductus vitello-intestinalis of birds, &c. by which the yolk is taken into the body of the chick.

---

### SPIRIT OF THE PERIODICALS.

### X.

### A Case of Gastro-Enteritis, simulating Cholera. By Dr. Broussais.

January, 1832. The patient had been seized suddenly with severe colic, painful vomiting, and profuse purging; aspect cadaverous, face shrunk, eyes red, and mind bewildered; pulse small, and scarcely perceptible; extremities icy-cold; tongue dry, red, and cold; sordes about the teeth; epigastric region tender, when compressed. Although the pulse was so faltering, and the skin so cold, Dr. B. ordered 15 leeches to the epigastrium, and as many to the anus. An opiate starch enema was given to quiet the bowels; lemonade to drink. The diarrhœa was stopped, but the vomiting continued for two or three days; the stupor increased, the eyes became more red, and the face more corpse-like. Ten leeches over the jugular vein of each side, and frictions with warm camphorated vinegar on the abdomen. The head was thus relieved, but in two days afterwards the stupor returned, and 20 leeches were now applied to the temples, blisters to the thighs, and the stimulating frictions to the belly.

Six days having now elapsed, without any marked improvement, Dr. B. ordered two more blisters to the thighs, and the embrocations to be continued; and although the pulse was not to be felt, he forbid all stimulants, and even broth. On the 10th day the patient appeared somewhat revived, and he asked with a weak voice for food. A little very thin broth was given. A blister was ordered between the shoulders; the frictions were assiduously kept up, and the only food permitted was lemonade and gum-water. A slight attack of bronchitis came on, and the pulse rose and heat of surface encreased; in three days the symptoms abated, and the pulse again fell; the same treatment was continued. On the 15th day after his admission, the symptoms were thus reported;—head is less confused, aspect not so cadaverous, but the coldness of skin and smallness of pulse little improved. Next day, however, the pulse rose, and the patient appeared altogether better. The abdomen was soft, compressible, and not tender; and the tongue was moist, and still rather red. Dr. B. was led to infer that now the spasmodic contractions, induced by the phlegmasia of the small intestines, had ceased, and that therefore the peristaltic movements, hitherto impeded, might permit the digestion of broth; it was accordingly given with excellent effects, and the patient progressively *advanced to recovery.*

*Reflections by Dr. B.* It appears that gastro-enteritic inflammations have the effect of greatly weakening the action of the heart, and of thus enfeebling the cutaneous circulation, while at the same time the blood is congested in the viscera. In such cases we do not approve of the employment of purgatives, or of any stimulants; and allow only emollient drink at intervals, employ frictions on the surface, and large warm poultices to the belly. If one part of the abdomen appears more congested than another, leeches are to be put on that part, and to be repeated according to circumstances. This plan of treatment must be undeviatingly persevered in, till the spasmodic contraction of the bowels ceases, and the peristaltic movements are re-established, the indications of which are the relaxation and pliancy of the abdominal muscles, which so faithfully correspond to the condition of the intestinal superficies; then, but not till then, are we to give nourishment. In short, our treatment is comprehended in three things; viz. local bleeding, external warmth and frictions of the surface, and lastly, patience.—*Anal. de la Médéc. Physiol.*

*Remark.* The above case, though not a rare one, merits a careful study, and illustrates well Broussais' opinions.—ED.

---

### XI.

ABSCESS IN THE SUBSTANCE OF THE HEART. Reported by Dr. CASIMIR BROUSSAIS.

A SOLDIER, aged 19, entered the hospital of Gros Caillon, with a variolous eruption upon him—he passed through the several stages, apparently favourably; towards the decline of the disease, diffused abscess formed on the left elbow; the discharge was scanty and of a greenish hue; the fore-arm and hand became prodigiously distended by infiltration, and cold to the touch. At the same time, sloughs formed on different parts of the body, and the patient's strength was quite exhausted;—he died on the 55th day from the attack.

On dissection the heart was found to be larger than usual; but the left side alone was hypertrophied and dilated. At the base of the left ventricle, behind the mitral valve, and in the fleshy substance of the walls, there was an abscess, of the size of a filbert, containing a whitish homogeneous pus; the cyst had no opening, either outwardly or inwardly. The rest of this ventricle was not diseased; but the ventricle towards its apex, and the auricle of the right side, presented two patches where the texture had degenerated into a purplish spongy substance, not unlike to ordinary eructile tissue. No disease was detected in any of the arteries, or veins, nor in the other viscera.

*Reflections.* The preceding case, proves, beyond all cavil, that an abscess may form in the fleshy parietes of the heart, without occasioning any marked distress, or very evident sign, and that its formation must result from a local circumscribed inflammation.

The early morbid anatomists frequently mention suppurations, ulcerations, and gangrene of the heart; but we have reason to doubt their correctness; as no instance of mortification of the heart, is now-a-days heard of. Senac is the first author, who accurately describes the degenerations of the heart's texture. He says that the seat of the abscess is always in the cellular tissue connecting the muscular fibres, which are found generally to be little altered by the suppuration, and that the pus is poured out between their bundles. The base of the heart, he states, is most frequently affected. Neither Morgagni, nor Lieutaud have reported any cases. Laennec describes one case, in which he found an abscess in the substance of the left ventricle, near its base, in the body of a child, who had been affected with pericarditis. From the accounts left to us by Senac, Corvisart, and Laennec, it appears that there are no discriminating, or pathognomic symptoms of this degeneration. In some cases, the patients have died suddenly, at the time when they seemed to be in good health; in others, palpitations, and a feeling of strangling had been observed. Of one thing we are assured, that an abscess of the heart may form, without any pain or suffering; a very satisfactory proof, that pain is not a necessary symptom of inflammation.

A very interesting and important feature in the present case, is, the occurrence of certain changes, probably antecedent to suppuration, in the textures of the right ventricle and auricle. Dr. Broussais describes these as small circumscribed inflammations, with a sanguineous turgescence, induration, and incorporation of a black-coloured blood with the muscular substance, which appearance was compared by some to an interstitial apoplexy, and by others, to the structure of the corpora cavernosa penis.—*Ibid.*

---

XII.

ERYSIPELAS, TREATED BY MERCURIAL INUNCTION.

DR. BROUSSAIS has repeatedly employed this method, with very satisfactory results. The parts affected are to be gently rubbed with a mixture of equal parts of "onguent Napolitain" and of lard. The cases suited to this practice, are such as supervene to diseases by which the body has been much weakened, and in which the inflammatory action is feeble.—*Ibid.*

---

XIII.

DR. BROUSSAIS' OPINION ON THE USE OF LARGE DOSES OF TARTAR-EMETIC IN PNEUMONIA.

THE employment of this agent in acute inflammation of the lungs is most efficacious, when the patients have been weakened beforehand sufficiently by bleedings. We are more and more satisfied that it acts, not as a specific in counteracting inflammatory excitement, but altogether as a revulsive, or, in other words, that the relief to the lungs is always in proportion to the rapidity and to the quantity of discharges produced, whether by vomiting, purging, sweating, or flow of urine. It is quite a mistaken notion, to suppose that the amendment of a patient begins only when the antimonial ceases to have some sensible effect, or, as it is expressed, when a "tolerance" of the drug is induced. When it checks an inflammation, without causing vomiting or purging, we shall find that profuse sweating or diuresis has generally taken place. But should no evacuation at all occur, the antimonial becomes an irritant to the stomach, and gastritis ensues. Dr. B. has seen this repeatedly take place, and, therefore, dissuades its employment, whenever there are any symptoms of tenderness of the stomach, whether this be primary or consecutive. He narrates the following case. A soldier entered the "Hôpital du Val de Grace," having all the symptoms of an acute pneumonia of both lungs, with a hypertrophy of the heart. He was bled largely and repeatedly, but did not experience relief; indeed the symptoms were aggravated, and the antimonial solution was ordered to be taken every hour. It produced copious purging, but the disease advanced rapidly to a fatal termination.

On dissection, the greater part of the lungs was perfectly normal, congestion being visible only in one part. On an examination of the stomach, its mucous coat was found intensely inflamed, and the inflammation was traced to have extended along the whole extent of the duodenum, as far as one-half of the track of the ileum. The inner surface was much softened, and in patches converted into the appearance of a boiled pulp. In short, this patient, says Dr. Broussais, died, not from a pneumonia but from an intense gastro-enteritis. —*Ibid.*

*Obs. by Ed.* The above report appears to us to be exceedingly imperfect and ill drawn up, and does not do much credit to the enlightened physician, as no accurate diagnosis of the transference of the malady seems to have been made before death.

---

### XIV.

### CASE OF DIABETES MELLITUS.

A MAN, aged 24, had for 5 months been affected with an unquenchable thirst, and a most voracious appetite, and yet had lost considerably both flesh and strength. Skin dry—pulse 60 to 65, very small — profuse diuresis — great wakefulness—no pain any where—respiration and digestion good—bowels regular. The colour of the urine was like that of clarified whey; it had no urinous smell, and was sweet to the taste. The patient had resided long in a damp climate, and had suffered repeatedly from several enfeebling diseases, as fevers, gonorrhœa, &c. He himself dated the commencement of his malady to a debauch in drinking beer, having, in the space of 12 hours, swallowed no less than 16 or 17 pints of it. In the present, as in many other recorded cases of diabetes, there was a marked tendency to blindness from amaurosis, a coincidence, or rather a result or sequence, worthy of the at-attention of oculists. He was put on an animal diet, and allowed milk and gum-water as a drink; was cupped over the kidneys—had the whole body well rubbed with camphorated spirits, and took one grain of opium at night; occasionally had a vapour-bath, which was subsequently changed to a sulphurated one; wine was sometimes allowed him; two moxas over the kidneys; the quantity of animal food was gradually increased. Under this treatment for three weeks, the quantity of urine was reduced from 15 to 16 pints, down to 6 or 7 pints; and at this period he left the hospital.—*Ann. de la Med. Physiol.*

---

### XV.

### ENDERMIC THERAPEUTICS.

THE method followed in France is as follows:—Apply to the skin, which we wish to deprive of its epidermis, a portion of ammoniacal pommade, which is made of equal parts of lard and the strong liquor ammoniæ, and renew the application in five minutes; in five minutes more the blister will have risen, and we then remove the epidermis, and sprinkle the raw surface with the medicament. Half a grain of acetate of morphia, applied on a blistered surface near the origin of the sciatic nerve, has, in 24 hours, cured a severe neuralgia of the limb; and sulphate of quinine has, when similarly used, quickly put a stop to an ague, when the medicine could not be administered inwardly.—*Ibid.*

---

### XVI.

### MODE OF DRESSING A STUMP, AFTER AMPUTATION OF THE THIGH.

WHILE the assistant draws down, with both his hands, the skin and muscles over the face of the stump, let the surgeon pass a bandage round the pelvis, and roll it regularly and firmly around the limb from the hip to the wound; apply the two cut surfaces neatly together, and retain them there by cross pieces of bandage, secured by a turn or two of the roller: over these put a piece of simple dressing and a pad of soft lint, which are also kept in their place in the same manner, without the use of any adhesive plaster, which are condemned as highly irritating to the wound.—*Journ. Complement.*

We quite approve of the above method, and have repeatedly observed the pernicious effects of applying adhesive plaister to large wounds. It gives us pleasure at the same time, to find that union by the first intention is now admitted into French surgery.—ED.

---

### XVII.

### CASES OF PELLAGRA, OR "MANIA PELLAGRINA" OF ITALY, COLLECTED FROM THE GREAT HOSPITAL AT MILAN.

THIS malady has received various names, such as pellagra (from two Italian words, denoting fissures or chaps of the skin) —dermatagra, or periodical chronic erysipelas—mal de misère—insolation du printemps — scorbut Alpin —paralysie scorbutique—erythema endemicum, sive pellagrum (Alibert)—maladie symptomatique des lésions du tube digestif (Biett). It is uncertain when the disease was first recognized; mention is made, in the records of the Milan Hospital for the year 1578, of a disease designated "pellarella," but no characters or symptoms are enumerated. Numerous authors maintain that it is of comparatively recent origin, and date its first appearance about the beginning of the last century. Its causes are equally uncertain, being by some attributed to the action of the sun, by others to atmospheric influence, or to the use of unwholesome, saltless food—of unfermented and deteriorated bread; Marzari blames the Indian corn and "nolcus sorghum;" the impure and muddy water which is drunk has been accused of it. When, from the causes, we pass to the characters of the disease, equal discrepancy of description is found; it has been compared to hypochondriasis, impetigo, vitiligo, lepra, elephantiasis, scorbutus, ephelides, mountain scurvy and "mal de la Rosa," and mange. Alibert says it is a species of exanthema, arising, not from ordinary inflammation, but from a virus which is generated; Biett regards it as always symptomatic of disorders in the digestive organs; Dr. Strambio, of Milan, thinks that it originates in an irritation and slow inflammation of the neurilema of the spinal nerves and marrow, which, in course of time, occasions a chronic gastro-enteritis; and Drs. Liberali and Carraro describe two degrees of the disease—the first of which, produced by the use of unwholesome, indigestible food, is designated a slow gastro-enteritis, and the second, brought on by bodily sufferings and mental distress, is a slow gastro-meningitis. This melancholy disease is not confined to one sort of locality; Strambio has seen it rage among the smiling hills of Seprio, Brianza, and on the confines of Olonna; and has remarked that, while it proved very destructive in the arid parts of the Duchy, it has been less malignant in those which are better watered. He found it to be prevalent among the poor peasants, who live in the dry plains and hills, drink clear wholesome water, and never feed on fish, or any salted meat; and, on the contrary, to be much less common among the inhabitants of the wet and rice districts, who live a great deal on salt provisions, and who drink a very impure water. Similar observations have been made by Cerri, on the frequency of the disease in the dry grounds between Lago Maggiori and the Lake of Como, where the people live entirely on bread made of Indian corn, wheat, and rye, while the inhabitants of the mountains and wet country escape it. But, on the other hand, Dr. Griva has seen it in many of the damp provinces of Piedmont, and in the very dissimilar regions of Friuli, Treviso, the Venetian States, Padua, Parma, Piacenza, Florence, &c.

Three stages of the disease have been described:—In the first, the skin of the feet, hands, and of such parts as are exposed to the sun, becomes red, is very itchy, and in time the epidermis falls off in scales; in the second, the skin is wrinkled, hard, and full of chaps; the person is haunted with fear, is anxious, and cannot sleep; he falls into a state of hypochondriasis, imbecility, or of mania, is troubled with diarrhœa and extreme debility, and loss of all muscular powers; yet there is no fever. The third or last stage is marked by fever, colliquative purging, stupor, and death. Strambio states that pellagra is successively of an intermittent, remittent, and continued type; that the cutaneous affection has also a threefold character, being at first scaly, then vesicular, and lastly desiccative, or in which the skin peels off without any preceding heat or redness; that the delirium is sometimes of an acute form, and that, at other times, it is chronic, shewing itself under the signs of deafness, melancholy, extreme taciturnity, attempts at suicide, especially by drowning. The duration of the disease is generally for several years. The class of the population who are by far the most subject to it are the peasantry. It is not often observed to attack children under 12 years of age. Some authors deem it contagious and hereditary; others deny both of these positions. The pathology of pellagra is not very satisfactorily made out. The following appearances have been noted: a collection of yellowish serum between the membranes, and in the cavities of the brain—congestion of the vessels of the pia mater, plexus choroides, and of the cerebral substance, suppuration and hardening of the brain itself—inflammation of the spinal chord and of its membranes—accumulation of serum in the pleural cavities—inflammation and abscesses of the lungs—ulcers of the trachea—dropsy of the pericardium and of the abdomen—chronic peritonitis—ulceration of the stomach and intestines—hypertrophy—tubercles and scirrhus of the liver, &c.

The prognosis is always very unfavourable; by many, the disease is deemed incurable.

*Treatment.* Diaphoretics, warm baths, vegetable decoctions, as of sassafras, guiac, &c. aperients, antiscorbutics, mercury, antimony, and a host of other remedies have been tried, but hitherto with little avail; we need not dwell longer on this part of the subject; but rather detail a few illustrative examples.

*Case* 1. A woman, aged 34, was admitted into the Milan Hospital with symptoms of gastric irritation. On examination, the hands, feet, and bosom were found affected with an erysipelatous eruption; the redness was uniform in some parts, patchy in others, and having the appearance of what artists term being stippled; its hue varied from a light to a very dark shade of red. The rest of the surface was not affected. The patient had been ailing for three months; had lost her appetite, strength, and she had become very desponding: the abdomen was slightly tender on pressure—the tongue was red—considerable thirst. Since her infancy, this woman had been engaged in rural labours. She was treated with aperients, cooling drinks, and light, wholesome diet, and speedily got well; the erythema died away, and the skin was detached in small scales.

*Case* 2. A man, aged 47, entered the hospital in June, 1830. He had suffered slightly from the disease two years before; but, after remaining for several weeks, it disappeared on the commencement of Summer. It returned in the first week of March of this year (1830) with more severity; he experienced headache and confusion of senses, and a feeling of dragging along the spine; great debility, especially of the limbs, and lowness of spirits. The skin of the arms, hands, and feet was dry, furrowed, slightly wrinkled, and of a brown colour; here and there it was desquamating in small white or brownish scales, and was observed to be red and shining beneath these. The abdomen was tender on pressure—tongue red, no appetite, frequent purging, and loss of sensibility of the legs. He was treated principally with tepid baths, and, in the course of three or four weeks, had greatly recovered his health.

*Case* 3. A young female peasant, aged 17 years. She was much wasted in flesh, and her face indicated great distress. Tongue red, especially at the edges, and white in the centre; sensation of burning heat in the throat—abdomen painful on pressure—diarrhœa—pain and confusion of the head—extreme weakness of the lower limbs—frequent darting pains along the spinal cord. The skin of the nose and cheeks was covered with small yellow scales, and a similar appearance was seen on the bosom. The skin of the arms, hands, and feet was brown, dry, rough, and hard, and presented a dappled appearance; here and there were a few vesicles, and in other places the surface was scaly. She was treated with tepid baths, aperients and refrigerants, and wholesome diet, and speedily recovered.

*Case* 4, *in which the Disease had existed for* 18 *Years.* A woman, aged 36, was first affected with symptoms of pellagra in 1812. At that time they were not severe, and did not prevent her from engaging in her labours in the country; the disease always abated during the Winter. When she entered the hospital, in May, 1830, the following is a report of her case. Irritation in the throat and stomach, purging, great emaciation, aspect of old age; tongue red, pulse full and frequent, epidermis of the arms, hands and feet dry, rough, brown, scaly on the fore-arms, and forming a sort of hardened cuirass on the fingers; when the scales were removed, the subjacent skin was white, and little altered. Leeches behind the ears, and a treatment similar to that pursued in the former cases was adopted with good effects; but there was every chance of the disease returning on the next year.

*Case* 5. Exhibiting the Effects of Pellagra on the Mind.

A woman, aged 25, had suffered several times from pellagric symptoms, but they were much aggravated in 1830; and when she entered the hospital, the following were the symptoms; skin rough and very dry, in some places scaly; on the fore-arms and hands it was brown and appeared like a long glove drawn over them; when minutely examined, it was found covered with very minute scales, like those of a fish. For several days there was delirium, or rather alienation of mind. The state of the tongue and of the abdomen indicated gastro-enteritic irritation; considerable diarrhœa. Leeches and cooling aperients were ordered. The cerebral irritation gradually declined; and she then complained of a dull pain in the spinal cord, and utter inability to support herself on her legs. Under the use of the baths and the above treatment, she gradually recovered her health in a considerable degree.

In the third stage or degree of the disease, the mental faculties become quite unhinged, and the bodily affection is much aggravated; the frame wastes, the features become sharp, complexion earthy, and aspect mournful and afflicted; headache, redness of conjunctiva, roughness and scaliness of the skin, which becomes disfigured with grooves and chaps; the reason falters, the mind is haunted with gloom and fear, and too often the wretched sufferer becomes quite lunatic, either moping in silent grief, or raving with madness. The most common species of mania is, however, a religious fatuity.

*Case* 6. Confirmed Pellagra, resembling considerably Elephantiasis, and the Lepra of the Ancients.

The face of the patient was much emaciated, of a clear yellow hue, features very sharp, had long suffered from gastric irritation and protracted diarrhœa. The skin of the upper extremities from the fingers upwards to four inches above the elbow was covered with scales of a deep brown colour, very thick, especially on the back of the hands and fingers, so as to resemble the horny elevations or tubercles which we notice on the backs of some fishes; it was crossed in all directions with deep furrowed lines, which thus divided the hardened patches into numerous small, rough, coarse, and elephantiasis-like tubercles. The issue of the case is not stated.

When pellagra affects young children they are generally found rickety at the same time, with large tumid bellies, and marasmus.

The following cases will illustrate the pathology of pellagra.

*Case* 7. A man, aged 44, had suffered for 10 years. The skin of the fore-arms, hands and feet, presented the usual appearances, previously enumerated; the mind had become affected; the vision imperfect, and sometimes double; he could with difficulty stand upright, and he felt as if there was a weight which dragged the head and spine backwards. He died suddenly.

*Autopsy.* Not much emaciation; the bones of the cranium were thickened; the arachnoid adhered in some places to the pia mater, and between this latter membrane and the brain, there was a copious deposition of an opaline, gelatinous substance; the meninges were injected with blood; substance of brain not changed; a considerable quantity of blood found at the base of the brain; a copious intermuscular bloody effusion over the lower cervical, and upper dorsal vertebræ; the membranes, especially the arachnoid of the spinal marrow, were uniformly red ; vessels much gorged, and a frothy serum in some parts. The cineritious substance was firm ; the medullary portion much softened, especially at the upper part of the cord ; the lungs were affected with pulmonic apoplexy ; the stomach exhibited an inflammatory redness ; a similar appearance was noticed in some parts of the intestines—other viscera healthy.

*Case* 8. A woman, aged 66, from the country, entered the Milan Hospital, labouring under enteritis, and also pellagra, of a long standing ; the tongue was dry and red, the pulse thread-like, bowels much relaxed, features sharp, limbs anasarcous ; she gradually sank from exhaustion.

*Autopsy.* Effusion of serum in the pleuræ and pericardium ; lungs nearly healthy ; heart not affected, except that the left ventricle was hypertrophied ; liver and spleen much congested, but of natural consistence ; a little serous effusion in the abdomen ; mucous coat of stomach affected with chronic inflammation ; meseraic vessels of a brownish hue ; and intestines exhibited patches of long-protracted plegmasia ; dura mater adhering in several places to the arachnoid, which was found thickened, nacreous, and opaline ; pia mater highly injected ; substance of brain denser than usual. Meninges of spinal marrow not much changed ; medullary matter very soft and creamy throughout the whole extent.

In conclusion, we give a comprehensive statement of the symptomatology. At first, a general lassitude, anorexia, and dyspepsia ; the tongue is white, yellowish, or red ; the belly tender ; thirst ; inability of active exertion, and speedy fatigue ; headache and vertigo ; pulse frequent ; sense of heat along the spine, and darting thence over the rest of the body, generally fixing itself in the soles of the feet ; tendency to gloom and lowness of spirits ; after a longer or shorter period, the skin becomes affected ; and this occurs almost always in the Spring months, and ceases in Autumn ; the earliest appearance is that of fulness or distention, accompanied with a feeling of burning heat in the part, which is usually the arms, hands, and fore part of the chest. An erythematous blush, which is termed "erythema solare," by the Italian physicians, next appears, sometimes in patches, at other times spreading more continuously ; as the redness abates, the part is covered with whitish scales. The health of the patients, at this early period of the malady, is always re-established when Autumn comes round ; again the symptoms return on the approach of Spring, and each successive year, with additional severity ; the feebleness and lassitude become greater ; the headaches more distressing and stupefying ; the patient feels dizzy, and his vision is impaired ; the feeling of a weight, and of a dragging of the head and spine backwards adds now much to his sufferings ; the general sensibility is much diminished, and he cannot grasp any thing in his hands ; and his tongue and lower jaw are in constant trembling motion, which he cannot restrain ; his mind becomes more and more sad, until complete alienation supervenes ; at this period of the disease, the tongue is redder than before ; there is a burning heat in the throat, excessive thirst, irregularity of appetite, and commonly a diarrhœa ; the skin has become dry, thickened, like parchment, rough, and furrowed with lines, or with chaps, and small vesicles are scattered over some parts of its surface, while others are covered with scales ; and when these are removed, the skin is observed of a shining red, or of a dirty white appearance ; when touched, it feels like a piece of moistened paper ; the most remarkable thickening of the skin occurs on the hands, fingers and feet. Sometimes, though rarely, the skin round the eyes, of the nose, temples, forehead, cheeks and ears, is affected with the pellagric cutaneous disease. When the patients, even in the second stage, have been under judicious treatment for two or three months, they speedily seem to recover their health ; but the amendment is only temporary, and the next year all the symptoms in a worse form are renewed. In some cases, the disease remains stationary for a number of years, say from 10 to

40, and if the patient fortunately removes from the districts where it prevails, to a more salubrious climate, he may conquer entirely its effects; for example, a man born of pellagric parents, and who had exhibited the symptoms of it when young, became a soldier, and served for 15 years in Hungary, France, and Germany, without experiencing any attack. He returned to his native country; the disease reappeared, and it attacked him every year. And again, a woman who had been subject to the disease more or less from her childhood, was made a nurse in the Milan Hospital; from that period she recovered quickly, and retained her health;—after some years she went back to her native country, and the disease as speedily was renewed.

In the third, or last stage, the face becomes yellow, earthy, shrivelled, and pinched to sharpness; the bones project almost through the skin, the eyes are sunk, the tongue is dry and black; there is insupportable thirst; appetite is quite gone; abdomen tender; profuse diarrhœa which cannot be checked; there is general anasarca, and the solid parts are wasted; frequently, offensive fetid sweatings, convulsions, or paralysis come on, and quite deprive the wretched sufferer of any power of motion, and at the same time he becomes idiotic or mad; the cutaneous disease not unfrequently at this period resembles icthyosis, or elephantiasis, and the fingers are encrusted with a cuirass of hard tuberculated skin; the patient dies either of dysentery, marasmus, typhus, scurvy, phthisis, or some other organic visceral disease.—*Journ. Complément.*

The above remarks are from the pen of M. Brierre de Boimont, who visited Italy in 1830; they are well worthy attentive perusal, as they faithfully delineate one of the many ills which afflict the peasantry of fair Italy. We can vouch for their accuracy, from our personal observation. It may be worth the reader's while to compare the preceding description with what we have written on the same subject in the "Change of Air," vide pages 75, 6, 7. 2*d. edit.*—Ed.

### XVIII.

### Bill of Mortality in Paris, in 1830.

| | No. of Deaths. |
|---|---|
| From Pulmonary Catarrh | 3535 |
| Phthisis | 2948 |
| Enteritis | 2452 |
| Pneumonia | 2150 |
| Gastritis | 1997 |
| Convulsions | 1880 |
| Apoplexy | 1308 |
| Cerebral Fever | 1288 |
| Scirrhus and Cancer | 602 |
| General Dropsy | 386 |
| Aneurism | 372 |
| Peritonitis | 331 |
| Hydrothorax | 358 |
| Small-pox | 329 |
| Measles | 224 |
| Croup, hooping cough, dentition, scarlatina | 700 |
| Children still-born, or which died soon after birth | 2720 |
| Old age | 958 |

*Remarks.* The fatal cases of pulmonary catarrh occurred chiefly in children under five years of age; in adults about 30 years old; and in aged people from 65 to 80 years.

With regard to phthisis, it appears that it most frequently manifests itself in young persons of 14 or 15 years of age; that it goes on with its destructive ravages, from that period of life, till they reach and pass the age of 40, after which its frequency declines. Many more females than males fall victims to consumption. Of the enteritic and gastritic cases, three-fourths occurred in children, under the age of five years. The cases of small-pox occurred chiefly in children under 10 years. Those of apoplexy in persons between the ages of 40 and 65, and still more frequently between 65 and 75; very rarely in those more advanced; a few cases occurred in young children, but none in those between 5 and 20 years. The total mortality in Paris amounted to 28,503 deaths, of which 14,046 were males, and 14,457 were females. The number of deaths in public institutions was 10,009, and in the arrondissements

18,494. The part of Paris in which the mortality was the greatest, was the district of the Hôtel de Ville, where one in 31 of the population died; whereas, in that of La Chassée d'Autin, only one in 63 died. The following table may be interesting to such of our readers as are acquainted with the topography of Paris:—

| Districts. | Population. | No. of Deaths. | Proportion of Deaths to Population. |
|---|---|---|---|
| 1 Palais de Justice ........ | 3043 | 182 | 1 in 37 |
| 2 Ile St. Louis ............ | 6073 | 411 | 37 |
| 3 St. Eustache ............ | 9877 | 225 | 44 |
| 4 Tuileries.. .............. | 9966 | 312 | 32 |
| 5 Arcis.................... | 10602 | 240 | 44 |
| 6 Marches ................. | 10766 | 308 | 35 |
| 7 Montmartre ............ | 10973 | 184 | 60 |
| 8 St. Honore ............... | 11006 | 231 | 48 |
| 9 Louvre.................. | 11215 | 292 | 39 |
| 10 Mail .................... | 11387 | 187 | 63 |
| 11 Sorbonne................ | 11688 | 358 | 33 |
| 12 Banque.................. | 11747 | 237 | 50 |
| 13 Cite .................... | 11925 | 328 | 36 |
| 14 Arsenal.................. | 11960 | 325 | 36 |
| 15 Bonne Nouvelle.......... | 12511 | 300 | 42 |
| 16 Hotel de Ville............ | 12598 | 411 | 31 |
| 17 Champs Elysees.......... | 13274 | 294 | 45 |
| 18 Mont de Piete............ | 14885 | 334 | 45 |
| 19 Lombards .............. | 14974 | 376 | 40 |
| 20 Marche St. Jean ........ | 15141 | 412 | 35 |
| 21 Montorgueil ............ | 15326 | 391 | 39 |
| 22 Teydau.................. | 15734 | 314 | 50 |
| 23 Ecole de Medecine ...... | 15766 | 336 | 47 |
| 24 Faubourg St. Germain.... | 15928 | 276 | 58 |
| | 287735 | 7264 | |

*Ibid.*

### XIX.

### ABSCESS OF THE LUNG COMMUNICATING WITH THE SURFACE.

A YOUNG man, aged 16, of a delicate and scrofulous constitution, had for many years been troubled with enlarged cervical glands. Two years ago an abscess, situated in front of the trachea, and below the upper end of the sternum, made its appearance; it broke, healed, and broke again, leaving a fistulous opening on the fore part of the neck. At the same time several strumous abscesses formed on the thighs; and he had always a cough which was worse during cold weather. The discharge from the neck abscess was checked; and immediately afterwards the cough was aggravated, and he felt sharp pains behind the sternum. During a violent fit of coughing, a considerable quantity of pus mixed with air was expelled from the orifice in the neck; and from this time the symptoms became more alarming, and he speedily died hectic. Before death there were two fistulous apertures by which the air entered and was expelled during inspiration and expiration; the discharge was not of unhealthy appearance, but very fetid.

*Dissection.* The two openings of the neck led inwards to an abscess seated between the sterno-mastoid muscles, and between the superficial and deep cervical fasciæ. At the lower part of this abscess was found an aperture, which proved to be the extremity of a fistulous passage, which led deep into the chest, through the texture of the lungs, till it reached the upper portion of the inferior lobe, and there it opened into an abscess of an inch and a half in diameter; the abscess was empty, its walls smooth; two or three bronchial tubes opened on its surface; the situation of this abscess was on the superficial or most anterior portion of the lung, and separated from the triangularis-sterni muscle, only by a very thin layer of pulmonary substance. The rest of the lungs was nearly normal, and quite free of any tubercular deposit. Other viscera sound.

*Remarks.* The above must be regarded as an instance of simple abscess unconnected with any softening of infiltrated tuberculous matter.—*Ibid.*

### XX.

### CASES OF SPONTANEOUS GANGRENE.

A MAN, aged 61, of a sound and strong constitution, was admitted into the Hôtel Dieu; there was an immense phlyctena, covering the lower third of the left fore-arm; the ring and small fingers on the right side were similarly affected; the skin around these vesicles was red, but in other parts of the limb of a purplish colour, and gave out a peculiar gangrenous smell. When the raised cuticle was removed, the subjacent surface was at first reddish, but subsequently became of a slaty colour, and then black; he could assign no cause. The sores were dressed with chloruret of soda, and healed slowly in the course of three months and a half, during which time he took tonics largely.

*Case* 2. A water-carrier, æt. 73, had, for, five days preceding his admission into the hospital, noticed a swelling on the right cheek, which appeared spontaneously. Five leeches had been applied; gangrene had followed, and now the eschar reached from the malar bone to the lower jaw, and from the mouth to the masseter muscle; this side of the face was quite paralytic —great general prostration. He was ordered cinchona, camphor, and musk, and the sores were dressed with the chloruret of soda; in time, the whole thickness of the cheek had sloughed off, exposing to view the inside of the mouth, and speedily he died.

No assignable cause could be traced. —*Ibid.*

---

### XXI.

### Case of Poisoning

[Reported by Professor Fodéré.]

Five persons had dined off soup made of dry pease, lard, and pork sausages, and afterwards they had eaten some dried chervil; they experienced no bad effects. The supper consisted of the remainder of the soup, which had been kept in a cast-iron vessel; and no sooner had they partaken of it than all were seized with violent symptoms, viz. vomiting, intense thirst, burning and sharp heat in the throat, stomach, and bowels, great lassitude, and headache; some were also purged. It was found that, on giving some of the soup to a moderate-sized dog, it immediately vomited. The five persons continued to be more or less indisposed for several days; one, a female, became worse on the sixth day, and then consulted a midwife, who administered some "theriaque;" but next day she died. On dissection, the alimentary canal was found violently inflamed, and there were several black patches on the stomach; the contents were carefully collected, and submitted, several days after the death of the patient, to Prof. Fodéré for examination. He remarked their extreme fætor and putridity, and, from this occurrence, was led to suspect the presence of a narcotic poison, having always observed that such was not the case when any metallic and acrid poison had been swallowed. So exceedingly offensive were the contents on the present occasion, that M. Fodéré, although much accustomed to enquiries of this sort, could not remain in the laboratory till it was purified with chlorine gas. Before we state the particulars of the analysis, it is proper to allude to some of the appearances during life and after death, viz. the extreme dilatation of the pupils, the cerebral congestion, the congested state of the bronchia, from which it was at first suspected that the leaves of conium might have been mixed in the salad with the chervil. The contents of the intestines, after being filtered, were carefully evaporated, and tested with sulphuretted hydrogen, nitras argenti, sulphas et ammoniuretum cupri; but these agents produced no changes. On exposing the collected deposit from the filter to heat, in a platina crucible, no odour, except that of animal matters, could be perceived; all traces of a garlic or arsenical smell were absent. Having now ascertained that there were no traces of mineral poison, our attention was directed to ascertain if any vegetable one had been swallowed; another portion of the fluid contents was taken, and part of it treated with alcohol, part with a weak solution of muriatic acid; the fluids were then filtered. On adding oxalic acid, traces of lime were found, and muriate of barytes shewed the presence of sulphuric acid. When liquor potassæ was added, a lixivial and ammoniacal odour was disengaged, and a gelatinous or glairy precipitate, which remained in suspension through the fluid, was occasioned. When carefully filtered, the precipitate was collected from the filter and dried; it was white, confusedly crystallized, and of a salt and bitterish taste. On adding a few drops of nitric acid, the colour changed from white to red, and then to yellow, as is the case with morphine, brucine, and strychnine. It was, therefore, suspected that death had been occasioned by the "theriaque," or opiate electuary; and it was, therefore, desirable to ascertain if there were any traces of its presence in the alvine contents: as iron is the best test of opium, from its striking a blood-red colour with it, we added a few drops of a solution of murias ferri on these, previously diluted with distilled water, and the characteristic change of hue was directly perceptible. On testing the substance which had been obtained by the first process, and which, by becoming red on the addition of nitric acid, was suspected to be morphine, with the same ferruginous salt, no change was effected; it became, therefore, a question, whence had this proceeded? and we are still left to suppose, that the opium of the theriaca may, probably, during the putrefaction, become decomposed, and the morphia be thus separated from the meconic acid. Unfortunately, none of the soup was preserved for examination; and, as some theriaca had been administered as an antidote to the person who died, much uncertainty hung over the case. Besides the greasy matter of the "charenterie," or pork sausages contained in the soup, might have acted on the metallic vessel in which it had been kept; and, accordingly, the hydrocyanate of potass detected the presence of iron.

The conclusions which Fodéré draws from the preceding case are the following, and deserve attention, as being applicable to many cases of suspected poisoning:

1. That the professional person who was first summoned ought to have examined the quality of the dried chervil, and ought to have saved for future examination the remainder of the soup; he ought also to have stated the age, previous health, and habits of the patient.

2. That the five persons who had partaken of the soup, had certainly suffered from some poisoning; but that the soup did not contain any of the mineral poisons, which are usually employed for criminal purposes.

3. That it is probable that the accidents depended on some spontaneous changes which the soup underwent, while kept in the metallic vessel.

4. That the death had been promoted by the theriaca, which had been improperly administered, not, indeed, with the intention of murder, but certainly in contravention of the law of "19 Ventôse, et 21 Germinal," by the midwife, who had been applied to for the purpose of recommending a medical man to the patient. One remark more:—Two of the persons had eaten milk and potatoes after the soup and chervil, and these two suffered less, which accords with what Fodéré has mentioned in his "Legal Medicine," that farinaceous, mucilaginous, and emollient substances, swallowed before or after a poison, remarkably assuage their deleterious effects.—*Journ. Complem.*

---

### XXII.

### CHOLERA, GREAT VARIETY IN THE TREATMENT OF, IN PARIS.

WE have regretted that we have not noted down each and every mode and method which has been recommended in this country; it would form a document at once curious and instructive, at least so far as in shewing us, how the monster has laughed to scorn all the futile attempts to arrest its fury; and how frail and feeble are the grounds on which the practice of medicine, in the hands of many, is founded. No one, we think, will dispute with us, that the only rational hope of successfully treating this, or any other disease, is by a comprehensive and philosophical scrutiny of its physiology or symptoms during life, and of its pathology, or its appearances after death. We must not look to one, or to another of these singly, but group them together, and cautiously endeavour to trace every link of the chain; it is only thus that we can expect to arrive at any rational conjectures as to the causes and essence of the disease; and till then, the treatment must necessarily be empirical, and on the whole, perhaps, as inefficacious, nay, even decidedly hurtful, as beneficial. If, in any future period, a method of cure, at once successful and generally applicable, be discovered, we, or our successors, must look back to the records of this disease, with feelings of little respect for the soundness and sagacity of the present race of medical men; for we have all along permitted ourselves to be too much carried along by an exclusive or undue attention to one particular phenomenon, and on that, as on a pivot, we have made our practice turn; but it is never too late to be wise, and our wisdom will be best displayed, by endeavouring to draw some useful lessons from the blundering errors we have hitherto committed. Some idea may be formed of the general malapraxis of the French physicians, from the following report of the different plans of treatment of the cholera in the various Parisian hospitals.

*Hôp. Necker—M. Bricheteau.* Vapour-baths, aromatic infusions—general and local bleedings, sinapisms and stimulating embrocations—wine, æther, and cordials—emetics, cold lemonade, iced water, effervescing draughts, ice laid on the epigastrium, opiate enemas, opium pills by the mouth. In less severe cases, energetic antiphlogistic treatment.

*Hôp. Orphelius—M. Blane.* Bleeding, emetics, effervescing draughts, purgatives, and then astringents, if the purging is obstinate—iced drinks.

*Hôp. Piepus—M. Patrix.* Fumigation with chlorine—bleeding—emetics—narcotic decoction—iced lemonade—blisters on each hypochondrium.

*Hôp. St. Antoine—M. Mailly.* Leeches to the anus and epigastrium—venesection, antispasmodic and opiate drinks—stimulating frictions—infusion of peppermint, with acet. ammoniæ.

*Hôp. St. Louis—M. Biett.* Subnitras hydrargyri—charcoal, in doses of ʒss. every hour.

*M. Lugol.* External warmth—æther, with laudanum and acet. ammoniæ—pills of acetas morphiæ. For drink, strong tea, well sugared, acidulated, and alcoholised! against the vomitings, seltzer water, either alone or with wine.

*M. Gerdy.* General stimulating frictions—blisters along the spine—sinapisms to the arms, legs, and epigastrium—gaseous, laudanised potion—pills of camphor.

*M. Jobert.* Sinapisms along the whole extent of all the limbs—laudanum—leeches to the anus. When vomiting is obstinate, seltzer water, or the withdrawal of all drinks.

*Hôp. Salpetrière—M. Piorry.* General or local bleeding—hot aromatic infusion—Malaga wine or light punch during the collapse—iced water, and, when re-action ensues, leeches, poultices, and gum drinks.

*Hôp. Pitié.* Fresh lemonade or warm tea—peppermint and laudanum, opiate enemata. M. Andral employs ipecacuan emetics—excitants during the cold stage—local and general bleedings during re-action—opium in small doses.

*M. Lisfranc.* Tea, lemonade, and punch—enemata, with sulphate of quinine—sinapisms and stimulating frictions.

*M. Velpeau.* Sinapisms, opiates, quinine enemata, to which are added laudanum and camphor.

*M. Bouillaud.* Bleeding, leeches to abdomen, frequently repeated—iced lemonade. In the state of complete collapse, weak coffee as a drink, and drawing a heated iron along a flannel band, which has been well soaked in equal parts of liquor ammoniæ and spir. tereb. and applied over the whole length of the spine.

*Val de Grace—M. Broussais.* When there is profuse vomiting and purging, the patient should be given ice alone to swallow: when the state of cyanosis ceases, we ought to substitute drinks. No frictions should be employed. General bleeding, or, what is better, numerous leeches, and afterwards hot poultices to the leech-bites — sinapisms, vapour baths — leeches and iced water to the head.—*Journ. Complément.*

The simple treatment of Broussais coincides nearly with the cold-water practice in this country.—ED.

---

### XXIII.

### CHOLERA ANECDOTES.

A GENTLEMAN, recently returned from Paris, informs us that it was most amusing to study the French character, when cholera made its first alarming onset in the metropolis. The cafés were crowded with persons, of all descriptions, calling for "punch à Majendie;" nothing else was drank, and many a severe griping belly-ache was the consequence; but, in addition to this punch, every one took to cigar-smoking, and you might see in the streets, as you passed along, those who had not served their noviciate to the practice laying hold of any rails or balustrade, and retching and vomiting after every whiff; yet they must nobly persevere, for nothing was so effectual a preservative as smoking, they were told. If any looseness came on, it was often no easy thing to ascertain, whether one of these "jeunes hommes de tabac" were really affected with cholera, or only with the effects of the smoke. So great was the demand for cigars at first, that our friend for some days could not obtain one, at a dozen or more shops where he applied.

---

### XXIV.

### CHOLERA.

UNWILLING though we are to occupy space with details of cholera, we consider it most useful to avail ourselves of any document or reports, which tend to throw light on the history of its propagation. Few, if any, are now so hardy as to maintain that it is by contagion, and by that alone, that the pestilence has spread from India's shores to inland America; such dogmatism would not be tolerated as the opinion of a sane mind; but we ought equally to avoid the other extreme, of stoutly denying the possibility of its conveyance from one patient to another, and of shutting the ears of our minds to any arguments advanced on the opposite side of the question. The zeal and anger of party-discussion has blown over, and we now can quietly look back on the introduction of cholera into this, or any other country, as we should do upon any historical event, and dispassionately weigh the probabilities of its origin. It is, indeed, very gratifying to us, that the views which we almost alone maintained, against a battery of numerous assailants, appear now to be those of all the rational members of the profession; they have even been embraced by the very men who, at one time, most vigorously opposed them: but such is, and ever will be, the triumph of calm and impartial truth, and the only merit we assume to ourselves, is that of considering both sides of the hotly-contested question, with a fair and unbiassed mind —of studying the history of its progress from the East to our own shores, and of comparing the opinions and statements of others with our own experience, both abroad and at home. An argument at once philosophical and important, on the question—"Is cholera essentially contagious or no?" may be drawn, we think, from the undeniable fact, that long, very long, before the acknowledged irruption of the disease into any country, it had given, as it were, a colouring of its own, or a peculiar and unusual character, to other existing diseases. We shall select a case or two in proof from a paper by Dr. Sabatier, of Paris, published in the Journ. Univ. et Hebdom. It was observed by all the medical men there, that for six or eight weeks previous to the occurrence of the pestilence, there was a very general tendency, in all diseases, to be accompanied with an extreme irritability of the alimentary canal, occasioning vomiting and diarrhœa. In the month of January, M. Baudelocque observed in two children, affected with small-pox and pneumonia, that the tongue was as cold as it ever is in the collapse of cholera. The following very anomalous case exhibits other analogies.

A boy, aged 15, of a strong, healthy constitution, residing in the Rue de Seine-Saint-Germain, was brought to the La Charité Hospital on the 25th October, 1831. Four days previously he had become indisposed, complaining of pain in the left fore-arm, of general weariness and debility. On the 24th, the pain of the arm was much worse—he was very restless, occasionally delirious, and then heavy and stupid—the light incommoded him. Profuse vomiting now came on, and also convulsive twitches of the whole body; alternately he was calm and agitated—at one time sitting up and answering questions, at another throwing himself on the floor, making strong efforts to vomit, and struggling violently: the action of the heart and carotids was powerful—pulse 130, sharp—respiration every now and then interrupted by a spasmodic contraction of the muscles of expiration, to force out the saliva which dribbled from the mouth. At times he was threatened with a feeling of suffocation, and experienced a very acute pain under the ensiform cartilage—bowels obstinately costive; symptoms of hydrophobia came on—the vomiting and ptyalism increased. Bleeding and opiates were employed, but nothing could be retained on the stomach, and soon after he was incapable of swallowing; still his consciousness and intelligence were unimpaired. But a great change quickly followed, and he sunk into a state of extreme exhaustion, and died in the afternoon of the day upon which he was admitted.

Dr. Sabatier alludes particularly to the obstinate vomiting, to the rapid sinking of the features, to the retention of the mental powers, even after the patient had become icy cold, and to the sudden annihilation of the pulse, as approximating the disease, in some respects, to cholera. [We must acknowledge that the resemblance is not great. —Ed.] The dissection of the patient did not disclose any satisfactory morbid appearances.—*Journ. Hebdom.*

---

### XXV.

### Fissure of the Anus, treated without an Operation.

In almost every case of this malady, the cause may be traced to a spasmodic constriction of the sphincter ani; the fissure, is merely consecutive; by removing, therefore, the original evil, the fissure will generally heal of itself. The local application of belladonna will effect this in a great number of cases. We take some lint, and grease it well with an ointment, consisting of a drachm of the extract, a drachm of acetas plumbi, and six drachms of lard, and introduce it into the rectum. It must be repeated several times a day, and the practice is to be continued for some length of time. —*Bullet. de Therap.*

---

### XXVI.

### Ingenious Method of applying Nitras Argenti to Ulcers of the Cornea, &c. &c.

Take a silver female sound, or large silver probe, and heat an inch of its extremity in the flame of a candle; then rub lightly upon it a stick of the lunar caustic; the salt is immediately melted, and unites with the metallic surface, coating it with a thin layer of caustic; if it be too thin, we have only to repeat the same process. When the instrument cools, it must be wiped clean, and then it is ready for use.—*Ibid.*

---

### XXVII.

### Contagion of Diseases.

Dupuytren states that he has repeatedly observed, that diseases assumed a contagious, or at least a spreading character, when more patients than the regular and proper number have been admitted into the wards of a hospital, and that the calamity has been at once arrested when these were thinned, and the ward was sufficiently ventilated.—*Ibid.*

---

### XXVIII.

#### RHINOPLASTIC OPERATION.

A FRENCH soldier, at the battle of Waterloo, had his nose clean cut off by a sabre-wound, the upper lip was also divided, and five of the front teeth, with a portion of the alveolar process of the upper jaw, knocked out; but hero-like, as all Frenchmen are, he pursued his opponent, an English soldier, and had ample vengeance, we are told. The wounds healed, and for several years he wore an artificial nose of silver, and afterwards one of copper, by both of which he was much annoyed. He was persuaded by a surgeon at Rouen to submit to an operation, which consisted in dissecting a flap from each cheek, and uniting them along the bridge of the nose-to-be. But the deformity was greater than ever. M. Blandin was now consulted; he performed his rhinoplastic operation, by dissecting the flap from the skin of the forehead. The artificial nose united very favorably, and even withstood an attack of erysipelas, which spread over the face, a month after the operation. It is to be remembered that M. Blandin does not ever detach, or cut through, the pedicle of the flap; it is, therefore, obvious that this portion cannot very easily, or possibly become attacked, in consequence of its having been necessarily twisted on itself, when reflected down, unless some ulterior pruning and embellishment by the scalpel be resorted to. Other surgeons have divided the pedicle, when the rest of the flap has become firmly united; but M. Blandin does not approve of this, and prefers the following process:—With a scalpel, he pares the already-cicatrized edges of the pedicle and also the surface of the corresponding integuments, which cover on each side the nasal processes of the upper maxillary bone; these raw surfaces are then carefully applied to each other, and retained in this position by a compress and bandage.

The cure in the above case was most satisfactory, or, in the words of our Continental brother, "it was a nose, a true nose—such a nose as is seldom to be seen (we believe it)—a regular, nay, even almost an elegant nose!! and one for which many of my acquaintances would gladly have exchanged their own; and not such a hideous stump or appendix as M. Delpech, and M. Lisfranc, and others, have grafted on the faces of their unfortunate patients!!" These latter operators, we are informed, have always divided the pedicle of the frontal flap.

The advantages of preserving the original attachment of the pedicle are manifold; the nutritious vessels are preserved, and the life of the new nose is, therefore, much stronger; the skin, moreover, retains its natural colour, and does not become purple, as has been the case after most operations; besides, the tip of the nose is prevented from falling downwards on the upper lip, and thus closing up either one or both nostrils, an accident which has frequently happened, and caused much annoyance both to patient and surgeon. It may be supposed that, in M. Blandin's method, an awkward and ugly button, or protuberance, must remain at the point where the pedicle is twisted; this is a mistake, for it gradually wears away, and no deformity remains.

Perhaps it is not generally known that, if we blindfold a patient a few days after a rhinoplastic operation, and the new nose be pricked, he will refer the sensation to the forehead; but when the union becomes perfect and complete, the mistake is no longer committed; this, therefore, may perhaps be taken as a test of the proper time at which the pedicle should be divided, if the operator determines upon that.—*Ibid.*

---

### XXIX.

### On the Changes which the Points of the Fingers undergo in Phthisis, &c.

Hippocrates remarked that, in those who died of pulmonary consumption, the nails became bent—"phthisitis ungues adunci;" the assertion used to be called in question by many medical authors, but M. Pigeaux, who has directed his attention to this subject, and written "a memoir on the etiology, symptomatology, and the mechanism of the fusiform development of the extremities of the fingers," fully confirms the truth of the aphorism. He examined the hands of 200 phthisical patients, and found that 167 of these were provided with "griffes Hippocratiques." Every tubercular patient does not certainly present this phenomenon, but in other diseases of atrophy the proportion is much smaller, not exceeding one in ten; it appears, therefore, that a certain relation may be traced between thoracic maladies and the curving of the nails, although it occurs in other diseases, but certainly not so frequently. In 183 cases of diseases, not tuberculous, but which had produced great emaciation, 17 exhibited the phenomenon of the curving of the nails in a very remarkable degree; of these 17, nine were cases of organic affections of the heart—four of emphysema—two of asthma and catarrh, and two doubtful. An obvious dyspnœa existed in 13 of these cases, and also in almost every one of the 167 tubercular cases. I have no doubt that some connexion may be traced between all such maladies as create an impediment to the respiration or circulation, and the appearances of the nails alluded to, or, at least, between the former and the fusiform swelling of the last digital phalanx, with which the curving is generally associated. In 20 of the 167 tubercular cases, the patients had not lost their embonpoint. After many examinations into the cause of these phenomena, I am satisfied that the change in the points of the fingers precedes, and is the cause of, the curvature of the nails. Now this change consists chiefly in an œdematous infiltration of the pulp of these, by which the nail becomes mechanically forced out and forwards, and thus its end is curved round. As a general rule, it may be stated that the fusiform development of the last phalanx of the fingers, with the curvature of the nails, is generally indicative of the presence of tubercles, or of any derangement of sanguification. If we notice particularly the change of form, we find that the swelling begins at the articulation of the 3d with the 2d phalanx—that it increases somewhat towards the root of the nail, which becomes the most projecting part, and then it tapers off to the end of the finger: the thumb and fore-finger are generally affected first. The progress of this affection does not depend so much on the "phases" of tubercular disease, or of organic affections of the heart, as on the influence which these have on the general state of "hematosis" and of respiration. I have observed it to increase, diminish, and even to vanish, with the removal of the cause which had produced it. It is more common in women than in men; it is much more rarely seen in the toenails, with the exception of that of the great toe, the swelling of which, and the consequent "growing of whose nail into the quick, often gives rise to much pain and annoyance." To impress his readers with the importance of the above appearances, as symptoms, the author says that he has, by attention to this particular, repeatedly been enabled to foretell the severity and danger of a pulmonary catarrh, of a pneumonia, &c. which were supposed to be of an innocent nature! he, therefore, regards it as a very unfavourable sign; it exists, he says, in six-tenths of consumptive patients, and, on the whole, it is more frequently seen in those who still retain their embonpoint, than in those who are much emaciated. If the above remarks be confirmed by experience, it must be considered as a valuable adjunct in guiding our diagnosis. The anatomy of this change will be readily understood from what has been stated above; the nail, separated from the finger, appears very little, or perhaps not at all curved; but when in sitû, it is found to be elevated and pushed forwards by the infiltrated pulp *underneath;* the bone is not altered.—*Archives Générales.*

We do not vouch for the entire correctness of the preceding details, but deem them well worthy of attention by all enlightened physicians.—ED.

---

### XXX.

### CASES OF DISEASES OF THE KIDNEYS.

THE history of the pathological states of the kidneys is still very imperfect; the late Dr. Dance, who died a few months ago of cholera, one of the physicians of the Hôtel Dieu, at Paris, left some manuscript observations on this subject; from these we select the most interesting.

*Case* 1. NUMEROUS CALCULI IN THE SUBSTANCE OF THE KIDNEYS; DILATATION OF THE INFUNDIBULA AND PELVES, WHICH WERE ALSO INFLAMED.

A girl, aged 23, entered the hospital on the 12th January, 1824. Two years and a half before, she first voided some blood with her urine, and felt severe pains at the time in the loins. The urine was muddy, thick, and afterwards whitish and purulent, and scanty in quantity. These symptoms continued more or less for 18 months, at which time she experienced a feeling of great weight and heaviness in the renal region, and the urine still deposited the same puriform matter, but there was no sand or gravel mixed with it. Three weeks ago the catamenia were suddenly suppressed by exposure to cold, and from that period she has been very ill, complaining of great pain and tenderness over all the abdomen and in the loins; thirst, nausea, urine voided with much pain, and only in small quantities. Leeches were applied to the anus, and an emollient ptisan ordered. The severe pains of the abdomen were relieved, but those of the kidneys became worse and worse. Vomiting, pulse weak and feeble, facies hippocratica, announced approaching death, which took place two days after.

*Dissection.* The kidneys were found larger by one third than usual, embossed on their surface, hard and resisting to the finger in some places, and fluctuating in others. On dividing them, the scalpel grated against numerous calculi nicked into the substance of the kidneys, and jets of pus escaped at the same time from many points. Nine calculi were found in the left kidney, and 15 in the right; each of these was contained in a sort of cyst, lined with a mucous membrane, and was bathed in purulent matter. These cysts were the dilated calices and infundibula. The calculi varied in colour, being white, yellowish, or ash-coloured; many were of the alternating sort, and consisted of numerous layers of uric acid, and ammoniaco-magnesian phosphates. The proper substance of the kidneys was much wasted; the ureters were greatly thickened; bladder small, and its texture indurated; its mucous membrane affected with chronic inflammation.

*Case* 2. ACUTE PARENCHYMATOUS NEPHRITIS, WITH SYMPTOMS SIMULATING THOSE OF MALIGNANT AGUES — SPEEDY DEATH.

A mason, æt. 35, had for three weeks suffered from a severe fixed pain in the renal region, which had been preceded by an œdematous puffiness of the lower extremities. No cause could be assigned for the attack. On admission, the renal region was found to be swelled and resisting to the hand; the whole abdomen was so tense as to preclude an accurate examination; the countenance expressed great anxiety, the pulse small and rapid. Venesectio; blood inflamed. The symptoms were not relieved; the tongue became red and dry; the lumbar pain extended round to the epigastrium; and the urine was voided frequently and in small quantities; no sickness or vomiting; no pain nor retraction of the testicle, nor numbness in the groins. Shiverings, horripilation, and other symptoms of the cold stage of fever came on; he was copiously bled; the pain of the kidneys not abated. For two or three mornings successively a similar febrile paroxysm recurred; the urine became of a blackish colour, but deposited a white sediment.

On the 6th day after admission, the patient was much worse; features greatly altered; breathing difficult, and severe pain in the epigastrium and region of the kidneys. The quotidian paroxysms begin with violent shiverings. Two days afterwards he died.

*Dissection.*—*Head;* three or four spoonfuls of serum in the ventricles. *Chest.* Lungs gorged with a frothy fluid. *Abdomen.* Left kidney quadrupled in size; at its upper part was a small abscess, between its tunica propria and the cortical substance, which was of a brown and purplish red colour. Numerous small abscesses, varying in size from that of a pea to a hazel-nut, scattered through the texture of the kidney, but found chiefly near to its surface; here and there the pus appeared to be infiltrated through the renal tissue, which had become much softened and converted into a flaky detritus; its colour was generally a reddish brown, but marbled with white points of suppuration. These morbid appearances were most conspicuous in the cortical substance. The pelvis was sound, and also the corresponding ureter. The right kidney was wasted, of a firm resisting texture, and not exceeding in size a hen's egg; its surface was irregularly undulating and embossed, as we observe in the fœtal state; pelvis and ureter healthy and quite permeable. Bladder contracted on itself; half filled with a thick muddy urine, like a decoction of bran.

*Remark.* The preceding case is one of inflammation affecting the parenchyma of the kidneys, and not as in ordinary nephritis, the mucous membrane of the pelves and infundibula. The symptoms, with the exception of the fixed and severe pains in the loins, very much resembled those of a malignant intermittent, or perhaps rather, remittent fever; the quotidian aggravation was very remarkable. Physicians should attend to this.

*Case* 3. Hypertrophy and Ramollissement of both Kidneys, giving rise to general Dropsy.

A female, aged 32, stated that she had been, for 18 months, more or less affected with dropsy of the legs and belly. No cause could be assigned. The heart was deemed sound, upon auscultation; there were no palpitations or dyspnœa,—could lie easily in the horizontal posture. A constant dull pain in the right hypochondrium; with the exception of this the patient complained of no other uneasiness. Urine thin, limpid, and very scanty; thirst moderate, pulse small. The disease increased in spite of diuretics, and she died suddenly and unexpectedly a month after her admission. The symptoms were altogether of a negative nature.

*Dissection.* Cellular texture loaded with serum. Half a pint of effusion in each pleura. Lungs and heart healthy. Several pints of serum in the abdomen. Liver healthy, although it was of a colour somewhat yellowish; other viscera sound, except the kidneys, which were greatly enlarged, and also softened in texture; their colour was that of yellow wax, the tunica propria adhered very loosely to the cortical substance; which was the structure chiefly affected, the tubular portion appearing healthy; the contrast between these two was very marked; by scraping with the finger, and having a stream of water to play on it, the whole of the former might be washed away, so soft it was, while the central medullary part was left. The infundibula, pelves, &c. were healthy.

*Remarks.* The preceding is a good illustration of that species of dropsy which is caused by an organic change in the texture of the kidneys, and which Drs. Bright and Christison first made known. We have already stated that the thoracic viscera were sound; and also the liver, which are the organs, to diseased states of which dropsy is usually referable; but in the present case, the "origo mali" was in the kidneys, and in the secretory part of these, in consequence of which they no longer can relieve the system of superfluous water of the system. Dr. Bright states, that the urine is albuminous in all cases of renal dropsy, and he regards this condition as pathognomonic. Dr. Christison has in similar instances detected urea in the blood. As far as our observations extend, we should say that the quantity of urine is very unusually small, and that it diminishes in the progress of the disease. The thirst is also not so considerable as in other dropsies.

It is still a question, what is the true nature of the changes which the kidneys undergo; it is too much the case in the present day to ascribe every example of ramollissement to a slow inflammation; there is seldom any pain, and if there is, it is rather a dull heaviness than an acute feeling; hæmaturia in several cases has preceded the first symptoms of dropsy. Many cases of what have hitherto been deemed essential, or idiopathic dropsy, are probably of the character of the above-related.

*Case* 4. A Contraction and Obliteration of one of the Ureters, with Suppuration of the Kidney.

A man, æt. 73, was brought to the hospital in a state of insensibility; on examining the stomach, a large swelling was felt in the left hypochondrium, extending from the edge of the ribs to the crista ilii; the patient could give no account of it. He died in an epileptic fit on the following day.

*Dissection.* Ramollissement of part of the cerebellum. The tumour of the left hypochondrium arose from a diseased kidney; it formed an immense cyst, which distinctly fluctuated under the finger; it extended from the diaphragm to the iliac fossa; when punctured, nearly three pints of true pus flowed out. The pus had not been contained in one bag, but in numerous compartments separated from each other by imperfect partitions; and each of which was lined with a distinct mucous coat. The outward walls of the kidney varied in thickness from one to three inches, and here and there presented some traces of cortical renal tissue; no calculus was found. The ureter was so enlarged as to resemble the large intestine; its upper extremity formed part of, and could not be distinguished from, the pouch of the kidney; it was much thickened, and strongly fibrous; an inch from the bladder, it resumed its natural dimensions, and had been converted into an impervious hard cord. The bladder was healthy. The right kidney double its usual size, and of normal structure.

*Remarks.* It is probable that the contraction of the ureter was the original cause of all the above mischief.

*Case* 5. Diabetes Mellitus, following Anti-syphilitic treatment—injurious Effects of an Animal Diet.

A man, aged 24, a short time after he had passed through a course of mercury, found the following symptoms come on; heat and dryness of the mouth; extraordinary thirst; and great increase of urine: he became gradually worse, and when admitted, his face indicated much distress; a yellow circle round his eyes; extreme emaciation and debility; skin dry; thirst intolerable, with a feeling of pain in the epigastrium; bowels costive; in the course of a night he drank more than 12 pints, and voided by urine as much; during the day the drink exceeded the excretion; the urine was transparent, almost colourless and tasteless, but sugar was found on analysis; for 10 days he was put on an exclusive animal diet; but his stomach could not bear it, and moreover no satisfactory result was obtained. A profuse diarrhœa supervened, and while this lasted, the diuresis was greatly diminished. Symptoms of hectic followed and he died three or four months after the commencement of the disease.

*Dissection.* Extreme emaciation; no vestige of fat; thoracic viscera sound; chylopoietic viscera nearly normal. There was only one kidney, and this was placed transversely across the spine; on each lateral extremity was found a supra-renal capsule, situated in its natural position; the shape of this single kidney was like that of a horse-shoe, convex above; its thinnest portion was that which rested on the vertebræ, and in bulk it equalled two kidneys of ordinary dimensions; the transverse length being from seven to eight inches, and its perpendicular from three to four; its texture and consistence were quite normal; perhaps only a little gorged with blood. The two pelves were directed forwards, and not inwards as usual; there were two ureters which were dilated, equalling in dimensions the little finger. In short, in the present, as in most other cases of this disease, the pathology is most unsatisfactory; perhaps we should look not so much to the secreting organ, as to the pabulum of secretion, viz. the blood. Animal chemistry may in time elucidate the subject.—*Archives Générales.*

---

### XXXI.

### Dothinenterite.

Our readers are probably aware that this appellation has been given by M. Bretonneau and others, to the diseased states of the glandulæ Brunneri et Peyeri, which are the mucous follicles found on the inner coat of the intestines, chiefly of the small ones, and are arranged either in groups, or are solitary; hence they are also named congregatæ et solitariæ. In the duodenum they are very distinct, especially near the pylorus, and in the ileum towards its lower extremity. Some physicians have ventured so far as to attribute the proximate cause of typhus fever to ulceration of these glands; but this opinion is certainly erroneous, as we shall be satisfied by reporting a case or two.

*Case* 1. A man, aged 21, had been troubled for a few days with diarrhœa; suddenly all the symptoms of adynamic fever came on, viz. prostration, stupor, deafness, starting of the tendons, &c. &c., and he died on the 10th day after.

*Dissection.* Patches of redness on different portions of the stomach and intestines; here and there the mucous surface presented numerous small ulcerations; the glandulæ Brunneri were tumefied, encircled with a red areola; some of them already ulcerated, so as to expose the subjacent infiltrated cellular substance. Towards the cæcal end of the ileum, numerous groups of distinctly projecting laminæ, which were hard, white, or slightly tinged with bile, and altogether resembling the crusts of some herpetic eruptions, (dartres crustacées). These laminæ or crusts were easily removed, being attached only by very slender filaments to the enlarged orifices of the mucous glands, which however did not appear ulcerated, but only redder and more prominent than is natural. The ileocæcal valve was nearly destroyed by these crusts. In the large intestines many aphthous spots were found.

*Case* 2. Was very similar in symptoms to the preceding, but *no* dothinenterite existed. A man had for some time an aphthous ulceration of the mouth, but most unexpectedly he sunk rapidly, in the course of one night, into a state of typhoid prostration; a profuse diarrhœa had also supervened, and he died two days after.

*Dissection.* Traces of extensive gastro-enteritic inflammation, especially in the jejunum and ileum, towards the cæcal end of which the redness became more and more intense. The large intestines were similarly, but in a much slighter degree, affected; no mark of disease in either set of glands.

*Remarks.* The preceding case was one of extreme rapidity. M. Bretonneau has never found the morbid alterations of the glands earlier than on the fifth day of the dothinenteritic fever, and even at that period they were by no means much reddened or tumefied. It appears, therefore, that the worst, or adynamic cases of typhus, do not always, if ever, depend solely on any affection of the mucous intestinal glands; and the truth of this statement is still further confirmed by the fact, that these glands may be greatly diseased, and yet not give rise to any symptoms of fever, as in phthisis: we relate one case as it occurred at the same time that dothinenteritis existed in the hospital.

*Case* 3. A young girl died of phthisis; a diarrhœa had existed for some time.

*Dissection.* We need not specify the thoracic disease. The stomach and duodenum were normal; the rest of the small intestines presented the appearance of capillary injection, which was more distinct towards the lower extremity; the mucous glands were red, inflamed, and prominent; near to the cæcal valve they were ulcerated.

*General Observation.* From the preceding cases, we infer that the only uniform and constant symptom of disease in the glands is an obstinate diarrhœa.—*Ibid.*

---

XXXII.

LARYNGEAL FISTULA, CURED BY A TALIACOTIAN OPERATION.

M. VELPEAU records an interesting case; it had existed for more than a year, and several surgeons, among whom was Dupuytren, had attempted in vain to heal it, by incising its edges and approximating them. A flap was dissected from the front of the neck, below the fistula, reflected upon itself upwards (so that the cutaneous surface was made the central or interior one), and fashioned into a plug or stopper, which was kept in its place by two needles and the twisted suture. The greater portion united, and one application afterwards of the cautery, to the part which did not heal, completed the cure.—*Ibid.*

---

XXXIII.

SYPHILIS, TREATED BY THE EXTERNAL USE ALONE OF MERCURY.

M. MALAPERT, a military surgeon, communicates the results of his experience, which, he states, is highly in favour of merely employing a strong solution of the corrosive sublimate, without any internal medicine. He has, in this manner, cured chancres of the throat, lip, penis, anus, and legs, by using a lotion of 8 grains to an ounce of distilled water, and applied with a camel-hair pencil. Chronic buboes he blisters first, in order to remove the cuticle, and then he cauterizes the denuded surface with a solution of a scruple of the sublimate to the ounce of water.—*Révue Med.*

We can confirm the accuracy of M. Malapert's remarks, by appealing to numerous cases of primary syphilis, which we have for several years past treated in the above way. The surface of the sore is rendered white on the application of the strong wash, and the surrounding skin is cauterized; the diseased action appears to be speedily changed, and we find, in the course of a few days, perhaps a much larger exposed surface, but certainly one that is much healthier, and disposed to cicatrize quickly, instead of the obstinately-indolent base and callous edges, so characteristic of the true chancre. Once or twice, we have discussed chronic glandular tumours by the topical use of the corrosive sublimate lotion.—REV.

---

XXXIV.

CANCER OF THE TONGUE, BY WHICH THE ORGAN WAS WHOLLY DESTROYED.

PROFESSOR DELPECH reports the following case.

A young man, aged 20, of a weak and irritable constitution, was the patient. At first, a pustule appeared on the left side of the tongue, midway between its edge and centre. Although its base was hard, and its edges were ragged, lunar caustic had been repeatedly applied; the ulceration increased and became more painful. In a short time, the cancerous mass attained the size of an orange; the mouth could not be closed, and the saliva dribbled from the lips. In this state Delpech saw him, and, as the tumor appeared to be circumscribed, and not blended with the substance of the tongue, he resolved to operate. He made a deep perpendicular crucial incision through its entire thickness, but not beyond its inferior margin or cyst, in order that the lingual and ascending pharyngeal arteries might not be cut; for, says Delpech, the normal vessels, around a defined tumour, never pass through its envelope or cyst; it has vessels of its own, and these are separated from the vessels of the adjacent parts by the proper and generating envelope of the diseased growth. The operator then laid aside the knife, and, with his fingers, worked or scooped out the four parts of the tumor from the inner surface of the sac, without any hæmorrhage. The muscles of the tongue had been absorbed by the pressure of the mass, and only the point had been left; and this was joined to the inside of the mouth merely by the mucous membrane, in which ran the ranine vessels. The next object was to destroy the cyst: for this purpose the cautery was employed, the lingual arteries having been previously tied in the neck; the cautery was applied twice again, at the intervals of six and ten days. The cavity thus left would have admitted an orange; it healed up most favourably. On the 15th day after the operation, the patient could articulate some sounds, and in course of time he recovered his speech astonishingly well. The guttural and labial letters he could pronounce distinctly, but the lingual, such as L, R, S, T, feebly and incorrectly. The sounds formed from L appeared to be rendered more liquid, as in the word "Llorente" of the Spanish language, or in the French word "bataille;" the sounds of R were given in a lisping manner—those of S not well—but those of *Th* much better. The greatest difficulty was in articulating the sounds of T and D. The sense of taste was not much impaired; and, from the results of this case, it appears that, if any portion of the skin of the base of the tongue is saved, the gustatory power remains.—*Ibid.*

The apparent success of the preceding case reflects the highest honor on the boldness and skill of the distinguished surgeon.

---

### XXXV.

### On the Etiology of Cholera.*

I. "An Examination into the Etiology of Cholera, founded on the Phenomena of the Disease, as exhibited during its prevalence in Glasgow and suburbs," has been sent forth by Dr. Bryce, and a perusal of it induces us to lament that its author had not been "bred to the bar," for assuredly he would have proved to be one of its brightest ornaments, as far as the art of making "the worse appear the better cause," or rather of proving to a demonstration, that "black is white and white is no colour at all." He has gone so artfully and casuistically to work, that one would suppose, from the train of data which he first lays down, and from the candour of his arguments and deductions, in the beginning of the paper, he was a man in search of truth, and not enlisted under the banners of any party, in the controversy respecting the etiology of cholera; but a deeper acquaintance with his lucubrations, shows that he is the very champion of the ultra-contagionists!

We care little how many converts he may make in this cause, since we are perfectly convinced that, among those who have seen most of the epidemic, and who are most capable of forming a true estimate of its etiology, few indeed will come to the conclusions which Dr. Bryce has drawn. We shall give the DATA first, and then offer a few remarks on Dr. Bryce's inferences.

"The principal facts, connected with the phenomena of cholera, as these have been exhibited during its prevalence in this city, are,

1st. The disease was officially announced to exist in Glasgow on the 12th February; but it is certain that unequivocal cases occurred for some days prior to this date.

2d. Its commencement was not preceded, nor accompanied, by any sensible unusual constitution of the atmosphere,

* Glasgow Journal, No. XIX. Aug. 1832.

as regards temperature, moisture, or electricity.

3d. It augmented gradually until the sixth week of its prevalence, when the weekly returns showed 140 cases. From this time it declined steadily till the sixteenth week, during which only 5 cases were reported, and at the end of which one case remained under treatment. Independent of any cause referrible to atmospheric intemperament, cases again multiplied rapidly to 120, the nineteenth week, since which time it has been gradually diminishing.

4th. *A very large proportion of the cases occurred in lanes, closes, and houses, remarkable for domestic and public filth.*

5th. Of these cases, many occurred in the same dwelling, within one, two, or three days of each other.

6th. It has been observed, that several cases have followed one another in the same tenement, so long as one diseased remained in the tenement; and that the disease has disappeared immediately and effectually therein, on the temporary removal of the infected, and all the healthy persons, from the locality, and on employing various means of purifying the same.

7th. *The persons attacked in these localities, were for the most part, ill-fed, badly clothed, of dissipated habits, residing in densely crowded, imperfectly ventilated, dwellings.*

8th. Under the preceding circumstances, females formed a large majority of the cases, and of those, persons of irregular lives.

9th. Comparatively few instances of the disease happened, among the middle and upper ranks of the community, *and no example was reported in these ranks of a succession of cases in a family; even although members of the family attended, as nurses, on the patients; nor did any case occur of a child or young person having been seized.* During the second exacerbation of the disease, the exceptions to this, and the two preceding conditions, were more numerous.

10th. *Numerous examples have offered, where children have slept with infected parents, nurses with patients, without contamination, as well in the chamber where the disease occurred, as in hospitals.*

11th. The town's hospital was the only public institution in which it prevailed.*

12th. Many examples have occurred of persons being seized while occupied at public works; yet, in no instance did it prevail in any particular work, and several remained altogether exempt.

13th. Of 52 nurses employed, in succession, at the Albion Street Hospital, 8 took the disease, and of 5 at the Dalmarnock Road Hospital, 2.

14th. *No one of the several medical attendants at the cholera hospitals, nor, with one exception, any one of the district surgeons, or their pupils, has been affected with unequivocal symptoms of the disease.*†

15th. The disease did not advance in any particular direction, nor follow any regular or cognizable route.

16th. *Cases have appeared on the same day, in quarters of the town most widely apart, in dwellings the most dissimilar, and among individuals the most different in their habits of life.*

17th. *Districts of the city having the greatest intercourse with, and approximating closely to, each other, have exhibited the opposite states of great prevalence, and total exemption, and this, too, for considerable periods.*

18th. The disease appeared suddenly, and prevailed largely in the Gorbals, a quarter strikingly exemplifying the conditions last specified."‡

---

* "Persons apparently healthy, removed from infected houses to places of refuge, have been subject to attacks at different periods, not more than three or four days after their removal. Five cases occurred in Bridewell, apparently under similar circumstances with those which happened in the Houses of Refuge."

† "At least 150 practitioners and students have attended on cases of cholera, yet, with the above exception, as far as is known, not one of them, nor of their households, was infected."

‡ "I have the satisfaction of stating,

The author expends many arguments, and a good number of pages, to prove that cholera was unconnected with any particular state of atmosphere.

"Common experience and meteorological observations have proved, that, during the prevalence of cholera here, neither its increase nor decrease, its development in one quarter and extinction in another, have been determined by, nor, in any observable manner, been dependent on, particular states of the atmosphere, as regards temperature, moisture, prevalence of winds in one quarter, storms, or calms. These are facts, in my opinion, irreconcilable with every explanation of the propagation of the disease by a general atmospheric influence."

We will not quarrel with the ingenious author on account of this conclusion, though we hold it to be rather unsafe to deny a general atmospheric influence, because we cannot prove, by chemistry, that there is some general morbific ingredient in the atmosphere.

The Doctor's theory is easily summed up. He maintains that the primary and essential cause of cholera is a specific contagion, generated in the human body—*but*, that this contagion can only be brought into operation in localities, where the air is corrupted by effluvia from the earth, or substances on the surface of the earth. He denies contingent contagion in fevers, from accumulation, &c., and considers influenza a contagious disease. The following short passage contains the pith of our author's doctrine.

"In a former paper in this Journal (for February), I endeavoured to show that the remote causes of fevers, and the laws of the propagation of contagion, may be reduced to a scheme conformable to the diversified properties of morbific agents (No. 17, p. 67). According to the principles of etiology then proposed, it was argued, that the elements of decomposed organic matter form a compound miasm, which acts as the essential vehicle of the specific contagion of cholera;* in other words, that cholera can be propagated only through the medium of air rendered impure by putrid animal and vegetable exhalations."

Thus, then, vegeto-animal exhalations are as necessary for the development of cholera as the specific miasm of contagion! The contagionists themselves will assure the Doctor, that the cholera has destroyed life in some of the most airy and cleanly parts of town or country. What! were Belgrave Square and Portland Place infested with putrid exhalations, when some of their fashionable inhabitants fell victims to cholera? Dr. Bryce affirms that these exhalations can never produce cholera without the specific contagion. Indeed; had he practised in tropical climates, he would have found the disease produced by these causes every year. Are the diseases described by Curtis, Paisley, and hundreds of others, half a century ago, to be struck out of the list and go for nothing? Did cholera never occur till 1817? And does not Dr. Bryce recollect, that what he calls cholera is only the highest grade of the disease, the infinitely greater number of cases being *bowel-complaints?* that these bowel-complaints shade off into a thousand other grades and forms of the same disorder, and arising from the same general cause? He tells us that—

"The principles embraced in the etiology here offered, are, that the living system under cholera eliminates an infectious poison; *that this poison cannot be extended nor reproduced in a pure air, or one impregnated simply with healthy human effluvia;* but in one rendered impure by putrescent organic exhalations, the same poison meets with that pollution, by the means of which

---

in proof of the accuracy of the facts detailed here, that the manuscript was submitted to the inspection of Dr. Corkindale, the gentleman, from his official situation, best qualified to pronounce on its correctness; and that his opinion corroborated each particular of the above statement."

---

* "Plague, yellow fever, and certain species of dysentery were included in the same class."

its specific power can alone be manifested; and that the body must be under the influence of this poison, in order to generate a similar source of infection."

To this we reply, that people have been affected by the epidemic, in all its forms, where there was nothing like impure air, in the sense here taken—consequently his hypothesis fails. But why waste words on a theory which includes typhus, yellow fever, and dysentery in the list of diseases which cannot be produced by any pollution of the atmosphere, unless there be present a specific contagious miasm secreted from the human body! Is Dr. Bryce in practice as a physician? We can hardly suppose that any man who has been conversant with hospital or private practice, could enunciate such doctrines. Dr. Bryce is more inclined to sophistry than candour. He dwells on the nurses that were affected in the hospitals; but makes no allusion to the immunity of the medical attendants. One observation convinces us that Dr. Bryce is either a superficial observer or a prejudiced thinker.

"Notwithstanding, says he, the great stress that some writers have placed on the influence which fear exerts in contributing to the propagation of the disease, my own experience, and it has been considerable, demonstrates to me in innumerable instances, that a more than Moslem indifference deadened a becoming sense of danger in this portion of the community. Prejudice and ignorance, fostered and strengthened by infamous publications, closed the minds of its victims to the remonstrances of medical men. It was vain to warn of danger, persons,

'———— quos ille timorum
Maximus haud urget, lethi metus.'"

There cannot be a doubt that Dr. Bryce includes this journal in the proscribed list of "infamous publications," because we sought to dispel the alarm of contagion, and procure for the sick those attentions which the doctrine of contagion was so well calculated to prevent. Conscious, not only of our own good intentions, but of the truth and justice of our cause, we smile with contempt on the uncharitable insinuations, to say the least of them, contained in the above passage, referring to the experience of the profession, the question, whether or not FEAR is a strong predisposing, exciting, nay, a directly occasional cause of cholera, and the whole class of gastro-intestinal disorders.

We shall take leave of Dr. Bryce, by alluding to the sovereign contempt in which he appears to hold the *contingency* of contagion in cholera, or in other diseases. Yet, in page 293, he speaks of "the cholera miasm, whose activity, *if not existence even*, depends on an impure state of the air."

Again, he repeatedly alludes to the certain prevention of cholera by seclusion, or in other words, quarantine restrictions—and yet, in the page above quoted, he tells us that—"the *only* limit, therefore, to the diffusion of the contagious miasm of cholera, is the circulation of pure air." This is the identical doctrine which we have all along maintained—it is the doctrine of contingent contagion—it is simple and practicable—whereas, the clumsy hypothesis of "a *compound* miasm which acts as the essential *vehicle* of the specific *contagion* of cholera," is as unintelligible as it is unphilosophical. We congratulate the contagionists on the accession of such a hair-splitting ally as they have now found in the person of Dr. Bryce.

### II. MR. WATT ON THE ORIGIN AND PROPAGATION OF CHOLERA IN GLASGOW.

THE distinguished Professor of Medical Jurisprudence, in Glasgow, has published a short paper in the same number of our contemporary on the Clyde, in which a very different view of the subject is taken. "The first case of cholera that occurred in the West of Scotland, was a boy named M'Millan, who was taken ill at church in Kirkintilloch, on Sunday, the 22d of January, and every pains was taken, no stone was left unturned to discover the contagious source in this case. Numerous were the conjectures, and not a few the positive assertions, of the exclusive and unlimited contagionists, *who almost en-*

*tirely monopolized the field, and regarded as fools all who dared to doubt their doctrine, and believe that cholera might arise in a spontaneous manner, but Dr. Lawrie has proved, as far as negative proofs can prove, that no source of contagion existed, from which, by any possibility, the boy M'Millan could have received the disease.* Dr. Lawrie's pamphlet also affords ample evidence, to prove that contagion, as an agent, had nothing to do with the propagation of the cholera, and Mr. J. Watt was also of decidedly the same opinion. The disease appeared in a particular district (Hill-head) of the village, and was confined to that district for thirty-one days, and although individuals actually labouring under the disease, were removed into houses in the centre of the village, and died there, not one of the inmates or attendants, in these houses, or neighbours who were in communication with the patients, or with them were affected."

When the disease broke out at Kirkintilloch, the Glasgow Board, a thorough scion from the first, or ultra-contagion Board of London, stationed agents at all the entries into the town to stop the *march* of the malady from Jessore to the Gorbals. The canals and rivers were all blocked up. The disease, however, broke out—but in none of the primary cases was there the least shadow of communication with "infected districts." The author goes on to detail a number of the first cases in Glasgow, proving the scattered, isolated, and spontaneous origin of the disease.

"It follows then from the history of these cases, that it is impossible to demonstrate the agency of a contagious or infectious principle in the production of the malignant cholera in Glasgow. There are not even the slightest grounds for supposing the existence of such agency. The disease arose spontaneously, that is, under the influence of epidemic causes."

But we shall pursue the subject no farther in this place. Dr. Bryce himself acknowledges, that a polluted atmosphere is essential to the propagation of cholera contagion (the only contagion, by the way, which requires this essential) and, if this be the case, it will require a cleverer man than the Doctor to prove, which of the two essential agents is the true cause of the disease.

---

### XXXVI.

### Proximate Cause of Cholera.

Our readers are aware that we have characterized cholera as a serous hæmorrhage from the mucous membrane of the stomach and bowels, looking on the cramps, coldness, and other phenomena of collapse as consequent on the hæmorrhage. This idea is adopted by Dr. Lawrie, of the Albion Street Cholera Hospital in Glasgow, as will be seen in the following extract.

"*Proximate Cause.* In a report of this kind speculative opinions are inadmissible, but I think it fair to state a few facts which appear to me to bear on this part of the subject. Of all the patients admitted into Hospital there has not been one in whom the disease did not begin in the bowels and stomach. In the vast majority purging was either premonitory, or the first symptom, Some there were who said that nausea and vomiting first annoyed them, but all had purging early in the disease. In some there was no vomiting, in others no cramps, in a few the pulse, capillary circulation and respiration were good, but all, to a man, had a discharge of characteristic watery fluid from the bowels. So much am I convinced of this, that I now consider watery purging essential to cholera, and have no hesitation, in my own practice, in pronouncing any disease not cholera in which this symptom is absent. The purging is usually unattended by griping pains.

In every case which I have had an opportunity of watching from its commencement, the failure of the pulse, capillary circulation, and other symptoms of collapse, have been in marked proportion, to the amount of fluid discharged, the rapidity of its discharge, and its approach to water in colour, consistence, and smell.

*I know no symptom by which a common diarrhœa or disordered stomach and bowels can be distinguished from the early stage of cholera.* I visited a young woman, at three in the morning, who had discharged the contents of her stomach and bowels, she said, in consequence of eating too much pork at supper. Her voice, pulse, skin, and respiration were natural, and she said she was as well as ever she was in her life. Who could have said this is cholera? To guard against accidents I gave an emetic followed by an opiate with brandy, and left her, she said, quite well. Three hours after, during which interval the discharges had continued, I found her voiceless and pulseless. Here the discharges are the primary disease, the asphyxia and cramps are secondary, and this my own experience would lead me to say, is universally true of cholera.

If the above be facts, what need is there to search for the proximate cause of cholera, in the ganglionic system, brain, heart, or lungs; the evidence of our senses points to the mucous membrane of the digestive system. I will not stop to enquire what part of this membrane is affected, but merely state that I believe that in this, as in every acute disease, the nerves of the injured organ sustain the first impression.

Are the following legitimate deductions from the facts stated? A morbid poison enters the system, impresses the nerves of the mucous membrane of the stomach and bowels, and forces the capillaries to discharge the serous portion of the blood and the salts which it holds in solution; the pulse fails because there is too little blood in the system; the lungs, because the thickened blood no longer finds its way into the capillaries of their air-cells; the skin becomes cold because respiration and capillary circulation are imperfect; and secretion ceases because the glands have no longer the 'materiel' on which to act. In a word, the serous discharges explain the whole subsequent train of symptoms, and the severity of the disease will, as I have already said, be in proportion to their amount, rapidity, and purity."

---

### XXXVII.

### Observations on the Powers and Effects of Cold, as a Cause of Disease, &c. &c. By Dr. J. Clendinning.

In our valued and oldest contemporary, the Medical and Physical Journal, Dr. Clendinning has published a monograph on that popular and real cause of multiplied evils—cold. Every medical practitioner is aware that nine-tenths of the diseases presented to his observation, are attributed by the sufferers to catching—"cold"—and there must be some, nay, there must be much foundation in truth for so general a persuasion. From the cold wash of our first nurse, to the heats and chills of our juvenile sports, and unavoidable exertions of our riper years, the effects of cold, or rather of atmospherical transitions, thermometrical and hygrometrical, are daily conspicuous to the common as well as to the medical observer. It has been recorded by Dr. Bateman that, during the Winter of 1814, which was very severe, the number of patients at the Cary Street Dispensary exceeded by 700 the ordinary average in other years! Dr. Heberden also records the fact that, in January, 1795, the whole mortality of London, was *double* that of the succeeding January. We question if the redoubted cholera of January, 1832, has produced such a tremendous change in the balance of our final accounts with grim Death.

The author of this monograph, a gentleman of highly cultivated mind and excellent education, general as well as professional, has arranged his observations under six heads—definition of terms—morbific properties of cold—diseases of cold—principal forms of morbific cold—circumstances most favourable to the morbific action of cold—and lastly, the means of preventing diseases of cold. These subjects, so clearly arranged, are scientifically treated, and ingeniously illustrated by Dr. Clendinning. We regret that, from the terse and didactic manner in which the talented author discusses each point, we are quite unable to attempt an analysis of the paper. We are therefore induced to insulate one or two propositions, and give them in the writer's own language, as they will prove interesting in themselves, and afford a fair specimen of the whole performance, which we urgently recommend to the attention of our readers.

"FORMS OF COLD MOST DANGEROUS.

The principal and most active forms of morbific cold met with in practical life are three: *moist atmospheres, damp clothing*, and *currents of air*.

Moisture is not of itself injurious to health. Moist warm atmospheres are indifferent to the vigorous, and they are generally favorable to the weakly. *Wet* summers are healthful in this country, provided they are not cold; the summer of 1797 furnishes a very striking proof of this truth. 'From the middle of May it was,' says HEBERDEN, 'one of the wettest ever remembered, it was nevertheless in every respect a healthy year.' Not so, however, *wet cold* seasons. BATEMAN assures us 'that the succession of rains to heat' (i. e. of a cool or cold moisture to warmth,) 'is amongst the most active causes of disease of the chest and abdomen,' which are the most destructive complaints in this metropolis. 'A foggy atmosphere,' he again observes, 'acts much more injuriously than a clear (i. e. comparatively dry) one of equal cold. Indeed there is,' he assures us, 'no condition of the air so invariably pernicious, so chilling and oppressive to the organs of respiration, as that frequent combination of frost with fog in the metropolis.' Of the truth of the preceding observations of that judicious physician (Bateman,) I have had frequent experience amongst the poor inhabitants of Westminster for the last three years, during which I have had considerable opportunities of watching the operation of weather and season.

The danger of inhabiting or sleeping in *damp apartments*, is proved by examples of daily occurrence in private life. The superior morbific activity of a *damp atmosphere* depends on its superior conductive power. A humid air absorbs free caloric with much greater avidity and rapidity than a dry.

*Damp clothing* is another active and dangerous form of cold. The mischievous energy of wet clothes is so well known, as to require no illustration. The great capacity of evaporating water for the matter of heat, is the cause. The frigorific power of damp clothing may be conceived from this consideration: that the only protection or antagonist influence that man requires to enable him to defy the summer fires of Sahara or South Carolina, is the power of cutaneous exhalation. Although inhaling and immersed for some time in an atmosphere exceeding very far the temperature of boiling water, the bakers' girls were found, by Reaumur, to have pertinaciously retained their normal heat. After twelve or fifteen minutes' immersion in an atmosphere many degrees above 212°, BLAGDEN, BANKES, DOBSON, and other experimenters, found their thermometrical heat little differing from that of ordinary health. Such is the frigorific power of perspiration, or, in other words, of evaporation from the surface.

But *currents of air*, perhaps, of all causes of diseases of cold, are the most active and extensively mischievous. *Damp clothes* may be avoided; *foggy atmospheres*, and extremely *humid cold winds*, are unknown in many seasons and climates: but *currents of air* must be encountered. The atmosphere is constantly in a state of agitation; its intestine and progressive motions, while, on the one hand, they promote our well-being by ventilation, endanger, on the other hand, our health and our existence by their refrigerant operation. The destructive power of exposure to cold winds without adequate protection, is strikingly illustrated by the narrative published by Dr. CURRIE, in the Philosophical Transactions for 1792. 'Of several individuals that clung to the wreck, two sat on the only part that was not submerged: of the others all were constantly immersed in the sea, most up to the shoulders; three only perished, two of whom were generally out of the sea, but frequently overwhelmed by the surge, and at other times exposed to heavy showers of sleet and snow, and to a high and piercing wind.' Of these two, one died, after four hours' exposure; the second died three hours later, 'although a strong healthy man of twenty-eight, a native of Scotland, in the flower of life, early inured to cold and hardship, and very vigorous both in mind and body." The third that perished had been a weakly man. The remaining eleven who had been more or less completely *submerged*, were taken from the wreck next day, after twenty-three hours' exposure, and recovered. The person amongst the whole who seemed to have suffered least was a negro: of the other survivors, several were by no means strong men, most of them had been inured to the warm climate of Carolina.' In the case of the two first that perished the morbific power of the 'high piercing wind' was aided no doubt very powerfully by evaporation. In Dr. Currie's account of his experiments on the cold bath, we have the following interesting illustration of the superior refrigerant power of wind or air in motion. After continuing in the water fifteen minutes, the subject of some of his trials exhibited 'little or no diminution of his heat in rising into the air in a perfect calm, though during a frost; while the like exposure in a second trial, under similar circumstances, but with a north-east wind blowing sharply, produced a rapid diminution (of animal heat,) though the air was many degrees warmer' than in the preceding experiment.

I have above cited several examples of even death instantaneously produced by the chilling influence of a piercing north wind. Every valetudinarian is aware of the inconvenience and even danger of exposure to blasts from chinks and other apertures in rooms otherwise close.

*Prevention of Diseases of Cold.*

The remarks I have to make in this section will come under the head of *Clothing*, *Exercise*, *Internal Heat*, or stimulating ingesta, and *Diaphoretic Means*, as hot diluents, bed-heat, &c.

Every considerable augmentation of refrigerant influence requires, on the part of the subject exposed, proportionate precautionary means for the protection of health: these preventive measures must consist either of increased clothing or of the use of means capable of compensating for defect of personal coverings, by diminution of intrinsic organic susceptibility. The class of preventive means last alluded to will be by and by considered, under the heads of exercise and stimulating ingesta: at present I shall confine myself to the question of clothing.

Transition from a tranquil into an agitated or progressive atmosphere, as from indoors into the open air, from the inside of a stage-coach to the outside, &c., is accompanied with a great increase of the refrigerant power, which the frame has to encounter, and will, in many instances, above all if moisture be present, require additional protective covering. When the exposure is but short, or the weather is fine, or the constitution vigorous, and reactive energy therefore ample, such precaution, no doubt, will generally be quite unnecessary: yet those compensative conditions must be often wanting in a greater or less degree, and exposure, therefore, not provided against by appropriate internal or external means, will often prove hazardous, and sometimes fatal. In how many cases has phthisis been traced to an indiscretion of the sort now alluded to; to a journey on the top of a stage-coach in bad weather, or by night with insufficient clothing, &c. In how many instances have youth, and accomplishment, and loveliness, fallen victims to the noxious influence of cool, perhaps damp out-of-doors atmospheres in passing from one rout to another, or in returning from scenes of splendid riot to domestic solitude and repose.

In passing from a state of activity or exertion to one of relative quietude, precautions are often required for security: such transitions occur when horse or foot exercise is exchanged for riding in an open carriage, or gestation on the water, and obviously demand the like precautions with transitions from walking, running, &c., to sitting, lying down, &c. But of all conditions that require provident measures, that of sleep stands most in need of them. In that condition the calorific function is less excited, less exposed to incidental stimulation from physical agents, or moral impulses, or muscular exertion, than in any other. Less heat is evolved; the body is much more readily chilled, the cutaneous functions more easily disturbed, and every derangement of internal parts, producible by frigorific impressions on the skin is more promptly effected. In the state of sleep, it is therefore, if ever, necessary to guard against exposure to cool moisture, currents of cool air, and every other cause of diseases of cold. All this is very plain, and is generally known, and requires no further notice. Before quitting this topic, however, I would briefly enter my protest against the absurd and mischievous extreme to which many, perhaps most people, carry the use of woollen and other night clothing. It is common for females, in particular, who seldom, amongst the richer classes at least, know the comfort, the real luxury of woollen or chamois coverings for the shoulders, chest, feet, &c., and who wear below the knee, on the arms, upper part of the chest, neck, or head, either slight or no covering, to retire to sleep on beds of feathers, under half a dozen or more folds of one material or another, mostly woollen, and this in soft, nay even in summer weather, and with every avenue for fresh air, every door and window, closed, and bed-curtains perhaps drawn closely all around: from such violent transitions what wonder if inconvenience result! The sleep is more or less disturbed by dreams and feverish uneasiness; the strength is not properly recruited, and the sleeper awakes unnerved, languid, indolent, often hot or chilly, generally anorectic. Under such circumstances, a susceptibility of inconvenience and injury from cold, above the average, may reasonably be looked for, and will, I believe, seldom fail, if occasion offer, to shew itself. Nor is the relaxation attending long immersion in warm air the only disadvantage in such cases; for there is obviously the further one of long-continued respiration of an impure atmosphere to be taken into the account: a disadvantage of no trifling importance in the cases of such as retire early to small rooms, and emerge into daylight after protracted slumbers.

Another point in which many fail, is the adaptation of clothing to season, weather, &c. No one questions the propriety of such adaptation in the abstract; but the number of those that commit the grossest errors on this subject in practice is enormous. What can be more obvious than the temerity of wearing the same kind and quantity of clothing in the heats of summer and frosts of winter; yet there are not wanting in the very first rank of the medical profession persons chargeable with such imprudence. I recollect very well the substance of an argument I once had with a fellow-traveller, an Austrian cadet, on his way through mountains, in mid-winter, *en voiture*, from Vienna to Laybach. He obviously suffered inconvenience from want of warmer clothing, yet would not admit the propriety of adding even a flannel vest to his wardrobe. He considered it, he told me, 'militärisch,' soldier-like, to dispense with woollen under-coverings. A like answer would no doubt be given by many defaulters on this side of the water. Ladies would hold it to be *feminine*, gentlemen, *manly*, &c., to dispense with the extra under-clothing proper for winter and cold weather. But indolence, temerity, and fine breeding, are bad protectives against inclement seasons.

*Exercise.* An observant individual can seldom fail to know when, from universal weakness or incidental exposure, he is in danger from external cold; and a provident man will easily, in general, foresee future exposure. When actually exposed, the great prophylactic is muscular exertion, and, if possible, locomotive exercise. Ritter's advice is excellent, when he recommends that we should counteract the chilling influence of a draught or of a damp atmosphere, to which we are constrained to expose ourselves, by proportionably increased exercise in order that we may be enabled to compensate for the augmented expenditure of caloric by an increased evolution of it. The calorific power of general muscular exertion is such that, but for the antagonist frigorific power of cutaneous exhalation and vaporization, there can be no doubt that even moderate exercise would be incompatible with health, and that violent locomotive exertion would, in comparatively tranquil atmospheres at least, prove destructive of life. It is so great, that, duly persevered in, and aided by clothing sufficient to protect the skin and extremities from the immediate contact of an intensely cold air, it has been, on innumerable occasions, found sufficient to bear man harmless through the most formidable trials, as the narratives of Parry, Franklin, Scoresby, and many others, abundantly testify.

Respecting the use of *hot drinks* and *aliments at once nutritive and stimulant*, before and during exposure, little need be said. All experience is in their favor; every traveller on our stage-coaches knows the protecting power of warm tea and coffee, punch, &c.; there is even unequivocal experimental proof of the power of stimulant drinks to sustain the animal temperature under exposure. During my experiments on the cold bath, I found, in some trials with warm drinks and wine, (taken before immersion,) the sensation of cold little less lively indeed, and the access of shivering little retarded; but the pulse and heat under the tongue were much less reduced by the cold than in other trials made without such preparation. As a preparative, however, for protracted exposure to cold, &c., pure vinous liquors are obviously unsuitable means: the excitement they produce is transitory, and is followed by dangerous depression of calorific power: and their repeated and free use is, amongst other objections, liable to this, that it favors that somnolency which is one of the most perilous effects of cold. I have little doubt that the protective power of punch, negus, &c. is more owing to the hot water than to the pungent spirit.

The fourth division comprises the means of cutting short incipient diseases of cold. On the supervention of chilliness and other symptoms, effects of recent exposure to cold, such as slight headach, horripilation, dejection of spirits, hoarseness, slight sore throat, coryza, lachrymation, cold feet, anorexia, lumbar pains, &c., we should have immediate recourse to the shelter of a warm bed; all solid aliment should be withheld; our only ingesta should be warm diaphoretic drinks. Diluted vinous liquors taken warm, such as weak hot punch or negus, are often excellent diaphoretics in such cases. But, in general, the alcoholic ingredients may be safely dispensed with, and when the excitement is considerable and headach is present, it cannot, without rashness, be recommended. The preceding measures are usually sufficient, if early enough employed, to cut short incipient derangements from cold. Where irritation is considerable, which is indicated by flying pains in the back and limbs, lively sense of cold, smart shivering, &c., opiates had better be employed in addition to the means already mentioned: for this purpose Ritter highly extols a combination of opium and camphor, two or four grains of the latter with from the eighth to a fourth part of a grain of the former every second hour, until the horrors, headach, pains, &c. shall have vanished or greatly declined. I have no doubt of the utility of such a combination; but pure laudanum or opium combined with warm diluents will probably be found fully as efficient. Dover's powder is also an excellent remedy. Another remedy, at once efficient and agreeable, is the common effervescing draught, containing half a scruple of nitre, a drachm (more or less) of the compound tincture of camphor, and in some cases half a drachm or more of nitrous æther, and as much of Hippo wine, to be repeated every third, fourth, or sixth hour. Where the feeling of cold, as evidenced by horripilation, rigors, &c. is lively, warm bathing, local or general, followed up by some of the remedies just proposed, is very proper.

Prevention of disease is better than cure: it implies a more masterly degree of skill and power in the prescriber, and a smaller expense of care and vital power on the part of the sick. In practical medicine the first indication in dignity as well as time, is prevention: in other words, the avoidance or counteraction, as far as possible, of morbific agencies; and when illness arrives, the employment, without loss of time, of the means best calculated to disperse the earlier groups of organic preternatural conditions or symptoms, and thus, by anticipation, get rid of the complications and difficulties so soon superinduced and accumulated upon primary simple and tractable derangements by the influence of sympathy and habit: with these views, I have thought it advisable to append to my observations on the morbid effects of cold, remarks on the circumstances that most favor the action of morbific cold, on the means best calculated to neutralize its agency, and on the remedies that should be employed after injurious exposure to prevent the establishment of any nosological effect or regular disease of cold: on the plan, as on the execution, it is the reader's province to decide."

The whole monograph which would have well deserved a place in the Cyclopædia of Practical Medicine, or in Dr. Copland's Dictionary, contains the most convincing proofs of the author's learning, talents, and discrimination.

---

XXXVIII.

### On the Employment of Tepid Baths, combined with Cold Affusion in Cerebral Affections.

### First Series.

*Case* 1. Count B., of studious habits, experienced, very soon after having been engaged in intense thought on a subject which had quite absorbed his attention, an indescribable weariness and even exhaustion of mind, so that he was unable not only to continue his more reflective pursuits, but even to read any amusing book. Though he slept well, his mind did not recover itself; he applied to M. Recamier, who ordered tepid baths, with cool affusion on the head at the same moment. He speedily got well.

*Case* 2. Duke L. member of the Chamber of Peers, had suffered much fatigue from unremitting application to parliamentary offices; his friends feared that he would be obliged to withdraw himself for some time from his official duties. The baths with affusions were ordered, and his health became almost at once restored and vigorous.

*Case* 3. A member of the Chamber of Deputies had been long subject to headaches; he became one of the ministers of state; his headaches increased tenfold; he was advised to take the baths, as in the former cases, very regularly, and so soothing and invigorating were their effects, that he himself ascribed his ability to remain in office to them, in a great measure.

### Second Series.

*Case* 4. Mad. D., æt. 34, had suffered for several years from almost constant headaches, which she ascribed to anxiety of mind; the sight had also become painfully sensitive. Counter-irritants had been used with no benefit; but the bath with cool affusions speedily brought relief.

*Case* 5. Abbé G., aged 65, had been first afflicted with headaches, while pursuing his theological studies; their severity had gradually increased in consequence of great anxiety and distress; he had thus suffered from these, more or less, for forty years; they were always exasperated by study; the sight and hearing had become excessively sensitive to their stimuli; and even the sense of touch had acquired a morbid acuteness; he was subject to spasms of the face and arms, and to general muscular uneasiness. Baths with the affusions were ordered: and having taken about twenty, he began to experience much relief; he continued their use for a long time, and was at length almost completely made well.

THIRD SERIES.

*Case* 6. A lady had been in the habit of taking anodynes every night, in consequence of a painful disease. Her mental faculties had become much impaired, and when M. Recamier visited her, she was in a semi-narcotic state. She was treated with the cold baths, with much temporary benefit, both to her bodily and mental health.

*Case* 7. A man, aged 34, was brought to the hospital in the following state; he was quite insensible, and could not be roused to answer questions, or to take notice of any thing; the pupils were dilated; the breathing and pulse natural; and he could move his limbs freely. There was no reason to believe that he was intoxicated, as he had spent the preceding night with his family, who stated that he had not drunk any spirits, &c. &c. By the use of the tepid bath, with cold affusions to the head at the same time, he was immediately well. M. Recamier designates such a case "nervous stupor," or "spontaneous narcotism."

FOURTH SERIES.

*Case* 8. A young lady had been much affected with the loss of an intimate friend; a headache, at first slight, and afterwards very severe, came on; leeches were applied behind the ears, but instead of relieving, they aggravated all the distress; the pulse became quickened, and the system feverish; the vision and hearing morbidly acute; and the whole surface of the skin, and also the organs of smell and of taste so exquisitely tender that she complained of the coppery savour of the water, which was sprinkled on her head; the vessel in which the water was held, was of copper. On the first day of treatment, by Recamier, she had three baths with affusion; three also on the second; and two on the following three days; and then only one daily; their temperature varied from 73° to 77° F. that of the affusions was about 40° F. their duration was from 15 to 18 minutes. Good effects were speedily obvious, and a decided amendment appeared on the ninth day, from which time she rapidly recovered.

FIFTH SERIES.

*Case* 9. A young female was thrown into deep affliction by the death of her husband; she became quite lethargic, and continued so for 11 days, during which time she shewed no sign of consciousness, and appeared to be quite insensible to all outward impressions; the pulse was variable, but always quickened; food was introduced by the stomach-pump, but it was instantly rejected by vomiting; she had been blistered and well physicked, with no advantage. Dr. Recamier ordered a bath of 76° Fahr. combined with affusion of cooler water on the head. On being taken out of the bath, she appeared to have awoke from a deep sleep, and in some degree recognised her attendants, but took no notice of her late bereavement. The baths were continued for several days, till the catamenia appeared and then they were discontinued; during the short interval of their presence the malady returned, but under a different form; instead of the uninterrupted lethargy, there was a double quotidian febrile paroxysm of a few hours' duration, during which the skin was so tender, that if merely touched convulsive movements of the limbs were induced. Subsequently cataleptic symptoms took the place of the preceding; and afterwards cardialgia and incessant vomiting were the most prominent distress. The state of the patient becoming thus more and more afflicted, recourse was again had to the baths, which were employed for about 15 or 20 minutes each time, and the cool affusion to the head for the last three or four minutes. This treatment was continued for a fortnight, and her health was then much benefitted. With the exception of a slight relapse, in consequence of the intermission of the bathing for a few days, the lady was eventually so restored to health, that the French reporter states that she was soon able to eat a "bifteack" of reasonable dimensions!

SIXTH SERIES.

*Case* 10. A young man, aged 22, of a healthy sanguineous temperament, had been for four years subject to monthly returns of a nervous paroxysm, whose most obvious symptoms were the following; ennui, melancholy, wish to weep, but he could not; excessive irascibility and obstinacy of temper; flatulency with borborygmi, and sense of constriction in the throat; then aberration of mind, piteous moanings, severe dyspnœa, and violent muscular movements. He was advised to take, for several days preceding the monthly paroxysm, several baths with affusions; and under this treatment alone, his health was re-established.

OBSERVATIONS ON THE PRECEDING CASES.

The *first series* illustrates well the "malaise" which follows excessive mental application. It is not quite easy to designate this state of indisposition; it is not altogether a disease, and yet the patient is assuredly not in health; he is between the two, but nearer to the former; he has, as it were, come within the influence of the poison tree, although the air is not yet so venomous as to be mortal: or he may be compared to a man who is dizzy on the edge of a precipice, who has not yet lost his balance, but a moment longer and he falls head-long. [Our readers will call to mind our remarks on this subject in the introductory chapter "On the Wear and Tear of Life," in the "Change of Air."—ED.]

In the *second series* we observe the symptoms of the malady somewhat aggravated; for in addition to general cerebral uneasiness, and to an inaptitude for mental exertion, the external senses have become disturbed; thus, the eye in one, the hearing in another, and in the third all the organs of perception may become the seat and cause of distress to the patient; this diversity can be accounted for only by a reference to an hereditary, or an accidental sensitiveness of the particular organ affected.

When the excitement of the brain is carried to a still greater extent, or when the constitution of a patient is such that the nervous energy is soon exhausted, a state of perfect stupor may be induced, as we find in the cases of the third series. The blindness from a dazzling light is an apt illustration of this law of life, and just as in this case, the eye is paralyzed for the time, so may the brain be overpowered by any excessive or protracted mental stimulus. But the degree of excitement does not necessarily nor uniformly cause a mere "spontaneous narcotism;" for not unfrequently the ganglionic system sympathises with the fatigued cerebro-spinal system; and hence we have the long and varied train of nervous affections of the functions of organic life super-added, viz. disorders of the respiration, circulation, digestion, and secretion; hence the cardialgia, and colic, cough, dyspnœa, palpitation, remittent febrile paroxysm, and so forth, and it is obvious that one treatment is applicable to all, namely, the treatment of the original complaint, which is the "fons totius mali." Case 9, is an example of the foregoing remark. It is of great importance to remember, that whenever the functions of the ganglionic nerves, namely, those of organic life, are once drawn into the morbid association, the obstinacy of the malady is increased ten-fold; for it is a law of living bodies, that in proportion as a part is more or less easily affected with disease, so it may be more or less speedily recovered from it. Hence the necessity of steadfastly persevering in the use of the treament above recommended for several months, at least in obdurate and protracted cases. Lastly, we advert to the good effects of the same treatment in cases of simple hysteria, or of neurosis of any particular organ, as of the lungs, by which the process of sanguification is disturbed; or of the heart, giving rise to most distressing palpitations; or of the alimentary canal, occasioning dyspepsia, hypochondriasis, &c. &c. Medical men are sometimes perplexed, when, after the violence of a fever has been subdued by active antiphlogistic means, a state of almost constant delirium, and of extreme sensitiveness of the eye and of the ear, (so that no light or noise can be tolerated) supervene: the patient is also at the same time exceedingly irascible and fretful;—all depletory means aggravate these symptoms, and no treatment is so soothing as by the use of the tepid baths with cold affusion once or twice a day.—*Revue Médicale.*

*Remarks.* We can add our most unqualified testimony in favour of the truth of the foregoing details; and, indeed, the practice of every medical man can no doubt do the same. The catalogue of cases, or of the series of cases may be much enlarged; but we shall only allude to one in addition; namely, the state of health predisposing and preliminary to an attack of hydrocephalus. By keeping the head of the child constantly wet with cold water, night and day, the disease may very often be warded off.—ED.

---

### XXXIX.

### VAGINAL CYSTOCELE.

THE bladder may be protruded as a hernial tumor in three different places; into the groin, at the crural arch—and into the perinæum (in men); and the vagina and perinæum in women. It is obvious that a perineal cystocele, in females, can be only an aggravated case of vaginal cystocele, in the same manner as a scrotal hernia is only an aggravated bubonocele.

Mad. M. aged 40, was mother of four children, all of whom had been born without difficulty; her last confinement happened four years ago, soon after which time she began to experience dysuria, pains in the renal region, and a feeling of severe dragging at the hypogastrium. Various opinions were formed of her case, some considering it as calculus, or chronic cystitis—others as disease of the womb, &c. &c. She experienced no relief from any treatment. After 3½ years of suffering she applied to Dr. Rognetta; her symptoms at the time were—an almost constant desire to void urine, which passed only in drops, and with dreadful straining—frequent dragging pains in the epigastrium, and extending over all the abdomen—nausea and retching—cold sweats, and general decay of strength. At first Dr. R. suspected cancer of the womb; but he found that, upon introducing his finger into the vagina, although there was a fleshy tumor which obstructed the entrance, and which he took to be the uterus, he could gradually insert it deeper, till he detected the os tincæ high up, and quite healthy. Upon ocular examination, the tumor was as large as the fist, of a red color, smooth, and glistening; it fluctuated on pressure, and a desire to make urine was thus induced. A female catheter could not be passed, in consequence of the displacement of the urethra, whose course was now from above downwards; a male catheter was introduced, with the convexity turned towards the abdomen, and a large quantity of water drawn off; immediately the vaginal tumor diminished, and the patient was in comparative ease; her distress returned when the water re-accumulated. All ordinary pessaries failed in supporting the protrusion; but, fortunately, Dr. R. succeeded by using a gum-elastic bottle, and rolling it into a cylindrical form; it was secured by tapes to the "ceinture" of the patient.

*Remarks.* In the history of such a case, it is of importance to attend to the symptoms of distress in the epigastrium, and feeling of painful dragging from the stomach downwards; as they probably arise from a portion of gut, or of omentum, being drawn down with the displaced bladder, and thus occasioning a real *dragging* of the parts. When, therefore, a patient complains of such a malady, our attention should be awakened to the possibility of some hernial protrusion. Baron Larrey mentions the following very interesting case in illustration.

On the evening after undergoing lithotomy, a patient had all the symptoms of a strangulated rupture. The Baron examined the wound, and found a swelling, which protruded outwardly between its lips, and which he ascertained to be formed by a noose of intestine, or of omentum, having forced a portion of the bladder before it; he immediately reduced it, and retained it in place by using an empty bladder, which he introduced to the bottom of the wound, and which he then distended with air. The patient did well.—*Ibid.*

---

### XL.

### Case of Anencephalism.

A poor woman, aged 30, mother of two children, was delivered of a third in April, 1830; it was male and anencephalic, and yet it lived for 32 hours. Since that period, the woman had dreaded being again pregnant, as "she was certain (she said) that she should bear nothing but monsters." On 4th July of next year, she was delivered of a female child; and this also was anencephalic. It lived for 20 hours. The respirations were feeble and distressing, and, soon after birth, the face and body became of a blue colour, which deepened more and more to a blackish hue. Its size was that of a full-grown and healthy child. The shape of the head was most extraordinary; the level of its vertex was on a line with the root of the nose—its dimensions were much contracted, as well from side to side as from before backwards; the face was fully formed, but the forehead and eyebrows were wanting: the eyes were startingly projecting, in consequence of the deficiency of the upper part of its orbit, and were situated at the most elevated points of the head; the inclination of the face was sloping backwards, so that the facial angle was much smaller than usual. On a careful examination after death, the whole of the vault of the skull was found wanting, and the open cavity was bounded by a rim, formed by the frontal, parietal, and occipital bones; the basis of the cranium was much contracted in size in all its dimensions; the posterior wall was inclined forwards, so that the os occipitis appeared to rise vertically, in a line with the margin of the foramen magnum, and hence there were, in reality, no occipital fossæ; the anterior fossæ were scarcely distinguishable; there was no trace of the cribriform plate of the ethmoid bone; the os frontis consisted of two small pieces, joined to each other in the median line; and each piece consisted of two laminæ, one of which represented the orbital plate—the other the globular portion; the foramen opticum of the sphenoid was awanting; the petrous process of the temporal was fully formed, while the squamous plate was very imperfect; the parietal bones, in shape and appearance, were like a narrow band or rim along the upper edge of the temporal bones; the basilar process was placed vertically. All the vertebræ were perfect. The scalp was covered with long hair, and extended somewhat beyond the bony margin of the cranium, and then became suddenly continuous with a red-coloured, thin membrane, which supplied the place of the vertex; it adhered to the subjacent parts, and had much the appearance of a cicatrix. On dissecting this off, a soft tumour, of the size of a walnut, and of a fibro-cellular tissue, without any trace of cerebral substance, was found to occupy the central part of the cranium; it adhered strongly to the dura mater in all directions: the cerebrum, cerebellum, and tuber annulare were quite deficient; the upper end of the medulla oblongata was to be seen, and it presented the appearance of having been cut from the tuber; it had no connexion with the fibrous substance described above. The spinal marrow was also quite normal. There was no trace of the optic and olfactory nerves in the cranium: in the orbit, the former was found, but very small, flattened, and reduced to an empty neurilema—posteriorly, it was lost in the dura mater; the eyes were large, and the retina perfectly developed. There was no trace of any of the cerebral nerves within the cranium, except of the trigemini, which appeared to proceed from a plexiform ganglion, situated in the fibrous mass; and, as this substance was infiltrated with blood, the nerves were with difficulty traced. The facial and auditory nerves were not attached to the medulla oblongata, but lay loose and unattached in the cavity of the cranium; on the outside of the cranium they seemed quite healthy; the auditory organs were fully formed. The spinal nerves were all normal in structure and arrangement. The carotid arteries were very small, and terminated in the fibrous mass; the vertebral were shrivelled to mere threads. Lungs nearly healthy; the ductus arteriosus and the foramen ovale were quite open. The abdominal viscera were perfect; meconium was found in the large intestines.

*Remarks.* The above case illustrates well, that a fœtus may live in utero, attain its full growth, and even breathe after birth for some hours, although the cerebrum, cerebellum, and pons varolii are absent; and examples are on record, in which not only these, but also the entire length of the spinal cord have been deficient, and yet the rest of the body has not suffered in development. We see that respiration is independent, at least for some time, on the brain; when, however, medulla oblongata is also wanting, the children exhibit signs of life only at the moment of birth, and never breathe; the duration of life, in all anencephalic cases, appearing to be inversely proportionate to the extent of deficiency in the great centres of the nervous system. Various conjectures on the causes of anencephalism have, at different times, been proposed. It has been attributed to a very early hydrocephalus, or to an inflammation of the nervous substance and of its membranes, by M. Blandin; but where are the pretexts for such ideas? Others, as M. G. St. Hilaire, more cautious, have not advanced farther than to suppose that, at some period of fœtal life, the development of the brain was "*somehow*" suddenly arrested, while other organs were gradually unfolded to maturity; but before admitting even this supposition, let us inquire whether the traces of brain, when these do exist, at all resemble the appearance of the organ at any period of the fœtal life; for if we say that the growth was checked at some time or another, it is but fair to presume, that there should be vestiges of its "*then*" development at the time supposed. Now, passing over those cases, in which not a trace of the cerebral mass is to be found, can we reasonably compare the fibrous mass, in the above case, to the brain, as it naturally is in any period of embryotic life? Assuredly not; and, therefore, we are forced to refuse our assent to the hypothesis of simply arrested development. We should rather say that there has been a destruction or degeneration of the parts; but how this has been effected, or to what cause it is owing, remains a mystery. It may be thus seen that we admit the opinion, that the embryo contains, at the first moment of its existence, the bases or elements of all its various parts and organs; that these organs are, therefore, only developed, and not formed in succession, and that, originally, they are invariably regular and normal; and, hence, that monstrosities are "acquired" during the early stages of uterine life: (what are the grounds of such belief we know not.—ED.)

M. Serres has advanced the opinion, that the development of any part is dependent upon its supply of blood, and, therefore, on the size and number of its arteries; and that, therefore, when these are small and scanty, the part necessarily withers; but, in answer, we ask for some proof. The reverse of the position may be equally true, viz. that the decay or absence of the organ dispenses with the supply of blood. Indeed, the circumstance of the entire upper part of the cranium being deficient, is adverse, one should think, alike to every theory we have mentioned; and it appears that the complete skull is not a necessary and original elementary structure, but is formed for the purpose of protecting its contents, on which it is moulded. In one case, all the anterior and middle portions, even of the basis, were much contracted, for there was no brain to occupy them. M. Breschet, in the article "Anencephalie," *Dict. de Med.*, is in error, when he states that this monstrosity is always associated with spina bifida, for the spinal column was complete in the example we have detailed; and his generalizing assertion, that the imperfections of development are always more marked in the posterior, than in the anterior parts of the cranium, is refuted at the same time; indeed the very reverse held true, as above stated. When the whole encephalon is wanting, and the spinal marrow begins at the foramen magnum, or lower down, we find that there is no cranial cavity whatsoever; the occipital bone is generally cleft in the median line, so that the foramen is not closed behind, and the upper cervical vertebræ are imperfect and open;—here, then, we have further proofs of the osseous structure being subordinate to, and dependent upon, the soft viscera; the rudiments of the bones are probably never wanting; and the pithy observation of Hilaire is correct—" un os ne rétrograde jamais jusqu'à zéro d'existence."

We have already stated that the cerebral nerves, with the exception of the optic, were perfectly and normally developed, on the outside of the cranium. It is not correct, therefore, to say that they arise from the brain; for it appears that they are formed with the organs to which they are subservient, independently of the presence of any central nervous mass. What was singularly interesting, was the discovery of the retina being perfect, while the optic nerve was wasted, or rather did not exist at all.

We ought to have mentioned that the supra-renal glands were wanting; Meckel says that they are always so in anencephalic children.—*Ibid.*

---

### XLI.

### On Muscular Contractions, and Animal Electricity.

The change which takes place in a muscular fibre at the moment of contraction, consists in its assuming a zigzag flexuosity, and is best illustrated by the following diagram, which at the same time points out the amount of shortening.

This may be stated to be nearly one-fourth, or 0.23. The above appearance is most readily detected by submitting a very delicate muscle, as the sterno-pubic of a frog, to a microscope; and at the same time we may observe that the summits of the angles, *c, d, e,* correspond precisely to the junction of the minute nervous filament, which, when the muscle is relaxed, ran parallel to each other, and perpendicular to the muscular fibres. Admitting the correctness of this opinion, it will easily be conceived that the living muscle is really a galvanometer, and one too of extraordinary sensibility. In all cases, where muscular contractions are produced, there also exists a development of electricity. For the purpose of displaying this, let two similar platina wires be fitted to the ends of the branches of a galvanometer; let one of them be plunged into the muscles of a frog, and let the nerves of the animal be touched with the other, heated to redness; the contractions will be strong, and the deviation of the needle very sensible.

We have ascertained by experiment, that when two living animal substances are pressed together, however slightly, they acquire opposite states of electricity. It is sufficient for two insulated persons to touch hands, and then withdraw from the contact; to develop electricity sufficient to affect the electroscope of Caulomb.

The insulation of the nervous fibres is effected by the abundant fatty matter which surround them—for the discovery of this we are indebted to Vauquelin, who has shewn that it envelops each of the fibres, and does not permit the electric fluid to pass from one to the other.

It is a subject of curious investigation to try to explain how some secretions, as the milk, chyle, urine, and sweat are acid; while others, as the saliva, bile, &c. are strongly alkaline. The fluid from which they are all derived, namely, the blood, contains pure caustic soda, in sufficient quantity to impart to it manifest alkaline properties. If we seek among the facts of chemistry for an explanation of this difference between the constitution of the blood, and that of the fluids secreted from it, we may soon be convinced that the action of the voltaic pile is the only one which approaches to it. Moreover it appears possible, to imitate artificially the principal conditions of the secretions, and to separate from the blood, by means of the pile, a liquid resembling milk, and from the food itself, a material resembling chyle. We cannot quit this most interesting subject without remarking, that if muscular motion and the secretions may be regarded as owing to eletrical movements, the production of animal heat can only be suitably explained in the same manner; for it is known to electricians that the conducting wire acquires considerable heat during the action of the pile. M. de la Rive, the learned professor of chemistry at Geneva, was the first to seize the happy idea of referring the phenomena of animal heat to electric agency.

Dr. Edwards adduces some very ingenious observations in favour of the electrical theory of nervous actions; they are contained in a paper which was read before the Royal Academy of Sciences of Paris in May, 1825, on "muscular contractions produced by bringing a solid body into contact with a nerve, without a galvanic circuit." The experiments consisted in passing a solid body along an exposed nerve, in the same manner in which we pass a magnet along a bar of steel which we wish to magnetise. In doing so, our object is not to act by pressure, or mechanical irritation, but rather to touch lightly various contiguous portions of the nerve successively; the sciatic nerve of a frog, which had been pithed, was exposed from ¼ to [illegible] of an inch, and a slip of oiled silk passed under it, to bring it better into view, and to render it more tense; the nerve was then gently touched with a slender rod of silver, and immediately the muscles were thrown into contractions; the same effects were produced when rods of copper, zinc, lead, iron, gold, tin, and platina, and even of glass and horn, were used. It is proper, however, to state that iron and zinc rods caused far less vigorous movements than other metals; and that indeed the movements varied with the nature of the rod employed; but no satisfactory scale of the powers could be established. The question to determine is, whether the muscular contractions are attributable to electricity, (which we know is developed every time one body exerts a mechanical action on another,) or to some other agent, perhaps unknown hitherto to us? If it be electricity, we may presume that we shall be able to vary the energy of the contractions, according as the nerve is made a more or less perfect conductor;—in its natural condition, lying among muscles, which are excellent conductors, a great quantity of the electricity is lost and expended; but if we place under the nerve a non-conducting body, as oiled silk, the whole of the electricity will be concentrated in the nerve. This precaution is had recourse to also in galvanic experiments, when it is wished to excite muscular contractions by very small quantities of electricity, such, for instance, as are produced by the mere contact of two metals. The above fact is therefore a convincing proof that the insulation of a nerve renders it much more energetically susceptible; so that when a circuit is established by means of two different metals, very obvious movements of the muscles may be produced, where, had the nerve been left in situ, it would have been insensible to all stimuli. Now similar phenomena occur in the experiments in which the nerve is merely touched with a rod, as above detailed; and it was found that the effects were scarcely notable if the nerve had not been previously insulated. After having in vain attempted to excite contractions by touching the nerve while resting on muscle, Dr. Edwards found that they might still be induced if the oiled silk were placed beneath the exposed nerve; and he was able to cause them alternately to appear and to cease by employing at one time a non-conductor, and at other times a conductor below the nerve. It was observed that the contractions were most readily induced by a quick and light touch of the nerve with the rod. On the whole, Dr. E. concludes that the contractions in the preceding experiments were dependent on electricity.—*Dr. Edwards on Life.*

---

### XLII.

### On the Phænomena to which the Names "Endosmosis" and "Exosmosis" have been given by Dutrochet.

We may premise that the above cabalistical terms are equivalent to impulsion and expulsion, or intrusion or extrusion; they are derived from the Greek "ὠθέω" pello.

"If we take a membranous sac or cavity, as for example, the cœcum of a fowl, or the air-bladder of a fish, and having put a small quantity of fresh milk into it, and secured the mouth by a ligature, we shall find on immersing this sac in water, that in the course of a few hours it will become quite full, and eventually turgid. This turgidity is not permanent, and after a few hours more have elapsed, the sac will again be flaccid. If the sac be now opened, it will be found to contain curdled and putrid milk; if the sac be cleansed from this offensive substance, and again partially filled with milk and immersed in water, we shall find a repetition of the phænomena, but the sac will neither become so turgid nor so long remain full as in the first instance; this may be repeated several times, but with diminished effect. It does not make any sensible difference to the experiment, whether the sac be inverted or not, or whether the mucous or peritoneal coat be removed. If instead of milk some other fluid be employed, similar phenomena may be observed, but by no means in the same degree in all; in fact, very striking differences may be observed, depending on the nature of the fluids within and without the sac, as well as upon the texture through which they have to pass."

Dutrochet has invented an instrument to which he has given the name of "Endosmometer;" by means of which he discovered that different fluids possess very different powers of inducing endosmosis; it was greater in dense than in thin fluids. Solutions of gum and sugar have the property in a very marked degree; so much so, that the former caused an elevation in a column of quicksilver of 45 inches and nine lines; saline and alkaline solutions are also endosmodic; most of the acids are very feebly so; the sulphuric, however, is a striking exception, and seems rather to have an exosmodic quality. Whenever any animal fluids become putrescent, they are opposed to endosmosis; and Dutrochet discovered that liquids which contained sulphuretted-hydrogen are, like sulphuric acid, inimical to it. There is very considerable diversity in the rapidity and force of endosmosis, according to the fluid which is employed.

It may be supposed, that the phenomena of endosmosis are referable to imbibition, transudation, or capillary attraction; but if so, then the more porous the substance which is employed to close the funnel of the endosmometer is, the more energetic should be the transmission of fluid through it; but this is not the case.

"Various materials, besides the membranous parts of animals and vegetables, were employed to close the funnel of the endosmometer, such as very thin plates of sand-stone, plaster of Paris, lime-stone, burnt slate, and the biscuit of earthenware; with some of these substances, the endosmosis was carried on with considerable energy, whilst with others, it seemed totally inactive."

And again:—

"Very little, if any, endosmosis was observed to take place through plates of sand-stone, whether the most porous, or the least porous were employed, but the presence of a little ferruginous matter in one of the specimens was observed to favour it."

The septa of lime-stone, though sufficiently porous to allow the passage of fluids, and to exert a capillary attraction upon them, were found to be extremely unfriendly to endosmosis; but those of burnt slate and baked clays, though but little promising, exhibited it strongly. The following particulars of the changes which are going on during the process are very interesting.

"As respects the fluid, in which the reservoir of the endosmometer is immersed, it would seem, that there is no fluid more favourable to endosmosis, and at the same time so convenient for experiment, as pure water. The examination of this fluid, after it had been for some time employed, afforded convincing evidence of a very curious fact. Notwithstanding the copious and forcible transmission of water through the septum, occasioning in some instances an elevation of several inches in the tube, there is likewise, at the same time, a transmission of fluid in an opposite direction; thus, if a solution of muriate of soda be employed in the endosmometer, it will not be long before traces of this salt will be found in the water surrounding the reservoir of the instrument."

The applications of the principle of endosmodic operation which Dutrochet has hitherto made, relate chiefly to vegetables; he attributes the ascent of the sap to a structure situated near the junction of the root and the stalk, consisting of minute cells, in which a powerful endosmosis is exerted. The forcible ejection of the juice from the fruit of the elasterium may be due to the same cause, also the different directions which the roots and stalks of different plants take; the curious phenomena presented by the balsimina impatiens, the hædysarum girans, and mimosa, &c. &c. It is very probable that the same agencies may be actively concerned in many of the functions of animal life, both in health and disease; perhaps, for example, the enormous enuresis which attends the generation of sugar in the kidneys in diabetes, may be partly, at least, explicable in this way, as we have already seen that a solution of sugar is one of the most favourable fluids for endosmodic action.—*Dr. Hodgkin, in Notes to Dr. Edwards on Life.*

---

### XLIII.

### ANATOMY AND USES OF THE LYMPHATICS.

WEPFER states that the lymphatics of the broad ligaments of the uterus communicate with the hypogastric veins; Steno traced many to the axillary, jugular, and cavæ veins; Lobstein those of the spleen to the vena portæ. Seiler often saw mercury pass into the veins from the lymphatics, and this was almost always the case when the mesenteric glands were injected. That the lymphatics communicate also with the internal surfaces of various cavities and canals, and with the arteries, is proved by Seiler, who has injected the former from the duct of the pancreas, of the parotid, and vas deferens; by Walter, from the lactiferous tubes; by Lippi, from the gall-bladder, from the arterial system, and from the surfaces of several membranes. This latter author has published a most interesting work, entitled "Illustrazioni Fisiologiche e Patolgiche del Systema Linfatico-Chilifero mediante la scoperta di un gran numero di communicazioni de esso col venoso." He is a strenuous advocate in favour of exclusive absorption by the lymphatics, and attributes the existence of foreign matters in the veins to their communications with these vessels. It may be objected to many of the experiments of Majendie, that the induction of the peculiar effects of a poison is not a conclusive proof of its absorption.

"The strongest confirmation of this objection, is furnished by the researches of my friends, Dr. Addison and J. Morgan, respecting the operation of poisons on the living body. They have clearly proved, that the effects of poisons do not depend on the contamination of the fluids circulating through the system.

A connexion having been established between two dogs, by means of the large vessels of their necks, the blood from the one expiring under the influence of a poison, with which it had been inoculated, was transmitted with impunity into the second. To render this trial as complete as possible, a mutual interchange of blood was established, the trunk of the carotid of one dog supplying the branches of the other, and the jugular vein of one discharging itself into the heart of the other. The animal which had not been wounded with the poison, exhibited not the least indication of its influence."

Some physiologists have certainly erred in supposing that imbibition and transudation are not much modified by the vital power.

"It is well known that those animals which secrete the most deadly poisons, are by no means exempt from the fatal effects which follow the introduction of the poison into a wound. For example, the sting of the scorpion is as fatal to his own, as to any other species; yet, each individual carries his reservoir of poison about with him with perfect impunity, although it is only cut off from the rest of his system by a membranous sac. The existence of many partial dropsies may be urged as another illustration of the limitation which life sets to transudation and imbibition. It is well known that during life, the large intestines are often distended with sulphuretted hydrogen, without the neighbouring parts appearing to suffer from its proximity; yet, after death, a short time is sufficient for these tissues to acquire, to a considerable depth, the peculiar leaden hue which it imparts. The yellow tinge, which the parts in the neighbourhood of the gall-bladder commonly receive after death, has long been pointed out as a striking example of the difference between dead and living matter, with respect to imbibition."
—*Dr. Hodgkin, ibid.*

---

### XLIV.

### Transmissibility of Diseases along an Electric Wire.

The following extract is taken from the appendix to Dr. Hodgkin's translation of Dr. Edwards' work on Life. As a matter of course, we are merely reporters, not vouchers, of the occurrences. —Ed.

"Another case of ague, which had lasted four months, and obstinately resisted bark, arsenic, and other medicines, was quickly cured by a few applications of electricity. The most extraordinary circumstance remains to be mentioned. P. Smith himself had held the ball, with which he took sparks from his first patient, during the hot stage. In the same evening, he found himself unwell; but had no suspicion of the nature of his complaint, until the recurrence of the paroxysm convinced him that he had become the subject of ague. He allowed these to recur to the seventh time, before he attempted the cure by electricity, which was speedily effected, being the second case already alluded to. As he had never been the subject of ague, and had not been more than usually exposed to causes calculated to give rise to it, he felt persuaded that it had been communicated to him by electricity from his former patient.

In order to ascertain this, he was desirous of trying experiments on some persons labouring under a disease which was inflammatory, but not considered infectious; he, therefore, had one of his men vaccinated. On the seventh day, the man was placed on the insulating stool, and connected with the positive conductor; a small incision was made with a lancet in the pustule, and an incision was also made in the arm of a lad with a *new* lancet; a wire four inches long was passed through a glass tube, one end of which touched the pustule on the man's arm, and the other the incision on the boy's arm—the electrification was continued for eight minutes, when the boy was removed. His arm was daily examined, and it was found, that he was as completely vaccinated by electricity as any person could be by the usual mode. My friend afterwards endeavoured to communicate the virus to two girls, by passing the electrical fluid from the pustule on the boy's arm, who had been vaccinated by electricity, to incisions made in theirs. For three days the medical gentleman supposed it had taken effect, but, on the fourth day, all appearances of vaccination died away. These girls were, however, afterwards vaccinated in the usual way in four places, two of which died away, and the other two took but very slightly. My friend Charles Woodward afterwards repeated this experiment upon an infant, the child of one of his friends, but with this difference, that he did not allow the conducting wire to come in contact with the child's arm. The electric fluid was consequently transmitted in the form of small sparks. The disturbance which these produced, though trivial, prevented the application from being prolonged for the full time, which my friend would have wished; inflammation however succeeded, and, until the sixth day, was such as to induce the medical attendant to believe, that the vaccination had been complete; from that day, however, the pustule died away."

---

### XLV.

### CHOLERA IN SCOTLAND.

AT a numerously attended PUBLIC MEETING of the MEDICAL GENTLEMEN of Dumfries, held in the Council Chamber on Thursday, the 18th October, 1832, ARCHIBALD BLACKLOCK, Esq., Surgeon, in the Chair,—It was moved by Dr. ROSS JAMESON, seconded by Mr. CHARLES BELL, Surgeon, and carried by a majority of 11 to 1:—

That it is the imperative duty of the medical men, both resident and non-resident, who have been engaged in the treatment of the malignant or Asiatic Cholera, since it appeared in Dumfries, to declare to the public that the rise and progress of the Pestilence here, in their opinion, were quite independent of, and unconnected with, human contagion or infection; and further, that from what they have observed they do not consider it capable of being conveyed from one individual to another.

ARCHD. BLACKLOCK, Surg.

T. ROSS JAMESON, M.D. Sec.

### MEETING OF THE MEDICAL FACULTY IN DUMFRIES.

In pursuance of the terms of a Requisition, a very considerable majority of the Medical Men of Dumfries, assembled on Tuesday last, the 18th, to discuss the important question—"Whether or not Cholera has been Contagious in Dumfries." We observed besides, that several Members of the Board of Health, and others of our townsmen were present.

Upon the motion of Dr. GRIEVE, seconded by Mr. M'LAUGHLAN, surgeon, ARCHIBALD BLACKLOCK, Esq. surgeon, was called to the Chair.

The Chairman said it would be superfluous for him to make any remarks on the vast importance of the question which they had met to discuss, and, if possible, to determine—namely, Whether the disease termed Cholera, which has so fearfully prevailed, of late, in Dumfries and Maxwelltown, and hurried some hundreds of our fellow-creatures to the grave, and laid prostrate, for a time, as many more, be of a Contagious nature? They were all, he said, well acquainted with the history of that extraordinary disease, and the great variety of opinions which had been offered concerning it elsewhere. But in judging of the nature of the epidemic as it had appeared here, he thought they ought to divest themselves as much as possible of all hypotheses and opinions which they might have formed from perusing the works of the many able authors who had written on the subject of Cholera Asiatica, and humbly endeavour to decide the question before them from what they had themselves observed.

Dr. ROSS JAMESON said—I trust that no apology is necessary for the share that I have taken in calling together this meeting upon the present occasion.

The question about to come under consideration is one confessedly of vast importance, as it bears upon the happiness, the safety, and the well-being of society. It has been distinctly stated from the Chair, that the object of this meeting is not to discuss the question of contagion or of non-contagion generally, but simply to learn from the verdict of the medical men of Dumfries, here in public meeting assembled, whether or not cholera in this locality has been merely epidemic, or merely infectious, or merely contagious; or whether the disease may not have been introduced and propagated partly through all these three channels. I shall endeavour to shew that cholera, as it has prevailed in Dumfries, has been most decidedly epidemic, and also that there has as yet been no proof that the disease has been either infectious or contagious. I may here mention that the facts I am about to adduce, rest partly on the authority of the surviving relatives of the sufferers, whom I myself have examined separately, and partly on the authority of some of the medical gentlemen by whom the cases were attended. The first case was Mary Paterson. She was attacked on the 15th September last. It has been proved satisfactorily, that she never had been in any district where cholera prevailed; that she seldom left Dumfries—nay, that she rarely left her own house, except to go to church. Report said that she washed the clothes of some medical gentleman from England. The report was unfounded. It was next rumoured, that a few days previous to the attack, she had received and washed some clothes from London. A slight stretch of the imagination converted the clothes thus alleged to have been received into the garments of a cholera patient. But how stands the fact? She received the clothes, not from London, but from Airdrie House, a gentleman's seat where quarantine was rigorously observed—she received them, not a few days, but a few months previous to her illness: lastly, with a single exception, they were all new—all belonging to an individual who assuredly had never witnessed a case of cholera in her life. I have it on the authority of one of her nearest surviving relatives, that no individual who saw Mary Paterson after her seizure on the morning of the 15th, has since been attacked with cholera. In fact, if ever there was a case which seemed hedged round against the idea either of infection or contagion, it was the case of Mary Paterson. The next three *well-authenticated* cases were those of John Paton, Robert Home, and Peter Brannaghan. Not one of these individuals saw one another after the seizure of the one, and before the seizure of either of the other two. Not one of them had seen Mary Paterson; not one of them had visited any district where cholera existed. Now what is the meaning of the word epidemic? When a certain number of individuals living in the same locality—holding at the time no communication with one another—and holding no communication with any diseased district, exhibit nearly about the same time the same morbid phenomena, these individuals are said to labour under the effects of an epidemic virus. Now, in this instance, we have four individuals who lived in the same locality—who held *at this period* no communication with one another; who had never been in any diseased district whatever, and who exhibited the same symptoms nearly about the same period. Hence, then, by my definition I am justified in concluding that these four individuals laboured under the effects of a morbid epidemic influence. If on the other hand, Paterson had just returned from Glasgow—if she had then been seized with cholera—if the greater number of her attendants had been seized likewise, but none else in the immediate neighbourhood—if moreover this had been the exact case with the *very great majority* of individuals attacked during the first ten days, then I would have inferred that the introduction of cholera into Dumfries was not to be ascribed to epidemic influence, but was to be attributed either to infection or contagion—to communication betwixt healthy and diseased individuals. So much for the rise. Now as to the progress of this epidemic in Dumfries.—In that range of buildings commencing with the King's Arms entry, along the High Street, and turning along Shakspeare Street, as far as the opposite entry of the King's Arms, and in all the intermediate lanes and passages, cholera has been severe. On the opposite division of High Street, the number of cases has been comparatively small, and on the corresponding division of Shakspeare Street only one case, and that one clearly imported. I state this fact on the authority of the resident district surgeons, Dr. M'Culloch and Mr. M'Kenzie. In that portion of Loreburn Street, commencing with the Relief chapel, and terminating at Gowanlock's close, the population on the east side is about 33; on the west side about 40; on the east side 9 individuals were attacked—on the west side not one, except a child, who died with suspicious symptoms. Out of eight of the closes, comprising a portion of Old Fleshmarket Street, commencing with Seaton's close, in the first four the cholera has been severe; in the last three even unusually fatal; in the fifth, the Cross Keys close, *not one* case has yet occurred. In that division of Old Fleshmarket Street alone, between 60 and 70 cases occurred. On the corresponding portion of the opposite side *not one:* nay, I have been told on good authority that in all that range of buildings betwixt that portion of Fleshmarket Street and the Town Hall, there has been *only one* well-marked case of cholera. Lastly, I may mention that I have been informed by Dr. Grieve, physician to the Infirmary, that a patient who came from the country, after being three days resident in that institution, was seized with cholera, but that the case has as yet proved a solitary one. These facts are so striking—so consistent with the wayward course of an epidemic—so contrary, nay, so utterly opposed to the usual tract of an infectious or contagious disease, that it would be alike unnecessary and impertinent in me to draw from them any elaborate deduction. I beg leave to state in conclusion, that I have never yet seen or heard of any case in Dumfries, which could not be most satisfactorily accounted for upon the hypothesis of epidemic influence; but a very great majority which can in no wise be accounted for either on the principle of infection or contagion. Is it not, then, altogether unphilosophical to adduce a second and more unlikely hypothesis, when one both more satisfactory, and more capable of demonstration has already been adduced? I must now apologise for having detained you so long. Yet would I fain trespass a few moments longer upon your attention, whilst I endeavour to refute an objection which has been raised, not by my opponents, but by certain sincere, yet timorous supporters of my hypothesis. The objection has been raised on the ground of expediency. "We are anti-contagionists," say these alarmists, "but certain medical men are contagionists; therefore impress the doctrine of contagion upon the minds of the unprofessional. It is the safest side to err on." I am grieved to say, candour requires me to confess that the premises of the proposition are undoubtedly correct; for some medical men are as *yet* contagionists; but the conclusion of the proposition, so far as relates to the comparative danger of the two doctrines, seems wholly untenable, and appears to me the deduction, not so much of cool reason as of partial and satirical observation. What, Sir! is there no danger in increasing the grand predisposing causes of cholera? no danger in increasing panic throughout the land? no danger in increasing the want of the necessaries or of the comforts of life? And moreover, is there not a far greater moral danger in tearing asunder with rude and rugged grasp all the dearest and tenderest sympathies of humanity? In separating the father from his son's death-bed—the son from the father's.—Aye, the mother, too, from the infant's whom she bore? Is there any one now present who has never witnessed such scenes? If such an one there be, he is more fortunate far than I have been. I have witnessed many such—some of them but too lately. Finally, I would ask is there any one here who has ever heard of the selfishness, the cruelty, and the brutality so frequently exhibited in every country in the world (but in these only) where the doctrines of infection and contagion have been successfully promulgated, who will now be prepared to deny that the tendency of these doctrines is alike hateful and pernicious? Yet, if they be true, deny them not.—"*Fiat justitia, ruat cœlum.*" If, however, as these alarmists readily confess, such doctrines are unfounded and sophistical, then it becomes a sacred and a social duty to remedy, if it be yet possible, an evil so terrible and vast—to impress the true opinion upon the minds of all our countrymen—to tell them confidently, that should they withhold even their personal attendance from the poor man's deserted death-bed, it will not shield them from this noisome and wide-spreading pestilence—to tell them that there is but one safeguard on which the Christian can rely, and that safeguard is the moral courage that results from faith in that Almighty Being, "who rules the whirlwind and directs the storm." Dr. R. J. concluded by moving the resolution, which will be found at the head of this article.

Mr. Charles Bell, in rising to second the resolution, said—After the very impressive speech they had just heard, it was unnecessary to say more than merely second the resolution, which he did unhesitatingly, as he most cordially agreed with all that had been said on the subject by Dr. Jameson.

Dr. M'Culloch.—I have only to remark, that after the eloquent, able, and conclusive speech of Dr. Jameson, it would be a work of supererogation in me to adduce more facts in proof of the non-contagious nature of Cholera. I have only to state, in continuation of my learned friend's able remarks, that it is both a matter of expediency and humanity to make these (in my mind) unanswerable arguments public, because, not only is the trade of the place suspended, but all connexion, generally speaking, with the surrounding country is cut off, thereby not only making provisions dearer, but depriving a great part of the population of the means of earning a livelihood, and consequently creating much famine and misery, most lamentable to contemplate; and almost to a certainty superinducing a second visitation of the pestilence from its very effects, viz. famine and frightful consequences. I now have only to declare my sincere conviction, that Asiatic Cholera has been in no instance that I have either seen or heard of, a contagious or infectious disease.

Dr. Thorburn observed, that able as Dr. Jameson's statement was, he must confess that his opinion, as to the communicableness of Cholera, remained unchanged; but before proceeding to give the grounds on which that opinion rested, he would beg leave to say, he by no means pretended to attempt answering Dr. J.'s remarks at present, as he had come to this meeting totally unprepared, from an expectation that the task would have fallen to much abler hands; but as he saw that several of his most experienced and talented medical friends, who entertain similar, or at least nearly similar opinions to his own, were absent, he would content himself with offering a few cases and observations in illustration of his view of the subject; he need hardly add, that he had prosecuted this important enquiry with every possible impartiality, and that though his hopes and wishes, from motives of personal feeling, popularity and interest, very naturally inclined him to view the disease as totally *non-contagious*, he had, nevertheless, found that such a conclusion would be quite at variance with a conscientious consideration of facts. Being absent during the occurrence of the first well-established case, and not yet having had leisure to enquire minutely into the circumstances of it, he could not speak so positively as was desirable, but he had no doubt the woman first attacked had contracted the disease from washing infected clothes belonging to one or other of the many strangers who at that time had flocked to Dumfries to witness the ascent of the balloon. Even were there no room for such a probability, it would not at all militate against the doctrine of contagion—for who has been able to trace the commencement of typhus or scarlet fever (diseases generally admitted contagious) here or elsewhere, to distinctly contagious origin? Nay, the proofs that cholera was directly imported, through the medium of goods, from Hamburgh to Sunderland, is at least as clearly established as most instances of the former visits of plague to this country or France. But he would now speak of what he had seen here. On the day of his return he went to visit a woman named M'Kaig, residing in St. Michael Street, and found her in the collapse stage of malignant cholera. What was her history? Her mother had just that morning died from a like attack, and had been carefully attended, her dead body dressed, and the sheets on which she expired burned, by this patient, who died a few hours after he saw her. On the same day, a man, Gass, living in Church Street, Maxwelltown, was seized; he was carefully attended by his son, who lived in Dumfries; the latter soon followed his father to the grave. Next morning, a man, Dixon, in Mill Brae, was seized; his family were so stupid and timid that his brother, who resided near Terregies' Manse, felt it incumbent on him to attend him during his last illness; he, and a daughter of the patient stretched the body, &c.; three days after the brother died at his own house of cholera, and the girl has also been attacked. In the same quarter, a poor woman died, and no one had the courage to approach the body except a girl named Halliday, who within a few hours also died. Then again, in Church Street, a man, Murray and his wife; in Red Lion Street, Wylie and his wife, and step-son; also, in the same street, M'Kilver and his wife were all one after the other attacked and killed by cholera. In Market Street, a man named Hay and his wife died in the same way; and their son and step-daughter, either on the same or following day, went to the house, eat and drank what was to be found in it, putting on some of the apparel of their deceased parents, and on the same evening they were attacked with cramps, vomiting, and purging, and other symptoms of cholera, though not severe enough to destroy life. A tailor named Carson, residing in Glasgow Street, was attacked—his wife, being then resident in the country, was instantly sent for; she, a sister of Carson, and a young woman named Mitchell, assiduously attended him to the last; the sister died four-and-twenty hours afterwards, and the other two just named have also had the disease. These cases coming under the actual observation of one out of a score of practitioners, are too numerous and consecutive to be called exceptions. How many similar must not others have had in the same short space of time and limited field! There are instances of entire families being seized, one after the other in Dumfries. At first one case, he understood, occurred in our jail, it was immediately followed by others; ditto in the Hospital. He believed *one* out of every *three* of our practitioners had been attacked, more or less severely, with cholera. Several nurses on duty in the Cholera Hospital have had the disease; two letter-carriers, one after the other, have died of it. What is the common-sense deduction from these facts? Is it not that all those who are most directly exposed to the atmosphere, poisoned by emanations from the sick and dead, were attacked in a ten times greater proportion than others—thereby exactly following the course observed in other contagious disorders. The non-contagionists say that the poison exists in the air,—granted; but *what* poisons the air? "O," say they, "it is something generated *in* the air, earth, or clouds." Have any particular changes in the elements been ever seen, heard, or proved to have taken place since 1817, when cholera first assumed its present character? Certainly not,—it is natural to conclude that since the foundation of the world the same terrestrial and atmospheric changes, as may have existed since 1817, had previously repeatedly taken place. Intermittent fever does, confessedly, originate from natural operations, but these we can, in most cases, trace or even see,—never so with regard to cholera. Now, we do, however, see individuals suffering from typhus, from cholera, &c. we know that certain exhalations arise from the morbid body into the atmosphere, and that those most exposed to this body, and to this atmosphere, are repeatedly attacked with a similar disease. Non-contagionists admit this practically, for they allow, that a person entering a sick room, is in more danger than outside of the door or wall. Now, how is this to be explained, but that the diseased body is contaminating the air of the room with a poison, similar to that under which it suffers.—These, and numerous other circumstances and observations, had brought him unwillingly to conclude that cholera derives its chief, nay, its only source, from poison emanating from the human body, and that it can be propagated in the same way as many other confessedly communicable maladies. As to the primary cause of cholera we know nothing, save that, like plague, or animal existence itself, it derived its origin from the hand of Heaven.

Mr. Charles Bell, in reply to Dr. Thorburn, said—He did not consider the cases brought forward by Dr. Thorburn tended at all to prove that cholera was contagious; on the contrary, they only proved that the atmosphere breathed by those patients was vitiated, by some unknown cause, and unless Dr. T. could produce several well authenticated cases, where people labouring under cholera, were carried to a distance from the infected district, and there communicated the disease to their attendants and neighbours, he could not admit that it was contagious or infectious; for the circumstance of one individual being infected by going to see another in cholera in an infected locality, could not establish his assertion. It might as well be said, if a person were drowned by going into a river to the assistance of another, that he was drowned by coming in contact with that person, and not by the water which surrounded him.

Dr. Reid—There is one circumstance which I would beg leave to lay before the meeting, as it appears to me to serve nearly all the purposes of an experiment on a large scale, in assisting us to come to some satisfactory conclusion on this question, and that is, what has occurred in the House of Refuge. We have there about a hundred children and widows, a great majority of whom have been kept in that house for the last fortnight, and all of whom immediately before their removal to the Academy were exposed some for a few hours, others for probably three or four days to the contagious influence of this disease, if there is any such thing, and yet upon inquiry I find that only one doubtful case has there shown itself. Some gentlemen I know seem to think this fact to be rather a proof in favour of contagion than against it, for say they, "the people in the academy have been removed from the contagious influence, and therefore have been comparatively free from the disease." If these individuals had not been exposed to the influence of contagion before their removal to the house of refuge, I grant them it would be a powerful negative proof in favour of contagion, but as the case now stands, it appears to me to be a very powerful argument in favour of the epidemic influence being the sole cause of its propagation. It must be remembered that when these individuals were removed to the House of Refuge from the houses where their fathers and husbands had lately died, and been carried out to the grave, that they were not removed from the contagious influence, but carried it along with them to their new abode, and indeed would be as much exposed as if they had remained in their own houses; for the bodies of their dead relatives, the only cause of contagion or infection, had been removed. It is perfectly well known that infectious and contagious diseases do not seize upon their victims *immediately* after exposure to their baneful influence, but it is generally a few days after exposure, that symptoms of the disease appear. It may be proved to demonstration that if cholera be an infectious disease, it resembles other infectious diseases in not attacking individuals immediately on exposure. We are all aware that it has frequently happened that individuals, after leaving a town suffering under cholera, and removing themselves to places perfectly free from the disease, have yet been attacked several days after their removal. And if any person should think that these individuals now in the House of Refuge, were not sufficiently exposed to the contagious influence before their removal, I may mention that I know it to be the opinion of a medical gentleman, whose authority on this or any other point connected with the profession, stands as high as that of any other medical man in the island, that if cholera is at all contagious, a very slight contact with persons affected is sufficient for the purpose. Will any gentleman pretend to say that if as many people had been equally exposed to measles, scarlet fever, or small pox, who had never been vaccinated or inoculated, diseases undoubtedly contagious, that an infinitely greater proportion would not have been seized? What must render this fact of no small weight, is the great number of individuals of different ages upon whom this experiment, if I may so call it, has been tried, since I believe that a great deal of the endless discussion on contagion and non-contagion has arisen from hasty generalization, from inquiries drawing general conclusions from insulated facts, as the rise and progress of the disease in one situation cannot be applied to every other; for what epidemic or contagious disease is there, whose type and character is not altered by time or place? It is, therefore, with great pleasure that I find the general question of the contagion or non-contagion of cholera to have been carefully avoided in the resolution now proposed to the meeting, and that we are only requested to give our conscientious opinion of what we have seen in Dumfries. I should wish that the gentlemen who look out for those cases which apparently serve to strengthen the doctrine of contagion would also give us all the insulated cases which have fallen under their observation. Now, if their experienee has been similar to mine, they will find these to have been pretty nearly equal, and since the introduction of the disease has been clearly and indisputably shown by Dr. Jameson to have been dependent upon epidemic influence alone, I am astonished that these gentlemen should think it necessary to call in the agency of another cause whose existence is at least problematical, when all the cases can easily be explained by the agency of a cause whose existence is undoubted. I therefore most heartily support the motion of my friend, Dr. Jameson, who certainly deserves the best thanks of this meeting for the very able manner in which he has investigated the subject now brought before us.

Mr. MACBRIDE.—As an argument against the present epidemic originating from atmospheric influence, it has been asked, how does it break out in one place without shewing the least symptom in the immediate vicinity? and how can you account for an individual having caught the disease after seeing a near relation, but by contagion? To such querists I would answer—that it was not from the person affected that the visitor caught the disease, but from inhaling the atmosphere where the disease originated—being predisposed by fear, or by agitated feelings in seeing his friend in such agony. It is well known, that at a certain height there are currents of air moving in opposite directions, and from late experiments it has been proved beyond doubt, that these currents sometimes come in contact with the earth. Now, Sir, from what I have stated, it is easy to conceive how one place may be affected, and its immediate localities escape; for instance, the current or vein of air containing the epidemic virus may have come in contact with the earth in that particular spot, and thereby affecting those who are predisposed in a very severe form, owing to the virus being concentrated; but as the virus intermixes with pure air, and becomes more diluted, in like manner the disease becomes more mild.

Dr. GRIEVE stated that in addition to the case already adverted to by Dr. Jameson, he could adduce many which had occurred in his private practice, and in the hospital, in proof of the non-communicability of cholera, but he thought it unnecessary to enter into a detail of them at present when so many facts illustrative of the non-contagious nature of the disease had been brought forward. He thought that the absence of all proof of the malady having been imported was much against those who advocated the doctrine of contagion. Inanimate matter, as clothes, furniture, &c., in his opinion, were not capable either of receiving or communicating the supposed contagion. He had no doubt that the disease originated in Dumfries from a local miasma—the specific nature of which he would not attempt to explain—as it had done at Jessore, in Bengal, in 1817.

Dr. Ross Jameson.—I beg leave to say a few words in reply. If I understand aright the opinion of Dr. Thorburn, he denies the existence of an epidemic influence as affecting the rise and progress of cholera. His arguments rest solely on the fact that occasionally cholera attacks the majority of a family. But this by no means bears upon the point in dispute. All epidemic diseases do the same. I think I have pretty clearly proved that the rise and progress of cholera are to be attributed to a morbid epidemic influence. —Now as far as regards the opposite side of the question the *onus probandi* lies with my opponent. No man can prove a negative. The fact of infection or contagion (as I have already remarked) can only be proved by an individual coming from a diseased into a district decidedly healthy, taking the complaint and communicating it to the attendants who *alone* in the locality are affected. It is by the production, not of one or two isolated cases, but of a *very considerable* number of such facts alone that the infectious or contagious nature of cholera in Dumfries can be proved. These facts do not exist.

The motion was then put from the chair, and carried by a majority of 11 to 1.

Mr. Spalding moved that the thanks of the meeting be tendered to Dr. Jameson for the trouble he had taken in calling them together, and for the opportunity thus afforded them of declaring their sentiments upon the subject so ably submitted by him to their consideration; which, being seconded by Dr. M'Culloch, was unanimously carried.

Dr. Ross Jameson returned thanks.

After a vote of thanks to the Chairman for his able and impartial conduct in the chair, the meeting separated.

### XLVI.

### Injury of the Penis, and Amputation of the Organ.

This case is related in the Lancet for Sept. 22d, by Mr. Williams, an intelligent surgeon of Llandovery. We will give an abridged account of it.

*Case.* In April, 1832, a farmer's son, æt. 11, had his penis bitten through his trowsers by a brood mare. No wound was inflicted, but mortification ensued, and on the separation of the slough the glans was left attached to the body of the penis by a small isthmus inferiorly. The wound was healed under the superintendance of an old woman, and on the 22d July the boy was taken for the first time to Mr. Williams.

The penis now presented no great deformity, but the urine escaped guttatim through an opening in the cicatrix scarcely large enough to admit the point of a bristle, and situate on the dorsum of the penis, just behind the corona. The portion of urethra lying in the glans was quite impervious, and formed a stricture, about three-fourths of an inch in extent. The bladder was much distended. Mr. Williams determined to amputate the penis, just behind the injured part, and in this opinion Dr. Bowden, of Carmarthen, coincided. The operation was performed as follows:—

"The root of the penis being held by an assistant, the glans and prepuce were grasped between the fingers and thumb of the left hand, and gently drawn forward, so as to elongate the penis; then, with a straight-bladed bistoury, the whole of the parts that constitute the organ were extirpated at one stroke, directing the incision from below upwards.

The urine was immediately evacuated in a full stream, and the force with which it was propelled, showed that the bladder had not lost its power of action. After making pressure for a short time on the bleeding vessels, the flow of blood was arrested without having recourse to ligatures, and the wound was dressed simply with dry lint. I found the next morning that a slight hemorrhage had taken place in the night, but it had now completely stopped; the urine freely passed, and a poultice was applied to the wound, which had now become considerably inflamed and swelled. A slight hemorrhage returned two or three times, generally in the night, but the inflammation and swelling soon subsided, the bleeding disappeared, and the surface began to granulate."

In order to try whether the orifice of the urethra would contract if left to itself, no bougie was introduced. At the end of nine days the wound was nearly healed, and the orifice so contracted that the point of a small probe could scarcely be introduced. Bougies were employed, and in four days a common-sized one could be passed. At the end of a fortnight the bougie was discontinued for 24 hours, when the urethra was again found very contracted. The bougie was resumed, and has been continued up to the 12th Sept. the date of Mr. Williams' communication. It will be seen, on referring to the operation, that no precaution was adopted to preserve integuments, yet they overlap the extremity of the stump sufficiently to offer no impediment to future erection of the penis.

We must confess that, so far as we can judge from the description of Mr. Williams, we should have been rather tempted to save the glans than to have removed it. Amputation of the glans is a serious evil—first, because it is the seat of much of the venereal pleasure—secondly, because the penis is much disfigured by its absence—thirdly, because the extremity of the urethra on the surface of the stump, after amputation is especially liable to contraction. We should have been inclined to attempt opening up the impervious urethra in the glans, not by the caustic bougie, but by a small trocar, and the subsequent employment of bougies.—Supposing that this attempt had failed, we believe it would have been better to have dilated the orifice on the dorsum of the penis behind the glans, than to have amputated the latter.

### XLVII.

### Removal of Calculi from the Female Bladder by Weiss's Dilator.

In the number of the Lancet containing the preceding case, is a short communication from Mr. Lovegrove, of Horsham.

A young woman, æt. 20, had distressing symptoms of stone in the bladder. After trying "various alkaline medicines," Mr. Lovegrove determined to remove the stone by dilatation of the urethra. He introduced the dilator, and gradually expanded the blades till they were opened to their greatest extent; this occupied forty minutes. The dilator was allowed to remain twenty minutes and was then withdrawn, when a calculus the size of a walnut was expelled. The patient was ordered an opiate and nothing untoward occurred. For three weeks all went well, but then other calculous symptoms occurred.—The operation was again resorted to, and was repeated three different times at varying intervals, the bladder on each occasion expelling two, sometimes three calculi, from the size of a walnut to that of a hazel nut. The patient retains her urine "as well as before the operation," but we are not informed how well that is.

Mr. Lovegrove should have stated the kind and composition of the calculi, and also the condition of the urine. We cannot judge of the propriety of administering "various alkaline medicines," in the absence of such information. Supposing that the urine was already alkaline, and that the calculi were composed externally of triple phosphate, we need scarcely inform our readers how injurious alkaline medicines would be, how calculated to establish or maintain an alkaline diathesis. We do hope that practitioners will attend to these points, and that they will record cases in a more scientific and exact manner than many have hitherto done.

### XLVIII.

### Physic and Surgery.

We have been much pleased with the perusal of the introductory lecture of Mr. Samuel Cooper, delivered to the surgical students of the London University on October 3d, 1832, and reported in the London Medical and Surgical Journal for October 6th. After giving a brief, but distinct and interesting sketch of the history of surgery and physic, he takes up the consideration of the present actual division between them. The sentiments of Mr. Cooper on this head perfectly coincide with what we have ourselves on several occasions expressed, and we cannot allow the opportunity of recording them to escape us.

"An interesting question now presents itself—has the division of the profession, into physicians and surgeons, assisted or retarded, its improvement? This is a point, on which it may be difficult to give a ready answer. Perhaps I should be justified in saying, that the division of practice, the division of labour, has had good effects, particularly when such division was exercised by men, who had the same foundations, and began their respective careers, enriched from the same stores of science; for, gentlemen, if I am certain of any thing, relative to professional education, it is that medical and surgical practitioners should all go through precisely the same elementary studies. Thus far I concur with many enlightened members of the profession; because, in whatever way the question, about the division of practice, may be disposed of, the unity and indivisibility of the science itself must continue. But I completely disagree with those who seem to desire nothing less than the annihilation of the physician and regular surgeon altogether. Human life is not long enough, and human faculties are not powerful enough, for any one man to attain, in both departments of the profession, the point of perfection, to which the talents and industry of many generations have now brought them. Had he the longevity of a patriarch, his time would yet be insufficient for so ambitious a purpose.

I calculate, that the young physician and the young surgeon, who mean to reach the temple of fame, ought to commence their journey and travel together many miles along the same road; but that, when they have proceeded a certain distance, they must diverge a little, each taking the path leading to the summit of that branch of practice to which he is particularly devoted. Each carries along with him, however, the knowledge both of physic and of surgery; and each is endowed with all that variety of information, which I have represented as forming the basis of medical science. For my own part I should never have any confidence in a physician ignorant of surgery; nor is it possible to suppose any man entitled to the name of a surgeon, who knows nothing of physic."

We can scarcely add any thing to these observations, unless we were to state in still stronger terms our disapprobation of the attempt, or rather the wish, for the attempt would be preposterous, to annihilate the physician and consulting surgeon altogether. Such a notion argues an utter ignorance of the natural progress of civilization, and the entertainers of it might as well proclaim at once that they think the establishment of Owenite communities possible. As man becomes civilized and social establishments gain strength, the division of labour likewise increases. In the wigwam of the savage, each individual is the manufacturer of most, if not the whole, of his necessaries and comforts. When a village is formed, the same individual practises many trades—is cobbler, tailor and draper. When commerce has erected that village into a town, the united trades are dissevered, and one person follows but one calling. The town increases to a city, such perhaps as the mighty one we dwell in, and with the augmentation of inhabitants is a proportionate augmentation of the subdivisions of labour. The tailor is no longer the artisan, he does not make, perhaps he scarcely sees, the clothes he sells. One workman fabricates the trowsers, another sews the coat, a third gets up the waistcoat, nay, waistcoat-making itself becomes a separate craft, and may be again sub-divided. Some of our readers may recollect the satirical remark of Lord Byron on this head :—

None are complete, all wanting in some part,
Like certain tailors, limited in art.
For galligaskins Slowshears is your man;
But coats must claim another artisan.

Turn which way we will, we meet the same subdivisions, continually increasing with the progress of society. Is it natural to expect that medicine can resist the operation of a law so general and so powerful? It is not; and we repeat, that the idea of annihilating the distinctions of physician and surgeon is at once preposterous and impotent.

The point to which all should direct their attention is the education of the young man. Let that be rendered as general as possible, and let circumstances or inclination determine his subsequent choice of a particular department. This, however, is not the whole of the case, nor is this the perfect solution of the difficulty. The general practitioners are a class continually increasing in intelligence and respectability, qualifying themselves for a high station in medical society, and determined to assume it. Yet the general practitioner is a sort of homo non in our constitution—he belongs to the College of Surgeons and the Society of Apothecaries, he really supports them, and yet he is an out-cast from either. These are anomalies that need not exist, that cannot endure. They are not the produce of present civilization, but the remains of institutions of a former æra. The day for the destruction of such things may be more or less protracted, but so surely as the mind of man does not retrograde, they will sooner or later be swept away.

### XLIX.

### Clinical Instruction.

In the Quarterly Journal of Education, for June last, it was asserted that the system of clinical instruction in Edinburgh was so infinitely superior to that adopted in London, that the latter was quite undeserving of the name. In the Introductory Lecture delivered by Dr. Elliotson, in the London University, on October 1st, that able physician takes up the gauntlet, and vindicates his own system of clinical instruction from the injurious aspersion of the reviewer. The comparison is interesting, and may perhaps afford some useful hints.

"Really, when he says that the clinical teaching of London will not bear comparison with that of Edinburgh, for that we ought to show the pupil 'how to use his eyes, his ears, his hands,' he convicts himself of perfect ignorance of the present clinical teaching of London, in those hospitals where clinical instruction is given at all. My plan has always been, to spend two or three hours at the visit; to converse familiarly with the pupils on the cases; to request every one to observe the countenance of the patient, the expression and hue of which are often sufficient to indicate the seat and nature of the disease, and always to indicate the changes that have occurred since the previous visit; to request every one to notice the appearance of the tongue, and to feel the pulse; to present each with my stethoscope who has not one, and stand patiently at the bed-side while he is listening; in short, to act the part of a private tutor in the wards to each, just as the demonstrator does in the dissecting-room. A true clinical lecture is thus given at every visit, though it does not bear the name, and is not published. Once a week I take a general view of the cases that have terminated, classing similar cases for comparison, contrasting others, applying general remarks to particular cases, and presenting a short abstract of each case and its treatment.

In Edinburgh, the lectures, as far as I have heard and read them, are similar; and if two are delivered in the week, and the remarks are sometimes of a more elementary character, this is but a compensation, and in my opinion, is very far short of a compensation, for the defective teaching in the wards. When I studied in Edinburgh, but a small number of the pupils got to the bedside; the greater part stood about, seeing few of the patients, and contenting themselves with hearing announced what was the state of the pulse, the tongue, the surface, the bowels, &c. and noting each announcement down as it was made. We might almost as well have read a case in a journal. Few or no remarks were made by the physician or surgeon; no reasons for his particular practice were given—no conversation took place, the students were not individually instructed in the use of 'their ears, their eyes, and their hands;' all was a dry announcement of the symptoms and the prescription of the day."

In the lecture from which the foregoing extract has been taken, there is much to challenge approbation. It is in keeping with the spirit of the times, and if the Senate and Professors of the University effect what they openly promise, by the mouth of Dr. Elliotson, it requires no gift of prophecy to foretel, that they will revolutionize the schools of medicine in London. After making an earnest and eloquent appeal to the public, to erect and support a hospital in connexion with the University, Dr. Elliotson proceeds as follows.

"It is the custom, where the physician does his duty to the pupils, for certain young gentlemen to hold the office of *clinical clerks*—to examine and draw up an account of each case at its admission, and keep a daily report, in books which are open to the rest of the pupils. For the privilege of performing this labour, they of course pay nothing. But other young gentlemen who hold a similar office under the surgeon, and have much drudgery to perform in dressing sores, bleeding, extracting teeth, and passing bougies, thus performing no small share of the humbler business of an hospital, under the name of *dressers*, actually pay, in addition to a large entrance fee, no less than fifty pounds. With us, I trust they will be placed on the footing of the clinical clerks, and pay nothing additional: Lists will be kept of the most meritorious pupils, and clinical clerkships and dresserships bestowed upon them in succession; none being permitted to retain his appointment longer than six months while another on the list is waiting for it. *(Loud applause.)* It will also be worthy of consideration, whether the most distinguished and able may not ultimately be allowed to practise, with certain limitations, under the eye of the physician or surgeon; and whether the office of house-surgeon, which will be annual, should not be annually given to the most distinguished surgical pupil. I trust that every day a physician and surgeon will visit all their patients, and at different hours, so that the students may go round with both."

This is as it should be. In order that a young man shall be house-surgeon at most of our London hospitals, it costs him a considerable sum. But this is not all. His election to the office is rather a matter of seniority than a reward of merit, or, at all events, it is not a gift to the most meritorious. By the university regulation, the office will annually be conferred on the most distinguished surgical pupil. If such principles really guide an institution, if such a system be sincerely, zealously, and steadily pursued, every man of liberal feeling must applaud, though private partialities might direct his wishes to other quarters.

---

### L.

### Mr. Carmichael on Tracheotomy.*

Our Dublin contemporary increases in value with each succeeding number, and it does no discredit to the character of the profession in Ireland. In the No. preceding the present, was the commencement of a review of Dr. Stevens' work upon the Blood, which evinced the sound judgment of the editors. At that time, we believe that the reviewer stood alone in his condemnation of the

* Dublin Journal, No. V.

Doctor's absurdities—too many of the medical periodicals appearing to have been humbugged by this modest gentleman.

The first paper in the No. before us is by Mr. Carmichael, and entitled, "Observations on the Use of Tracheotomy in Chronic Diseases of the Larynx, illustrated by Cases." In Mr. C.'s Essay on the Venereal Diseases, a work which we presume that most medical men have read, and all should read, he submitted the propriety of tracheotomy, in some of those miserable cases of venereal ulceration of the larynx, which are always so obstinate and often so fatal. "The presumed modus operandi (he observed) of an opening into the trachea, as a remedy, is to allow the patient to breathe through the artificial opening, and permit the larynx to remain undisturbed by the presence of a constant current of air, and thus induce that favourable state of quiescence, which is necessary to the healing of an ulcer in any situation." At that time, Mr. Carmichael had not tried the operation, but since that period he has had very ample experience of the remedy, and can confidently recommend it. Mr. C. relates some cases; let us look at them.

*Case* 1. "The first case that occurred to me, in which I performed tracheotomy for this form of disease, was that of a man who had lost the velum and uvula, from venereal ulceration; ulcerated patches were visible at the time of his admission, deeply seated on the back of the pharynx, while the superior part of it exhibited the cicatrices of former ulceration. His voice was so hoarse as to be almost indistinct, while a constant croupy cough, and incessant endeavours to hawk up a viscid phlegm, marked sufficiently the existence of ulceration of the larynx. He was greatly enervated, and so exhausted by the malady and the means which had been previously employed, consisting of mercurial courses and fumigations, blisters, caustic issues, &c., that the late Mr. Todd, in consultation on the propriety of the measure, observed, that any experiment was fair in a case in which all the known means had failed, but that in his opinion neither operation or any thing else could save the man's life. The operation was however performed, in his presence, that of Mr. Peile, and the pupils of the hospital—and the man experienced immediate relief, rapidly recovered, and was discharged the hospital in three weeks afterwards, perfectly well."

The next case was very nearly similar. The operation was followed by immediate relief; the patient was discharged well in a fortnight, and has had no return of the disease.

Now it may be objected to these cases, that if the recovery could be so speedy as it was, the disease itself could not have been extensive. It is difficult to imagine that serious ulceration of the larynx, or that chronic disease of any extent, could be perfectly removed in a fortnight under any circumstances. Whether this view be strengthened or invalidated by the following case, we will let our readers determine.

*Case* 3. "M'Namara; about forty years of age; broken-down constitution; countenance anxious, sallow, and emaciated; admitted into hospital in the summer of 1826. The man attributed his present complaint to cold caught after a severe mercurial course for venereal, the remains of which were at the time well marked on his thighs, legs, and chest, cicatrices the size of half a crown, livid, smooth, and slightly depressed; voice almost gone, the whisper *hoarse;* attempts to speak with great exertion, heaving of the chest; the inspiration I recollect to have been greatly laboured; his chief complaint, a soreness which he felt in the larynx, which was painful on pressure, accompanied by a hollow sounding cough and expectoration of tenacious phlegm; his breath very offensive. The treatment was pills of calomel, hippo, and opium, repeated blisters to the fauces and nape of the neck. He was between a fortnight and three weeks in the hospital before the operation was performed by Mr. Carmichael; little or no blood lost at the time, but a troublesome hæmorrhage followed after the operation, from small capillary arteries and veins; it was more an oozing from the edges of the wound, which during the evening became tumid, everted, and painful; it was, I recollect, particularly distressing to the patient, from the severe dyspnœa that came on from the blood making its way into the trachea, but it was easily suppressed by lint and the pressure of the retractors, which kept asunder the edges of the wound. Late the same evening, Mr. M'Dowell was sent for (as Mr. Carmichael was not in town), who enlarged the wound in the trachea, upwards, with bistoury and scissors, as the opening did not appear sufficient for the passage of the frothy mucus, which collected in large quantities. The man died the following morning.

On examination, the epiglottis was so thickened as to be almost immoveable, and appeared incapable of acting as a valve to the air to the larynx during the passage of the food down the stomach; a circumstance which occasions, in these unfortunate cases, great additional suffering to the patient whenever he is obliged to take nourishment. The lining membrane of the larynx was every where so much thickened as nearly to close its cavity, and on the lower part of the thyroid cartilage was a large, foul, and deep ulcer, of a size sufficient to contain a large bean. The diseased parts are preserved in the museum of the Richmond School."

That this patient would have died under any plan of treatment is most probable; but tracheotomy did not save him. Now these are all the cases of venereal affections of the larynx, for which tracheotomy was performed. Mr. Carmichael, indeed, says he has not brought forward one half the cases he might have done; but the public must judge by the evidence presented, not by that which is withheld. If our readers are satisfied with that evidence, it is well; we must own that it does not bring conviction to our own minds, of the powers of tracheotomy in severe venereal affections of the larynx. At the same time, we would earnestly direct the attention of surgeons to the subject. Mr. Carmichael relates two other cases in which tracheotomy was performed, the one an instance of chronic, the other of acute inflammation of the larynx. We will briefly mention them.

*Case.* A lady, aged 50, had all her life been subject to catarrhs, which always occasioned hoarseness. In Dec. 1831, the voice was nearly gone, and she had most of the ordinary symptoms of chronic laryngitis. In February she could only speak in a stridulous low whisper. She consulted Mr. Colles, who made her mouth sore and herself worse. A solution of argent. nit. (ʒss. ad ℥j.) was applied to the pharynx by means of a piece of wire, with lint attached to the end of it, passed through an elastic tube. This was abandoned, in consequence of the irritation which it occasioned. Towards the end of April, her feet and legs began to swell, and orthopnœa was very distressing. On the 4th May, Mr. Carmichael was called to her. The dyspnœa was very urgent, the inspirations croupy, the lower extremities œdematous to the knees, and the patient had been unable to lie down for several weeks. On the 5th, Mr. C. operated, and on that night she slept. On the 7th, the breathing was much oppressed, and the margins of the wound swollen. A number of leeches were applied, and followed by great and decided relief. Her subsequent recovery was progressive. She breathes through the wound, which is kept open by two concave plates of silver, that form a tube when pressed together. She cannot inspire at all by the natural opening, and speech is annihilated.

There can be no question, as Mr. Carmichael remarks, that in this case there was great thickening of the lining membrane of the larynx, that that thickening was the real cause of the destruction of the voice, and, but for the operation performed, would certainly have destroyed her life.

In "all the other cases of affections of the larynx, either acute or chronic, upon which I performed tracheotomy, the voice was gradually restored as the inflammation and swelling of the larynx subsided; the wound in the trachea, notwithstanding the removal in every instance of a portion of the rings, progressively closing as the obstruction to the passage of air in the larynx diminished."

CASE. *Acute Laryngitis—Tracheotomy.*

On July 20, 1831, a boy, æt. 2, either drank some boiling water from the spout of a tea-pot, or, what is more probable, inspired the steam. He did not suffer much till late in the day, when a gentleman leeched his cheek and gave him some purgative medicine. On the following day Mr. Carmichael was sent for, and found the child breathing as in the last stage of croup. With the assistance of Mr. Adams he performed tracheotomy. Difficulties occurred, but they were surmounted, and a circular piece of the trachea was cut out. Next day there was re-action, which was put down by venesection. Every now and then paroxysms of dyspnœa would occur, from the accumulation of tough mucus in the trachea. On these occasions a probe armed with lint was introduced by the attendants, and swept up and down the trachea, with immediate relief. In ten days the child was well, and has continued so. He is now three years old; there is a cicatrix half an inch in length; the respiration and voice are perfectly natural.

"The operator," says Mr. C. "will find great advantage by employing a double hook, which he may boldly plunge into the trachea, as soon as the rings are sufficiently bared for the purpose. By this measure he fixes a part which it is difficult to operate on with safety, as it is in perpetual motion in a person struggling for breath, and at the same time situated at the bottom of a deep wound, and surrounded by the most important vessels: but by means of the hook he is enabled to draw forward the trachea and perforate it with ease. I always employ scissors for this purpose, and cut out a portion of the trachea, as has been fully detailed in two papers on the subject inserted in the 2nd and 4th vols. of the *Transactions of the Association of the Dublin College of Physicians*, in which a considerable number of cases of tracheotomy is detailed, in not one of which was a tube introduced. The opening made in the trachea being found quite sufficient for the passage of air and mucus, and not attended with that irritation which the introduction of a foreign body within the highly sensitive internal lining of the trachea must inevitably occasion.

Let no young practitioner estimate the performance of tracheotomy by the ease with which it is done upon the dead subject. In the living, the parts we have to divide are often swollen, and so turgid with blood that the successive steps of the operation are observed by a rapid oozing of blood with every touch of the knife. The person upon whom we operate is all anxiety, and struggling for breath. In order to avoid the thyroid gland, you must frequently make your incision so low in the neck that it comes upon the sternum, and it will be found often necessary to open the trachea close to the sternum, where it is most deeply situated, and where the surgeon runs the risk of opening the arteria innominata or subclavian vein, or even one of the carotid arteries (the left,) where both arise from the arteria innominata. In young subjects, also, upon whom the practitioner is so often called upon to operate, in cases of croup, the rising of the thymus gland upon the trachea, until it even touches the thyroid gland, in the struggles of the young patient to breathe, also renders this operation very difficult, and demands, upon the part of the operator, the utmost coolness and collection of mind; and when, with all these embarrassments, he knows that anomalous large arterial branches often course in front of the trachea, it will be readily allowed that there is cause for as much anxiety in the performance of this, as of any operation in surgery. But if the practitioner is obliged to perform it at night, the difficulty is greatly increased; for it is impossible, as I have often experienced, to throw the light of a candle into a narrow and deep wound, so as to enable the operator to see the parts it is necessary to divide.

Under all these circumstances, after the first incisions have exposed the dense junction of the muscles in front of the trachea, it is better to lay the latter bare, by scraping with the nail, the director, or any blunt instrument, than to use the knife in a deep wound, obscured by blood, and in the midst of important vessels which you cannot see. When the trachea is rendered sufficiently bare to admit of being seized upon by the double hook, the remainder of the operation, although the most important part of it, is comparatively safe, and may be completed either with the knife or scissors. If with the former, after the trachea is pierced with a sharp-pointed knife, a round piece of it may be cut or scooped out (including that transfixed by the double hook) by means of a straight-buttoned bistoury. If with the scissors, a lozenge-shaped piece may be cut away, as was particularly described in my paper on the subject, inserted in the 4th vol. of the *Transactions of the Association of the College of Physicians.*"

---

### LI.

### Mercury in Chronic Affections of the Larynx.

Dr. Graves makes some remarks on this subject deserving of attention. They are coupled with a case which we shall abridge.

"When hoarseness or loss of voice are of recent occurrence, and evidently connected with laryngeal inflammation; when they are of long standing, but still accompanied by symptoms usually recognized as indicative of local inflammatory action, then few British practitioners will fail to apply the mercurial plan of treatment. But when all the other symptoms of local inflammation have subsided; when there is no soreness, no tenderness of the throat or air-passages; when there is no cough, no dyspnœa; in short, when the lost voice, or a low and feeble whisper, is the only remaing indication of disease, then are we to have recourse to mercury, even though months have elapsed since the disappearance of the symptoms above enumerated!"

*Case.* Miss G. æt. 20, a governess, of strong constitution, caught cold in July, 1831. Hoarseness and sore-throat ensued, but under antiphlogistic treatment she recovered. In September the hoarseness, &c. returned, and were very variable in their appearance. She was treated for hysteria by tonics, &c. In November the symptoms were exasperated. Repeated leechings and small blisters were employed with temporary benefit. In February she had another attack, for which she had again leeches and blisters, followed by calumba and nitro-muriatic acid. She was no better for these measures. In April Dr. Graves saw the case. The young lady was pale, somewhat emaciated and weak—the appetite was gone, the bowels were torpid—no pain or soreness about the larynx, but the voice reduced to a mere whisper—the throat and pharynx apparently relaxed. The application of a solution of nitrate of silver, and Lugol's solution of iodine internally, were prescribed without avail.

In despair she left off medicines. In the middle of August she caught fresh cold, and Dr. G. was again sent for. After venesection and antiphlogistic treatment, Dr. G. gave calomel and opium so as to affect the gums. A rapid improvement ensued, and in five days from the commencement of the mercurial action the hoarseness had disappeared. Bed for a fortnight, and strict silence were enjoined, and the result appears to have been quite successful.

---

### LII.

### The Doctors deceived.

A young woman of nervous temperament appeared to labour under strange symptoms, and excited much curiosity amongst her neighbours, as well as created some surprise in the minds of her medical attendants. Her alvine evacuations were passing strange, and by no means like alvine evacuations in ordinary. Sometimes they contained substances which the surgeon in attendance pronounced to be gall-stones; at another there was seen in them a substance that the physician in attendance conjectured to be the nest of a tape-worm. The matter was of such import, that Drs. Graves and Speer proceeded to investigate the circumstances, and the analytical talents of Dr. Barker, were invoked to bring the aid of chemistry to the inquiry. The gall-stones proved to be pebbles, and the tape-worm's nest was resolved into chewed orange-peel. This interesting young creature had been humbugging the doctors.—*Ibid.*

---

### LIII.

### THE ACTUAL CAUTERY IN VESICO-VAGINAL FISTULA.

WE all know how dreadful a malady this is, how great a boon would be conferred upon humanity by the discovery of a moderately successful plan of treatment. Dr. Kennedy has written a paper in our contemporary of Dublin, with the view of recommending the employment of the actual cautery. It was first introduced to the notice of the profession by Dupuytren, but Dr. Kennedy thinks the description of the manner in which it is used by that able surgeon defective. He therefore describes the manner in which he has usually performed the operation.

" The instruments requisite are, a flat female catheter, two female sounds, a speculum, and cauterising iron. Where the aperture is very high up in the bladder, the speculum I prefer, is the large two-bladed pewter one used by the French; as Weiss's small three-bladed speculum, which answers in ordinary cases, when the lesion is more within our reach, does not, in the former, so well expose the part, or protect the vagina from the iron. Where the opening is in the neck of the bladder, or urethra, the operation may be quite as well performed by dilating and protecting the vagina with curved spatulæ. In an operation of this kind, in which I lately assisted Dr. M'Dowel, three broad brass spatulæ, one introduced towards the perineum, and one at each side of the vagina, exposed the lesion remarkably well: this plan has the advantage of not pushing up the bladder further into the vagina, an inconvenience which it is next to impossible to avoid with the speculum. The cautery should be as nearly as possible the shape of the opening in the bladder, but somewhat larger. That which we shall generally find to answer, is one of an oval shape, with its longer diameter placed transversely. As in the majority of cases, the longest measurement of the aperture is from side to side, and in such the difficulty of affecting an union is greatest; in some cases, however, the aperture has its longest measurement from before backwards, and then our cautery must be similarly constructed. The margin of the cautery ought to be rather more raised than the centre, as our object is to touch the edges of the fistula without injuring the mucous membrane of the bladder.

In applying the cautery we should place the patient lying forwards upon a table, with her limbs hanging over the ends, which should be near a window; elevating the pelvis upon bolsters or blankets placed under it. The limbs should then be separated, and the light thrown as much as possible into the vagina. Where sufficient light cannot in this way be procured, a candle must be used. The speculum is to be introduced, and the lesion brought into view, a flat, female catheter must now be passed through the urethra, and placed across the opening, within the bladder, taking care, at the same time, to reduce any protrusion of the vesical mucous membrane, and retain it out of the reach of the cautery. When the opening into the bladder is very considerable, or the catheter is insufficient, it may be necessary to pass a second instrument through the urethra to effect this object. I have found the introduction of two female sounds answer remarkably well, where a second instrument was necessary. As folds of the vaginal mucous membrane sometimes protrude between the blades of the speculum, the operator must guard against this, and examine whether the instrument be so adjusted as to prevent the vaginal passage being injured by the iron; taking care that the interior of the bladder is well protected, and the edges of the aperture completely within his reach. Having satisfied himself in these respects, he is carefully to introduce the cautery, heated to a white heat, and, having steadily touched the edges of the fistula, to withdraw it and introduce a pledget of lint dipped in cold water, after which he may gradually remove the speculum. The cautery must only touch the part, for if retained too long in contact with it, might produce a sloughing escar.

The operation is extremely simple, and performed in a minute; and although the name of the actual cautery sounds rather harshly, its application is, in my mind, by no means as severe, or attended with such suffering, as the suture. The patient should be put immediately to bed, and strict quiet enjoined. The after-treatment necessary is merely to keep the bowels gently open, and pay attention to the abdomen, lest tenderness should set in; this I have but once known to occur after the cautery; and then it was relieved, immediately, by warm stuping and aperients. After the immediate irritation has subsided, it is necessary to introduce a gum elastic catheter into the urethra, and retain it in the bladder, to insure the urine's passing through it; a precaution the more called for, as in some cases of long standing, this passage has become so collapsed as to be almost impervious. However, in cases where the catheter, on being introduced, passes through the fistula into the vagina, it is better not to leave it in the bladder, else it will do more harm than good. In such it will be sufficient to pass it into the bladder once or twice a day, to endeavour to restore the urethra and bladder to their proper state by bringing the urine in its natural direction. It is necessary that the patient should retain the recumbent posture, and remain perfectly quiet while under treatment; and this point cannot be too much insisted upon. We should also ascertain in what position, namely, whether lying on the back, on one or other side, or the abdomen, she can retain her urine for the greatest length of time, and cause her to remain in that position as much as possible. This has two recommendations; in the first place, it prevents the urine from constantly escaping out of the fistulous aperture, which interferes with the union of its margins, and progress towards a cure; and in the second, by retaining the urine as long as possible, the bladder, which, from being continually empty, had become contracted, is, by this means, gradually distended, and rendered more capable of fulfilling its office of reservoir. By attending in this way to position, and the situation of the fistula, the bladder will be induced to contain a considerable quantity of water, in which it is assisted by the transverse distention observed in it as the effect of pregnancy. It is very desirable, however, with a view to recovery, that the urine should be induced to pass through the urethra, and not by the fistula; and, if the plan here recommended interfere with this, it is better to let the patient assume the position in which the urine escapes through the urethra, if it does so more in one than another.

The effect of the cautery is to produce a thickening of the margins, and consequent contraction and diminution of the aperture, and ultimately an adhesion of its edges, closing it up altogether. Upon the size and position of the aperture will depend the greater or less likelihood of perfect cure.

The operation may require to be several times repeated. Whether by persisting in repeating it sufficiently often, we should, even in the majority of cases, succeed in closing the aperture, I cannot say—but rather think not. Fortunately, however, it does not require that the aperture should be actually closed to enable our patients to retain their urine, as a very good substitute for the adhesion of the sides of the fistula occurs in the extension of its margin or lip across the aperture, thus forming a kind of valvular closure of it, by which means the bladder becomes capable of retaining the urine almost as well as if the opening were closed."

Dr. Kennedy alludes briefly to two cases, in which the circumstance just mentioned occurred. In one, the urine, which before the operation was constantly escaping from the patient, was retained without difficulty after it for six or seven hours. In the other case, the operation was performed six times, and, though the opening did not close completely, the patient could retain her urine during the entire night, and for several hours whilst using exertion during the day. We would recommend a fair trial of the cautery in these cases.

---

### LIV.

### GLOSSITIS.

IN the Number of the Cyclopædia of Practical Medicine for September last, is a short article on Glossitis, which we shall notice. Passing over some general considerations, of no value, we are presented with the following description of the invasion of the disease.

"Idiopathic inflammation of the tongue, or glossitis, is a very rare disease, and very formidable in itself, as well as in reference to its influence on the functions of respiration and deglutition, both of which are in general materially impeded by its presence; the former so much so as to endanger life. It commences with the usual constitutional symptoms common to inflammatory diseases, accompanied with some uneasiness of deglutition; the tongue is rendered painful, and the patient sensible of its enlargement, which is evident on inspection: its surface, at first very red, soon becomes coated, except at the tip and lateral margins, with viscid whitish mucus; the articulation is indistinct, and any attempt to move the organ, or pressure upon it, increases pain: the saliva appears to be profusely secreted, but the inability and disinclination of the patient to remove it from the mouth accounts, in a great measure, for the accumulation and dribbling which are always going on. The local pain increases with the progress of the swelling, which is very rapid; speech and the natural motions of the tongue are consequently more and more difficultly performed; and the augmented bulk, encroaching posteriorly on the space assigned to the passage of air and nutriment, increases the difficulties of respiration and deglutition. The pressure also is a source of irritation to the larynx, and occasions a cough, which under the circumstances of the disease is peculiarly harassing; and the cavity of the mouth being too small to contain the tongue in its increased volume, the organ is consequently protruded. In this state it is obvious that a mechanical impediment must exist to the free course of the blood to and from the head; and from this cause there takes place a throbbing of the arteries, an undulatory motion in the jugular veins, lividity of the complexion, an unnatural prominence of the eyeballs, altogether occasioning an appearance of fulness of the face similar to that consequent to strangulation from any other cause: the accompanying sensations are pain of head, and generally in the ears, vertigo, sometimes indistinct vision, and confusion of mind, or even delirium; considerable pain is also often experienced in the tract of the spinal cord and parts adjacent, from the cervix downwards."

The constitutional symptoms correspond with the progress of the complaint, being those of acute local inflammation in the first instance, and afterwards partaking of the united characters of irritability and exhaustion Sometimes the inflammation is confined to one-half of the tongue, the raphé bounding it. The constitutional symptoms are then milder, and they are also modified, of course, by the circumstances of age, sex, &c. Glossitis terminates in resolution, suppuration, &c. like other inflammations. When the inflammation of the mucous membrane has been excessive, an expansion of lymph has been formed, as in croup. A case of this kind has been recorded by Frank, who refers to a preparation of a similar result of glossitis in the museum of Hunter. The period during which glossitis continues will, of course, vary, but resolution seldom takes place before the fifth or sixth day. The local and general symptoms progressively pass away. The following case is transcribed by Mr. Kerr, the writer of the article, from Frank.

*Case.* "A healthy youth, nineteen years of age, was suddenly attacked with febrile symptoms, together with pain in the head and throat, difficulty of deglutition, and cough: these having been neglected, increased, and during the night he experienced very acute pain at the end of the tongue, increasing in extent and severity with its progressive swelling, which was rapid and considerable, filling the whole cavity of the mouth, and rendering him unable to articulate. The following day he complained of pain in the head, especially towards the forehead, with increase of sensibility of the eye to the impression of light; the tongue was remarkable for its red colour, increase of size, rigidity and heat; the patient could neither draw it inwards nor extend it; the sublingual glands and tonsils were tumefied; he was incapable of speech and deglutition; complained of great thirst, had a dry burning skin, and a frequent strong pulse. Copious perspiration of a sour odour came on in the night, the swelling of the tongue and tonsils subsided, and with it the febrile symptoms; the tongue became moist, deglutition easy, and the following day restoration to health seemed to be established."

Suppuration is attended with the usual constitutional symptoms attending that termination of inflammation. If the pus is deeply imbedded in the substance of the organ, no relief takes place; because, there being little cellular membrane, the included pus exercises much pressure, and is not diffused. In this, as in subfascial suppuration, no time should be lost in giving exit to the matter. Gangrene is a very rare consequence of glossitis, and has only happened in very debilitated constitutions. It has been observed that the separation of the mortified from the living parts is particularly rapid.

"Idiopathic glossitis must at all times be considered a very formidable disease, and the degree of danger, in a previously healthy subject, will be proportionate to the obstacle which the tumefied organ may present to respiration, and to the opportunity which may be offered of subduing the inflammation on which it depends. From active treatment in the early stage, a favourable issue may reasonably be anticipated, particularly if a mitigation of symptoms is seen to follow the successive application of remedial means; but if the disease be neglected in the early stage, or the volume of the tongue increase, in resistance to the measures resorted to, respiration will be performed with proportionably greater difficulty, threatening extreme danger to life by suffocation; and in persons predisposed to apoplexy, or other cerebral disease, in an additional degree, by the impediment occasioned to the free return of blood from the head, and the consequent aggravation of these diseases. Diminution in the volume of the tongue, whether by artificial means directly applied, or through the medium of the system, will proportionately subtract from the danger and increase the rational hopes of recovery; but if the inflammation should have proceeded to gangrene, the danger to life will be influenced by the probability presented by constitutional circumstances of arresting its progress, and, when effected, by the extent of the mortified part: the tongue being the organ of taste, and necessary to the perfection of speech, of mastication, and of deglutition, these functions will be affected commensurately with the local destruction."

With respect to treatment, we need scarcely say that it should be actively antiphlogistic in principle, and we leave it to the practitioner to supply the detail; his judgment must seize, combine, or separate general and local bleeding —purgatives and stimulating enemata —diaphoretics—pediluvia, &c. After leeching, a piece of ice in the mouth is recommended—as is a blister early applied round the throat. But there is a powerful means of relief yet unmentioned, we allude to free and deep scarifications, made from the base to the apex of the tongue, but clear of the ranine arteries.

"Several instances of the advantage of incisions in extreme enlargements of the tongue have been transmitted to us by M. de la Malle.* Camerarius has recorded a case in which the patient was rescued from impending death by this operation; and Zacutus Lusitanus, another of a child, ten years of age, where the usual remedies had failed of affording relief, and the symptoms yielded to deep scarifications. Job a Meckoen, a Dutch surgeon, who lived in the seventeenth century,† adopted this practice on several occasions with the most complete success; and it is probable, as Mr. Samuel Cooper has remarked, that a fatal issue from suffocation, consequent to various kinds of enlargement of the tongue, might in many instances have been averted by its timely adoption. In the twenty-eighth volume of the Edinburgh Medical and Surgical Journal, page 77, an interesting case of the disease is recorded, in which the free use of the scalpel was attended with the best effects; allowing an exit for puriform matter. In the twenty-first volume of the same work, page 135, there is another case, illustrative of the advantage of incisions of the tongue, in a case of its inflammation, apparently consequent to suppression of the menstrual discharge from exposure to cold."

Incisions, however, have failed. A case in which they did so occurred at the Winchester Hospital, and is related in the Lancet, Vol. II. for 1827. When incisions fail, bronchotomy yet offers a chance of life. A successful case of this description is recorded Mr. Benjamin Bell. Mr. Kerr observes that Dessault would have preferred the introduction of an elastic gum catheter from the nose into the trachea. We conceive that, were this actually necessary, it would be objectionable on account of the extreme irritability of the parts. It might be tried. When suppuration has occurred, the pus should be freely let out with a lancet or scalpel. A deep incision may be sometimes necessary. In a case reported in the Glasgow Journal, by Mr. Orgill, two incisions, half an inch deep, were made, from as far back as the scalpel could be made to reach to the tip of the tongue. In the evening still deeper scarifications were made, and on the next day, the tip of the tongue being livid, an incision an inch deep was made with a scalpel, and a gush of matter took place. In eight days the patient was well.

After the evacuation of the pus, Mr. Kerr recommends the employment of merely a gargle of honey and barley-water, though it may sometimes be necessary to resort to astringents and detergents.

---

### LV.

### ENORMOUS TUMOR ATTACHED TO THE ILIUM.

CHRISTIAN MASSON, aged 25, unmarried, about 14 years ago had a very severe fall upon the ice, by which her left hip was cut and contused. Soon after a small tumour made its appearance in the seat of the injury, and gradually acquired the enormous bulk undernoted. It originates at the lower half of the left os ilium, going rather farther back than the great trochanter of the left thigh; it is firmly attached by its neck as far forward as the symphisis pubis; it proceeds downwards in front of the thigh (the attachment being in the line of the rectus muscle), until about three inches above the linea. It spreads out (being of an enormous bulk) until it ends behind the great trochanter, as formerly stated, all this part being, comparatively speaking, free and loose, so that, when seated on a chair of ordinary height, the bulky part of the tumor more than touches the floor. I find that, at its upper attachment, it measures in length two feet; that a line drawn from the centre, at the upper middle part of the os ilium,

* Mém. de l'Acad. de Chirurgie, vol. v.

† Dict. des Sciences Méd. Art. *Glossite*.

and carried round the tumor at this place, measures 3 ft. 5 in., and that a line drawn from its attachment at the great trochanter, and carried round its outer edge all the way, measures 7 ft. 5 in. The tumour is probably steatomatous, but apparently containing matter of every degree of consistence, from the most condensed fat to the softest pappy or lardaceous substance; its surface is traversed in all directions by tortuous vessels, some of a very large size, and there is a great cleft towards the inner and middle part of it, which she ascribes to its striking on the other thigh and knee when she was able to walk. She is now unable to move, and with difficulty turned in bed; she does not complain of much pain, but of great itching, and a sensation as if insects were moving through the tumor; it seems to incommode her most by its bulk and weight. Her general health is now very indifferent; her pulse quick and feeble; she is affected with almost constant dyspnœa; her face sallow: left leg and foot anasarcous; yet her appetite is pretty good, and her bowels regular. The menses finally ceased about a year ago.

I have often been solicited by the woman and her friends to remove this tumor (which I conceive cannot weigh less than 100 lbs.) by operation, but always declined, from dread of the event; which dread has been increased by a knowledge of the fate of Hoo Loo, the Chinese, operated upon in Guy's Hospital last Summer.

J. Bell.

*Forres, Aug. 22d,* 1832.

We perfectly coincide with Mr. Bell, in his repugnance to operating on this poor woman. When we consider the great size of the tumor at its attachment, the large vessels supplying it, the age of the patient, and her state of health, we think the chances of recovery from an operation so very slight, as to preclude any prudent man from employing the knife. It is true that the woman must die, if the tumor be allowed to remain; but when we consider that she has attained the age of 52, and reflect that all unsuccessful operations bring a certain degree of discredit on surgery, and perhaps deter patients from submitting to the knife when they might really be benefited by it, we must again express our decided conviction of the judgment and propriety of Mr. Bell's determination.

---

### LVI.

### Mr. Crampton on Inflammation of the Brain and its Membranes.

In the Number of the Dublin Journal for September, were some valuable observations, by Mr. Crampton, upon Injuries of the Head. We had the pleasure of introducing those observations to the notice of our readers, and we feel gratified by observing a continuation of them, in the Number of the same able Journal for November. We trust that this will not be the last, as it has not been the first, occasion for expressing our approbation of the candid manner in which Mr. Crampton lays the results of his extensive and varied experience before the public. Would that the generous example were more widely followed than it is.

The subject of Mr. Crampton's present paper is acute inflammation of the brain. The subject is one of equal interest to surgeon and physician, too often one of equal difficulty. Mr. Crampton is one of that practical class of surgeons, who look upon inflammation of the brain as inflammation of the brain, and while they admire the bold and clever generalizations of the French, lament that *they* cannot find them confirmed by Nature. In other words, Mr. Crampton agrees with those, who acknowledge themselves unable to distinguish with nicety between inflammation of the substance and inflammation of the meninges of the brain. Accordingly, by the term, inffammation of the brain, he implies inflammation of the parts included within the encephalon generally. Premising thus much, we may pass on.

Mr. Crampton's object in the present paper, if we rightly understand it, is rather to shew that Nature does not altogether support our current notions, than to establish, by arranged facts, any previously-admitted principle, or to build, on an inductive basis, a new one. We will give an abridgment of Mr. Crampton's cases, and then our readers may judge for themselves.

*Case* 1. Mr. J. Cooper, æt. 17, strong and healthy, indulged more than usual in gymnastic exercises on the 11th March, 1832. On the following day he went to a heated church, and subsequently dined out. At midnight he awoke with an excruciating pain in the left eye-ball, and felt as though the eye must burst. Next morning he was seen by a gentleman, who ordered him some purgative medicine. The pain was severe throughout the day, but was relieved in the evening after the operation of the physic. During the night it returned, and next morning he requested the application of leeches. Twelve were applied, but they had scarcely fallen off when the pain became more violent—he raved—imagined that the gymnastic pole was pressing against his back—and sometimes almost screamed with agony. At 6, p. m. he was seen by Mr. Crampton, who found him thus:

"He was lying on his right side, his eyes closed, his mouth half open, and his head thrown backwards; his face was deadly pale, (the leech-holes were still bleeding,) the breathing not hurried, but he moaned almost incessantly, like one who is suffering in his sleep. Sometimes he cried out with a voice of agony, 'Oh dear, oh dear, what shall I do?' and then muttered indistinctly. When roused, however, he was perfectly rational, but was impatient of being questioned; sometimes, when spoken to, tossing himself to the far side of the bed, and hiding his head in the bed-clothes. On being questioned, he said, 'the pain had left his eye, and had settled in the back of the neck,' where the slightest pressure seemed to cause severe pain. In short, his whole aspect and manner was that of a person who had received a severe blow or fall on the head, and who was beginning to awake from the deep coma of *concussion*. The pulse was 84, soft and regular while he remained at rest, *but it rose to* 120 *when he moved, or was raised in the bed*, establishing another remarkable resemblance in the symptoms to those attendant on concussion; skin hot and moist. Bowels had been moved three times; urine natural. Twenty ounces of blood were ordered to be taken from the back of the neck by cupping; two grains of calomel every hour; a cold lotion to the shaved scalp."

Next morning, at 9 a. m. Mr. Colles was added to the consultation. He was much the same, but had some appearance of collapse. He had raved much during the night and was much more comatose, but he could be roused when spoken to loudly, and then conversed rationally though reluctantly. Pulse 100, soft, regular, more frequent on motion—hearing obviously impaired—his mother thought he was blind—pupils fixed, of medium size. *Forty leeches to the forehead and temples—blister to the nucha. P. c. cal. et pulv. Dov.* At 6, p. m. deep coma — perspiration — pulse 140, small. At 10, p. m. respirations 70 in the minute and stertorous —frothy mucus flowing from the mouth —radial pulse extinct. At 9, a. m. of the following morning, 80 hours from the commencement of the pain in the eye, he died.

"*Inspection* 22 *hours after death.* On raising the dura mater, the whole surface of the hemispheres appeared of a pale greenish colour, in consequence of the effusion of a thick green-coloured pus beneath the arachnoid: that membrane preserved its normal structure and transparency, but the pia mater was highly vascular, and its surface flocculent; the more fluid purulent matter was intermixed with portions of a greenish matter of some consistence, which might be called adventitious membrane; this substance chiefly followed the course of the sulci which separate the convolutions, dipping between them to the depth of half an inch. The chief seats of the purulent effusion were the inferior surface of the anterior lobes of the cerebrum, the whole of the base of the brain and the cerebellum, but especially the pons and medulla oblongata. On squeezing the pituitary gland, two or three drops of purulent matter escaped through the cut extremity of the infundibulum; the brain itself was of a natural colour and consistence, but appeared to me more vascular than in the healthy state; there were about two ounces of limpid fluid in the ventricles; the choroid plexus was unusually pale."

Mr. Crampton thinks the following circumstances most worthy of notice.

"1st.—The absence of '*delirium ferox*,' *convulsions*, or *paralysis*, either spasmodic or atonic symptoms, which are described by nosologists as essentially characterizing inflammation of the brain, or its membranes.

2nd.—The resemblance which the whole assemblage of symptoms presents to those which are usually thought to denote concussion of the brain, from external violence."

We bow to the authority of Mr. Crampton, and yet we would fain persuade ourselves that the assemblage of symptoms, as recorded, does differ in some important respects from those thought to denote concussion. In this case there was excruciating pain in the head, only obscured by obscured sensibility—on the second day there was raving and delirium, the patient imagining himself pressed and injured by what was not near him—and the skin was hot. On the third day still delirium—coma—impaired hearing—probable blindness—fixed pupils. In the evening deep coma and stertor, with the pulse 140. On the following morning death. We have not seen a tithe of the cases which Mr. Crampton must have witnessed, but certainly we have seen no case of mere concussion like this, nor, so far as we can judge, do the ordinary descriptions of concussion tally with it. Such symptoms *after* concussion indeed, we have seen, and have seen them prove fatal; but they marked inflammation of the brain, and such has been found on dissection. In the present instance we cannot but express our surprise as well as our regret, that so little should have been done by the gentleman in attendance previously to Mr. Crampton. It is surely not good practice to allow most intense and fixed pain in any part of the head to remain hour after hour without a recurrence to local or general depletion.

*Case* 2. Mark Moore, æt. 10, an intelligent and delicate boy was attacked while travelling in Wales, with intense pain in the head, accompanied by vomiting and somnolency. Mr. Crampton saw him fifteen hours after the commencement of the attack.

"He lay on his side, with his head thrown back and his eyes half closed; the face flushed, but without a circumscribed patch of red on the cheek; he moaned almost incessantly, sometimes shrieked, started up on his seat, looked about wildly, and then tossed himself to the opposite side of the bed. Pulse 120; skin hot, but moist; tongue white in the middle and bright red at the edges; stomach irritable; bowels constipated." "It appeared to me that I had to deal with an acute inflammation of the brain, and I took my measures accordingly. I opened, on the instant, the jugular vein, and took away at least twelve ounces of blood; twenty leeches were applied to the forehead and an iced lotion to the shaved head; two grains of calomel and one of Dover's powder every two hours; iced barley-water for nourishment. The symptoms were completely relieved for six hours, but returned, at the expiration of that period, with increased violence. The bleeding was repeated at midnight, and leeches were applied in relays, twenty at a time, so as to keep up a continued flow of blood from the skin of the head. The inflammatory symptoms, accompanied with delirium, recurred five successive times, at the intervals of 6, 5, 10, 11, and 14 hours; the bleeding was repeated on each of these occasions, and on the eve of the fourth day the system was brought fully under the influence of mercury. The recovery was rapid and complete. The patient is now, after an interval of eighteen years, in the enjoyment of perfect health. I had the advantage of Doctor Cheyne's valuable assistance in the treatment of this important case, after the second day."

Here was a case really similar in many respects to the preceding, and, beyond question, one of cerebral inflammation. It would puzzle us to point out much resemblance between the features of such a case and those of concussion. Mr. Crampton acted promptly and boldly, and the result displays in a striking manner the beneficial consequences of early and judicious vigour in the treatment of these dangerous forms of disease.

In Mr. Crampton's preceding communication he related the case of Fagan, a pipe-maker, which case we detailed in the last number of this Journal, at page 512, et seq. Circumstances worthy of record have since occurred, and Mr. Crampton resumes his report in the present paper. In order that our readers may understand Mr. C. we will recapitulate a few of the previous particulars of the case, referring our readers for the details to our last number. The man was wounded in the head with a dragoon's sword, for dragoons, in Ireland, appear to take great liberties. The skull was fractured, the membranes wounded, and the brain protruded. On the eighth day he was attacked with convulsions followed by stupor. A portion of the bone was removed by Hey's saw. The convulsions gradually passed away, but fungus cerebri appeared on the 10th day. In 24 days this had disappeared, and in 11 days after this the wound was healed. In a fortnight more Fagan was discharged and resumed his employment. He was unable to remember the names of things. At this point the last report ended.

After this man was discharged he led a very irregular life, suffering after each debauch from severe pain in the head. On the 22d of August (he was discharged on the 15th May) he nearly lost all power in the right arm and hand, and the right side of the face was paralyzed. On the 24th he was re-admitted.

"The following statement is abridged from the hospital journal: John Fagan, re-admitted August 24th, complaining of severe pain in the seat of the original wound; and although his head pain is not constant, the paroxysms recur several times in an hour, and last for two or three minutes; vomits occasionally; vision indistinct; pupils dilated, and very sluggish; strength and sensibility of the right arm and leg much diminished; pulse 100, soft and easily compressible; tongue clean; bowels free; memory very defective, particularly with respect to names and recent events; but the defect is not confined to the faculty of memory, as, with few exceptions, he cannot repeat proper names, but miscals almost every thing; although he can perfectly describe the use of it, he calls, for instance, a watch, a gate; a book, a pipe, &c.; a pipe is the word that he pronounces most frequently; it is remarkable, however, that the moment he employs a wrong word he is conscious of his mistake, and is most anxious to correct it. The cicatrix of the wound, which is six inches long, and half an inch broad, is *raised*, particularly at its centre, above the level of the scalp; it is of a purplish red colour, tense, and shining, very painful to the touch; and at the centre, which is the softest and most prominent part, there is a strong pulsation, obviously synchronous with the radial pulse.

26th. Yesterday had several severe paroxysms of pain, accompanied with grinding of the teeth and contortions of the features, and succeeded by complete insensibility, which lasted for five or six minutes, during which time the pulse fell to 50 in a minute. Twenty leeches were applied round the cicatrix, a blister to the nape of the neck, and a cold lotion to the head; purgative pills.

27th. No return of paroxysms; pain relieved.

28th. Several paroxysms of convulsion, followed by stupor; cicatrix more tense and red, but the fluid which it covers disappears on pressure, and returns when the pressure is removed; pulse 72, and regular; tongue foul; bowels open. Continued to improve; paroxysms becoming less frequent until the 4th of September, when he had violent vomiting followed by convulsion, after which he remained insensible for several hours; pupils dilated; pulse 54; respiration natural; a small opening was made into the prominent part of the cicatrix, and two drachms of healthy pus were discharged ; *the pulse immediately rose to* 68 ; *he sat up in the bed, answered questions rationally, and said he was quite free from pain.*

7th. Continued free from pain or convulsion ; the little opening is healed, and the tumour is as large as before ; a larger opening was made into it, and a small quantity (about half a drachm) of bloody serum was discharged.

Oct. 9th. Has had no pain or convulsion since the 4th of September, when the abscess was opened ; he appears in perfect bodily health, with the exception of some remaining weakness in the right arm and hand, and some slight confusion of vision ; the cicatrix is perfectly on a level with the head, and there is no sensible pulsation in the seat of the former abscess ; the mental phenomena are as before described, and are most remarkable ; he speaks correctly, and even fluently ; describes his sensations with great clearness, but avoids all proper names, he says, (for example,) 'I have a great weakness and numbness here,' (pointing to his shoulder,) 'and along here,' (drawing his finger along the arm to the palm of the hand ; 'but no pain.' 'When I sit up suddenly I don't see rightly ; but I soon see as well as ever.' He counted 5 on his fingers ; but could not say the word 'finger,' though he made many attempts to do so. He called his thumb, 'friend.' When desired to say 'stirabout,' he said, and invariably says, 'buttermilk ;' but was immediately conscious of his error, and said, 'I know that's not the name of it.' Sometimes (as in the last instance) one could trace the association of ideas through which he was led to the misnomer: stirabout and buttermilk being associated in the mind of every man of his class in this country ; but in the greater number of instances, no such association could be traced ; but this should excite no surprise, as the disturbing cause, which was of sufficient force to dissociate the idea of the *name* from the *thing*, would, naturally enough, be sufficient to disturb the faculty of 'association.' Generally, these experiments, if they may be so called, were performed in the presence of Dr. Marsh, and a great number of the pupils of the hospital."

On these cases Mr. Crampton makes tbe following remarks.

"Considered separately, these cases possess but slight claims to attention, either on the ground of their singularity or of their furnishing any new views with respect to the pathology or treatment, of diseases and injuries of the brain. It appears to me, however, that when viewed in connexion with one another, they acquire some degree of importance. By placing a case of acute inflammation of the brain arising from external violence, and therefore probably complicated with some other organic lesion, beside a case of acute inflammation, arising idiopathically, we are the better able to compare the symptoms of each with the other, and separate those which are the proper signs of inflammation from those which belong either to *concussion* or *compression* of the brain.

In the instance of Fagan, (for example,) the symptoms in the early part of the case, though less acute, were of the same character as those which occurred in the case of Mr. Cowper; we had in both violent pain of the head, great nervous irritability, followed by stupor, and that peculiar kind of delirium in which the patient seems like one talking in their sleep, from which, however, they can be roused into perfect, though transient rationality. In both the pulse was quick, and the skin hot. In the case of Mr. Cowper, the inflammation terminated in *the effusion of purulent matter, generally on the surface of the brain, and of serous fluid into the ventricles,* and this was attended with blindness, deafness, and derangement of the functions of life generally; but there were neither convulsions nor paralysis. In the case of Fagan, the inflammation terminated in *circumscribed abscess on the surface, or within* the substance of the brain, and *then* appeared the symptoms which denote *irritation* and *compression* of that organ : namely, convulsion, followed by stupor, paralysis of the side opposite to that on which the injury has been inflicted, destruction or suspension of one or more of the intellectual functions. That these were the proper signs of *compression,* (as distinguished from *inflammation* or *concussion,*) appears from the fact, that the moment the compressing cause was removed by the evacuation of the matter, the symptoms disappeared.

Should these views be confirmed by numerous observations collected from various and independent sources, one important step will have been made towards elucidating the pathology of the brain."

It will be observed, that we do not much condense cases of this description with the practical remarks appended to them. We look upon them as written cliniques, the advantage consisting in the discrimination of minute features, and comments on the subtler phenomena of disease. To fuse these into a consistent mass, would evidently be to destroy them. In conclusion, we trust that Mr. Crampton will continue to favor the profession with such cases and such observations, and perhaps he will pardon us for again hinting, that the greatest service he can render the state is by communicating facts, digested, arranged, compared, and reasoned on. A man of Mr. Crampton's surgical calibre *must,* now he is embarked in the publication of his experience, go on. He and his coadjutors, Mr. Colles and the rest, must recollect that Ireland is looked upon as slothful. Like Sampson, they have the power of the strong man; —but why, like Sampson, should they sink before the Dalilah of indolence?

By the way, Mr. Crampton explains away an expression in his last communication, on which we took the liberty of commenting. The explanation in question is perfectly satisfactory, and we beg to thank Mr. C. for the handsome compliment which he pays us. None can entertain a more high opinion than we do of the Irish surgeons, and it is because we wish them well that we stimulate them thus.

Macte virtute.

---

### LVII.

THE ELEMENTS OF ANATOMY. By JONES QUAIN, B.M. Professor of Anatomy and Physiology in the University of London. Second Edition, 8vo, pp. 812. John Taylor, London, 1832.

WE would fain persuade ourselves that a new æra is dawning on medical education in this country. Within these few years a revolution has taken place in our schools, and we do not yet perceive its ultimate consequences, though the shadow of coming events may possibly be apparent. It is especially in the teaching of anatomy that we anticipate great changes. In 1828, that excellent anatomist of Edinburgh, Mr Knox, published his translation of Cloquet's System of Anatomy. Much about the same time there appeared the first edition of the present work of Mr. Quain; and Mr. Bransby Cooper's Anatomy is but just completed. It would be invidious in us to compare these works, but we may safely assert that each is possessed of merits of a high order, and in former days would certainly have conferred upon its author a degree of reputation, that modern intelligence and modern competition must necessarily conspire to diminish.

There is one leading feature in which M. Cloquet's volume differs from both Mr. Quain's and Mr. Cooper's. It is strictly confined to descriptive anatomy, and contains little general anatomy, and no references to the modes of performing surgical operations. We must say that, on the whole, we feel disposed to prefer this method, and we imagine that it is better to leave the directions for tying arteries, and performing other surgical operations to works especially devoted to their consideration. By this separation either subject is considered more fully, rigorously, and systematically, and a taste for minute observation and precise reasoning is consequently generated in the student. It is fair to state that persons well acquainted with the teaching of anatomy think differently, and it must be allowed that the means of many are so scant, as to make them of necessity prefer those works which convey the most general information.

Differences then exist in the plan on which anatominal systems are at present constructed, but descriptive and general anatomy are more carefully taught in all. Whether we look to the pages of Mr. Quain, Mr. Cooper, or M. Cloquet, we find a degree of precision and minuteness in their descriptions, which reflect great credit on the present state of anatomical science. It would not be difficult to shew that this is mainly, if not altogether, owing to continental influence, and that the exact and methodic character of French anatomists has modified the rambling and discursive manner of our own. Two of the systems of anatomy to which we have made such frequent reference, have been greatly indebted to those of Meckel, Boyer, and Cloquet. Of this there can be no doubt, and we repeat that the improvements which seem to us to be now taking place in anatomical instruction in Great Britain, have their source in the schools of France and Germany.

The most opportune occurrence for medical science in this country is the passing of the anatomical act. It is a lever of which we can scarcely estimate the power. Even now its advantages are apparent in the great schools, and when it comes fairly into operation, and works, as no doubt it ultimately will do, well, we anticipate a new order of students and practitioners. Anatomy will then be taught from the body, and the profession will no longer be scandalized, by seeing crowds of young men learn just enough from plates and the diagram board, to get through the examination of the College of Surgeons. But this is not all, it is but the beginning of the end. We do not pretend to the gift of prophecy, but we feel assured that before many years have passed away, great changes will occur in other departments of medical education.

A sufficient proof of the popularity of Mr. Quain's book may be found in the fact of a second edition being called for thus early. This edition is much augmented in size, and, what is better, proportionately increased in value. Having formerly noticed the first edition, we need not again display the plan adopted by Mr. Quain, but we will advert to a passage or two in his prefatory address to the reader. Mr. Quain has asked, "what is the true method of study?" and thus he proceeds to reply.

"The objects of study, from their very nature, must present themselves in different points of view, and require different methods of investigation. Thus you may consider the situation, form, and size, of a given organ, and then its relations to contiguous parts. When you have stated all the facts relative to these points, you have given a description of the part, inasmuch as you have followed the *descriptive method;* and this is all that is usually done in works on Descriptive Anatomy; they give merely the topography of parts. It must be obvious that it is not sufficient for our purposes thus to confine attention to the surface of things; our inquiries are not to be limited to external qualities, or mere relations of place. We desire to become acquainted with the composition and structure of the human frame, or, in other words, with the elements of which it is made up. With this view, we resolve it into its constituents, and then examine the character and properties of each of these separately, as a necessary preliminary to a just appreciation of their powers when combined. This is the *analytical method.* Its application and use have been already pointed out in the remarks on General Anatomy.

As the phenomena included in a particular function, or in the derangement of it, do not present themselves to us at once, but occur in succession, it becomes necessary not merely to enumerate them, but to set them down in the exact order of their occurrence. When treating of the digestive function, for instance, we have to consider the mechanism employed in the prehension and mastication of food, its impregnation with saliva, its deglutition and conveyance to the stomach, the changes which the mass undergoes in that viscus, and afterwards successively as it passes, step by step, through the different parts of the alimentary canal. When these particulars are fully stated, the narrative is complete, and we have conformed to what is termed the *historical method.*

We cannot, however, confine ourselves to a mere statement of facts, or an enumeration of events. The very constitution of our minds compels us to draw inferences from the facts we have observed: we cannot help thinking, and to think is to theorise. This we at once recognise as the starting-place of all the speculative views and visionary opinions which the history of medicine records; and, unhappily, they are but too numerous: some of them evidently flow from the disposition so constantly manifested to deduce general principles from inadequate data; others are referable to that proneness which persons evince, when entering on speculations concerning the phenomena of life and the functions of living beings, to carry with them, and even rigorously apply, notions and principles taken from such pursuits as had previously, and perhaps exclusively, engaged their attention. Hence it is that the Philosophers of old introduced into medicine their peculiar hypotheses, the Heathen priests tinctured it with their superstitious rites; whilst, in more modern times, the Mechanists sought to explain the functions of the body in health, and its derangements in disease, by principles deduced from hydraulics, and the Chemists referred them to the affinities which govern the processes they were wont to observe in their laboratories." xi.

After quoting from the Novum Organum of Lord Bacon a short exposition of the inductive method, introduced by that greatest of revolutionists, Mr. Quain continues thus:—

"This is the *inductive method*, as taught by Bacon. To observe patiently, experiment cautiously, and generalise slowly, are the precepts, it enjoins for the guidance of our own researches, and the tests which it suggests for estimating the value of the opinions and researches of others. The bias of prepossession and the influence of authority have for too long a time led away the mind from a conformity with its precepts: but happily these disturbing causes have now nearly passed away; for in our schools no general theory is taught—no uncompromising dogma is inculcated—no individual, however eminent he may be, can draw after him a crowd of followers ready to take his dictum as law, and resolved, when they set forward in life, to make their practice square with his injunctions; in a word, there no longer exists a monarchy in medicine; and, were we to look back to the history of those times in which the men of that profession were little else than the obsequious followers of a few distinguished individuals, we should find little reason to regret that their dynasty is at an end. And let not this excite surprise or regret—it should rather be a ground of satisfaction and gratulation, inasmuch as it has arisen, not from any causes tending to depress the few, but from the wide spread of knowledge, which has tended to elevate the many. There never was a period in the history of medicine in which there was less to discourage inquiry than the present—there never was a time in which so many circumstances conspired to invite a scrutiny into all its departments. The mind is no longer prostrated by the domination of authority, nor is reason warped by the influence of system. The errors of preceding inquirers are so many beacons to warn those who succeed from straying into the devious tracts into which they wandered, and the failure of their methods of investigation points to the necessity of pursuing a different line of research from that which they adopted. Speculation and hypothesis have reigned too long; it has been too much the practice of system-makers to construct their edifices, if I may so say, 'from their own conceptions, and to draw from their own minds all the materials they employed.' Whilst notoriety, perhaps even distinction, could be attained by such compendious methods, we cannot feel surprise at the number of hypotheses which the history of medicine records, or at the fleeting credit they maintained. One heresy gave way to another, and a third succeeded as short-lived as either of its predecessors. But, even at the present day, it is not a little difficult to repress speculation altogether, and confine inquiry within its legitimate bounds."

No observations can be more true than the preceding. Henceforward there is no monarchy in surgery or medicine, henceforward we are a republic, one and indivisible. There will always indeed be men who will excel their fellow men, but their superiority will not be one of mere talent over ignorance, but of great and solid attainments over less. We might shew that this is the case with surgery at present, and the man who now bears the most deservedly high reputation in this department of our profession, is one who has worked indefatigably in the study of facts, and proceeded as philosophically as cautiously in his deductions from them.

---

### LVIII.

### What is the Contagion of Cholera?

Such is the question that Dr. Kennedy of Ashby-de-la-Zouch, an able, learned, and practical physician, asked himself in a lecture delivered in the assembly rooms of that town. And how did Dr. Kennedy answer it? Did he say it was pestilent, furious, dire, and unsparing—that it ought to unite doctors of all degrees in owning it—journals of every colour in arointing it—that it ought to fright the isle from its propriety—shut the theatres and the hospitals and open the churches—that it ought to put our country in quarantine, and our parishes in cordons—that it ought, as the writer in the Quarterly recommended, to make families bar their doors, and import their provisions in iron buckets through the windows—or, that, owing to its presence, districts should be officially declared infected, and a sort of Italian "immondezzaio" be inscribed upon their outskirts? Dr. Kennedy said nothing of the kind. Well, then, did he laud preventive, we beg pardon, "precautionary" measures—did he think them a benefit to a poor, but *not* a pauperized population—did he weigh the commerce minus against the blankets plus, and find the balance in favour of the blankets—did he look at the bills of mortality, and see in them a salve for theoretic knocks? Oh no. Dr. Kennedy has done no such thing. He has written some sensible remarks upon contagion, which we quote.

"We cannot be required to show, by facts or arguments, that Cholera is *not* contagious, because attempts to prove a *negative* are self-evidently illogical and absurd. Should we, however, find ourselves justified in asserting that this pestilence *is* contagious, then we would necessarily undertake the responsibility of establishing our assertion. Moreover, common sense and science alike reject the assumption of an extraordinary cause for any circumstance or event, when what is ordinary will suffice to account for the results. Now, is it probable, is it possible, that the malignant principle of Cholera can be generated by other causes than contagion? You may reply in the affimative without suspense of opinion, if the *state*—predisposition—be conceded; and, you may reply in the affirmative with confidence, because this fact rests on the most perfect demonstration—that many spontaneous and fatal explosions of Cholera have occurred in remote hamlets and cottages, whose inhabitants enjoyed complete exemption from intercourse with those of other places where the disease was prevalent. When, out of several persons all maintaining the same intimate and frequent communication with the sick of Cholera, and equally exposed to the influence of its known and reputed causes, one man alone of the entire group sustains an attack of the disease, it is the fashion to account for such a seizure by declaring authoritatively—that, among the whole of his associates, the seized One exclusively was in a state of *predisposition*, an imaginary state, the reality of which is altogether incapable of proof, by inference, analogy, or experiment. Many persons are represented as having been infected with Cholera, chiefly in consequence of their predisposition to imbibe its elements; but, we look in vain for any thing resembling an attempt to show what this predisposition *is*—to prove, that predisposition was then existent. We must acknowledge, indeed, that very few of such patients exhibited discernible signs of difference between themselves and others who were more fortunate in resisting equal danger: besides, very few of these sufferers exhibited discernible signs indicating—that, at the time of being seized, any one of their own conditions was at all different from what it had been, for days or weeks, previously to the hour of their calamity. Many things there are, doubtless, which predispose mankind to sustain the inroads of mortal diseases; but, it is undeniable, that the something which predisposes us *exclusively* to Cholera, and the something which *is* Cholera, are equally and entirely unknown.

They who represent Cholera as an infectious pestilence, have carefully accumulated—as a means of confirming their theory—cases of persons who became ill, soon after having touched or used articles of dress or bed-clothes of those who were suffering or died of the disease,—and cases of persons who became ill, soon after being exposed to the influences of an atmosphere supposed to be impregnated with cholerous exhalations. Now, these cases are not numerous; and, though the whole of them were admitted as fair evidence, without scrutiny, they could reasonably be held for nothing more than so many exceptions to a general rule; and, consequently, they do not invalidate the doctrine which that rule goes to confirm. Rightly estimated, such cases prove this much, and nothing more; that—soon after being in the situation, or doing the things, ascribed to them—persons have been seized with Cholera; but, in these cases, we find no proof whatever that they were cholerous by contagion, or indeed by any other cause: the incident of one condition or action being merely subsequent in time to another of the same kind, can never authorize us philosophically to regard the latter as having been consequent on the former—to regard the two conditions or actions as standing in the relation of cause and effect. Moreover, many medical inquirers, zealous for the advancement of knowledge and the welfare of mankind—have voluntarily and experimentally placed themselves in close contact, in bed, with the dying and the dead of Cholera, have there inspired profusely the breath of their departing patients,—have inoculated themselves with the blood, perspiration, and other fluids of cholerous sufferers,—have endeavoured, in every imaginable way, to impregnate themselves with the infection of this pestilence,—and, with very few exceptions indeed, these adventurous philanthropists sustained no injury from their remarkable experiments. Altogether, in fine, by these and many other facts which tend fairly to strengthen the same position, we are justified in esteeming the process to show that *Cholera* is *contagious*, as a palpable and perfect failure; and, in concluding that Cholera *generally* originates from other causes than contagion."

Dr. Kennedy does not declare cholera absolutely non-contagious, but admits that it may be so under circumstances and with limitations. With this view he agrees with those, who, doubting, act as if it was contagious and use precautions against it. This is a specious doctrine, but may it not be mischievously enforced? In a matter of doubt it is better to act as if the worst were impending, with this proviso, that the measures we employ be not themselves so severe, so injurious as to form a serious addition to the evil itself. Now let us apply this maxim to quarantine. There can be no question that this is a cruel hindrance to commerce, a hindrance so keenly felt that it should not be applied unless there were a fair prospect of benefit accruing from it. What had experience told us before cholera reached these shores? It informed us that in all countries quarantine, much more rigorous than we could enforce, had proved entirely inefficacious. Well, this lesson of experience was disregarded, and what was the consequence?—that cholera arrived, as it had arrived elsewhere, and that in addition to its ills, we had the depression of commerce and stagnation of trade dependent upon quarantine restrictions. In France—in Holland it has been the same. Now look to Spain and Portugal. In Spain quarantine is rigidly enforced, and the priests of despotism in every form, the Camarilla, have sagely, as wiser men in this land of freedom did before them, disregarded all experience, and hoped to keep out cholera by law. In Portugal the force of events has been stronger than the force of prejudice, and the thunder of artillery has choked the cholera cry. Yet, in spite of all the unrestricted communication that exists between infected London and Glasgow, and uninfected Oporto—in spite of the volunteers from our poorer and therefore cholera-loved ranks, that enrol themselves for problematical liberty, and still more problematical pay —in spite of all this, there is yet no cholera at Oporto, whilst the obstinate and well-quarantined Dutchmen are affected with it. It is just a toss up which gets it first—Portugal or Spain. What we mean to say is this—that supposing cholera to be highly contagious, quarantine has been proved, by the experience of Asia, Europe, America, to be useless, and if useless, to be pernicious. We care not, for the sake of the argument, what may be the cause —whether the contagion be too subtle to be restrained by quarantine—or whether so many means of breaking through quarantine exist, that it becomes inefficient in that way—whether in short it be too little for the disease—or whether man by fraud, or force, or gold be too much for it—whether cholera come in by the smuggler, or Jove-like be conveyed in a shower of gold—it matters not whether any of these reasons be valid or none—the general result is against quarantine,—against this the first and last of "precautionary" measures, and practice has eternally damned it, if theory has not.

We hold it then to be a sophism in the case of cholera, to act as if it were contagious, even though we felt inclined to suspect that it were not so.

But there is another sophism that happens to have been applied to cholera, although it is in far more general use. It is a common observation that if one cause be adequate to explain a circumstance in medicine, it is unphilosophical to look for another. No remark can be more shallow, more absurd. Do we not find in life that there are many and very opposite obvious causes of disease? Is pleurisy always brought on by cold? Will not a very high temperature produce inflammation, and will not a very low one do the same? Nature abhors those petty ligatures that fools would fancy, and philosophers would forge, and she will not be trammelled by these logical refinements and scholastic subtleties. Our readers know that we are not of the class that sneer at philosophy, and see in vigour of expression, or exact discuscussion, nothing but rhetorical flourishes or useless refinements. But we would not willingly lay hold of the eel of science by the tail, nor would we substitute the scholastic forms of reasoning, for the substance of rigorous and inductive reasoning itself.

---

### LIX.

### Ligature of the Subclavian Artery for Subclavian Aneurism.

This case is related in the *Lancet*, for Nov. 17, by the operator, Mr. Nicholls, senior surgeon of the Guardian's Dispensary, Norwich.

Early in April last, Miss N. æt. 21, residing near Norwich, consulted him respecting a pulsating tumour in her neck, which had commenced after a sudden exertion of her left arm. "On examination (says he), I found a tumour the size of a hen's egg, situated in the left side, occupying the triangular space which is bounded below by the clavicle, on the inner side by the clavicular portion of the mastoid muscle, and on the outer side by the anterior fibres of the trapezius, evidently the result of some injury done to the subclavian artery, in that part of the canal which stretches from the edge of the scalenus muscle towards the axilla, before it has passed under the clavicle; indeed, so near the edge of the scalenus was the injury, that I was not able to compress the vessel with my thumb, between the muscle and the tumour." Mr. M. ordered rest and some aperient medicine, and, having obtained the young lady's consent, performed the operation of tying the subclavian, immediately external to the scalenus anticus, on the 30th April. As there are some peculiarities in the mode in which the operation was performed, we must give the operator's own description.

"The patient was placed in an horizontal position on a table about three feet in height, having her head hanging over the end, and supported by an assistant. The integuments being drawn down, an incision was then made through the skin and platysma myoides, along the clavicle, three inches in length, from the outer fibres of the clavicular portion of the sterno-cleido-mastoideus, outwards: another incision was carried from the inner point of the former one, upwards along the clavicular portion of the sterno-cleido-mastoideus for four inches; this incision passed between the fasciculi of the platysma myoides, which, in this case, were remarkably large. The triangular flap formed by the two incisions was dissected back, carrying with it, imbedded in its substance, the external jugular vein, as far as the tumour would allow of its being done; and a little dissection now brought into view the omo-hyoideus at the upper part, passing obliquely upwards to its insertion. This muscle was divided, and a small artery passing across the wound immediately below it, was secured; the deep fascia of the neck was here exposed, having on the inner side the anterior scalenus beautifully distinct, and passing to its insertion into the tubercle of the rib. By slightly rotating the head, the different direction of its fibres from those of the sterno-cleido-mastoideus, became remarkably apparent, shewing how important at this stage of the operation it is, that this muscle should be your guide. The fascia was then cautiously divided along the outer edge of the scalenus, and the transverse artery of the neck drawn upwards by a blunt hook, whilst the large vein which accompanies it, but which crosses the wound considerably lower down, was secured by two silk ligatures, and divided. This enabled me to pass my finger along the scalenus to the tubercle of the rib, and to compress the artery where it leaves the chest, about half an inch above that process. The space, however, between the aneurismal tumour and the scalenus was so small, that it was thought advisable to divide a few of its fibres, in order the more readily and securely to tie the vessel. This having been done, a strong blunt aneurismal needle armed with a silk ligature was very readily passed under the artery from below, and its blunt extremity having been pressed upwards, I cut through the cellular tissue upon it, and thus passed the instrument without detaching the vessel from its connexions. The ligature was tied with great ease, and the tumour immediately subsided. All pulsation ceased from that time; the edges of the wound were brought together by means of a suture and some adhesive plaster, and the patient returned to her bed. She bore the operation with remarkable firmness throughout."

There is little in the subsequent occurrences to deserve notice. On the 5th day, the wound was dressed for the first time, and was partially healed. On the 12th day two of the ligatures came away—on the 14th the radial pulse returned—on the 21st day, the ligature on the subclavian separated—and, on the 24th, she is reported as cured. We are told that, up to the present time (November), she continues to enjoy good health, and feels no inconvenience from the operation.

The circumstances to which Mr. Nicholls would direct attention are, 1, the position of the patient during the operation—2, the turning back of the jugular vein, imbedded in the flap—3, the non-division of the clavicular origin of the sterno-cleido-mastoid muscle—4, the drawing upwards of the transverse artery—5, the division and ligature of the transverse vein without bad consequences—and, 6, the ligature of the subclavian, beneath the outer fibres of the scalenus anticus. To these memorabilia, we would add the circumstance of so young a lady having an aneurism at all, and regret that the state of the tumour, subsequent to the operation, has not been more particularly noticed by Mr. Nicholls. We are told that, after tying the vessel, the tumour "immediately subsided." Did it subside entirely, or partially? This we are not told, and yet, to say the least, it would be interesting information—because, if the former, there could have been no coagulum in the aneurismal sac, and it must have been a dilatation only of the artery—if the latter, we ought to be acquainted with the final result.

We throw out these hints to Mr. Nicholls, believing him sincerely desirous of benefitting science by the publication of the case, and we think he will agree with us, that facts, to be really serviceable, should be perfectly explicit.

---

### LX.

### Death after the Introduction of the Catheter.

This case is related in the same Number of the *Lancet* which contains the preceding. We notice it for more reasons than one.

A sailor, æt. 45, had for some time been troubled with stricture, the difficulty of making water being greater at one time than another. In the morning of Sept. 10, he was brought into the London Hospital labouring under retention of urine, with urinous extravasation into the cellular membrane of the scrotum. The introduction of the catheter being impracticable, Mr. Scott "determined on laying open the membranous part of the urethra." The patient was placed in the position for lithotomy, and an incision, as in that operation, was made in the perinæum. A quart of urine was evacuated with relief. There was troublesome bleeding "from the corpus spongiosum," and it was only arrested by including in the ligature a portion of that structure. By this, and the application of cold water, the bleeding was arrested. The volatile alkali was given, and no further bad symptoms occurred. The urine flowed exclusively through the wound in the perinæum till the 2nd October, when a small quantity issued through the urethra. So matters continued till the 19th, when Mr. Scott, after much difficulty, passed a catheter into the bladder, and ordered it to be retained by tapes. At 11, a.m. of the 20th, it slipped out, and, on the 21st, the catheter was re-introduced. Early in the morning of the 23d, symptoms of "acute peritonitis" came on. The patient was ordered castor-oil and leeches, and the bowels not being opened, Mr. Scott directed house-medicine every half hour till they should be so, with calomel and Dover's powder every four hours. In the evening, leeches were again applied. On the 24th he felt much easier, but there was distressing and constant sickness. The bowels had not been opened since the preceding evening, and he was again ordered house-medicine every half-hour till they should be so, to be succeeded by blue-pill and opium, and leeches. The greater portion of urine was passed by the urethra. Next morning he was extremely low, and at 3, a.m. of the 27th he died. The friends would not allow the body to be examined.

It would, perhaps, be idle to throw out many conjectures on the cause of death, certainty on that point being unattainable. But we may be permitted to express a doubt of the existence of peritonitis, to the extent imagined by the reporter. At least, the symptoms were very similar to what we see after lithotomy, and it is now pretty well ascertained, that diffuse inflammation of the cellular membrane of the pelvis is more frequent after that operation than pure peritonitis. Besides, we have seen a case very similar, in many respects, to that before us, in which dissection shewed no peritoneal inflammation. The case was that of an elderly man, with a bad stricture. An instrument was with much difficulty passed, and the urine flowed. On the same evening, or next day, tenderness of the abdomen, with rapid pulse and hot skin, came on. The patient became worse, had vomiting and hiccup, but no rigor. It was thought that there might be mischief in the perinæum, and an incision was made there, but without any result. The man died. On examination, it was found that the instrument had made its way through the side of the urethra, into a cavity behind the neck of the bladder, containing pus. Whether this was, or was not, an old abscess, we cannot say, but certainly the pus thus locked up was the cause of the symptoms and death. Now we do not say that such was the case in Mr. Scott's patient, but we point out the similarity of the cases, and the probability, or, at all events, possibility, of a nearly similar state of things existing in both. We entertain no doubt that the introduction of the instrument, in Mr. Scott's case, was the exciting cause of the mischief, whatever that may have been.

But supposing peritonitis, as was thought, to have existed, we question the propriety of administering a drastic purge, like "house medicine," every half-hour, until it should operate. Are inflamed parts usually the better for such disturbance as this must necessarily have created? If Mr. Scott's patient had inflamed leg, would he set him to run about the ward for some five or ten minutes, in order to answer some end in view? We think not, and, though we may be wrong, we do not approve of the practice to which we have adverted.

A word on the operation at first resorted to, when the patient was affected with retention of urine. As it was determined to cut into the membranous part of the urethra, in the perinæum, might it not have been better to have finished the business, by endeavouring to establish the urethral channel at once? Mr. Scott might have commenced by introducing the staff, or other instrument, as far as it would go, cutting upon it, and following up the incision by dividing the strictured part of the urethra, and opening into the dilated membranous portion. Subsequently a catheter would have been retained in the bladder, and the fatal risk which the patient ultimately ran would have been avoided. It would have been avoided had the operation succeeded, and that would have been quite as likely to have done so, as the operation which Mr. Scott actually performed with temporary success. The procedure to which we have adverted is not merely a speculative one; Mr. Mayo has adopted it on several occasions with success, and we do think that surgeons should give it a trial, to establish fairly its merits or its faults.

---

### LXI.

### WOUNDS OF THE INTESTINES.

DR. WISE has inserted a paper on this interesting subject in the fifth volume of the Transactions of the Medical and Physical Society of Calcutta. We shall notice some facts related by Dr. Wise.

"In many cases of Hernia and Intrsusception, in which the persons recovered, after portions of the intestine had sphacelated, it has been found that an adhesion took place between the parts of the peritoneal sac, naturally in contact with each other. By this mean, extravasation of the contents of the gut, and its fatal consequences, were prevented. In a case of strangulated Hernia, Mr. Hunter found that organized coagulated lymph had formed 24 hours after the operation had been performed. To set free the strictured portion, recourse is sometimes had to the same principle, for the cure of other diseases, as of hydrocele; but it appears that it might be employed with advantage in many cases, in which it is at present neglected. To prove this, experiments on the inferior animals have been instituted; and Dr. Thomson found, that a ligature may be passed round so as to encircle a piece of intestine, and drawn tight with impunity. This is found to be in consequence of the ligature dividing the internal coats of the intestine, producing an adhesive inflammation between the two sides of the serous membrane, immediately above and below the ligature; and a slow ulcerative process, by which the ligature passes into the gut, and is discharged. In Introsusception the same adhesive process takes place between the two serous surfaces, at the commencement of the included portion, which is removed by a slow ulceration at the part where the enclosed joins the included portion. Mr. Jobert, an expert and judicious surgeon of Paris, performed a number of experiments on dogs, to discover to what extent this principle could be relied on. I assisted at some of these experiments, and as I believe they are not generally known, shall now relate the results obtained. Part of the stomach, and different portions of intestines, were exposed, and incisions made in different directions. The lips of the wounds were inverted, or if the Epiploon was near, a piece of it was placed between the lips and several stitches of the interrupted suture were made, including a small portion of the serous and submuscular coats, near the edges of the wound, so as to keep the two serous surfaces in contact; the gut was then returned, and the external wound closed. The dogs were killed at different periods after these experiments, when the wounds were found healed, without any extravasation having taken place of the contents of the gut. In other experiments, the gut was divided across; the serous coat of the inferior portion inverted, and the superior portion passed for a short distance within this. It was secured in this situation by the interrupted suture. The catgut ligatures divided near the knot, and the gut was returned into the abdomen. In these cases, adhesion took place in a few hours, and the animals quickly recovered without any unfavourable symptoms."

It is important to observe that the presence of the ligatures in the abdomen was productive of no ulterior inconvenience. Our readers may probably be aware that in a case in which Mr. Earle, we think, tied some vessels in protruded omentum, cut the ligatures short, and returned the whole into the abdomen, an abscess subsequently formed at the umbilicus, and by it the pieces of ligature were discharged. In the case of the intestine there is this great difference, that the ligature may, and in all probability often does, separate into the cavity of the gut. In an instance of wounded intestine Sir Astley Cooper included the wound in a ligature, cut both ends short, and returned the whole with success. The following two cases were similarly fortunate.

*Case* 1. A man, æt. 60, was brought to Bartholomew's Hospital, while Dr. Wise was house-surgeon with strangulated inguinal hernia, of large size, soft, and the seat of great pain in the neck of the tumor. Seven hours after the commencement of strangulation Mr. Lawrence operated, various means having been employed in the interim to produce reduction. On opening the sac about a foot and a half of small intestine, of a chocolate colour, was exposed. A small hard stricture was found high up in the sac, admitting the director with much difficulty. The stricture was removed, and the gut pulled down a little for the purpose of examination, when a gush of brown fluid took place from a small wound in it, apparently produced by the director. The wounded part was pinched up with a pair of small forceps, a fine ligature passed round beyond the points of the instrument, tied tight, and the extremities cut close to the knot, and the intestine returned into the abdomen. As there was a large mass of omentum slightly adherent, much of it was removed, and the rest left in the wound. Laxatives were given half an hour after the operation, and repeated till the bowels acted. The patient recovered without a bad symptom.

When the wound is more extensive the same principle is applicable. The next case occurred to M. Jule Cloquet, and is related by M. Jobert.*

* *Memoire sur les Plaies du Canal Intestinal. Par A. Jobert, Paris,* 1826.

*Case* 2. "Nicolas Lejeune, aged 41 years; of a middle height, and spare habit; had a Congenital Hernia, which obliged him to wear a truss, as it had several times been strangulated, and was always reduced with difficulty. On the 13th of July, 1826, at ten o'clock, it became strangulated, without any apparent cause. After numerous ineffectual efforts to reduce the hernia, assisted by a surgeon, he was conveyed to the Hospital of St. Louis, five hours after the symptoms appeared. He was then found in the following state:—On the left side, a large, soft tumour descended from the inguinal region, and distended the scrotum. The patient had vomited, and complained of nausea and great thirst; the pulse was small and rapid; respiration frequent and short; great sensibility of the abdomen, with general prostration of the system. The foot-bath, local blood-letting, and the taxis, were employed, without success, and Mr. Cloquet proceeded to remove the stricture with the knife. A transverse portion of the skin, over the tumour, was pinched up, and divided across, for nearly two inches in extent. The fascia superficialis, the cremaster and dartos muscles being divided in the same direction, exposed the hernial sac, a portion of which was raised by the forceps, and a small perforation made into it, and enlarged by a pair of straight scissars. The portion of the intestine included in the hernia, was much inflamed, and distended. While two assistants removed the intestine to the side, Mr. Cloquet passed a bistoury, to divide the stricture, which he had discovered situated in the neck of the sac. He then tried to reduce the gut, but failed, and the bistoury was again introduced, to enlarge the stricture; as he was withdrawing the instrument, a portion of the intestine, held by one of the assistants, escaped, and was divided to the extent of an inch and a half. A quantity of gas and fluid escaped from the wound in the gut, which was found thickened to double its natural size. Two ligatures were passed through the lips of the wound in the intestine, about five lines from its edge, and when tightened, thus brought the serous surfaces together. The extremities of the ligatures were cut close to the intestine, which was then returned into the abdomen. Simple dressing was applied to the external wound, and secured by a T bandage. All the unfavourable symptoms disappeared; and the patient recovered."

The cases will probably be interesting to practical surgeons. We may add to the foregoing, the following instance of wound of the abdomen, though apparently not of the bowels, which we find related in another part of the same volume.

Mr. Thompson reported to the Medical Society a case of wound of the abdomen, which occurred in a Malay man, at Malacca. The wound was inflicted with a spear, which entered above the posterior spinous process of the left os ilii near the last dorsal vertebra; and came through the body, passing out at the linea semi-lunaris of the right side, two inches above the navel, where there was an opening an inch in length, at which a portion of omentum protruded. Very little blood was effused externally. Six hours after the injury was inflicted, the patient had violent pain in the belly, urgent thirst, vomiting, anxious countenance, a small weak pulse, and cold perspirations. The omentum was reduced, and the wounds closed by sutures. V. S. was attemptd, but the man soon fainted; next day he was bled to 12oz.; leeches were ordered on account of painful tension of the belly; and a blister was applied to the abdomen. Enemas, and aperient medicines was administered repeatedly, until the bowels were freely opened. The unfavourable symptoms gradually subsided, and by the 16th day after the wound was inflicted, the patient was able to walk from his house, two miles to the hospital; the wounds were then in a good state, and affording every prospect of recovery. As no blood was observed in the stools, or in the matter vomited soon after the wound was inflicted; there seems reason to believe, that the spear passed through the body, quite across the belly, without wounding the intestines or stomach.

### LXII.

### Ligature of the Carotid for Hemiplegia and Epilepsy.

There is a curious paper on this subject by J. R. Preston, in the volume of the Calcutta Transactions from which the preceding article was taken. We say curious, for the reasoning does appear to us to be extraordinary. It amounts to this, that ligature of the carotid is likely to be serviceable in cases of inflammation, congestion, irritation within the cranium, in our author's own words, in "apoplexy, phrenitis, hydrocephalus, many cases of injury of the head, palsy, epilepsy, and insanity." All this practical induction appears to be bottomed on the principle, that ligature of the carotid diminishes the volume of the blood sent to the head. Now this principle seems to us most futile. The cranium cannot be emptied, nor can the quantity of its contents be diminished. If we take blood out, something else must be sent in. But there is nothing else to supply, on the emergency, the place of blood. Therefore, if we tie one carotid, blood must pass in increased quantity by the other carotid and vertebrals, or, supposing these channels obstructed, it must flow back into the cranium by the veins, or remain stationary in them. This seems to us to be as clear as any demonstrable truth. The principle, therefore, on which Mr. Preston sets out is a false one. We may alter, and, by our medicinal and surgical operations, we do alter, the disposition of the blood in the brain, the energy, or the rapidity with which it circulates, but its actual quantity, other things being the same, we do not, and we cannot alter.

There are other considerations to deter us from expecting benefit from ligature of the carotid in inflammation, congestion, or irritation of the brain. The operation itself is not a slight one, but, in a certain number of cases, would itself be the cause of death. We have no just grounds for concluding, that irritation of any part is removed by diminishing the quantity of blood sent to it. Is depletion useful in tic douloureux, or those nervous symptoms that occasionally follow amputation? Irritation, on the whole, is not benefited by depletion, general or local, and yet it is on the principle (we have shewn its erroneousness) of lessening the quantity of blood sent to the brain, that Mr. Preston proposes ligature of the carotid for epilepsy.

After tying the vessel of a limb, we not unfrequently find that inflammation is set up in the limb below the ligature. After ligature of the femoral artery, we have seen a low inflammation, like chilblain, attack the foot. Numbness and nervous pain are frequent consequences of such an operation, and they often remain for a considerable time. Here, then, we see both inflammation and irritation the result of the ligature of an artery in the extremities. As we never saw the converse experiment made, that of tying the vessel for inflammation or irritation in an extremity, we cannot speak to its results. Now, then, if Mr. Preston were correct in his principle, that ligature of the carotid diminishes the quantity of blood in the brain, analogy would be against the application of that principle in the cases which he mentions. But, in fact, we do not conceive the analogy a fair one, because we do not admit the correctness of Mr. Preston's principle.

We will not pursue the reasoning any further. Mr. Preston may be manifestly wrong in his theory, and yet his practice may be good. Let us look at the facts themselves.

Case 1.—*Ligature of the Common Carotid Artery for Hemiplegia.*

Peter Rochford, æt. 50, king's pensioner, was admitted into hospital, Oct. 25th, with hemiplegia of the left side—some pain in the paralytic leg—circulation unaffected—skin cool. The symptoms came on during the preceding night. He had been a hard liver.

He was ordered a blister, calomel, iodine. On the 28th, the mouth was slightly affected—on the 29th, a seton—on Nov. 1st, the mouth sore, and the calomel omitted. On the 5th, sensation had returned in the arm and leg, and there was much pain in the knee. On the 8th, more difficulty of speaking. Croton oil. On the 9th, better; he now took tinct. iodin. ♏xviij. 6tis horis. On the 13th, much the same. Nux vomica. On the 17th, some uneasiness in the liver, for which leeches were employed. On the 21st, much the same—pulse 72, and intermitting more frequently on the left than on the right side; an ulcer on the sacrum, from pressure.

On the 23d, Mr. Preston tied the right common carotid artery. He commenced the incision, three inches long, at the thyroid cartilage, and carried it *upwards*. He found the artery very deep, and tied it. Mr. Preston appears to have made his incision somewhat too high. This artery usually bifurcates at the upper border of the thyroid cartilage, and, therefore, an incision commencing opposite this cartilage, and carried upwards, must have had the common carotid quite at its inferior extremity, instead of near its centre. The common direction for securing the common carotid, in the upper part of its course, is to prolong the incision downwards to near the lower border of the cricoid cartilage.

On the 23d there was slight fever, some cough, and difficulty of swallowing. On the 24th, slight uneasiness in the side of the head. On the 25th rather more cough and uneasiness of the chest. Castor oil and opiated sudorifics. On Dec. 1st, he was put on half diet. On the 4th, he could draw up the leg a little. He had much pain in the paralytic arm. On the 12th, he could walk about with the assistance of a stick. On this day the ligature came away. On the 21st, he "could walk about pretty well with the assistance of a stick." The arm seems to have continued paralysed, and he had much pain in it. On the 22d, the report ends.

Now we really see nothing whatever in this case to recommend the operation. Is it not common, we speak to practical men, is it not common for patients affected with hemiplegia so mildly as this man was, to recover gradually some use of, not only the lower, but also the upper limb? To be sure it is. Hemiplegiacs may be seen creeping on with a stick in any of our public streets, in any of our public hospitals; and yet, after undergoing the pain and the risk of a formidable operation, Mr. Preston's patient is only reported to be "walking pretty well with a stick," his arm continuing paralysed. We venture to say that, with regular cathartics, slight mercurial action, and judicious and consistent counter-irritation, the man would have been as well, if not better, on the 22d of December, where Mr. Preston's report left him.

CASE 2.—*Ligature of the Common Carotid Artery for Epilepsy.*

Michael Cox, pensioner, æt. 25, had for five years been subject to severe epileptic fits, which generally recurred about once in a fortnight. He was first attacked while on duty at Burmah, after having been much exposed to the sun, and undergone extreme fatigue. Since the first seizure, the fits had generally occurred without assignable cause, but were occasionally induced by intemperance; he was unable to drink as much liquor as European soldiers usually do, a very small quantity producing giddiness, &c. He had frequently been bled, but no other treatment appeared to have been adopted.

Mr. Preston performed the operation on the 4th February, and was obliged to bleed him during its performance, in consequence of a threatening of an attack. On the 24th he was discharged the hospital, the wound being healed, with the exception of the part through which the ligature hung out. On the 5th March, the ligature came away. The report ends on the 13th April. He had had no fit, although he had returned to his work, and had drunk hard.

Thus, in this case, there was no epileptic fit for upwards of two months from the performance of the operation. If there is none after the expiration of two years, the evidence will assume a satisfactory appearance; but Mr. Preston will excuse us from attaching much importance to it yet. In conclusion, we recommend these cases to our readers' notice. Let them discard all theoretical notions, and look to the facts.

Mr. Preston might be utterly wrong in his reasoning, and yet the operation founded on that reasoning might be good. Its value is only to be tested by the cases in which it was performed. If our readers are satisfied with them, it is well—we are not so.

---

### LXIII.

Surgical Anatomy of the Arteries, with Plates and Illustrations. By Nathan R. Smith, M.D. Professor of Surgery in the University of Maryland, and one of the Surgeons of the Baltimore Infirmary. Baltimore, 1832.

Nothing affords us more sincere gratification, than any opportunity of directing the attention of medical men in this country to the labours of their brethren in America. Sprung, we may say, from our loins, speaking the same language, cherishing the same customs, actuated by the same love of political liberty, and displaying the same energy of individual purpose as ourselves, what Englishman but must look with pride upon their growing greatness—feel an interest in their welfare—and offer his best wishes for their success. It may be, that in the changes now ominously impending over Europe, Britain, the modern nurse of freedom, science, and the arts, may fall a prey to despotism or to anarchy, and her name be blotted from the list of nations. Should that day arrive, and such a fate has overtaken far mightier empires, we shall live in our offspring still, and America will shew what Britain was.

Americans may be assured, that the feeling entertained towards them by the mass of the liberal and enlightened here is one of unmixed good will. We know how important it is, that kindly sentiments should take root and flourish in either land, and we look upon the attempts of fools or knaves to sow disunion with deep indignation and bitter contempt. This spirit pervades our scientific, as well as our political relations, and the paltry scribbler hardly exists, who would dare insult the public taste by such a tirade against American literature as once was penned in Scotland. The Review which contained that ill-advised criticism has since made ample and honorable amends to America, and few can read its notice of Mrs. Trollope's book, without applauding its manly and liberal tone.

The volume before us is calculated to be highly serviceable to students in surgery, old or young. The object of Mr. Smith, the able author, is to place before his countrymen a native work, which shall satisfy their wants in this department of science. We think he has succeeded, and, as children of a soil where patriotism is not altogether uprooted, we can fully appreciate Mr. Smith's motives, and not only appreciate, but approve.

The work is in quarto, contains 104 pages of close letter-press, and 18 lithographic plates, the arteries in which are coloured. The three concluding plates represent the mode of exposing and tying the carotid, subclavian, brachial, ulnar and radial, inguinal, popliteal and anterior tibial arteries. The fifteen preceding these are devoted to the anatomy of the arterial system. They are copies of the excellent plates of Cloquet, and well executed. In justice, however, to Mr. Knox, our valued friend, the able translator of Cloquet, we must confess that they are inferior to the engravings of the same vessels which he has published, and is selling at a very cheap rate. We think that if Mr. Smith had an opportunity of seeing these engravings, he would candidly admit the inferiority of his own.

Mr. Smith has adopted a method of shewing the absolute size of the arteries which he describes, which appears quite new. Thus, the arteria innominata is marked in this manner :—

The diagram is coloured red, and the numbers mark the branches, as well as the spots whence they arise.

In a journal of the nature of ours, it is incompatible with our plan to notice elementary works at length. We cannot, therefore, analyze or review Mr. Smith's. When we say that it comprises all the information of the day on the subject to which it is devoted, we only render a due tribute of praise to the able and industrious author. But there is one part to which we can allude, and to which we are, indeed, pleased at having the opportunity of alluding. Our readers will see, in another part of this Number, the experiments of Mr. Hawkins on hæmorrhage from arteries. Now it happens that Mr. Smith has been following up the experiments of Dr. Jones, and while Mr. Hawkins has been employed in confirming them, Mr. Smith has been equally profitably occupied in extending them. The process adopted by Nature to arrest bleeding from lacerated arteries was not thoroughly investigated by Dr. Jones. He himself confessed it. Since his time, as before it, a degree of discreditable mysticism has hung over the subject. Mr. Smith has, we think, gone far to dispel it. We cannot compress, with advantage, the experiments and conclusions of Mr. Smith. We, therefore, give them entire; and we do this the more readily, as they will necessarily be new to the majority of our English readers.

*Cause of the Spontaneous Cessation of Hæmorrhage from Lacerated Arteries.*

"It is a well known, fact, that when a limb is torn from the body, or when organs involving very large arteries are rudely lacerated by obtuse instruments, hemorrhage will often spontaneously cease, even from vessels which always bleed fatally when smoothly cut. Cheselden's case, in which the arm, with the scapula, was torn from the body, will occur to the reader, and perhaps others of a similar character. Such a case fell under my own observation some years since, in the state of Vermont. A young man was caught by the arm in the drum wheel of a factory, and the limb, together with the scapula, was rent from the body. I saw the boy a week after the accident, and witnessed the dressing of the stump. I was informed that but a very small quantity of blood had been lost, although no arteries had been secured. No secondary hemorrhage ever took place, and the patient recovered with surprising facility. The case was treated by the intelligent Professor of Surgery in Dartmouth college, Dr. Mussey, who has given an interesting account of the case in the New England Journal of Medicine. Many similar cases are on record.

Mr. Jones paid but little attention to the subject of lacerated arteries, and does not appear to have performed any experiments particularly for the purpose of comparing such injuries with others, in relation to hemorrhage. In one instance, however, he lacerated the carotid of a horse, and the animal bled to death. In another instance, he did the same, but arrested the bleeding by pressure on the artery. He reports that, in these cases, the internal coat was lacerated in many places, and that there were formed internal coagula, large enough to fill the artery, and that they were attached to it by lymph effused from the fractures in the internal coat. Although he seems to think the internal coagulum a more perfect barrier in this case, yet he does not appear to have ascertained its comparative influence in suppressing hemorrhage, indeed, he says that 'the natural means of suppressing hemorrhage, the peculiar state of the coagulum excepted, were the same in these cases of lacerated arteries, as in ordinary wounds of arteries, but I am not solicitous of pressing this opinion.'

The opinions which have been stated by various surgeons, relative to the spontaneous cessation of hemorrhage from lacerated arteries, are exceedingly vague and contradictory. From this we may infer that the subject has not been thoroughly investigated by experiment. M. Richerand* states that large arteries, when ruptured, become closed, (*se reserrent*) partly in consequence of the chill which they suffer,

* Nosographie Chirurgicale, tome i, p. 170.

producing spasm, and partly by the pressure which the muscles, within which they retract, exercise upon them. M. Delpech* states, that when a limb has been torn from the body, the principal artery is sometimes broken within the parts of the stump which have resisted the violence, so as to hang, out at the wound, and sometimes within the lacerated limb. In neither case, he says, is hæmorrhage apt to take place. He has so much confidence in the security of the vessels, that he advises not to seek for them in treating lacerated wounds unless they bleed. Mr. Charles Bell says, ‘A torn artery does not bleed. I have heard it affirmed that, in this case, the blood was stopped by the rugged portions of the inner coat of the vessel, which is torn into shreds by the violent elongation of it. It has been said, if we disclose the radial artery of a dead body, and, putting a probe under it, tear it forcibly, the inner coat will present an appearance of valves to intercept the flow of blood. I believed in this statement, but, upon the experiment being repeated, I found that in a young and healthy artery the change could not be exhibited.’ Professor Gibson† asserts that, the indisposition manifested by a lacerated part to bleed is owing to the injury sustained by the nerves, not only in the immediate vicinity of the wound, but to a greater extent around than the eye can discover. Hence, the arteries are paralyzed, and do not contract to propel the blood which coagulates in their cavities, or among the torn muscular fibres.

It is apparent, therefore, that the mode in which hemorrhage from lacerated arteries is arrested, is by no means an established principle in surgery. For the purpose of furnishing facts which may aid to render it such, the following experiments were instituted.

Exper. 1. Having exposed the femoral artery of a young slut, not fully grown, I passed a smooth iron hook under it, and lacerated the organ with a sudden pull. Blood immediately gushed from it in a rapid stream, and continued to flow copiously for about four minutes. At the end of that time, the blood on the table began to coagulate, and, simultaneously, the bleeding began to be less impetuous. It gradually diminished, and in ten minutes had ceased altogether. The animal was then shut up, but suffered to move about the room. No bleeding recurred. At the end of twenty-four hours she appeared quite well—moved the limb with freedom, and took food greedily. She was then killed with prussic acid. On examining the limb, it was found slightly swelled. Blood was injected, in small quantity, into the common tissue, and a coagulum had formed in the sheath, around the artery. The upper extremity of the artery was not retracted between the muscles, but was quite superficial. The external coagulum had not exercised much pressure upon it, for its extremity was larger than natural. I dissected the artery from its sheath, to the extent of three or four inches, and opened it longitudinally from above downward. Two inches from the wound I encountered a slender coagulum, which increased in diameter as I traced it downward, and completely stuffed the organ for one inch from its orifice. The external coat presented a lacerated margin, which, however, had become somewhat indistinct by the effusion of lymph. The internal coat was lacerated transversely in many places. Into many of these, slips of the internal coagulum were inserted. The blood which had issued from them appeared to have incorporated itself with that which filled the vessel, and thus to have, at first, attached the coagulum. From many other fissures a very apparent quantity of lymph had been effused—had blended itself with the coagulum, and fixed it so firmly in its place, that it was difficult to scrape it away. The artery was so firmly stuffed with the coagulum as to be considerably dilated. Not a drop of blood could possibly have escaped from it in this state.

Exper. 2. The carotid artery of a full grown dog, of large size, was ex-

* Precis des Maladies Chirurgicales. Par J. Delpech, tome i, p. 188.

† Gibson's Surgery, vol. 1, p. 92.

posed on the left side of the neck, and lacerated as before. The artery broke deep in the chest, and bled for five minutes with great rapidity. The animal then gave signs of fainting, but these soon disappeared and the blood quickly ceased to flow. He was suffered to live for four hours, during which time there was no bleeding. He was then killed, and while dying he struggled very violently, but there was still no bleeding. The chest was then opened, and the artery traced from its origin. It proved to be a branch of the innominata. Its internal coats were broken at its very origin—the external was broken at the distance of an inch and a quarter from the innominata. The internal coats were withdrawn from within the external, which formed a loose pouch projecting from the innominata and stuffed with a firm coagulum. In this case there was no lymph effused, sufficient time not having elapsed. The external coagulum was voluminous and firm, occupying the interstices of the adjacent organs, and extending to the external wound. It had not, however, made pressure enough to interfere with respiration. The cervical portion of the broken artery was hanging from the wound, to the extent of two or three inches. This had also bled freely at the moment of the rupture, but had soon ceased to do so. Its internal coat was ruptured transversely at many places along the trunk of the artery. Near the extremity, it was filled with a coagulum which adhered to the transverse fissures in the internal coat. This must have been, of itself, an effectual barrier against the effusion of blood.

Exper. 3. I procured a horse, twelve years of age, of pretty good constitution, though very lean, and having cast him upon his side, laid bare the carotid. I then passed a smooth iron under the artery and broke it, as I had done in the previous experiments. The blood gushed in a torrent from the wound, and in a few minutes the animal lost two or three gallons. In about ten minutes the blood upon the ground began to coagulate, and then a diminution in the rapidity of the current was manifest. The extremity towards the chest hung out at the wound to the extent of three inches. While the blood was flowing rapidly, the animal moaned once or twice, as if faint; but soon after he rose from the ground without difficulty, and stood till the blood had entirely ceased to flow, which was after about thirty minutes from the time the artery was ruptured. The projecting artery was then returned to its place, and the wound closed.

The animal was suffered to live for twenty-four hours, during which time he appeared nearly as vigorous as before the operation, and took food with avidity. He was then killed by a blow on the head, but, while dying, he struggled very violently. Blood gushed from small vessels in the wound, and I feared, at first, that the obstructions in the artery had given way. But, on examination, I discovered that not a drop of blood had issued from either extremity of the artery. The lower portion of the organ was found perfectly naked, to the extent of three inches from the rupture. Beyond this, its sheath was occupied with a coagulum, as was also the common tissue in the vicinity. No lateral pressure, however, had been exercised on the artery, to impede the passage of blood, as the organ was even increased in volume. The interior of the artery was found firmly plugged with a coagulum six inches long, and completely filling its cavity for nearly the whole extent of the coagulum. The internal coat, as in the preceding experiments, was ruptured transversely at a great many places, and little productions of the internal coagulum were inserted into them so firmly that they must have fixed the coagulum securely, as soon as coagulation had taken place. From many of the fractures in the internal coat, lymph, in quantity, had been effused, and become blended with the coagulum, which was thus so firmly attached to the surface of the organ that it required an effort to detach it. No blood could possibly have passed through it, and, as the coats of the artery every where retained their vitality, no secondary hæmorrhage could have subsequently occurred. The accompanying plate accurately represents the preparation which I made of the parts by slitting open the artery longitudinally, and leaving the coagulum in its place. Fig. 1 marks the coats of the artery, near the broken extremity; 2, the coagulum; 3, a point at which the thyroid artery was broken off at its origin, and where the internal and middle coats were lacerated more extensively than at any other part. Here, too, was the greatest quantity of lymph, completely sealing up the organ.

Exper. 4. The carotid of a large dog was laid bare, raised with a hook from its sheath and divided. Its extremities then retreated into the sheath. The gush of blood was impetuous, but at the first moment little if any more so than when, in experiment 2, the artery was torn. There occurred no abatement in its force, however, till the animal fainted, which was after five minutes. The bleeding then almost entirely ceased for a moment, but presently returned, and in less than ten minutes the animal expired. The abdomen of this dog was then opened, a hook was passed under the aorta and the vessel was broken. The organ being dissected out was then examined. Its internal coat was ruptured, precisely as by similar means in the other experiments. In some places it was peeled up from the middle coat, so as to form pockets on the side of the artery; but this was the only instance in which I found any thing of the kind.

Exper. 5. I opened the abdomen of a large dog, and having exposed the aorta above its bifurcation, I attempted to break it in the manner mentioned above. The flow of blood was very rapid, and this, together with the irritation necessarily produced by the exposure of the abdominal organs, rapidly prostrated the powers of life, and the animal died in about five minutes. There was, however, an ineffectual effort at re-action. No other result could have been expected in this experiment, because the bleeding was so copious as to destroy life before coagulation could be effected. On examination I found that the aorta itself was not ruptured, and that the laceration had taken place in the external and internal iliacs. The blood, therefore, had flowed from several large trunks, a wound of either of which is ordinarily fatal. I was surprised to find, however, that each of the lacerated arteries had its orifice closed with a coagulum which had probably been formed *in articulo mortis.* The internal coat of each was lacerated as in the other experiments, and a portion of the external coat, in every instance, projected beyond the lacerated margin of the internal coats, to the extent of half an inch or more. The extremity of each artery appeared enlarged and bulbous, from the pressure of the coagulum within it, adhering to its rough cellular surface. I was persuaded that, had not the flow of blood been so rapid as to destroy life before coagulation could be completed, this animal would not have perished immediately.

The above experiments will, I think, justify the following conclusions. 1st. That Dr. Jones errs in ascribing the cessation of hemorrhage from lacerated arteries mainly to the same causes which avail against bleeding from divided arteries. In the experiments detailed above, the retraction and contraction of the organ availed nothing. The same was true in regard to the external coagulum, which, according to Dr. Jones, is the principal agent. In each of the experiments, the extremities of the vessel were rather dilated than constricted or compressed. In one instance, (Exper. 3d.) the artery hung naked from the wound, and yet the bleeding ceased as promptly as under other circumstances.

2d. Equally untenable is the doctrine that the artery is paralysed by the shock inflicted upon its contractile tissue, and that the blood refuses to flow through a passive tube. In every experiment there was extensive injury inflicted upon the artery, and in one the organ was torn from the surrounding parts to a great extent; consequently its vital intercourse with them must have been for a time interrupted, and its own actions suspended. This paralysis of the artery should be most perfect instantly after the injury; but we find that the blood then flowed in a rapid stream which was undiminished till it had had time to coagulate. It is true that in those cases on record in which limbs have been torn from the body, there has been, even at the moment of the injury, no considerable bleeding. But this has undoubtedly arisen from the shock given to the general system, and the suspension of the action of the heart till the blood had coagulated in the extremity of the artery.

3d. Although the pockets, or valves, mentioned by Mr. Bell, were, in one experiment, formed on the sides of the lacerated vessels by the rupture and partial detachment of the inner coat, yet, when the experiment was performed on the living animal, they were not found efficient in suppressing hemorrhage in a single instance.

4th. The efficient, and almost only cause of the cessation of hemorrhage from lacerated arteries, is the unequal laceration of the external and internal coats. Generally the internal coat is fractured transversely at numerous places, so as to present an indefinite number of small fissures, into which the blood of the artery is injected, and thence, perhaps, conveyed by interstitial absorption into the arterial tissues. Blood also probably flows from the ruptured tissue, and mingles with that in the cavity of the artery. As soon as coagulation begins to take place, blood concretes, probably first on the fissures produced in the internal coat, attaching itself to the rough surface which is there produced, and insinuating itself into the interstices in such a manner that the coagulum becomes so firmly fixed as to resist the impulse of the circulating blood. When an artery is smoothly cut, and the integrity of the inner coat is uninjured, the coagulum finds no point-d'appui, or roughness upon which it can take hold. The surface is every where polished and lubricated for the very purpose of facilitating the passage of the blood, and therefore the internal coagulum, as it forms, either glides from the artery, when the organ is very large, or perhaps does not form at all, the particles finding no rallying point within the vessel. But when the internal membrane is extensively ruptured, blood must necessarily concrete upon the lacerated surface, precisely as it is uniformly observed to do in other wounds.

In some instances the complete separation of the internal coats will take place at some distance from that of the external, or cellular, which will then hang as a loose pouch from the end of the more rigid middle coat. Its surface being cellular and lacerated, the blood, rushing along it with force, is injected into this tissue, coagulates upon it, and attaches itself firmly to it. The external coagulum, which forms in the lacerated sheath, will then aid to sustain the internal coagulum, and suppress the hemorrhage. After the internal coagulum has been for some hours attached to the fissures produced in the internal coats, from each one of the ruptures there takes place the effusion of lymph. This still more firmly attaches the coagulum to the internal surface of the organ, and the more effectually stuffs its cavity; finally it takes the place of the coagulum of blood, and obliterates the cavity of the artery.

It may be asked, if nature resorts with so much uniformity, precision and effect, to the means of suppressing hemorrhage from lacerated arteries, how does it occur that fatal hemorrhage should so often result from the rupture of arteries which are broken without a corresponding rupture of surrounding parts and of the skin? These injuries are sometimes inflicted on large vessels in effecting the reduction of old dislocations. Many cases of the kind are on record. The probability is that arteries thus yield in these instances, sooner than the surrounding parts, because they are diseased and brittle. The external coat, having lost its extensibility, in consequence of the deposition of lymph in and around it, breaks abruptly without effecting the laceration of the internal coat at more than one place. Besides, we know that when there is no external wound, effused blood does not coagulate with facility, and, remaining fluid, opposes no obstacle to fatal hemorrhage. If Mr. Scudamore's explanation of the coagulation of blood be correct, it refuses to coagulate promptly under these circumstances, because it cannot exhale its carbonic acid."

The experiments speak for themselves, the conclusions are inductive, and we can add nothing worthy of attention to either.

Mr. Smith has made what we really think is an improvement on the artery-forceps. All surgeons know that the "tenaculum of Assalini" is the best instrument, on the whole, for taking hold of arteries in order to tie them. But, occasionally, they are less adapted for the purpose than a pair of forceps which will not transfix the vessel. We then use a pair of forceps with a slide; but all who have used such, must acknowledge its clumsiness and inconveniences. Mr. Smith has made an alteration in the instrument. Instead of a slide, a spring projects from the inside of one of the blades, and passes through a hole in the other. On this spring there is a catch, which, when the blades are firmly compressed, takes hold of the blade, which it pierces, and keeps the instrument closed. "Its peculiar utility consists in the operator being able, when he has caught the organ with his forceps, by gently pressing the blades, to fix the instrument by merely increasing the pressure, instead of employing both hands, as is necessary in moving the slide, and which is very apt to disengage the forceps." The blades are excavated just above the points, in order that they may be less apt to slip, and that any small substance getting between them may not defeat the seizure of a small artery.

### LXIV.

### Mr. Schloss's Anatomical Plates.

*The Anatomy of the Horse. By Dr. E. F. Gwilt, translated from the original German, by J. Willimott, &c. In two Parts—Part* I.

These are folio lithographic, uncolored plates. They represent the osseous and muscular systems, the viscera &c. of the horse. They are fairly executed, and we do not doubt that they are accurate. Many of our own profession are probably anxious to become acquainted with the anatomy of an animal, of essential service to themselves, and of high interest to the mass of the community. To such, and to the veterinary student, these plates must be acceptable, and to such we recommend them. Mr. Schloss, indeed, deserves every encouragement. Few booksellers, native or foreign, are as enterprising or as persevering as he is, none more attentive and obliging.

### LXV.

### Excerpta Choleralogica.

#### I. Dr. Haslewood and Mr. Mordey on Cholera.

So long as the pestilence continues amongst us, we must dedicate a portion of our Journal to the mournful subject. We had intended to notice the joint publication of the gentlemen above-mentioned in our last Number, but the work was mislaid among the pile of cholera monographs which crowded our library, during a temporary absence from home. Our present notice of the book—indeed of all books on the same subject, must be short, since novelties in theory or practice are sure to be antiquated, and even annihilated, in the course of a few weeks—so ephemeral is the existence of new lights in this fatal epidemic.

The authors in question had charge of the cholera hospital in Sunderland, and, consequently, had ample opportunities for observation. They have laudably enforced attention to the premonitory symptoms, by which success is almost secured. We quite agree with them, that those who relate cases where the violent forms of the disease manifested themselves, without previous disorder, have deceived themselves from want of accurate investigation. The following concise, but graphic portrait of the premonitory stage, is deserving of record.

PREMONITORY SYMPTOMS.

"In general it has been found that slight oppression of the breathing, with a soft and somewhat accelerated pulse, a degree of mental depression and inaction, anxiety of countenance, giddiness, and some muscular debility, are the earliest indications of the disease; the abdomen almost invariably feels distended, and is affected with slight transient pains: the urine is scanty and pale. These symptoms are usually followed by a moderate diarrhœa, the discharges being natural, consisting of the usual ingesta: the character of the discharges, however (if the diarrhœa continues long enough uninterrupted by other violent symptoms), gradually changes; and they have, in some instances, assumed precisely the choleric character before any vomiting, cramp, or collapse had unequivocally declared the nature of the disease. Two other symptoms remain to be mentioned, which are of very frequent occurrence, and which, in conjunction with those previously noticed, may be considered almost diagnostic. We allude to slight cramps affecting the fingers and toes, or prevailing still more generally, and coming on during the night; and to a numbness, and feeling of inability to move the limbs, approaching to paralysis: it is not a real inability, as a strong effort is sufficient to dispel the illusion. But the sensation recurs; and it is fortunate for the patient if it create sufficient alarm to induce him to seek, without delay, medical assistance.

After the duration of the premonitory symptoms for a period varying from a few hours to as many days, the disease manifests itself in a manner which, once witnessed, can never be forgotten."

The stage alluded to is that of collapse, of which we need not repeat the portrait. Of the treatment, too, we need not speak, since subsequent experience has shewn the inefficacy of almost every remedy enumerated by our authors. The same experience, indeed, has proved that many of them are not merely inefficient, but that they are actually injurious. We mean the large and repeated doses of stimulants.

In respect to the mode of propagation, our authors are contagionists of the very first class—of the brightest water. For although many of our stanchest advocates for that doctrine have acknowledged that the laws of choleric contagion were obscure, while others of them have lowered the degree of contagion almost to a minim, yet Messrs. Haslewood and Mordey see nothing but contagion, and that as plainly working as one of the steam-engines in a Sunderland colliery!

"From the facts above stated, which in conformity with the *great preponderance of testimony from the Continent, and especially with that contained in the very interesting letters of Dr. Becker*, concerning the facts observed at Berlin, we can no longer doubt that the disease propagates itself *in strict accordance with the known laws of contagion*.

This view of the subject suggests at once the necessity of precautions being used to stop the progress of the malady."

Thus, it only wanted the cordons, the red crosses, the total seclusion and sequestration of the sick at Sunderland, to stop the epidemic!! God help you, good easy gentlemen! Ye have been at least twelve months too late in publishing.

---

II. DR. KEIR (OF MOSCOW) ON CHOLERA.

THIS gentleman, who has grown grey in the practice of his profession, and who is also a contagionist, is very far from being so positive in his opinions as our brethren of Sunderland abovementioned. The following extract is interesting.

"Although some of the medical men of this city are of opinion that cases of this epidemic had begun to appear as early as August, still no case of the disease, as far as I have been able to learn, came to the knowledge of the medical police, or was recognized as Cholera, till the evening of the 14th (26th) September, when Demetry Michaeleff, a doorkeeper, residing in the quarter of the city called the Sretinka, near an open canal, which was under repair, and being covered over this last

Autumn, 1830, was attacked with it. The ground through which this canal runs is apparently for some depth a black loam, and though open for a considerable space in the neighbourhood of the doorkeeper's dwelling, has for many years past been a waste, on both sides of the canal, not unfrequently boggy, and a receptacle for the impurities of the neighbourhood. The water running through this canal empties itself into the river of Moscow at a distance of about nearly three versts from the doorkeeper's dwelling, and the ground rises gradually by a considerable acclivity from this water-course on either side of the canal, in nearly an easterly and westerly direction, till it is intersected by the Sretinka street on the east, and the Tverskaia on the west, both situated at a considerable elevation above the level of the canal, and in the most elevated ground in Moscow.

I have been thus particular in describing these localities, because here one of the first, if not the first case of the disease was met with, and because it was in the hospital attached to this quarter, of which I was inspector, that I had my principal experience of the disease."

"After an interval of a few days the disease began to shew itself in different parts of the town, at a considerable distance from the doorkeeper's dwelling, and without there being any suspicion of communication between him and others affected by it. It now became more widely extended, and proceeding with rapid strides affected persons of all ages and constitutions, from five to above seventy, but more particularly the lower classes from fifteen to sixty."

The following is Dr. Keir's very moderate creed.

"At the same time that I admit the communication of the disease from one person to another, under particular circumstances, as in the wards of an hospital, or in a small ill ventilated room, and *a fortiori*, if other depressing causes are at the same time in action, and while I think it probable that it was imported to Moscow either from Nijney Novogorod, or Saratoff, still it must be granted, that the agency of an epidemic constitution of the atmosphere was sufficiently marked, while the communication of the disease from one person to another, excepting in situations as above-mentioned, was much less evident."

A few pages farther on, where K. comes to develope his ideas more fully, we find the following passage :—

"To have produced it, some very widely extended cause must have been in action; what that cause may have been, or how produced, I know not; but judging by its effects, and the character of the disease, it seems to be a poisonous agent or miasm, possessed of considerable specific gravity, by which it is disposed to lodge more particularly in the lower strata of the atmosphere, *perhaps proceeding from the bowels of the earth;* hence one reason of its tendency to affect more especially those who live on rivers and canals, in low situations, cellars, ground floors, &c."

From the source in question we cannot expect any interesting information on the subject of treatment. Dr. K. is an advocate for mercury in the epidemic cholera.

---

### III. Mr. Morrah on Sulphate of Copper.

This gentleman, who we know to be a very intelligent practitioner, was induced to try the sulphate of copper, in a scruple dose to a cholera patient. It remained a quarter of an hour on the stomach, and then acted. After its operation was over, the skin became warmer, the pulse more distinct, and the spasms mitigated. Two grains of calomel and a table-spoonful of beef-tea were given every half hour. At night three drops of croton oil, which acted on the bowels, and appeared to improve the secretions. The patient slowly recovered. Some other successful cases are related by Morrah in the Medical Gazette.

---

### IV. Dr. Tweedie on Cholera.

In the Medical Gazette Dr. Tweedie remarks that in his opinion the epidemic may be stopped at any period anterior to collapse by efficient doses of opium. He properly observes that some secretions are more easily stopped than others—that the biliary secretion will be impeded by a dose of opium, which will have little or no effect on diarrhœa—"but that if a sufficient dose be given to stop every secretion, there has not, so far as he has witnessed, been any but the happiest result from it." Dr. T. observes that there is no necessity for giving aperients for a day or two after the diarrhœa is checked, as the bowels will generally act spontaneously, if let alone. In the stage of collapse he has seen no remedy that deserves especial praise. "The *saline* treatment has increased involuntary purging, and done harm; or the powders have been immediately vomited, and did no good." Excessive opiates have quieted the sufferings, but have done no good. Stimulants, in *excess*, have appeared injurious. In moderation, "they are not injurious." The few who have recovered from collapse, have done so under frequent doses of calomel, with or without small doses of opium, according to the degree of purging, &c. Cold water or other cold drink is allowed ad libitum, and its repeated rejection is no objection.

MR. STEWARD.

The above gentleman, who had charge of the Droitwich Lunatic Asylum, administered brisk doses of tartrite of antimony (three grains) till the stomach became quiet, which it usually did, and afterwards, the same quantity of the tartrite, with one grain of opium every hour. "In no case thus treated from the commencement, did the sickness rest beyond four doses." Very often both the vomiting and purging ceased; but where the latter continued, rhubarb and magnesia with laudanum were given, followed by calomel and capsicum.

In the Lancashire Lunatic Asylum, the medical officers have combined the saline treatment of Dr. Stevens with the cold water of Dr. Shute, and the mercurial treatment of the India practitioners, with success, according to their statements. Mr. Langford, of one of the Manchester Cholera Hospitals, has practised the antimonial treatment, on the principle of inducing the *tolerance* of the medicine.

DR. AYRE.

This intelligent physician boasts, and we hope with reason, of the "*eminently successful* practice" which he has pursued in cholera—all the cases being treated on the same plan, and all by one remedy. Our readers are aware that Dr. Ayre pursued the mercurial practice, and he now offers to prove his success under the inspection of any competent commission.

MR. HICKMAN.

This gentleman, who had served several years, and treated cholera on a large scale there, has addressed the Central Board of Health, stating his conviction that "all our hardly-earned pathological, physiological, anatomical, and therapeutic experience, has been laid aside, in order to seek for new modi medendi, which have been blazoned forth to the public only to suffer defeat—nay, more, have by their failure increased the dread, and aggravated unnecessarily the feelings of the public respecting this vampire in the shape of disease." Mr. H. then gives a brief outline of his own mode of treatment, which we transcribe from the Lancet.

"1st. When in the form of bilious diarrhœa:—In this stage, avoiding as much as possible all known *lædentia*, and employing, in an equal degree, all known *juvantia*. I have trusted entirely to the conf. opii in doses gr. xxxvj. in aquæ pimentæ ℥j. to adults, repeated at well-timed intervals, watching the patient attentively. The success attendant on this treatment has been most signal.

2d. In that of rice-water evacuations:—In conjunction with the above, I have employed doses of calomel and camphor, gr. iij. of each in the form of a powder.

3d. In the stage of collapse:—Here I have relied (cæteris paribus) entirely on full doses (℈j.) of calomel, with moderate quantities (gr. j. or gr. ½) of opium, *mercurial inunction* and calomel and camphor gr. iij. of each every half hour, or every second, or every third hour, combining with each powder as reaction became established one grain of pulvis calumbæ, which I consider highly valuable."

Mr. Hickman protests against the employment of a farrago of medicines, poured into a stomach, already highly irritable, with deadly and empirical velocity.

---

### V. Messrs. Tweedie and Gaselee on Cholera.

These gentlemen have written a joint pamphlet on the epidemic, which is deserving of attention. Mr. Tweedie having been resident medical officer in the cholera hospital, in Abchurch-lane, has had ample opportunities of studying the disease, and observing the effects—therefore his remarks are valuable. The authors are ultra-contagionists—their belief is as deep as the ocean, and, if they had been Romans or Neapolitans, they would have believed in the liquefaction of the blood of Januarius much more firmly than in their own existence. But we shall pass over this part of the subject, and see how this deep and solemn faith in contagion tallies with the "*precautions*" which our authors recommend against catching the disease. Respecting " protecting belts, anticholera medicines, spells, charms, and bags to dispel infection," our authors seem doubtful whether they are useful or injurious ; but—

" The only precautionary measures which we would recommend, are temperance, cleanliness, and avoiding, as much as is consistent with duty, unnecessary exposure to those who are diseased."

This is very moderate for ultra-contagionists, and the same precautions apply to almost every disease into which pyrexia or profluvium enters.

The authors pass on to less debateable subjects.

" The disease, as it has occurred to us in this metropolis, is almost invariably preceded by an irritable condition of the bowels or actual diarrhœa, varying in its intensity and duration from one hour, to two, three or more days ; occasionally even a longer period ; prior to which anomalous feelings may have been experienced in the abdomen, especially a griping pain in the epigastric and umbilical regions ; and a state of constipation may even have pre-existed, but frequently mere diarrhœa is present, and the ease with which the bowels are evacuated, and the freedom from all pain, deludes the victim into a belief that his ailments do not require attention, and that 'all will soon be right.' Occasionally the complaints first attract notice, from nausea and sickness coming on, and in other cases the patient experiences, at first, an acute attack of pain, either in the stomach or colon, or is seized (though more rarely) with cramps in the extremities. The diarrhœa, in some instances, is soon followed by vomiting ; in others, they both commence together. It is in these early stages that the complaint is certainly remediable under judicious treatment, variously adapted to the form of attack, the length of time which it may have existed, the rapidity of the progress which it has already made, and the condition and powers of the patient."

The authors divide the treatment of the premonitory symptoms into three parts—that necessary for the tormina—the feculent diarrhœa—and the serous diarrhœa. For the first, a proper evacuation of the bowels, followed by carminatives and anodynes, is sufficient. For the feculent diarrhœa, the same remedies will generally apply as if the epidemic did not exist, except that greater attention is necessary to check the disease in due time. An emetic is often useful in this stage, after which, opiates and astringents will usually be proper ; though the retention of vitiated secretions will often require warm, but efficient purgatives, combined with, or quickly followed by, light anodynes. Many cases, however, will occur, where the intestinal discharges must be stopped as quickly as possible, without regard to bad secretions, since the danger of collapse, by draining off the fluid and saline parts of the blood, is greater than any other danger. Opium, of course, is the sheet anchor in such cases. The authors of the volume before us give two grains to an adult, in the form of pill, for the first dose, and half a grain after every motion, till the discharges are restrained. Sometimes they combined a few grains of calomel with the first dose, but are not certain whether it added to the benefits of the opium alone. The purging being stopped, there is no need of soon opening the bowels again. A couple of days may be allowed.

When the serous diarrhœa has become associated with cramps and sickness, the most decisive treatment is necessary, in order to prevent collapse.

"To *stop the discharges* and *stimulate the flagging powers*, are now the immediate indications, and any means which risk the continuance of the purging are less preferable in proportion to the degree of such hazard. It has been said that the object is to *stop the discharges*, and nothing will effect this more, nay, so speedily, as *opium* in *full* and *efficient* doses.

The patient should be placed in bed between warm blankets and two grains of opium given, and followed immediately by a draught of the following nature:—

Sp. myrist. vel. sp. junip. comp. ʒx.

Sp. ammon. aromat. ........... ʒj.

Vin. opii ..................... ♏xx.

Misce.

If these are retained on the stomach, it will be proper to wait, lest, by giving more, vomiting should be induced; and it is better to trust, for one hour at least, to the above medicines (if they are not rejected), before any thing else is given. If they are returned from the stomach, it will be necessary to repeat them, and, if the purging continues, to administer the following enema:—

Decoct. amyli.. ℥j

Tr. opii. ...... ♏xl.

Vel. vini opii .. ʒss. ft. enema, which may be repeated likewise with half the quantity of the tr. or vin. opii, if expelled within a reasonable time. It occasionally happens that not more than one, sometimes not a single, evacuation follows after this, and the patient rallies from his depression; notwithstanding which, cramps will come on. These need not excite alarm, they will readily yield to hot fomentations, particularly if the system is influenced by opium. The case is henceforth easily managed, but cordials with small opiates should be continued."

It is hardly necessary to remark, that the "saline treatment" of Dr. Stevens is regarded by our authors as unnecessary in the early stage, and inefficient in the collapse of cholera. Under the head of collapse, our authors make many sensible and modest observations. They deplore the conflicting opinions and practices which have been published and urged, to the manifest injury of the medical profession. They justly observe, that too much is usually attempted in the stage of collapse. "Remedy has followed remedy, and dose has accumulated upon dose, in a manner more creditable to a sense of anxiety for the sick, than was consistent with sound professional judgment."

"In natural recovery from collapse, the warmth gradually returns to the features and extremities, the vomiting ceases, the purging diminishes, the cramps subside, the circulation is restored, the secretions of health return, and the individual gets well, sometimes immediately, and at others with the interruption of consecutive symptoms.

The design of medicine should obviously be to bring about these very desirable results. The indications of cure are at once simple and manifest, and not the less certain because human art is in a majority of instances totally incompetent to accomplish them. The indications of cure are simple, so ought to be the measures adopted to fulfil them; the only difficulty is to know what are the best means by which this is to be done.

It is very far from our inclination to attempt to solve this knotty problem. We do not venture to affirm, that such and such only is the '*sine quâ non*' of treatment; our sole object and desire being freely and candidly to state the result of personal practice; and we leave it to others to determine whether our views be deserving of notice or not.

According to these views, in collapse the patient should be dressed in a flannel shirt, and placed in a warm bed between blankets, with bottles of hot water to the feet and thighs; the head should be laid rather low, and a sufficient quantity of clothing thrown over the body to render him comfortably warm. In this respect his feelings are not to be altogether disregarded; if he complain of much heat, and the general temperature of the room be pretty warm—say 70° F. a single blanket and coverlid will suffice.

*Sinapisms* are valuable in collapse. A good large mustard poultice to cover the belly and chest, kept on till it has excited a redness, and can no longer be borne, and then removed to the back in like manner, is of great utility in bringing back pulse to the wrist, and in promoting the warmth of the surface. It also aids in allaying irritability of stomach, when that symptom exists. Mustard poultices require this caution in their application:—if kept on too long they distress the patient, and completely wear him out, so that more harm than good may be done by them; it is always better to remove them when they have excited moderate erythema. Under this simple precaution they may be used with benefit in all cases of collapse. The authors have seldom found it necessary to keep them on longer than half an hour at a time, or thereabouts. When, however the collapse is advanced, and the skin insensible, they must of course be retained until they produce some effect.

If the consequent erythema be of a bright colour, recovery is probable; if it be dingy and livid or leaden, death is almost certain.

We have never been satisfied of the utility of frictions: the limbs are mechanically warmed by this means, but they soon cool again; and we have seen no case in which restoration of pulse could fairly be attributed to it.*

Frictions unquestionably afford relief to cramps, and it is the common practice to rub the extremities when under this state; but by far the most effectual and grateful relief from cramp is obtained by hot fomentations, with a strong solution of salt in water, and by wrapping up the limbs in flannels soaked in that fluid. Tight ligatures also afford ease in a very marked manner, and may be used when salt and water is not convenient; but undoubtedly the best means by far is that of fomentation. Patients feel greater benefit and comfort from this than from any other part of the whole treatment.

From the time that a faltering of the pulse and other symptoms indicate the close approach of collapse the remedies recommended in the earlier stages are to be laid aside. Opium, which before was our sheet-anchor, will still stay the purging and quiet the cramps; but in large doses, it will now only do so by oppressing the nervous system, and crushing every effort at re-action. In collapse, large doses of opium are consequently injudicious. Our main reliance now, in the way of medicine, is to be placed in *minute* and stimulant doses of opium, combined with calomel. From two to five or six grains of the latter, and from one-sixth to one half-grain of the former, made into a pill, may be given every one, two, or three hours. If cramps be absent, and there be not much purging, the calomel may be given by itself without the opium; but when purging is profuse, and when cramps are severe—far from being injurious—opium in such doses has, in our observation, produced the happiest possible results. The object *now* is to moderate the purging, not to suddenly stop it; if therefore, notwithstanding this administration of calomel and opium, the purging goes on uncontrolled, it will be advisable to have recourse to additional means. We have

* "The pulse may often be brought back to the wrist, by merely placing the hands in a bowl of pretty warm water for a few minutes. In one case where the pulse was so brought back, the good effect was permanent."

found, under such circumstances, nothing so good as small enemata of brandy and water cold, with the addition of a few drops (twenty or thirty) of laudanum or vinum opii; this may be repeated every two hours, and the relief by which it has been followed has been the indication for its general adoption."

We have exceeded our intended limits in the notice of this little work, but really there is more practical information and good sense in it, than in many octavos of ten times the size.

---

### VI. Letter from a Friend, dated Bolton, 10th Oct. 1833, to a Gentleman in Dumfries.

"That a current of poisonous impregnation, of great intensity, has passed through your place, must evidently be allowed; but that its effects have been aggravated and extended, through the enervating influence of panic and personal fear, I, moreover, am led to conjecture. I have witnessed a good deal of cholera in three of the chief towns in Lancashire, and my experience leads me to the remark, that after a predisposition from real poverty, and intemperance in drinking, a dread of the disease is the most certain provocative of an attack; indeed in the cases of weak and nervous females, I should consider fear alone to be the most exciting cause during the prevalence more or less of the epidemic. I may also remark, that the dread of the pestilence in the South of Scotland is a prevailing epidemic of itself. In Galloway the mere rumour of a case, 40 miles distant, is sufficient to bring the segregated Members of a Board of Health to a speedy convocation, and spread alarm to the most sequestered cottage. To give an instance of this panic in educated people, a most respectable lady, living in a large mansion in Wigtownshire, lately caused all her family and house-servants to get into her carriage, on the instant her butler was seized with symptoms of cholera, and kept them sitting and waiting in the vehicles on a damp evening for two hours on the lawn, till post-horses could be brought from the town. On the contrary, I know a clergyman in Manchester, who continually went about visiting the cholera patients, and sat on their beds conversing with them in their dying moments, who never was the least affected; while his servant, who sat in his gig, and waited on him, but who had much dread of the disease, was seized, and narrowly escaped with his life. In this town, where we have not had above 40 cases, yet the people are decidedly sceptical, not that it does not exist, but against all measures taken for its prevention and cure; and the only patient that was attempted to be taken to the hospital, was forcibly stopt, taken from the porters to the neighbouring beer-shop, where they thrust warm beer, and toasted bread down his throat, and strange to say, he eventually recovered, without the future aid of the Faculty. I hope, therefore, as you seem inclined to do, you will cheer up your fellow-citizens, to put off the mortal evil of their fears, and put on the armour of confidence. It is this that shields the medical man, who is believed by the vulgar to possess a charm against it and fever, and about which I was asked seriously the other day by an otherwise intelligent patient."

---

### VII. Cholera-phobia in Ireland.

Let any one peruse the following extract from a paper of Dr. Howison, published in the Lancet of Nov. 10th, and say whether the terror of cholera is not one of the strongest predisponents to the disease?

"The effects of imagination on fear, during the epidemic cholera which prevailed in Ireland, amongst almost the greater number of individuals, were striking and surprising, and even the greatest minds gave themselves up to that impulse. On the morning when the death of a respectable banker became known in one of the large towns, almost every wealthy individual of the place imagined his turn was to come next, and for days gave themselves up to gloom and despair—many actually believing that they were labouring under the disease, and a considerable time elapsed before that disagreeable feeling eventually passed away. A wealthy individual, who felt within him an idea that he would fall a victim to the disease, actually left six different towns as the disease reached each one, and at length fell a victim to it in the seventh. Whilst I remained at Belfast, almost every night I felt a disagreeable sensation, which no effort could completely overcome. And during a whole night, whilst attacked with diarrhœa, from what cause I cannot distinctly say, whether a slight effect of the prevailing epidemic, or from living too well, or from cold, I never shut an eye. The melancholy state of the streets, deserted by the inhabitants, most of the few who remained being in mourning, hearses passing continually by day, and the noise of the wheels of cholera carts rattling at a gallop over the causeway, under the windows in the night time, carrying individuals to the hospital,—appalled the strongest minds. Almost in every large company during dinner parties, individuals arose from the table, imagining they were ill; and it was evident that the gaiety kept up by others was forced, and far from the heart. Such was the case in Ireland, the first country in the world for those merry and hospitable bursts of laughter which shake the sides. I feel no hesitation in believing and stating, that the best antidotes against cholera are nourishing, good, moderate, living, and having not the slightest fear of the disease. Individuals who possess these two good preventatives will escape it; but is it possible to instil the latter into those who at an after period may become its victims? I fear not. Is not the very circumstance of an individual feeling a presentiment haunting him day and night in all situations, a part of the disease.

So great was the terror of cholera at Londonderry, where it never had as yet reached whilst I was there, that dining with a family the same day on the morning of which the guard of the Sligo mail-coach had died of it, every other individual refused to go back for him, until a man was got to do so upon receiving double wages. If my memory do not fail me, it was stated at table that the mother and sister of the unfortunate man, the only individuals who resided with him, were also taken ill and had died since—a report which afterwards proved false, they having only been removed to a place of quarantine, and the house shut up. The master of the house, a very sensible literary man, actually wrote his will, brought it down stairs, before he sat down to dinner, and gave it to his wife, in my presence, and before his family, informing us that his feelings were, that as soon as the disease had made progress in the town he would be attacked. At that period and since, no other case followed, and I have no reason to doubt but that my friend has as yet escaped it."

---

### VIII. Dr. Thompson, of Belfast.

This gentleman, who has had 20 years experience, as a naval surgeon in various parts of the world, and also much in the late epidemic visitation of cholera in Belfast, has published a pamphlet, the second edition of which has just reached us. Into the controversy between him and Dr. M'Cormack we must decline entering. Their pamphlets are before the public in Belfast, where alone the conflicting statements can be fairly decided.* It appears, however, that Dr. Thompson's treatment was eminently successful, and that too, in the stages of collapse. We therefore readily give a summary of his plan in his own words.

---

* We are extremely sorry to observe the language that has been used in this controversy—more especially that employed by Dr. M'Cormack. Into the authenticity of the facts, it is not for us to go; but Dr. M'Cormack's language and temper, in the controversy, are exceedingly injurious to his cause. The general public, in medical controversies, will judge more by language than facts—and if, as Dr. M'Cormack insinuates, Dr. Thompson has employed a lawyer to revise his letters, it would have been beneficial to the character of the profession if he had done the same. —*Ed.*

"My first object is, to have my patient placed with his head very low, as in cases of syncope, from loss of blood, or from any other cause. I then determine to put a stop to the watery discharges *ab ano*. This, in some cases, will be found very difficult, but by perseverance it can, in general, be effected. The following I have found to be the quickest and most efficient remedy. I order a pint of arrow-root, a glass and-a-half of whiskey, eighty or one hundred drops of laudanum—all to be mixed together, and made sufficiently thin for an enema, and to be thrown up the intestines as warm as the patient can bear it. By this means I introduce nourishment, heat, medicine to allay inordinate action, and a very diffusive stimulus—being, at the same time, a powerful diuretic—all coming in contact with an extensive absorbent surface, viz., the mucous membrane of the intestines. Should this be retained, and when collapse has decidedly taken place, I repeat it in an hour, with or without the laudanum and whiskey, or with a smaller quantity of each, according to the symptoms. Should it be immediately dejected, as often happens, I repeat it as soon as another can be got ready; and if the second be returned immediately, I forthwith proceed to give a third, a fourth, a fifth, or any number, until one of them be retained, always taking particular care to keep the patient's head very low. In one of my cases, the patient appeared to be just expiring at the moment an enema was administered, but this was retained, and likewise the one previously given—so that by this time she had three glasses of whiskey, 160 drops of laudanum, and a quart of arrow-root. In this condition I left her, about six in the evening, convinced that I had done my duty, if she died the next moment; and, indeed, to judge from appearances, there could not be the slightest shadow of hope entertained of her recovery. Under these circumstances, and at the suggestion of her brother, as they lived some distance out of town, I made out an admission ticket, for her body to be received into the dead-house immediately she expired, which was momently looked for. To my great surprise and satisfaction, however, at eight o'clock on the following morning, unequivocal signs of reaction had taken place. The pulse could be found slightly fluttering at the wrist; there was a little warmth in the extremities, and the secretion of urine had been restored in a slight degree; the vomiting of every thing taken into the stomach continued for five days after this reaction. Three days after reaction, bile was thrown off, and the pulse became full and natural—but even then, if she raised her head, she became exceedingly faint. About this period, when the bile began to be secreted, I indulged the patient with a pillow, but I did not permit her to raise her head higher than the pillow for some time afterwards.

Reaction, in this case, can only be attributed to the two last enemas that were retained. I need scarcely state, that upon the least sign of resuscitation, the enemas were resumed, and continued for five days, which is the usual period in such cases, when the stomach begins to retain nourishment. They were generally administered every three hours, gradually diminishing the quantity of whiskey and laudanum, and sometimes leaving them out entirely, being guided, in this respect, by the pulse and other symptoms. Occasionally, a laxative enema has been substituted. I endeavoured to regulate the passage of the bile through its proper channel, by giving her small doses of calomel, but the effect was uniformly depressing. As to giving calomel in large doses, in collapsed cases, or in those approaching to that stage of the disease, or, indeed, in any dose under such circumstances, is, in my opinion, a mistaken practice, for at the time when it is my object to raise the pulse, it has the effect of depressing it still more. We are told by the advocates for the use of that medicine, that it is given to restore the secretion of bile, and all the other natural secretions. In my judgment, calomel requires a circulation to act upon; and, to be judiciously administered, it should be given before there has been too great an expenditure of serum, or after that and the circulation have been restored by other means. Two great objects I keep always in view, viz., that there is a deficiency of serum and animal heat, and that the blood being drained of its serum by watery evacuations, it becomes so inspissated, that it can no longer circulate, unless some means be devised to make up for this loss, or at least to put a stop to the further separation of this water, so necessary to the circulating mass; or, in other words, that the loss of a certain portion of the serum of the blood will occasion the same faintness that the loss of blood itself will produce. I believe my plan is the best calculated to answer the end in view. The stomach is always in too irritable a state to be the medium of introducing fluids, or any other thing, into the circulation; every thing is almost instantly rejected; while the intestines, much less irritable, afford, at the same time, a much more extensive surface for absorption —and by keeping the head low, the enemas will be retained. The exhalents of the intestines, by change of position, and pressure from the fluids introduced, will cease to pour out any more serum, and the blood now in the heart and large vessels, assisted by gravity (the head being low), and thinned by the fluids, will gradually find its way into the arteries of the brain, and produce that distention of them which, by its pressure and stimulus, is necessary to call the nervous system into action.

Soon after this the tip of the tongue, which before was cold as ice, gradually becomes warm. It is the nearest point to the source of the circulation and origin of the nerves, and will always be the first to receive heat.

With caution and perseverance in the injections, all will do well. The upper extremities will gradually regain the circulation and warmth; then the lower extremities; and after this, if there never had been a grain of calomel in the universe, it will soon be found that there will be a perfect secretion of bile, and a restoration of all the natural secretions. Only restore the circulation in full force, and it will in due time restore all the natural secretions."

---

### IX. "New Proofs of the Veracity of Contagionists."

This is the title of a short paper in our contemporary, the "Medical and Surgical Journal," for Nov. the 10th, 1832. It exhibits, in a striking light, the shifts to which a drowning party will have recourse, in articulo mortis! The language is somewhat personal.

*(Extract from the French Lancet.)*

"A medical Journal, in giving in its last number an account of a Memoir by Drs. Trompeo and Rolandis of Turin, on the cholera morbus of Paris, states, 'that one of the most remarkable circumstances cited by the authors in favour of contagion is, that of a certain number of mattress-makers, who, having been occupied in carding the wool of beds previously used by cholera patients, were, almost all, attacked with the disease,—but (says the journalist), I do not know where those gentlemen obtained their information, or whether it be true.'

They obtained it from the mouth of the grand master of contagion, M. Pariset, who positively affirms that it occurred at the Salpêtrière. As to this statement, we can assert that it is not true,—that, far from almost all the mattress-makers in question having been attacked, very few of them had the disease; and we do not see why they should have been more exempt from the epidemic influence than the other inhabitants of that vast establishment, who suffered greatly from cholera, not excepting the insane.

In a similar manner did Messrs. Pariset and Audouard assert, that the mattress-makers of Barcelona were almost all attacked with the yellow fever, as the consequence of their particular occupation, during the epidemic of 1821 in that city: but M. Chervin subsequently placed before the Academy of Medicine authentic declarations from the mattress-makers, proving that the statement of Messrs. Pariset and Audouard was utterly fabulous.

It is thus, nevertheless, that *Messieurs les Contagionistes* write the history of events, and enlighten governments which place confidence in them.

Why is it to be wondered at that our neighbours adopt the most rigorous and absurd mesaures against the pretended contagion of cholera, when we see certain paid agents of government become the apostles of that chimera, and, in their zeal for the cause which they defend, falsify the facts which pass before our eyes."

"The two persons referred to in the foregoing extract, Drs. Pariset and Audouard, have, from the nature of their employments, long proved the very great utility of quarantine regulations to themselves. How truly was it said last year by Magendie to Lord Auckland,—'If you wish, my Lord, to get rid of contagion, *pay no quarantine people.*' He told the same to the Prime Minister of France. What thousands of lives might have been saved had this advice been taken! In this country, the mischief arising from the fatuous decision, against evidence, of the committee of imbeciles first consulted, might have been stayed: events would have reached the public eye in a purer shape, instead of being artfully dressed up by a *grand-maitre,* with his tried lieutenant and a troop of mercenaries. Communities, not being foully swindled out of their common sense, would have been able to appreciate, very soon, the difference between a cause and a mere coincidence; the gates of the splendid public hospitals, which are such an honour to the country, would not have been closed against the sick, who as we well know, have perished, in many instances, for want of due accommodation and attendance. We are well aware that the most consummate art has been practised by a knot of fattening contagionists, to propagate through every possible channel their doctrines from this to other countries. In our last number, we gave proofs that the medical men in the United States seemed to be judging for themselves, however, unbiassed by management practised here. But far otherwise it seems to be, we are sorry to say, with the people at large there; for it would appear that, with them, the falsehoods propagated by some of the *employés* in this country, as to cholera being transportable by such things as the sails and ropes of a ship, &c. &c. have gained credence, and, as may be seen in an extract of a letter from Watertown, given in the Paris Medical Gazette of the 9th inst., the sick are, consequently, treated like wild beasts—'*On traite une pauvre malade comme une bête farouche!!*' A dungeon and bread and water for the remainder of their lives would be but inadequate punishment for the mischief inflicted on society every where by persons who have, from interested motives, been active in propagating the most fiendish doctrines."

---

### X. CHARCOAL IN CHOLERA.

IT appears that an old man of singular aspect, oddly attired, and wearing a long white beard, drove about the streets of Montreal, from morning till night, administering charcoal powder to cholera patients, and curing all to whom he gave it. Mr. Parkin, a young East India surgeon, has proposed, some months since, the same remedy in this country; but we believe that he had but a few opportunities of putting it to the test, and those not fair ones. Dr. Garrandan avers that he was successful with this remedy given by enema, in a dozen of cases continuously, in the department of Calais.

---

### XI. DR. WILLIAMS, OF IPSWICH.

IN our last number Dr. Williams' suppository (opium and soap) was mentioned without remark. We confess that we have little confidence in the remedy proposed, nor can we persuade ourselves, after what we have seen of real cholera, that any suppository can be "easily and comfortably received and *retained* in the bowels." *p.* 9. The Dr. declaims, with suitable vehemence, against the "awful fatality" which "may be attributed to the use of calomel, opium, &c." in cholera. We should hardly have expected that a learned "*fellow*" of the College of Physicians would have come forward, at this time of day, with a specific suppository for cholera, a disease which, like all others, requires different remedies in its different stages. At page 16 of his pamphlet, we find the learned Doctor animadverting upon some papers or letters published by Dr. Baird, of Ipswich; but into this controversy we mean not to enter. Not having a *nostrum* of our own to propose for cholera, we adhere to the good old rule, "non *nostrum* inter vos," &c. But in the same page, we observe a passage which is interesting beyond the confines of Ipswich.

"MEMBER OF THE ROYAL COLLEGE OF PHYSICIANS IN LONDON AND GRADUATE OF THE UNIVERSITY OF EDINBURGH. All membership of the College of Physicians, with the *honors* and *duties* attached to it, centre in the FELLOWS *alone*. DR. BAIRD's name is to be found amongst the *Licentiates only*.* The real or legal qualifications of a Licentiate, and those of a Graduate of the University of Edinburgh to *practice* as a Physician in ENGLAND, and *to what extent*, it is not my intention now to enter upon or discuss."

The passage and note are printed and Italicised precisely as they appear in Dr. Williams' pamphlet, and they exhibit an exquisite specimen of the feelings which some "FELLOWS" entertain towards those outcasts of the profession —the licentiates of the London college and the graduates of Edinburgh!! Admitting that a Blane, a Parry, a Gregory, an Abercrombie, were not worthy to lick the dust from this Ipswich "fellow's" shoes, what must the Babbingtons, M'Gregors, Prouts, Rogets, Hollands, &c. think of the dunghill from which they have sprung, and the brilliant *via lactea* into which they have been transplanted! We may venture to assert, that not a single "FELLOW" of the College in London would venture to publish such a libel on his fellow-practitioners here, even were he so inclined—and we are happy to say that very few indeed of them are so inclined, if we can judge of their character! It is in provincial towns only, that such narrow-minded and illiberal sentiments could be engendered or emitted.

* "Even in the appendix to the report of the committee of the House of Commons respecting the late Dr. Jenner's petition, the licentiates of the College of Physicians are designated licentiates only—*not members*—in proof of which, a brief extract from such appendix is here subjoined."—*p.* 17.

---

### LXVI.

### THE POISONOUS, OR TOAD-FISH OF VAN DIEMAN'S LAND.

*(Communicated by James Scott, Esq. R.N. Colonial Surg. at Hobart Town.)*

THE fish, of which I send you a delineation, is found in the bays and on the shores of Van Dieman's Land, and is supposed to be a species of the toad-fish.

The melancholy and dreadful effect produced by eating it was lately instanced, in the neighbourhood of Hobart Town, on the lady of one of the most respectable merchants and two children, who died in the course of three hours, without having been able to give any notice of their danger; and several servants (who had also partaken of the fish for dinner) were only saved by the timely discovery of the death of their mistress, and their fellow servant's children. The poison is of a powerful sedative nature, producing stupor, loss of speech deglutition vision, and the power of the voluntary muscles, and, ultimately, an entire deprivation of nervous power, and death.

At the inquest over the above bodies, the effect of the poison was satisfactorily proved, by giving part of the fish left by the unfortunate individuals to two cats, which soon became affected. When both were in a dying state, one had twenty-five drops of the arsenical solution introduced, with a silver tube, into the stomach, and rapidly recovered, while the other, which was allowed to take its chance, quickly died. The bodies, at death, were flaccid and blanched, with no fetor, but rather a smell like that of new hay, particularly about the mouth; but in about twelve hours, they became livid, swollen, with bloody serum issuing from all the external parts, intolerably fetid, and rapidly running into decomposition.

The general size of the fish is about five inches in length; the girth is great in proportion to the length; the back is of the colour and spotted like tortoiseshell; the belly is of a white kidskin feel and appearance; the animal has one ventral fin posterior to the anus, one caudal and two pectoral fins; the tail is perpendicular; the gills are anterior to the pectoral fins, and are about three-eighths of an inch in length, and of a semilunar form; the eyes are rather large and prominent, like those of the toad; the nares are anterior to the eyes.

*Hobart Town, Van Dieman's Land, 27th Dec.* 1831.

N.B. The specimen of the fish preserved in spirits, accompanying this account, has been presented to Sir W. Burnett, Knt., in trust for the museum at Haslar, by Dr. Sinclair, R.N.

---

### LXVII.

### ST. GEORGE'S HOSPITAL.

#### I. Excision of the Head of the Humerus, and Amputation of the Shoulder-Joint.

Within the last three years, two operations for the removal of the head of the os brachii, and one for amputation of the upper extremity at the shoulder-joint, have been performed at this hospital. All were for disease of the articulation. We will give the particulars of the cases, in order that some sort of comparative estimate may be formed of the value of the operation of excision and amputation. We are aware that such an estimate is by no means a complete one. It would require many cases, varieties of circumstances, and careful comparison, to warrant a decision.

In 1768, Mr. White, of Manchester, removed the head of the humerus through a vertical incision in the deltoid muscle. The case was one of that acute necrosis which surgeons occasionally witness, and which is not unfrequently fatal. Soon after the publication of Mr. White's case, a similar operation was performed by Mr. Bent, of Newcastle, and Mr. Orred, of Chester. In France, the elder Moreau performed the operation successfully in 1786; and the army surgeons, particularly Barons Percy and Larrey, frequently resorted to it, on account of recent wounds. Such is the brief history of the operation offered by Mr. Syme, in his Treatise on the Excision of Diseased Joints.

There can be no question, that it is owing chiefly to Mr. Syme himself that the operation has recently attracted much attention. The cases that he has published have been so numerous, some of them apparently so satisfactory, that it became impossible for unprejudiced men to disregard them. Mr. Syme has related two cases of excision of the head of the humerus for disease of the articulation. In the first case, the extremity of the acromion was "bare and rough;"—it was removed by the cutting pliers; the glenoid cavity was divested of its cartilage, but otherwise sound:—it was left undisturbed. This case ended favorably. When Mr. Syme last saw the patient, four years and a half had elapsed since the performance of the operation. She could move the limb across the chest, both forwards and backwards, with considerable force and freedom, but had very little power of abduction. The arm was about an inch shorter than the other. We think there can be no doubt that such a member is vastly better than none. The second case was not so favourable. At the time of the operation, the patient, a man, had symptoms of thoracic disease. The root of the coracoid process and upper part of the glenoid cavity were carious; they were freely removed by the cutting-pliers. In six weeks the wound was nearly healed. Then an old sinus, which ran along the suprà-spinous fossa, became larger—his pectoral complaints increased—and in six months after the operation he died.

The lungs were extensively affected. The extremity of the humerus was rounded off, and connected to the scapula by strong ligamentous bands. Nothing is said of disease still remaining in the scapula. Such was the issue of Mr. Syme's cases. It appears that in neither was the scapula extensively diseased, and perhaps it would be a fair induction from the first to suppose, that where the disease is confined, or nearly confined, to the head of the humerus, and no visceral alterations exist, the operation of excision offers a reasonable chance of success. We conceive that there is nothing in the cases we are now about to relate, which is calculated to disprove such a supposition. But we anticipate. We are sure that the only method of determining the value of this, or of any doubtful plan of treatment, is to lay before the public a candid and explicit detail of facts. They cheat the world, inflict a vital injury on science, and degrade themselves, who hastily publish their successful cases, but forget to recount their subsequent failures.

Case 1.—*Disease of the Shoulder-joint—Scapula implicated—Excision of the Head of the Humerus—Issue unfortunate.*

Sarah Jones, æt. 18, admitted March 2d, 1831, under Mr. Brodie.

General enlargement and globular form of left shoulder, commencing near the sternal end of the left clavicle, and increasing gradually to either border of the axilla, where the tumefaction is most marked. Integuments rather blue, with enlarged cutaneous veins. Swelling firm, save at the anterior border of the axilla, where a degree of puffiness almost raises the suspicion of the existence of deeply-seated matter. Deltoid wasted, as is the whole upper extremity, offering a striking contrast to the fulness of the thoracic portion of the shoulder. Over the centre of the left clavicle is the opening of a sinus, in which the probe passes beneath the clavicle towards the neck of the scapula and coracoid process. Other sinuses over the coracoid process—opposite the posterior surface of the neck of the scapula—at the lower and posterior border of the axilla, the latter apparently leading to the inferior costa of the scapula, below the neck. Over the anterior surface of the humerus, about two inches below the joint, the cicatrix of a sinus, and posteriorly another similar cicatrix. Much pain on pressure of the diseased parts, on employing the probe, or on attempting to move the joint. Arm retained in the position of approximation to the side, but not anchylosed, though incapable of much motion, from the condition of the soft parts, and the pain occasioned by such an attempt.

Complains of pain occasionally in the front of the chest—back of the shoulder—but chiefly in the side of the neck; the pain shoots down to the fingers. Pain worse by day than by night.

Health indifferent. She looks pallid, and older than she is—has slight cough at times, without expectoration—shooting and transient pains in the chest—some hectic—appetite good—bowels variable. She has never menstruated.

About six years ago, she received a slight blow upon the shoulder. Five years ago, a small swelling formed on the scapular part of the shoulder, and one year afterwards the present disease commenced. She entered this hospital, and Mr. Brodie, under whose care she was, applied a caustic issue. From that time to this, the disease has continued to make progress. A year or two ago, she refused to have the arm removed at the shoulder-joint, an operation which Mr. Brodie proposed. Has had startings at night, and a disposition to perspirations from the first. Cough has existed for a few weeks.

Such were the symptoms of the complaint, and such its history. The former we have given fully—they were evident facts. The latter we have related cursorily, for whoever has watched cases carefully, knows that long and complicated histories are always fallacious.

On the 28th, there was a consultation of the surgeons of the hospital. It was determined to cut down upon the joint, and, if possible, remove the diseased portions of bone.

A director was passed down a sinus to the bare part of the scapula, below its spine. A large scalpel was passed under the guidance of the director to the bone. The incision was then downwards and forwards to near the insertion of the deltoid—from this another incision was carried nearly directly upwards through the substance of the deltoid to the acromion, forming a flap of the posterior half of the deltoid. Flap raised—several large pieces of carious and dead bone with loose portions of the scapula removed with dressing forceps. Head of humerus then raised, carried outwards, sawed off with key-hole saw. Several vessels were tied during the operation—there was considerable venous bleeding—one vessel lying deep could not be tied: here blue-lint was introduced into the wound, and pressure made upon it. The soft parts were much thickened and altered. The flap was brought down, a single piece of lint introduced between the sides of the wound—light bandages supporting elbow.

On examining the excised head it was found nearly destroyed by caries, which extended for an inch, or so, down the inner side of the neck. About an inch and half of the shaft had been removed; this was white, apparently dead, with no periosteum on it. The shell of bone was not a line thick—the medulla pale, soft as jelly—the diameter of the whole shaft not exceeding half an inch.

We shall not enter much into detail with this case, but content ourselves with mentioning the more prominent features of it.

An opiate was given immediately after the operation and repeated at 4, p. m. On the 30th she was ordered beef-tea, and on the 1st April half a pint of porter daily. The pulse before the operation had been about 100. On the day afterwards it was 148, from which it gradually diminished to 120, which it was on the 1st April. The patient was of course weak and irritable. In the evening of the 3d April the little and ring finger, and the ulnar side of the hand of the limb that had been operated on, presented a dull erythematous blush, and were extremely tender when touched. The patient was unusually irritable. On the following morning all the fingers of the affected hand were blue at their extremities, rather painful, not cold; the hand was blue also. The pulse in the radial artery of this side was less distinct than in the other limb. Some sponge which had been passed into the wound came out, but the blue lint remained. The wound looked pallid and glassy.

The patient remained for some time in a precarious condition. The fingers of the affected side continued blue, and became cold and numbed; no pulse could be felt in the radial or ulnar arteries; and for some days there was every appearance of gangrene being about to invade the limb. By the application of warm flannels, &c. the circulation was slowly restored. A great deal of irritation was occasioned by the retention of some blue lint in the wound. Some sloughing took place, and it continued angry and troublesome for a length of time The dressings were light and calculated to support the limb, the constitutional treatment suited to the character of the symptoms.

The following is a report which we made on the 1st October, upwards of six months after the operation.

The wound itself is healed, but ulcerated openings and sinuses still remain in various places, evidently pointing out the existence of disease of the scapula. Fresh abscesses form every now and then, and are attended with febrile exacerbations. Her health is indifferent —her appearance much as before the operation.

The arm itself hangs powerless by the side; it is supported in a sling contrived for the purpose; she has little or no power of motion, except in the fingers.

We regret to say that the patient is now, December, 1832, dying. Symptoms of thoracic disease have become established. We know not that we add much in any way to our description of the state of the limb written in October 1831.

CASE 2. *Disease of the Humerus and*

*Scapula—Excision of the Head of the former.*

We have unfortunately lost our notes of this case, and must therefore give a brief memorandum of it from memory.

The patient, a huge clodhopper from Uxbridge, about 40 years of age, was received into the hospital under Mr. Babington. He had disease of the right shoulder-joint, but how he got it we have no conception. There were various sinuses leading some towards the head of the humerus, some towards the scapula about its neck and below its spine; the precise situation and extent of the diseased bone were of course uncertain. The limb was anchylosed, but admitted of little motion from the consolidation of the soft parts. The constitutional symptoms were trivial.

Mr. Babington excised the head of the humerus and a portion of its shaft. Whether he removed any of the scapula we cannot positively say; we rather think he removed none. No constitutional or local disturbance of consequence ensued. The operation was performed prior to the occurrence of the preceding case. The condition of the patient at present is, so far as we can learn, as follows:*

The arm is shorter than the other; the fore-arm is usually retained flexed at a right angle, or nearly so, upon the arm. He can move the arm forwards and backwards on the chest, can support a not inconsiderable weight in the hand, and has some power of abduction of the arm from the side. This latter motion is of course the most limited. There is still some disease about the scapula, a sinus opening every now and then, indeed we believe there is one or other always open. His health is good.

Case 3. *Disease of the Left Humerus and Scapula. Amputation at the Shoulder-Joint.*

This case has been very well reported by an excellent friend of ours in the Medical Gazette. We shall make short work of it.

James Spawforth, æt. 22, admitted July 11th, 1832, under the care of Mr. Brodie.

Left arm hanging powerless by the side—wasted—nearly motionless at the shoulder-joint—much pain in that articulation on attempting to move the arm—little pain in it when at rest. Around the shoulder-joint the openings of seven or eight sinuses, chiefly on the anterior and posterior margins of the deltoid, the central portion of which is consequently free from them—the openings of two or three situated immediately below the scapula spine, to which and to the anterior part of the infra-spinal fossa they would seem to lead: here the probe reaches exposed bone—the anterior appear to lead towards the coracoid process and neck of the scapula. The whole aspect of the shoulder is that of diseased bone of scapula and humerus, with ulceration of the cartilages, &c. of the joint.

Aspect pallid, unhealthy—not much emaciation—no cough at present—little hectic—pulse usually 96.

Two years and a half ago he struck his shoulder. In a month it became stiff and somewhat painful. Two months after this Dr. Gordon and Mr. Guthrie applied leeches, blisters, and caustic issues with great benefit. Partial anchylosis would seem to have taken place, and discontented with his lot, he applied to a country bone-setter, who pulled and tugged at the shoulder-joint, and told him all was right.* An abscess soon followed, presented in front of the articulation, and was opened. Since that time several abscesses have formed and burst, and given origin to the sinuses. He has been in this hospital four months, and taken some sarsa and nitric acid to improve his health. Three months ago, whilst in the house, he had a severe attack of what we are told was "bronchitis." He has lived intemperately.

On the 22d November the arm was

---

* He attends occasionally as an out-patient.

* In one of Mr. Syme's cases the same thing occurred. If fools will seek the aid of scoundrels, they must take the consequences.

removed at the shoulder-joint. We need not be minute in our account of the operation.

The subclavian was commanded by the pressure of an assistant. A flap was made from the deltoid by a hermaphrodite sort of instrument, between a scalpel and an amputating knife. It was formed by two incisions, one commencing behind the posterior margin of the acromion and terminating near the insertion of the deltoid, the other begun anterior to the acromion and carried down to meet the former. The flap was raised, and the consolidation of the parts rendered this less easy than might be supposed—the head of the bone tilted forwards, upwards, outwards, by an assistant holding the elbow—the articulation opened by an amputating knife directed horizontally—the instrument made to coast the head of the bone—the limb severed from the body by one swoop, by turning the edge of the knife downwards, or axillad, and outwards or towards the arm. From this it necessarily resulted that a lower flap, less than the upper was preserved. Imagine this done with decision and dispatch in the midst of the most furious and uncontrollable struggles of the patient, struggles so violent as to make the assistant who pressed on the subclavian all but faint in his efforts to maintain his pressure—imagine this, and the reader will have a far better notion of the operation, than any description of our's would give him.

The lad lost about eight ounces of blood, perhaps more. He was faint and required wine. The vessels were secured as quickly as possible. The axillary, we believe, bled smartly.

It was deliberated whether any of the scapula should be removed. The surface of the glenoid cavity—a portion of the neck below, before, behind it—a portion also of the spine were found by examination to be diseased. In making this examination a cavity near the cervix anteriorly was broken into, and much curdled pus escaped. The restiveness of the patient, his exhaustion, the extent of scapula diseased, decided the consultants not to protract the operation. Some lint was introduced between the edges of the flaps—an opening to the bone maintained—and the dressings completed by light adhesive straps and a roller.

The upper extremity of the bone was sawed vertically in halves. The articulating cartilage was more or less destroyed by ulceration. At the centre of the head the ulceration extended through the shell of bone for a few lines into the cancelli which here were carious. The remaining cancelli of the head, and cancelli of the shaft for two or three inches from the head, were occupied by a yellow unorganised substance, seen in the cancelli of scrofulous bones; below this deposition the cancelli were soft, pulpy, vascular.

Nothing of any consequence has occurred since the operation. The patient has now the ordinary diet, and goes about the ward. On Dec. 7th, Mr. Brodie broke up some adhesions between the posterior margins of the stump, and introduced lint to secure a future opening. The object is, of course, to obtain a free passage for the exit or removal of diseased bone.

Mr. Brodie made some clinical remarks upon the case. After alluding to the particulars of the case, he observed that it had been determined in consultation to amputate at the shoulder-joint, and, if possible, remove the diseased portions of scapula. Mr. B. did not excise the joint, in consequence of the result of the two cases, already related in this report. Having removed the arm, he had not proceeded with the operation, for the reasons we stated in the report. By keeping the wound moderately open, an opportunity would be afforded for the exfoliation of the diseased bone, and as all of it had been fully ascertained and exposed by the finger, there was a fair chance of ultimate recovery, provided the patient's constitution was equal to the effort required. In the course of his remarks Mr. Brodie alluded to the case of a young gentleman whose arm he had removed at the shoulder-joint. In that case there was disease of the articulation, yet not sufficient to prevent subsequent anchylosis and recovery. It was necessary however to amputate in consequence of repeated hæmorrhages that occurred and were seriously injuring the patient's health. This patient did well and continues so.

### II. Malignant Tumour in the Calf of the Leg.

We have seen three cases of this description, two occurring at this, the third at the Westminster Hospital. We do not intend to become reporter for that institution, yet we venture, for the sake of illustrating the subject, to mention the particulars of the case we saw there.

Case 1. *Tumour in the Calf of the Leg — Amputation—Death from Inflammation of the Vein and purulent deposite in the right Elbow-joint.*

Susan Walker, æt. 38, a laundress in a gentleman's family, admitted October 3rd, 1832, under the care of Mr. Keate.

In the calf of the right leg a solid oblong tumour, rounded and nearly uniform on its surface, commencing nearly opposite the insertion of the sartorius into the tibia, and extending downwards to nearly opposite the insertion of the fleshy fibres of the solæus into the tendo Achillis, so that the general form of the calf appears merely exaggerated. Tumour hard, resisting, saving on its inner and posterior part, where it is rather elastic. Tumour moveable upon the bone—gastrocnemius and solæus apparently spread over it—skin moveable upon the tumour. No perceptible enlargement of inguinal or other absorbent glands—veins both of affected and other leg varicose. Pain extending in the course of the posterior tibial nerve to the sole and outside of the foot, occasionally along the sciatic nerve to the hip—pain aggravated by walking, and worse at night. Tenderness on firm pressure of the tumour.

Says that her health is good—functions performed pretty regularly—occasionally pain in the back—aspect sallow—slight emaciation.

Two years ago first observed pain in the foot, which was considered rheumatic. There might or there might not have been a swelling in the calf for it was not examined. Various means were used with little benefit. She first observed the tumour about 15 months ago; it was then about half its present size, hard, rather painful. From that time the tumour has increased, though not progressively. She seems to have taken some mercury. She has been unable to follow her occupation for the last six or eight months.

On the 10th Mr. Keate punctured the inner part of the tumour with a needle, and serum issuing, plunged in a small trocar; three or four ounces of yellow serum escaped. The puncture healed. Iodine, in the form of ointment, was tried; it irritated the skin and was discontinued on the 24th. In consultation it was determined to amputate the limb. The operation was performed by Mr. Keate on the 25th. An incision having previously been made into the tumour to ascertain the nature of its structure, and that appearing medullary, the limb was amputated above the knee.

On examining the limb after amputation, the tumour was found to be situated between the gastrocnemii and the deep muscles, to which it was more or less adherent above and below. The posterior tibial nerve was enlarged and spread out over the posterior part of the tumour above, and seemed involved in the tumour at its middle. The tumour was enveloped in a cyst one-fourth of an inch in thickness. The structure of the tumour was peculiar; it resembled in parts firm medullary sarcoma, especially that which grows from bone or fibrous membrane; it was chiefly composed of a more "friable" opaque material, of dirty white colour, not unlike the adipocire of a limb macerated with much flesh on it; it displayed cellular partitions; it was little organized.

Opiates were required after the operation. Next day there was slight pyrexia, for which she had salines with antimony. On the 27th she complained of pain in the loins and left iliac region. She attributed it to position, and on altering it she felt relieved. The bowels being constipated she was ordered aperient medicine.

*Vesp.* Countenance rather anxious with slight flush — fidgettiness — says she is free from pain—pulse 120, rather sharp—bowels have not acted.

28th. Bowels acted last night, and she slept for two hours this morning. At 3, a. m. she was sick. Some pain in the left iliac region, and whole left lower extremity tender to the touch—slight discharge of brown thin matter from the stump, and issue of a few bubbles of air.

*Straps loosened — simple dressing — cold lotion. Salines with hyosciamus.*

Towards evening she was sick and restless, with pulse 130. The sickness was relieved by two doses of saline draught with liq. op. sed. ℞x.

29th. Restless — anxious — says her head feels light. Pulse 130, sharp, yet without strength—skin warm—bowels confined. Sparing, dark discharge from stump.

*Strapping further loosened. Camphor mixture with ammonia.*

*Vesp.* Occasional wandering delirium.

Strapping removed — adhesions, which were partial, broken up—sloughy cavity of stump exposed. *Warm dressing and poultices. Dover's powder in saline.*

On the 30th she had some aperient medicines which brought away some dark and offensive motions.

31st. Manner hurried—aspect rather collapsed and sallow. Pain on pressure over the iliac vessels on the side of the stump—tenderness also on pressure of the calf of the opposite side. In the evening she was delirious and very restless—her aspect was pallid, anxious—her skin bedewed with perspiration. There was now extreme tenderness on touching the right elbow, the skin on the outer side of which had the slightest erythematous blush, whilst the subcutaneous cellular tissue was infiltrated with serum.

She was ordered camphor mixture with morphia and tincture of hops. She had been allowed for some days beef-tea and arrow-root.

In the morning of Nov. 1st she was much the same. Pulse 130,—tongue dryish, streaked — thirst. The stump was covered with an ash-coloured slough—the limbs trembled.

*Vesp.* Raving delirium. She screeches, sings, shouts, laughs wildly at the bye-standers. She awoke from sleep in a profuse perspiration. Escape of two ozs. of blood from a vessel, the ligature on which was accidentally pulled.

She continued delirious till 4, a. m. of the following morning—then sank exhausted into sleep, and so remained for two hours—awoke—was sensible—soon afterwards became lethargic—and died at 8, p. m.

*Dissection.* The head was not examined. There was nothing particular in the thorax or abdomen, with the exception of the spleen, on the surface of which were some common tubercular deposits.

*Femoral Vein.* Traces of inflammation of its coats, which, for the space of half an inch, were invested with recent lymph. Vein partially filled with coagulum.

*Right Elbow-joint.* Serum in the subcutaneous cellular tissue. Pus in the cavity of the articulation, without ulceration of the cartilage.

*Left Leg.* Serum effused between the muscles—No pus discovered.

To the details of this case we would direct attention. They are the representatives of a class. Such are the symptoms, and so occurring, which mark inflammation of the veins, or deep, diffuse inflammation of the intermuscular cellular tissue, or even the purulent depôts in the viscera, or in other parts of the body, though, indeed, the depôts ordinarily occur at a later period.

Case 2.—*Tumour in the Calf of the Leg—Amputation—Death.*

About three years ago, we saw a nearly similar case at the Westminster Hospital. The patient was a middle-aged woman, under the care of Mr. Guthrie. The tumour was situated higher in the calf than in the preceding case, and passed into the inferior portion of the popliteal space. Its history we do not recollect; but we do remember that, in its external characters, its apparent consistence, &c. it resembled the case we have related. The woman was thin, and looked ill.

Mr. Guthrie amputated the limb above the knee. No tourniquet was used, Mr. White compressing the artery below the groin. We never saw less blood lost in an amputation ; but perhaps all assistants may not be so good as Mr. White, nor all operators so dexterous as Mr. Guthrie. However this may be, the operation was performed with the dexterity for which Mr. Guthrie is celebrated, and very little blood was lost. The woman died about a fortnight after the operation. We were informed that she had phlebitis.

The tumor was of much such a structure as in the case of Susan Walker. It presented an ill-organized crumbling mass, of a dirty-white colour, not unlike adipocire. The gastrocnemii were spread over it. The precise tissue from which it grew was uncertain ; in this case, the periosteum was separated from the upper portion of the fibula, but the bone itself was not involved in the disease.

Case 3.—*Tumour in the Calf—Death.*

We remember witnessing, some six years ago, the dissection of a similar case in this hospital. The patient was under the care of the late Mr. Jeffreys. The tumour was in the calf. The gastrocnemii were spread over it. The man died, but whether amputation was performed we cannot say. Our impression is, that the man died of phlebitis, from meddling in some way with the tumour, but that amputation was not performed. In the remarks of Mr. Hey upon fungus hæmatodes, is a case exactly in point ; it is his fifth case. We will give it in that excellent practical surgeon's words.

*Case.* "A boy, about fourteen years old, was admitted an in-patient of the General Infirmary, on account of a large deep-seated tumour in the calf of his leg. The cause of this disorder he judged to have been a sprain, from a sudden and violent exertion ; for, soon after this accident, he perceived the calf of the diseased leg to be larger than the other. The tumour had continued to increase during six months, and he was now rendered very lame by it.

It was impossible to ascertain, with precision, either the situation or nature of this tumour. It was clearly situated betwixt the gastrocnemius muscle, and the bones of the leg, and might have its origin near the latter ; so that an attempt to extirpate it by incision was out of the question. There was no pulsation in the tumour ; nor any discolouration in the integuments. The accident which had preceded the appearance of this tumour rather indicated that it had arisen from the rupture of some vessels in the leg.

Upon a consultation, no probable method of cure was suggested but that of amputation ; and, the parents of the boy giving their consent, I performed the operation above the knee.

After the operation I dissected the leg, and found the tumour to consist of a substance similar to that which I have described in the preceding cases, situated between the gastrocnemius and solæus muscle, and extending a little before their edge on the outer side of the leg. Wherever this substance lay in contact with the muscular fibres, they were of a brown colour, and had lost their usual distinct appearance. We could perceive no ruptured vessel; but the lymphatics were not injected.

The patient had a good recovery."

The case must have occurred between 1789 and 1793, and our edition of Mr. Hey's work is dated 1810. Our readers must judge for themselves as to the probability of the recovery having been as permanent as good ; but we may remark, that the complexion of many of Mr. Hey's cases is somewhat too favorable for modern experience. At the time Mr. Hey wrote, the visceral contaminations, following operations on external malignant tumours, were not recognized.

It would seem that, in all these cases, the tumor was seated between the superficial and deep muscles of the back of the leg—that it probably grew from the cellular tissue between them, or from the fascia covering the deeper set—that, in its characters, it approaches nearer to fungus hæmatodes than any other morbid growth—and, from the event of three of the cases, that it is a very dangerous disease.

### III. OBSTINATE ULCERATION IN THE GROIN.

We think the following cases not devoid of practical utility. It is true that they are not all of a similar description. and it is not strictly philosophical to bring them thus together in a lump. But this may be excused in a rambling report like the present.

CASE 1.—*Obstinate Ulceration in the Groin—Death from Coma—Sinus found leading to bare Os Pubis.*

This patient was under the care of Mr. Walker, surgeon of the Lock Hospital, and was in that establishment. He was a Greek courier, about 30 years of age, or somewhat older, apparently broken in health, and of an irritable habit.

In the commissure of the right groin was an indolent ulceration—skin around the ulceration rather elevated, warty, whitish, semi-organized—sore not very deep, irregular, with dull red, glassy granulations, and islets of cuticle here and there—sore very irritable and painful. Pulse always frequent—skin rather warm.

This sore had existed for some time, little benefitted by treatment. We saw the oxymuriate employed, but the ulcer became worse under its use. Various other means were employed, and in the latter end of February he seemed better, and was allowed to get out of bed. Two or three days afterwards, the house-surgeon, Mr. Clarke, was called to him suddenly, and found him in a state of coma. He soon died.

DISSECTION. *Cranium.*—A considerable quantity of serum in the ventricles of the brain, which seemed enlarged, and the white substance forming their boundaries was soft. Substance and membranes of the brain perhaps rather more vascular than natural.

*Thorax and Abdomen.*—Nothing particular.

*Sore.* This extended into the adipose and cellular tissues, and seemed to involve, though not to penetrate, the fascia lata, particularly that portion covering the pectinæus muscle. From the sore a sinus extended inwards in the cellular membrane above the fascia, then penetrated the latter, and passed to the tubercle of the os pubis, which was carious. There was not great induration around the sore.

CASE 2. *Obstinate Ulceration between the Scrotum and Thigh—cure.*

A man, young, and though of scrofulous aspect, not otherwise unhealthy in appearance, had been an in-patient of St. George's under Mr. Brodie for six or seven months, when we had the honour of becoming house-surgeon to that gentleman. A long, dangling, irregular sore occupied the space between the right side of the scrotum and the right thigh. It had a very similar appearance to that of the sore in the preceding case — the same high, and, as it were, wetted and warty margins—the same glassy, dull red, small granulations. Skin occasionally formed and again ulcerated. He was put on porter, more generous diet, sulphate of iron and other tonics, with local stimulants, as the tinct. benz. comp. &c. The improvement was soon remarkable, and though it did not continue uninterruptedly, the cure was completed in the course of a month or two. Once or twice the sides of the sore became united superficially, while a sort of sinus or gutter ran beneath. It was then necessary to break up the new skin and begin afresh. After cicatrization the scrotum was puckered into the thigh.

We are not sufficiently acquainted with the early history of either of the preceding cases to warrant us in saying any thing about it. We would be as accurate as possible. Where we entertain any doubt we express it.

CASE 3. *Sore on the Penis — Mercury used — Phagadenic ulceration in the Groin—Tonics.*

A young man, from Putney, was admitted into the hospital on the 21st Feb. 1832, under Mr. Hawkins.

In the right groin was a large, flat, irregular ulceration — its edges rather undermined, blueish red—its surface of a dirty yellow colour, with here and there slight ecchymoses—ulcer extending into the cellular membrane, but not apparently through the fascia—no diseased gland observable—much pain in the sore. Looks ill—face pallid—tongue slightly furred—pulse frequent, irritable.

Three months previously he contracted a sore upon the penis. In a fortnight after that a bubo appeared in the right groin. In five or six days after that he went to Mr. Shillito, a very respectable surgeon at Putney, who gave him mercurial pills, and kept his mouth sore for five or six weeks. The quantity of mercury taken daily was varied a little, according to the state of the mouth. At the end of this course the sore was healed ; its exact time of healing we do not know. In a week or ten days after commencing the mercury "the bubo broke." When the mercury was discontinued there was a sore in this situation larger than a crown-piece. Various draughts were taken, but his health became more severely implicated and the sore increased. Under these circumstances he entered the hospital.

25th. *Hyd. sub.* gr. v. *h. s. H. sennæ cras.*

26th. Sore spreading—very low.

*Mist. camph. c. Ammon. carb.* gr. vii. *Tinct. opii,* ♏x. *6tis. horis. Cat. douci. Ol. ric. cras.*

*March 1st.* Sore spreading downwards on the thigh and inwards towards the perinæum. Constitutional symptoms as before. Looks pinched and anxious.

*Tinct opii,* ♏xx. *in H. ol. ric.*

3rd. *Cat. lini. c. Liq. plumb. acet. Cal. pulv. ant.* āā. gr. iij.—*Pulv. opii,* gr. j. *h. s. s.*

6th. *Lot. acid. nit.* (gtt. ij, ad aq. ℥j.) *H. Sennæ cras. Rep. H. 4tis. hor. c. Tr. opii,* ♏xxv. *Vini rubri,* ℥iv.

8th. Sore has spread very considerably in extent, and is now larger than the palm of one's hand. Sore of the same character as before—not materially increased in depth. Pulse frequent, weak — tongue moist, whitish — looks pinched and blueish.

*Rep. Haust. c. Tr. opii,* ♏xxx. *Mist, camph. Dec. cinch.* āā. ʒvi. *Conf. arom.* ℈j. *Ammon. carb.* gr. v. *Tr. opii,* ♏xl. *6tis. hor. Vini rubri,* ℥viii. *Ol. ric. cras.*

10th. Improved—granulations in the sore, which has ceased to spread, and in parts shews florid edges. Looks better.

*Hyd. sub.* gr. ij. *Opii,* gr. iss. *o. n. H. Sennæ stat.*

19th. *Rep. Haust. c. Tinct. opii,* ♏ xviii. *tanto.*

23d. *Hyd. sub.* gr. j. *Opii,* gr. ½. *o. n.*

25th. Sore healing rapidly — health much improved.

*H. cinch. c. Ammon. carb.* gr. v. *Conf. arom.* ℈j. *t. d. s. Cerevis. fort.* Oj. *quotid. Haust. anod. o. n.*

*Om. alia medica. et vinum.*

On the 17th the ulcer was nearly healed ; on the 24th it was quite so. We should state that the diet in the first instance consisted of beef-tea, arrow root, and food of that description, and subsequently of animal food, when the patient's appetite was equal to it.

Our narrow limits prevent us from noticing some other cases, which we had intended to report.

---

LXVIII.

PART V. OF THE ANATOMICAL ATLAS OF DR. M. J. WEBER, Professor at the Royal Prussian University, Frederick William, at Bonn.

WE have noticed with much commendation the previous parts of this anatomical atlas. In our favourable opinion of it we have not stood alone, many of our best anatomical teachers and surgeons having given their testimony to its merits. The present part consists of 13 folio plates, the representations being, in most instances, nearly as large as life, and in many larger. Some are lithographic—others, for instance, the delineations of visceral structure and of the nerves, are engravings. The vessels are coloured. The Part displays the whole anatomy of the ligaments—two plates, from Scarpa, of the Nerves of the Neck and Chest, and three Plates, displaying the anatomy of the male generative and urinary organs, and the female generative organs and mamma. We can speak of them in very commendatory terms, and not less commendatory than merited. We may give a sample of the whole, by mentioning the manner in which the ligaments are delineated. An anterior and a posterior view being given, separate views of the individual ligaments are added, and thus a complete representation is presented. The plates are accompanied by a letter-press explanation, in German and in English. The price of each part is one guinea. We beg again to recommend the work to those who are anxious to acquire, or to maintain, their anatomical knowledge.

---

### LXIX.

PRINCIPLES AND ILLUSTRATIONS OF MORBID ANATOMY, ADAPTED TO THE ELEMENTS OF M. ANDRAL AND TO THE CYCLOPÆDIA OF PRACTICAL MEDICINE, WITH WHICH IT WILL CORRESPOND IN SIZE; BEING A COMPLETE SERIES OF COLORED LITHOGRAPHIC DRAWINGS, FROM ORIGINALS BY THE AUTHOR; WITH DESCRIPTIONS, AND SUMMARY ALLUSIONS TO CASES, SYMPTOMS, TREATMENT, &c. DESIGNED TO CONSTITUTE AN APPENDIX TO WORKS ON THE PRACTICE OF PHYSIC, AND TO FACILITATE THE STUDY OF MORBID ANATOMY IN CONNEXION WITH SYMPTOMS. By J. HOPE, M.D. F.R.S. Physician to the St. Marylebone Infirmary, &c. London, Whittaker and Co. 1833.

THE following is the plan on which this work is to be conducted.

" The work will consist of Twelve Monthly Numbers, each containing 25, or more, highly finished, coloured Lithographic Drawings, from originals by the Author, executed with a view to the exhibition of the minute structure, no less than of the general characters of the several morbid alterations. The quantity of Letter-press will be adapted to the subject. The size Royal Octavo.

The arrangement will be according to organs, as being best adapted to the study of the diseases of organs, but the lesions of each organ will be considered in reference to the particular tissues which they occupy on the principles of General Anatomy.

The descriptions will be, for the most part, copies of those taken from the original specimens; but reference will be made, where necessary, to the corresponding descriptions of Andral, of other authors, and of the Cyclopædia.

The Author having, with few exceptions, taken the histories of the cases personally, he will, from the funds thus supplied, render the Work as practical as is compatible with its character, adverting very succinctly to the nature of each instructive case, and to the connexion between the lesion and the symptoms.

The price of each Number, coloured, will be Eight Shillings and Sixpence; and the several subjects will be complete in their respective Numbers, in order that, if desired, a portion only of the Work may be accessible to purchasers."

Such is the plan of the work. By the kindness of Dr. Hope, the first part is now before us, although it will not be actually published until the 1st of Jan. 1833.

On the importance of morbid anatomy we need say nothing; neither need we stop to discuss the extent to which it may be carried with advantage. We are not among those who anticipate evil from the zeal with which this portion of medical science is studied. False theories and brilliant hypotheses may be prejudicial to the cause of truth; the pursuit of exact investigation never can be so. Individuals may be led away, may be rendered ridiculously sceptical or overweeningly arrogant, according to the state of mind in which they examine and argue on facts; but exact knowledge, we repeat, can never be extensively or permanently injurious. Morbid anatomy has been as the spear of Ithuriel to medicine; has disclosed what is demonstrable and what is vague; and given, and will give, to our science all of the genuine inductive character of which it is susceptible. We hail, then, the appearance of good works on morbid anatomy with unfeigned gratification, and we feel assured that they will be productive of that best of all effects, the *general* diffusion of scientific information.

At this late period of the quarter we can do little more than direct attention to the work before us. Let us look at it a little in detail.

The present, or first part consists of four lithographed coloured plates, and 23 pages of letter-press. The subject to which it is devoted is pulmonary disease, and the particular affections delineated and described are—acute and chronic peripneumony—gangrene of the lungs—abscess of the lungs—and pleurisy.

Each plate contains several figures, severally representing particular lesions or varieties of lesions. The size of the plate is, as has been mentioned, what is technically termed royal octavo, that is, about the size of the Cyclopædia of Practical Medicine. The size of the particular figures varies according to the character of the lesion; thus a drawing of gangrene of the lung occupies nearly two-thirds of the first plate, whilst Fig. 19, shewing tubercles passing from the grey to the yellow state, is not an inch square.

The execution is extremely good—the outline clear—the tints distinct yet not overcharged—the character of the lesion defined yet not exaggerated. As drawings, these delineations possess high merit, and yet that almost irrepressible sense of the beautiful, the *τοκαλον*, which animates all who pursue the lovely art, has not seduced Dr. Hope into sacrificing truth for its sake.

The present part contains 30 figures. Amongst the best we would venture to point out the representations of diffuse gangrene of the lung—purulent infiltration—pulmonary granulations of Bayle—cavern in the lung with bands—and some of the delineations of tubercle. All lesions are not represented with equal facility; those offer least difficulties in which the tints are most uniform and present most body, or in which the contrasts are definite and decided. Thus the lobular hepatization seen on the pleura, and the purulent infiltration in its most advanced stage, (figs. 9 and 8,) are so characteristic, that they would convey to the mind of the most inexperienced student an image, the reality of which would be immediately recognized when the actual lesion came before him. We do not think the purulent deposites after injuries (fig. 10) so plainly drawn, and some of the designs of tubercular infiltration do not appear to us to have that instantaneous force of truth, possessed by those we have particularly commended. But—

> "He jests at scars who never felt a wound;"

and the critic in his easy chair may point out defects that all the limners's art could never rectify.

The price of each part is eight shillings and sixpence, and the general feeling will be one of surprise at the extreme cheapness. We know that Dr. Hope can receive no pecuniary remuneration from the work. He labours for fame. Dr. Hope is right. At the present period it would be utter folly to expect that a work which could *pay* would *sell.* The man who embarks in such undertakings as these must be content with the solid reputation which merit will confer upon him, and the indirect emolument that reputation will draw after it. It must not be the speculation of a bookseller; it must be the careful anxious production of an able and industrious man, willing to take the chance of ultimate advantage, or, even if that fail, not utterly disappointed at obtaining the solitary recompence of well-earned, and freely accorded fame. This may be discouraging to mere speculators. We do not care if it be so. We are sure that it is not discouraging to him of a true philosophic spirit, a spirit, we regret to say, more prevalent abroad, than in this commercial and too sordid country.

Did we address after this any personal commendation to Dr. Hope, we lay ourselves open to the imputation of indulging in panegyric. We shall say no more than that we are glad to find the work in his hands, and that we shall feel great pleasure in bearing ample testimony to its merits. We again regret that our crippled limits, and the late period of the quarter prevent our noticing it more fully, and particularly from referring to the letter-press.

## Figures and Tables

**Figure f1:**
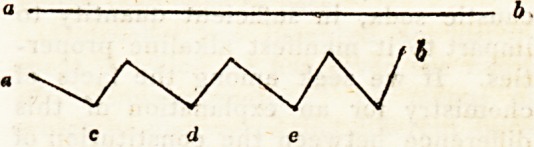


**Figure f2:**